# Supplementary material for: Design, Synthesis, and Evaluation of Novel 3-Carboranyl-1,8-Naphthalimide Derivatives as Potential Anticancer Agents
Source: Int J Mol Sci. 2021 Mar 9;22(5):2772. doi: 10.3390/ijms22052772 (PMC7967199; doi:10.3390/ijms22052772)
Supplement: Supplementary file 1 [file ijms-22-02772-s001.pdf]

# Design, synthesis, and evaluation of novel 3-carboranyl-1,8-naphthalimide derivatives as potential anticancer agents.

Sebastian Rykowski[a], Dorota Gurda-Woźna[b,1], Marta Orlicka-Płocka[b,1], Agnieszka Fedoruk-Wyszomirska[b], Małgorzata Giel-Pietraszuk[b], Eliza Wyszko[b], Aleksandra Kowalczyk[c], Paweł Stączek[c], Andrzej Bak[d], Agnieszka Kiliszek[b], Wojciech Rypniewski[b], Agnieszka B. Olejniczak[a,*]

[a]Institute of Medical Biology, Polish Academy of Sciences, 106 Lodowa St., Łódź 93-232, Poland

[b]Institute of Bioorganic Chemistry, Polish Academy of Sciences, 12/14 Z. Noskowskiego St., 61-704 Poznan, Poland

[c]Department of Molecular Microbiology, Faculty of Biology and Environmental Protection, University of Lodz, 12/16 Banacha St., Łódź 90-237, Poland

[d]Department of Chemistry, University of Silesia, Szkolna 9 St., 40-007 Katowice, Poland

# Table of contents


**Figure S1**. $^{1}$H NMR spectrum of **6** 7
**Figure S2**. $^{13}$C NMR spectrum of **6** 8
**Figure S3**. $^{11}$B NMR {$^{1}$H BB} spectrum of **6** 9
**Figure S4**. UV spectrum of **6** 10
**Figure S5**. IR spectrum of **6** 11
**Figure S6**. HPLC analysis of **6** 12
**Figure S7**. MS spectrum of **6** 13
**Figure S8**. $^{1}$H NMR spectrum of **7** 14
**Figure S9**. $^{13}$C NMR spectrum of **7** 15
**Figure S10**. $^{11}$B NMR {$^{1}$H BB} spectrum of **7** 16
**Figure S11**. UV spectrum of **7** 17
**Figure S12.** IR spectrum of **7** 18
**Figure S13**. HPLC analysis of **7** 19
**Figure S14.** MS spectrum of **7** 20
**Figure S15**. $^{1}$H NMR spectrum of **8** 21
**Figure S16**. $^{13}$C NMR spectrum of **8** 22
**Figure S17**. $^{11}$B NMR {$^{1}$H BB} spectrum of **8** 23
**Figure S18**. UV spectrum of **8** 24
**Figure S19**. IR spectrum of **8** 25
**Figure S20**. HPLC analysis of **8** 26
**Figure S21.** MS spectrum of **8** 27
**Figure S22.** HRMS spectrum of **8** 28
**Figure S23**. $^{1}$H NMR spectrum of **9** 29
**Figure S24**. $^{13}$C NMR spectrum of **9** 30
**Figure S25**. $^{11}$B NMR {$^{1}$H BB} spectrum of **9** 31
**Figure S26**. UV spectrum of **9** 32
**Figure S27**. IR spectrum of **9** 33
**Figure S28**. HPLC analysis of **9** 34
**Figure S29.** MS spectrum of **9** 35
**Figure S30**. $^{1}$H NMR spectrum of **10** 36
**Figure S31**. $^{13}$C NMR spectrum of **10** 37
**Figure S32**. $^{11}$B NMR {$^{1}$H BB} spectrum of **10** 38
**Figure S33**. UV spectrum of **10** 39
**Figure S34**. IR spectrum of **10** 40
**Figure S35**. HPLC analysis of **10** 41
**Figure S36**. MS spectrum of **10** 42
**Figure S37**. $^{1}$H NMR spectrum of **11** 43
**Figure S38**. $^{13}$C NMR spectrum of **11** 44
**Figure S39**. $^{11}$B NMR {$^{1}$H BB} spectrum of **11** 45
**Figure S40**. UV spectrum of **11** 46
**Figure S41**. IR spectrum of **11** 47
**Figure S42**. HPLC analysis of **11** 48
**Figure S43**. MS spectrum of **11** 49
**Figure S44**. $^{1}$H NMR spectrum of **15** 50
**Figure S45**. $^{13}$C NMR spectrum of **15** 51
**Figure S46**. $^{11}$B NMR {$^{1}$H BB} spectrum of **15** 52
**Figure S47**. UV spectrum of **15** 53
**Figure S48**. IR spectrum of **15** 54
**Figure S49**. HPLC analysis of **15** 55
**Figure S50**. MS spectrum of **15** 56

**Figure S51**. $^{1}H$ NMR spectrum of **16** 57
**Figure S52**. $^{13}C$ NMR spectrum of **16** 58
**Figure S53**. $^{11}B$ NMR {$^{1}H$ BB} spectrum of **16** 59
**Figure S54**. UV spectrum of **16** 60
**Figure S55**. IR spectrum of **16** 61
**Figure S56**. HPLC analysis of **16** 62
**Figure S57**. MS spectrum of **16** 63
**Figure S58**. $^{1}H$ NMR spectrum of **17** 64
**Figure S59**. $^{13}C$ NMR spectrum of **17** 65
**Figure S60**. $^{11}B$ NMR {$^{1}H$ BB} spectrum of **17** 66
**Figure S61**. UV spectrum of **17** 67
**Figure S62**. IR spectrum of **17** 68
**Figure S63**. HPLC analysis of **17** 69
**Figure S64**. MS spectrum of **17** 70
**Figure S65**. $^{1}H$ NMR spectrum of **18** 71
**Figure S66**. $^{13}C$ NMR spectrum of **18** 72
**Figure S67**. $^{11}B$ NMR {$^{1}H$ BB} spectrum of **18** 73
**Figure S68**. UV spectrum of **18** 74
**Figure S69**. IR spectrum of **18** 75
**Figure S70**. HPLC analysis of **18** 76
**Figure S71**. MS spectrum of **18** 77
**Figure S72**. $^{1}H$ NMR spectrum of **19** 78
**Figure S73**. $^{13}C$ NMR spectrum of **19** 79
**Figure S74**. $^{11}B$ NMR {$^{1}H$ BB} spectrum of **19** 80
**Figure S75**. UV spectrum of **19** 81
**Figure S76**. IR spectrum of **19** 82
**Figure S77**. HPLC analysis of **19** 83
**Figure S78**. MS spectrum of **19** 84
**Figure S79**. $^{1}H$ NMR spectrum of **20** 85
**Figure S80**. $^{13}C$ NMR spectrum of **20** 86
**Figure S81**. $^{11}B$ NMR {$^{1}H$ BB} spectrum of **20** 87
**Figure S82**. UV spectrum of **20** 88
**Figure S83**. IR spectrum of **20** 89
**Figure S84**. HPLC analysis of **20** 90
**Figure S85**. MS spectrum of **20** 91
**Figure S86**. HRMS spectrum of **20** 92
**Figure S87**. $^{1}H$ NMR spectrum of **31** 93
**Figure S88**. $^{13}C$ NMR spectrum of **31** 94
**Figure S89**. $^{11}B$ NMR {$^{1}H$ BB} spectrum of **31** 95
**Figure S90**. UV spectrum of **31** 96
**Figure S91**. IR spectrum of **31** 97
**Figure S92**. HPLC analysis of **31** 98
**Figure S93**. MS spectrum of **31** 99
**Figure S94**. $^{1}H$ NMR spectrum of **32** 100
**Figure S95**. $^{13}C$ NMR spectrum of **32** 101
**Figure S96**. $^{11}B$ NMR {$^{1}H$ BB} spectrum of **32** 102
**Figure S97**. UV spectrum of **32** 103
**Figure S98**. IR spectrum of **32** 104
**Figure S99**. HPLC analysis of **32** 105
**Figure S100**. MS spectrum of **32** 106
**Figure S101**. $^{1}H$ NMR spectrum of **33** 107

**Figure S102**. $^{13}C$ NMR spectrum of **33** 108
**Figure S103**. $^{11}B$ NMR {$^{1}H$ BB} spectrum of **33** 109
**Figure S104**. UV spectrum of **33** 110
**Figure S105**. IR spectrum of **33** 111
**Figure S106**. HPLC analysis of **33** 112
**Figure S107**. MS spectrum of **33** 113
**Figure S108**. $^{1}H$ NMR spectrum of **34** 114
**Figure S109**. $^{13}C$ NMR spectrum of **34** 115
**Figure S110**. $^{11}B$ NMR {$^{1}H$ BB} spectrum of **34** 116
**Figure S111**. UV spectrum of **34** 117
**Figure S112**. IR spectrum of **34** 118
**Figure S113**. HPLC analysis of **34** 119
**Figure S114**. MS spectrum of **34** 120
**Figure S115**. $^{1}H$ NMR spectrum of **35** 121
**Figure S116**. $^{13}C$ NMR spectrum of **35** 122
**Figure S117**. $^{11}B$ NMR {$^{1}H$ BB} spectrum of **35** 123
**Figure S118**. UV spectrum of **35** 124
**Figure S119**. IR spectrum of **35** 125
**Figure S120**. HPLC analysis of **35** 126
**Figure S121**. MS spectrum of **35** 127
**Figure S122**. HRMS spectrum of **35** 128
**Figure S123**. $^{1}H$ NMR spectrum of **36** 1296
**Figure S124**. $^{13}C$ NMR spectrum of **36** 130
**Figure S125**. $^{11}B$ NMR {$^{1}H$ BB} spectrum of **36** 131
**Figure S126**. UV spectrum of **36** 132
**Figure S127**. IR spectrum of **36** 133
**Figure S128**. HPLC analysis of **36** 134
**Figure S129** MS spectrum of **36** 135
**Figure S130**. $^{1}H$ NMR spectrum of **39** 136
**Figure S131**. $^{13}C$ NMR spectrum of **39** 137
**Figure S132**. $^{11}B$ NMR {$^{1}H$ BB} spectrum of **39** 138
**Figure S133**. UV spectrum of **39** 139
**Figure S134**. IR spectrum of **39** 140
**Figure S135**. HPLC analysis of **39** 141
**Figure S136**. MS spectrum of **39** 142
**Figure S137**. $^{1}H$ NMR spectrum of **40** 143
**Figure S138**. $^{13}C$ NMR spectrum of **40** 144
**Figure S139**. $^{11}B$ NMR {$^{1}H$ BB} spectrum of **40** 145
**Figure S140**. UV spectrum of **40** 146
**Figure S141**. IR spectrum of **40** 147
**Figure S142**. HPLC analysis of **40** 148
**Figure S143**. MS spectrum of **40** 149
**Figure S144**. HRMS spectrum of **40** 150
**Figure S145**. $^{1}H$ NMR spectrum of **41** 151
**Figure S146**. $^{13}C$ NMR spectrum of **41** 152
**Figure S147**. IR spectrum of **41** 153
**Figure S148**. UV spectrum of **41** 1540
**Figure S149**. IR spectrum of **41** 155
**Figure S150**. HPLC analysis of **41** 156
**Figure S151**. MS spectrum of **41** 157
**Figure S152**. $^{1}H$ NMR spectrum of **42** 158

**Figure S153**. $^{13}C$ NMR spectrum of **42** 159
**Figure S154**. IR spectrum of **42** 160
**Figure S155**. UV spectrum of **42** 161
**Figure S156**. IR spectrum of **42** 162
**Figure S157**. HPLC analysis of **42** 163
**Figure S158**. MS spectrum of **42** 166
**Figure S159**. Influence of compound **6** on cell cycle distribution in HepG2 cells 165
**Figure S160**. ROS production in HepG2 cells after 24 h incubation with compound **6-11**, **16-20**, **32**, **39-42** 166
**Figure S161.** Autofluorescence analysis of the tested compounds in HepG2 cells 167
**Figure S162.** Flow cytometry analysis of apoptosis/necrosis in HepG2 cells after cell toxicity induction with compound **6** 168
**Table 1S**. Percentage distribution of early and late apoptotic and necrotic cells assessed by flow cytometry 169
**Figure S163.** Flow cytometry analysis of apoptosis/necrosis in HepG2 cells after cell toxicity induction with compound **31** 170
**Figure S164**. Flow cytometry analysis of autophagy in HepG2 cells after cell toxicity induction with compound **8** 171
**Figure S1651**. Flow cytometry analysis of lipid peroxidation in HepG2 cells after cell toxicity induction with compound **15** 172
**Figure S166**. Human Topoisomerase II$\alpha$ relaxation assay 173
**Figure S167**. Melting curves of ct-DNA upon addition of **6-11** 174
**Figure S168**. Melting curves of ct-DNA upon addition of **15-20** 175
**Figure S169**. Melting curves of ct-DNA upon addition of **31-36** 176
**Figure S170**. Melting curves of ct-DNA upon addition of **39-42** 177
**Figure S171**. Melting curves of ct-DNA upon addition of mitonafide and pinafide 178
**Figure S172**. Changes in the CD spectrum of ct-DNA upon addition of mitonafide and pinafide 179
**Figure S173**. Changes in the CD spectrum of ct-DNA upon addition of **6** and **7** 180
**Figure S174**. Changes in the CD spectrum of ct-DNA upon addition of **8** and **9** 181
**Figure S175**. Changes in the CD spectrum of ct-DNA upon addition of **10** and **11** 182
**Figure S176**. Changes in the CD spectrum of ct-DNA upon addition of **15** and **16** 183
**Figure S177**. Changes in the CD spectrum of ct-DNA upon addition of **17** and **18** 184
**Figure S178**. Changes in the CD spectrum of ct-DNA upon addition of **19** and **20** 185
**Figure S179**. Changes in the CD spectrum of ct-DNA upon addition of **31** and **32** 186
**Figure S180**. Changes in the CD spectrum of ct-DNA upon addition of **33** and **34** 187
**Figure S181**. Changes in the CD spectrum of ct-DNA upon addition of **35** and **36** 188
**Figure S182**. Changes in the CD spectrum of ct-DNA upon addition of **39** and **40** 189
**Figure S183**. Changes in the CD spectrum of ct-DNA upon addition of **41** and **42** 190
**Figure S184**. UV-vis absorption spectra of compound **6** (20 μM) in the presence of increasing amount of cf-DNA (0-15 μM) (left). The plot of $A_0/A-A_0$ versus 1/[DNA] yielded the binding constant (right) 191
**Figure S185**. UV-vis absorption spectra of compound **7** (20 μM) in the presence of increasing amount of cf-DNA (0-15 μM) (left). The plot of $A_0/A-A_0$ versus 1/[DNA] yielded the binding constant (right) 192
**Figure S186**. UV-vis absorption spectra of compound **8** (20 μM) in the presence of increasing amount of ct-DNA (0-15 μM) (left). The plot of $A_0/A-A_0$ versus 1/[DNA] yielded the binding constant (right) 193
**Figure S187**. UV-vis absorption spectra of compound **9** (20 μM) in the presence of increasing amount of ct-DNA (0-15 μM) (left). The plot of $A_0/A-A_0$ versus 1/[DNA] yielded the binding constant (right) 194

**Figure S188**. UV-vis absorption spectra of compound **10** (20 μM) in the presence of increasing amount of ct-DNA (0-15 μM) (left). The plot of $A_0/A-A_0$ versus 1/[DNA] yielded the binding constant (right) 195
**Figure S189**. UV-vis absorption spectra of compound **11** (20 μM) in the presence of increasing amount of ct-DNA (0-15 μM) (left). The plot of $A_0/A-A_0$ versus 1/[DNA] yielded the binding constant (right) 196
**Figure S190**. UV-vis absorption spectra of compound **15** (20 μM) in the presence of increasing amount of ct-DNA (0-15 μM) (left). The plot of $A_0/A-A_0$ versus 1/[DNA] yielded the binding constant (right) 197
**Figure S191**. UV-vis absorption spectra of compound **16** (20 μM) in the presence of increasing amount of ct-DNA (0-15 μM) (left). The plot of $A_0/A-A_0$ versus 1/[DNA] yielded the binding constant (right) 198
**Figure S192**. UV-vis absorption spectra of compound **17** (20 μM) in the presence of increasing amount of ct-DNA (0-15 μM) (left). The plot of $A_0/A-A_0$ versus 1/[DNA] yielded the binding constant (right) 199
**Figure S193**. UV-vis absorption spectra of compound **18** (20 μM) in the presence of increasing amount of ct-DNA (0-15 μM) (left). The plot of $A_0/A-A_0$ versus 1/[DNA] yielded the binding constant (right) 200
**Figure S194**. UV-vis absorption spectra of compound **19** (20 μM) in the presence of increasing amount of ct-DNA (0-15 μM) (left). The plot of $A_0/A-A_0$ versus 1/[DNA] yielded the binding constant (right) 201
**Figure S195**. UV-vis absorption spectra of compound **20** (20 μM) in the presence of increasing amount of ct-DNA (0-15 μM) (left). The plot of $A_0/A-A_0$ versus 1/[DNA] yielded the binding constant (right) 202
**Figure S196**. UV-vis absorption spectra of compound **33** (20 μM) in the presence of increasing amount of ct-DNA (0-15 μM) (left). The plot of $A_0/A-A_0$ versus 1/[DNA] yielded the binding constant (right) 203
**Figure S197**. UV-vis absorption spectra of compound **34** (20 μM) in the presence of increasing amount of ct-DNA (0-15 μM) (left). The plot of $A_0/A-A_0$ versus 1/[DNA] yielded the binding constant (right) 204
**Figure S198**. UV-vis absorption spectra of compound **35** (20 μM) in the presence of increasing amount of ct-DNA (0-15 μM) (left). The plot of $A_0/A-A_0$ versus 1/[DNA] yielded the binding constant (right) 205
**Figure S199**. UV-vis absorption spectra of compound **36** (20 μM) in the presence of increasing amount of ct-DNA (0-15 μM) (left). The plot of $A_0/A-A_0$ versus 1/[DNA] yielded the binding constant (right) 206
**Figure S200**. UV-vis absorption spectra of compound **39** (20 μM) in the presence of increasing amount of ct-DNA (0-15 μM) (left). The plot of $A_0/A-A_0$ versus 1/[DNA] yielded the binding constant (right) 207
**Figure S201**. UV-vis absorption spectra of compound **40** (20 μM) in the presence of increasing amount of ct-DNA (0-15 μM) (left). The plot of $A_0/A-A_0$ versus 1/[DNA] yielded the binding constant (right) 208
**Figure S202**. UV-vis absorption spectra of mitonafide (20 μM) in the presence of increasing amount of ct-DNA (0-15 μM) (left). The plot of $A_0/A-A_0$ versus 1/[DNA] yielded the binding constant (right) 209
**Figure S203**. UV-vis absorption spectra of pinafide (20 μM) in the presence of increasing amount of ct-DNA (0-15 μM) (left). The plot of $A_0/A-A_0$ versus 1/[DNA] yielded the binding constant (right) 210
**Table 2S**. Crystallographic data 211

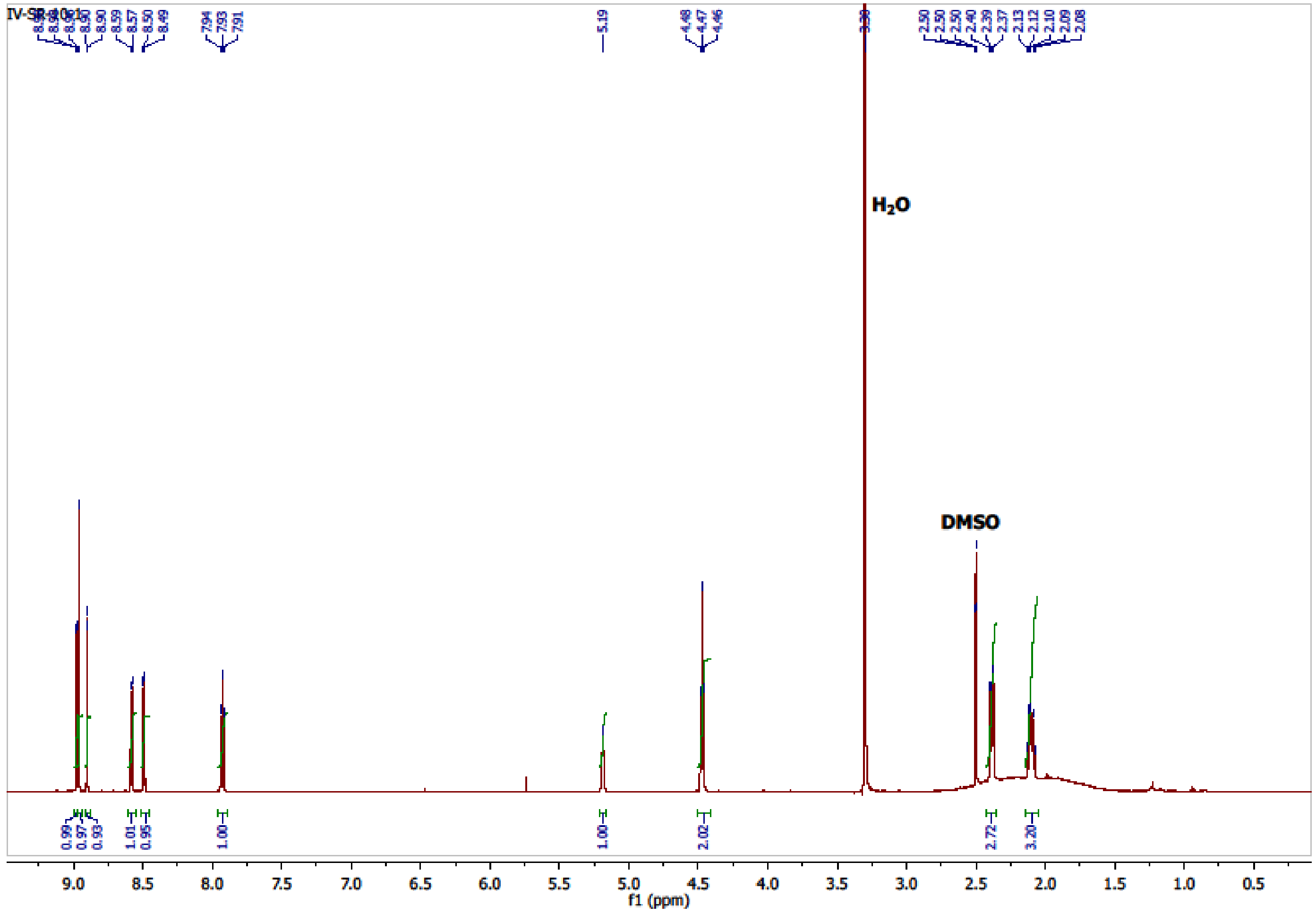


**Figure S1**. $^{1}H$ NMR spectrum of **6**.

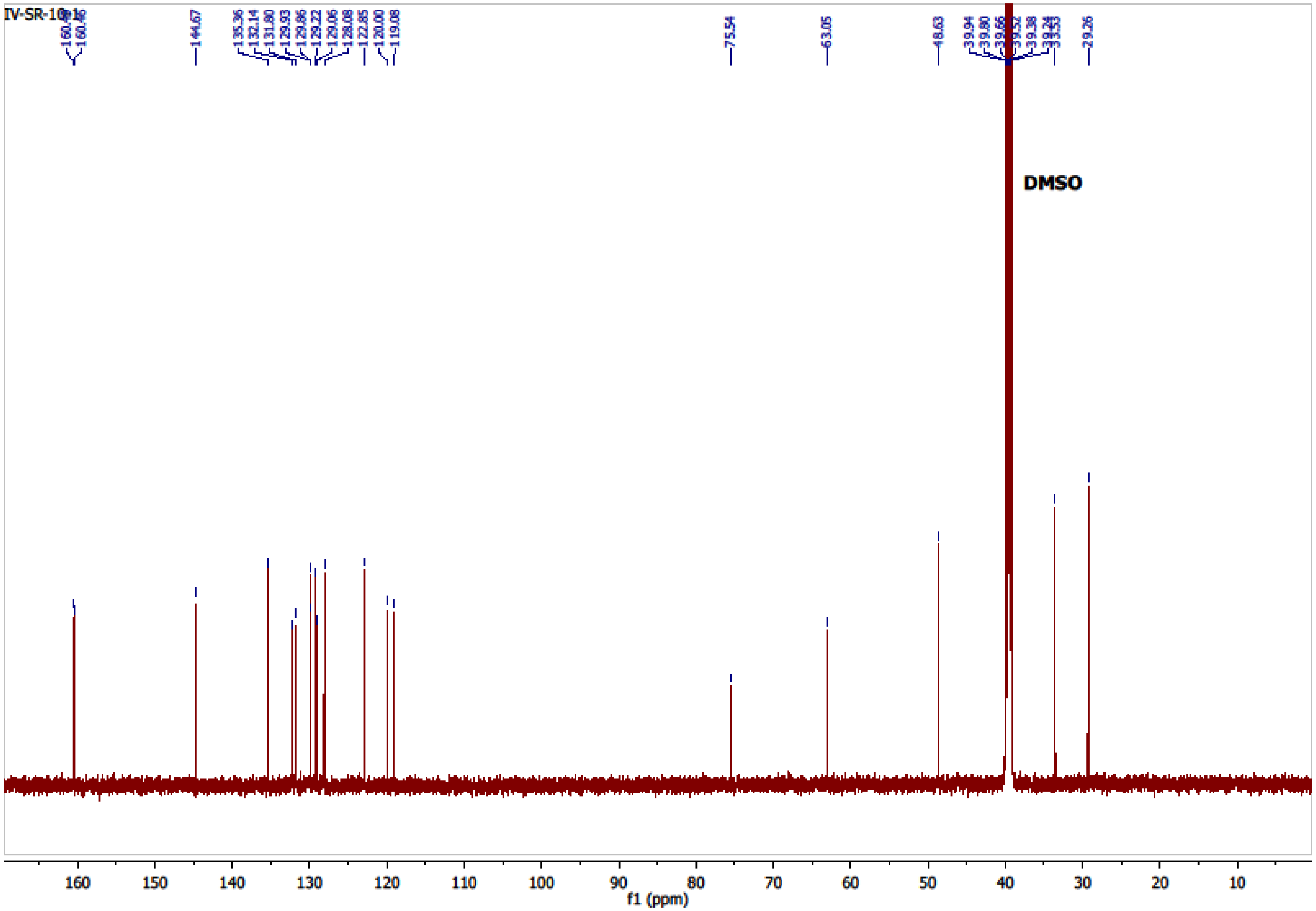


**Figure S2**. $^{13}C$ NMR spectrum of **6**.

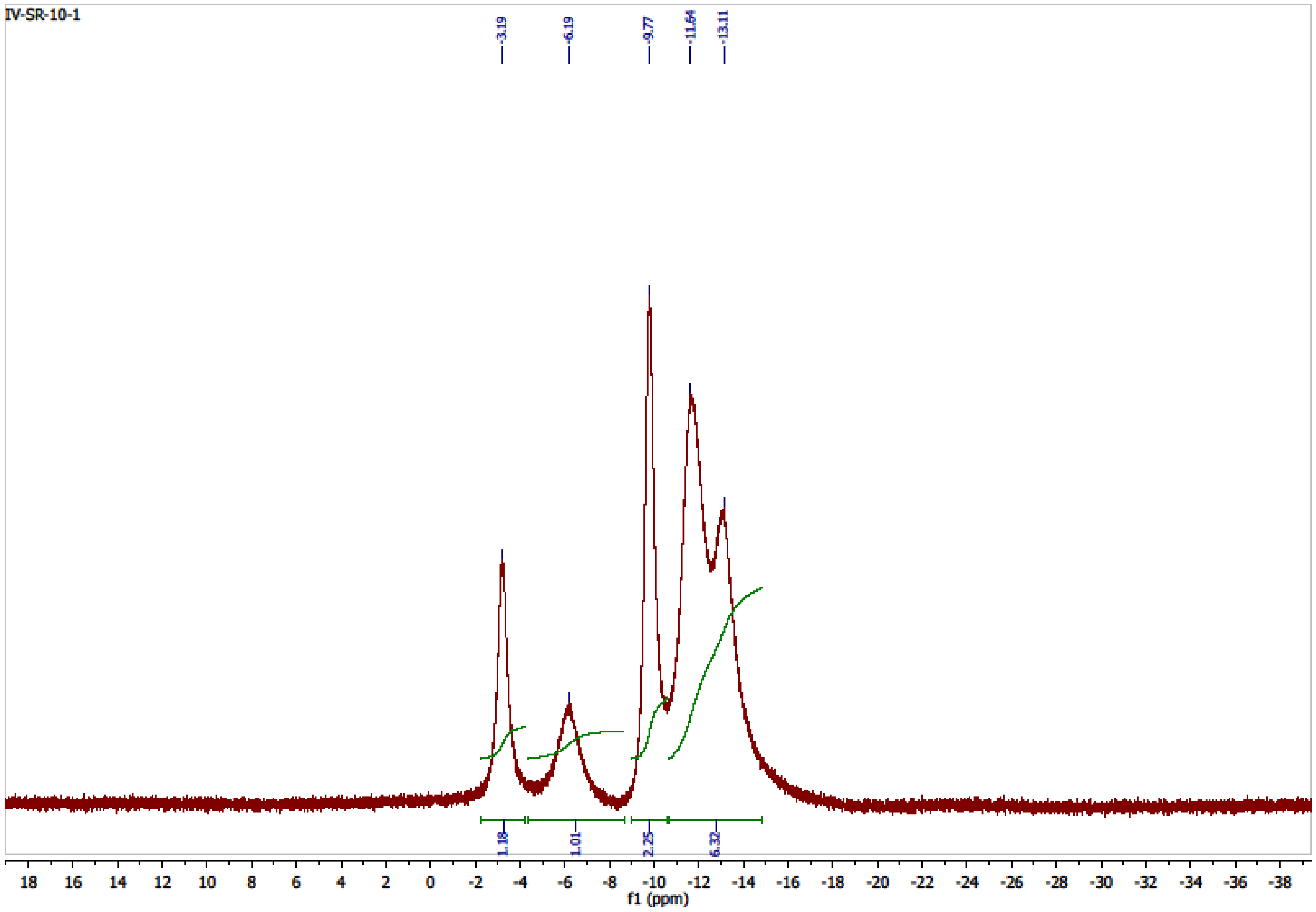


**Figure S3**. $^{11}B$ NMR {$^{1}H$ BB} spectrum of **6**.

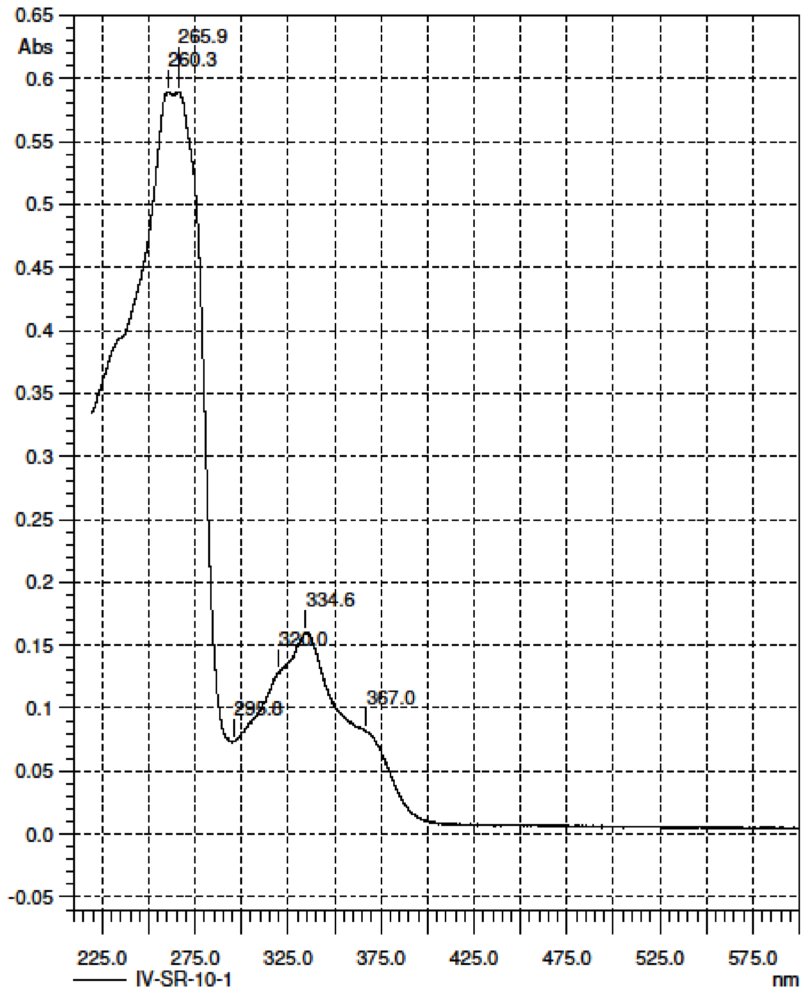


**Figure S4**. UV spectrum of **6**.

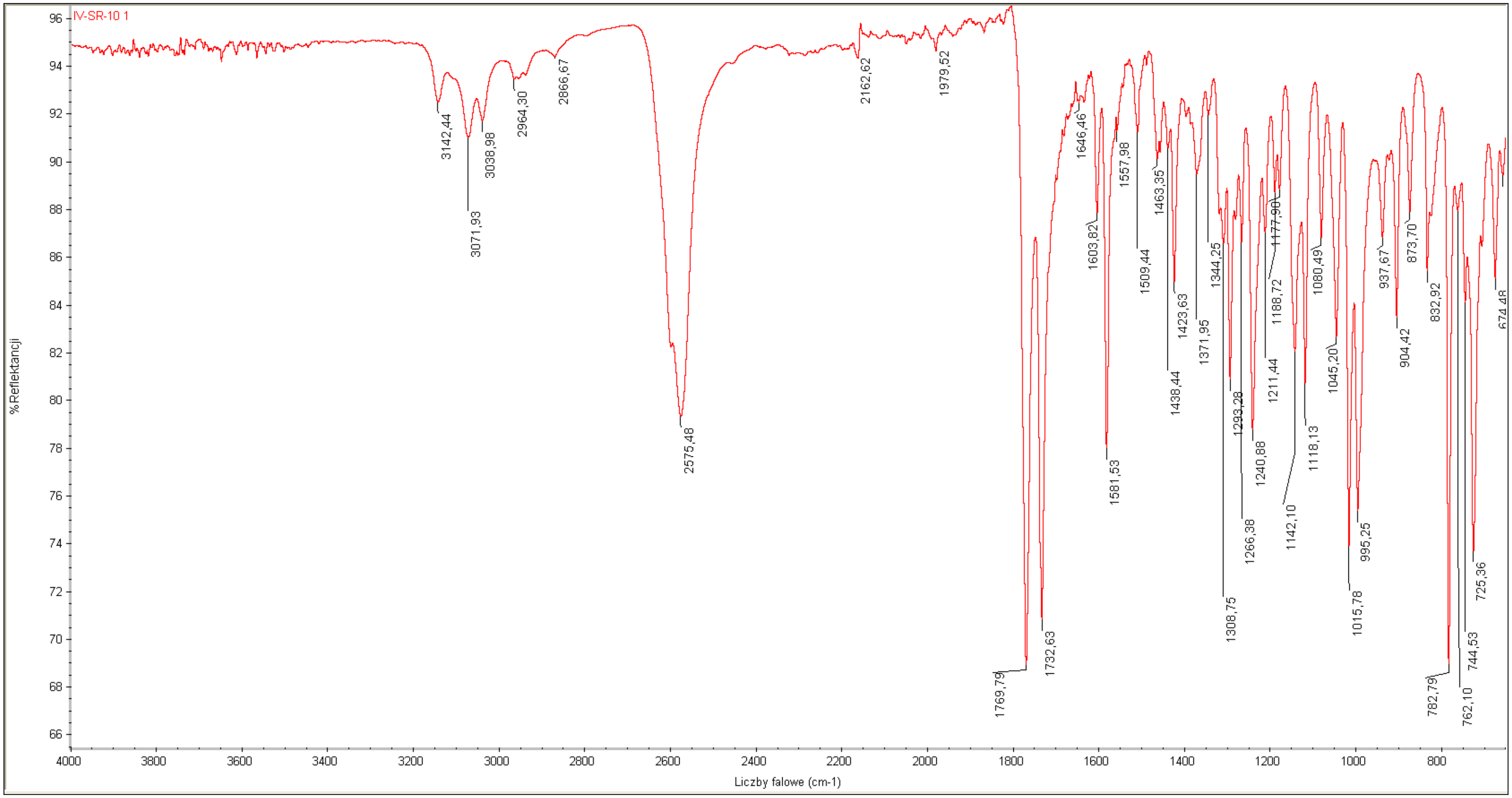


**Figure S5**. IR spectrum of **6**.

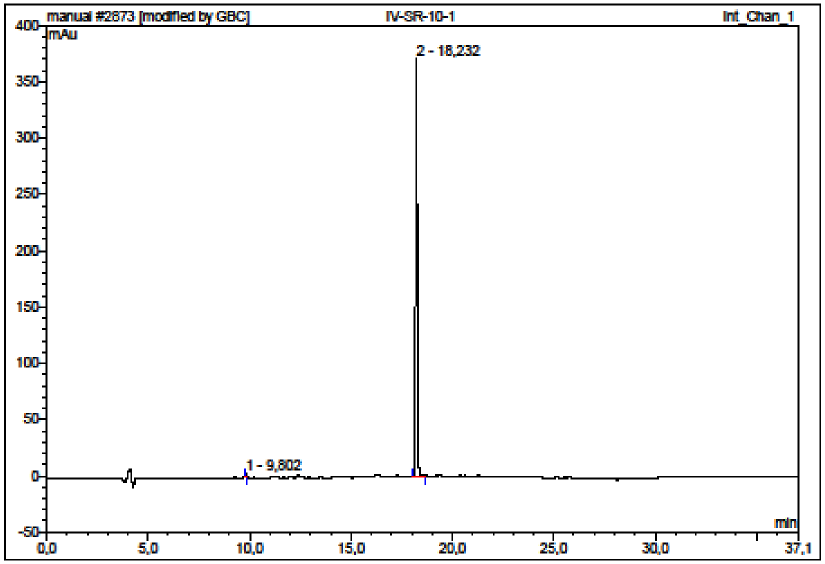


| No. | Ret.Time min | Peak Name | Height mAu | Area mAu*min | Rel.Area % | Amount | Type |
|---|---|---|---|---|---|---|---|
| 1 | 9,80 | n.a. | 2,965 | 0,240 | 0,63 | n.a. | BMB* |
| 2 | 18,23 | n.a. | 371,895 | 37,820 | 99,37 | n.a. | BMB |
| Total: | | | 374,860 | 38,060 | 100,00 | 0,000 | |

**Figure S6**. HPLC analysis of **6**.

Spectrum Name: IV-SR-10_1_PT
Start Ion: 400
End Ion: 600
Source: APCI + 10.0µA 400C
Capillary: 180V 300C Offset: 30V Span: 20V

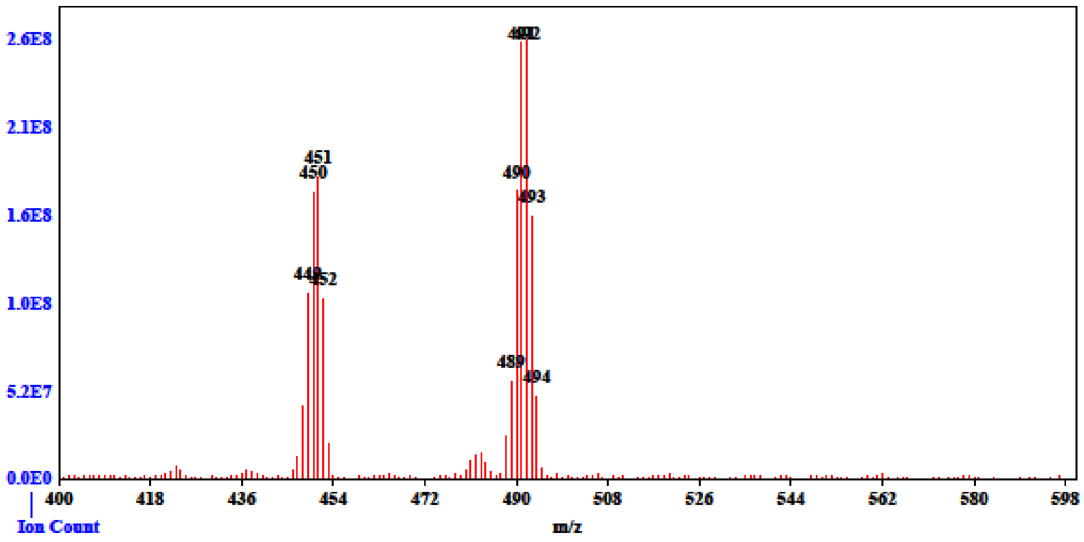


**Figure S7**. MS spectrum of **6**.

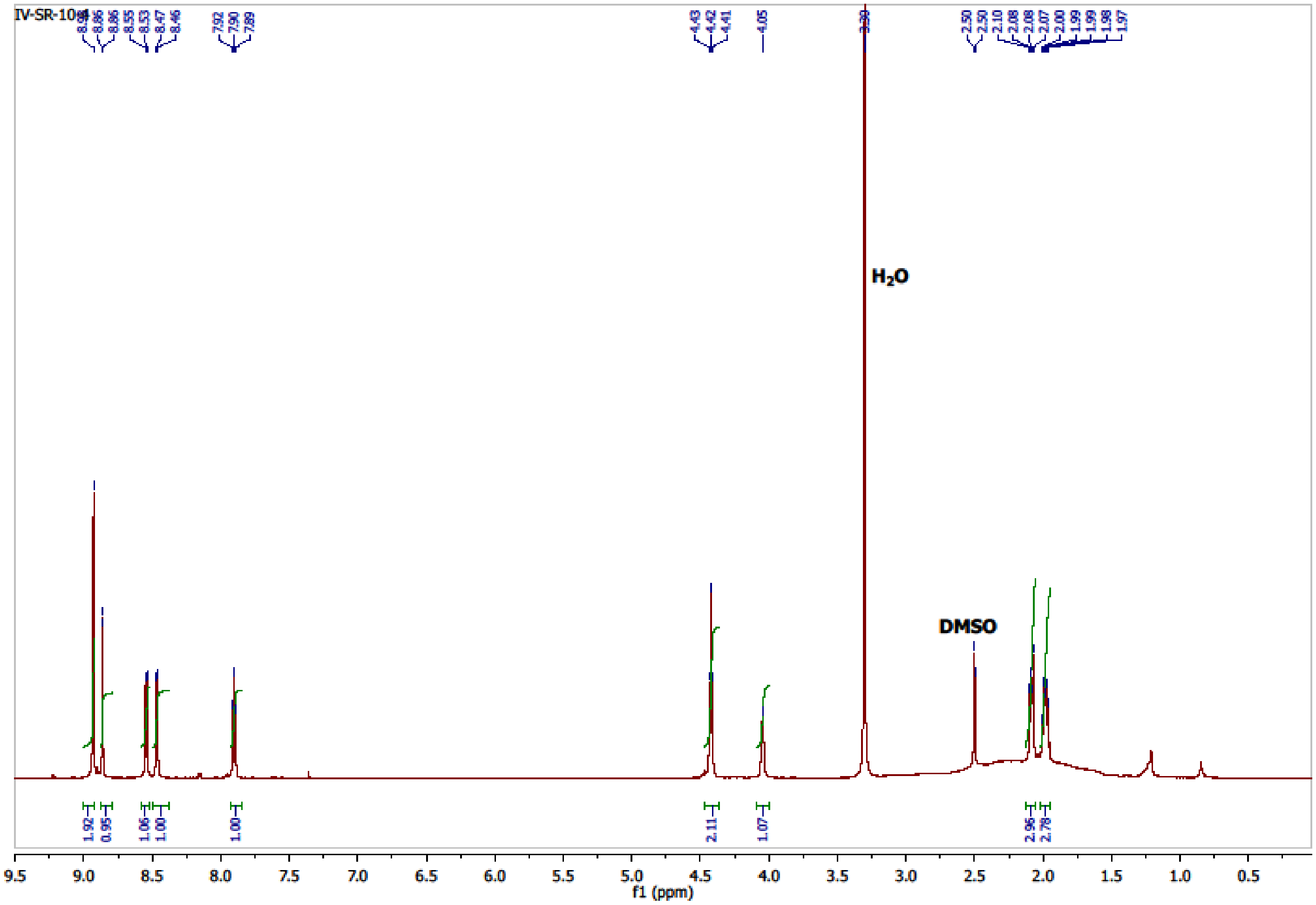


**Figure S8**. $^{1}$H NMR spectrum of **7**.

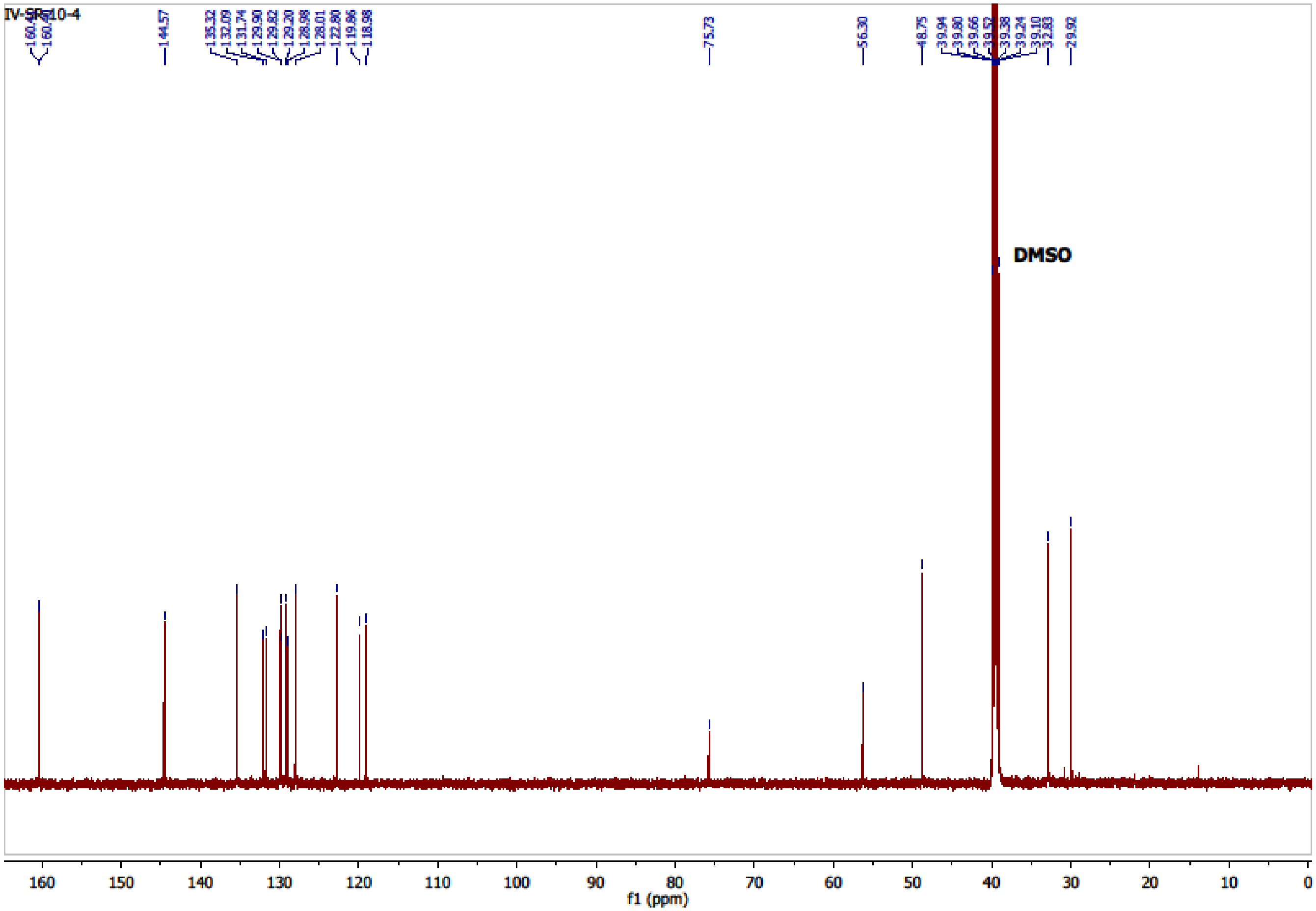


**Figure S9**. $^{13}$C NMR spectrum of **7**.

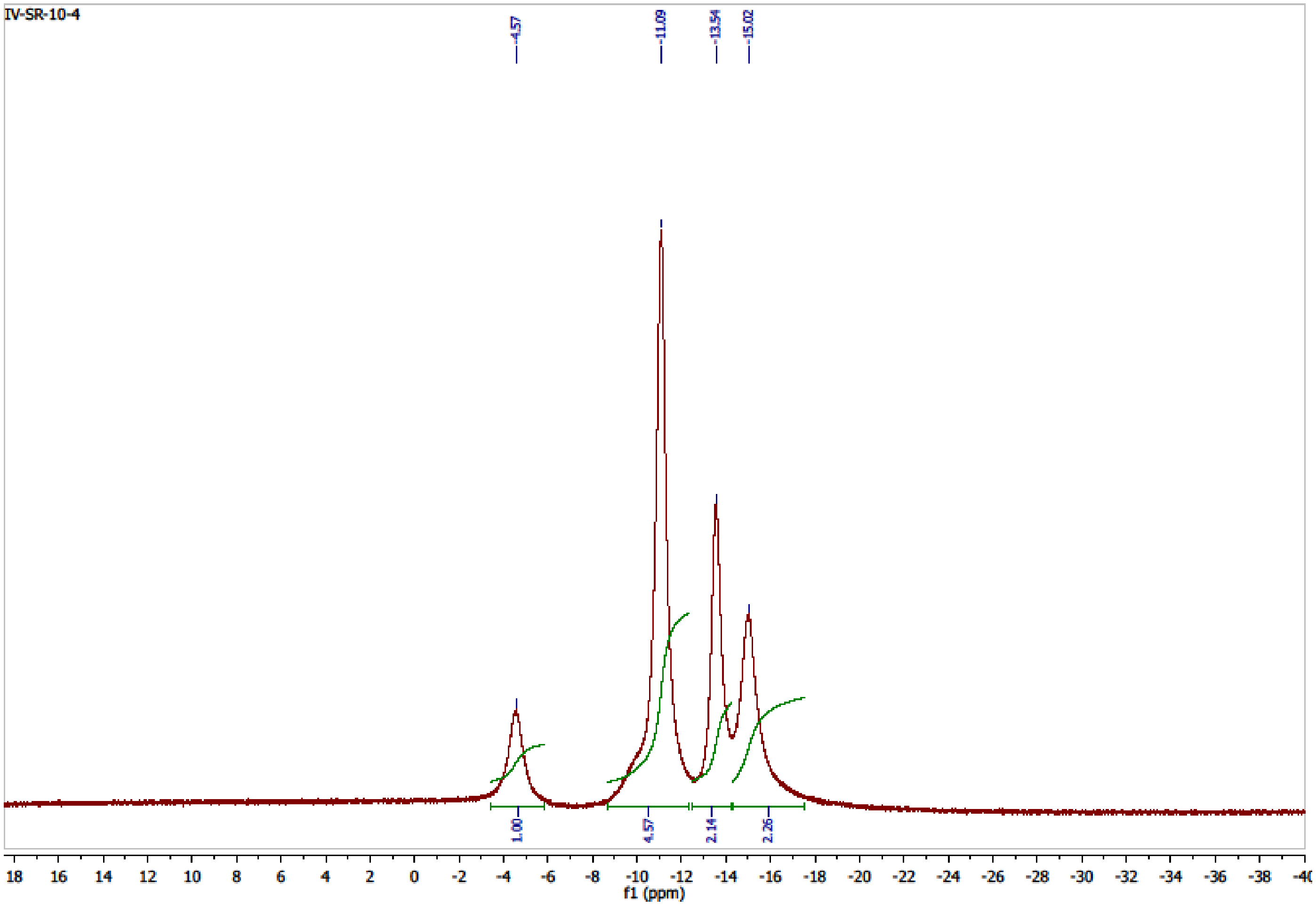


**Figure S10**. $^{11}B$ NMR {$^{1}H$ BB} spectrum of **7**.

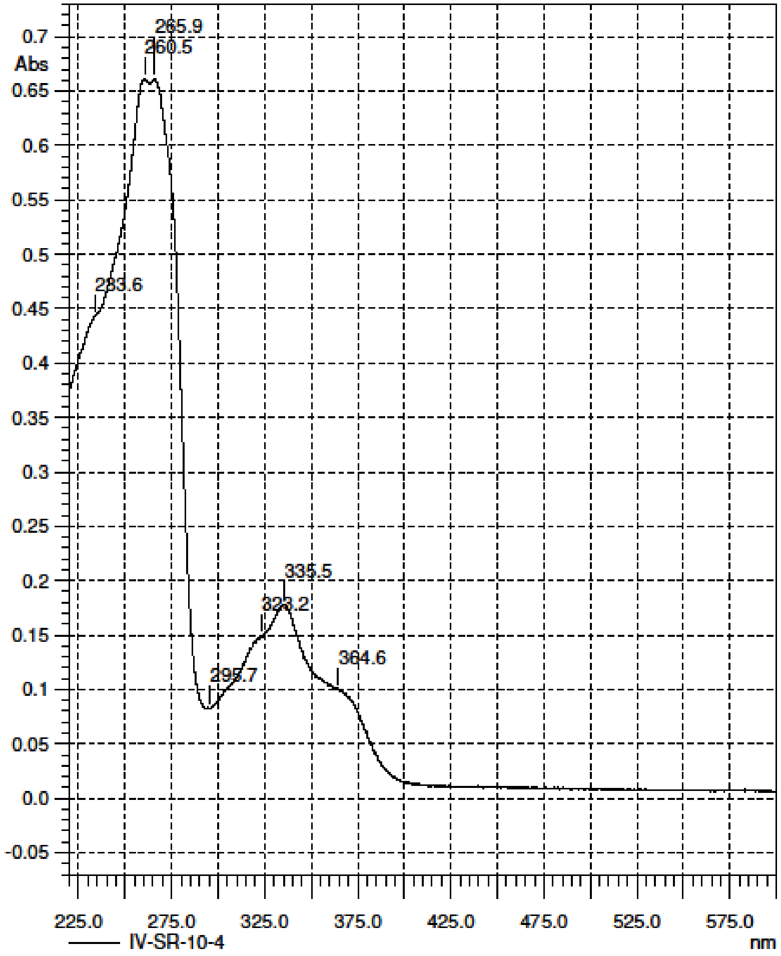


**Figure S11**. UV spectrum of **7**.

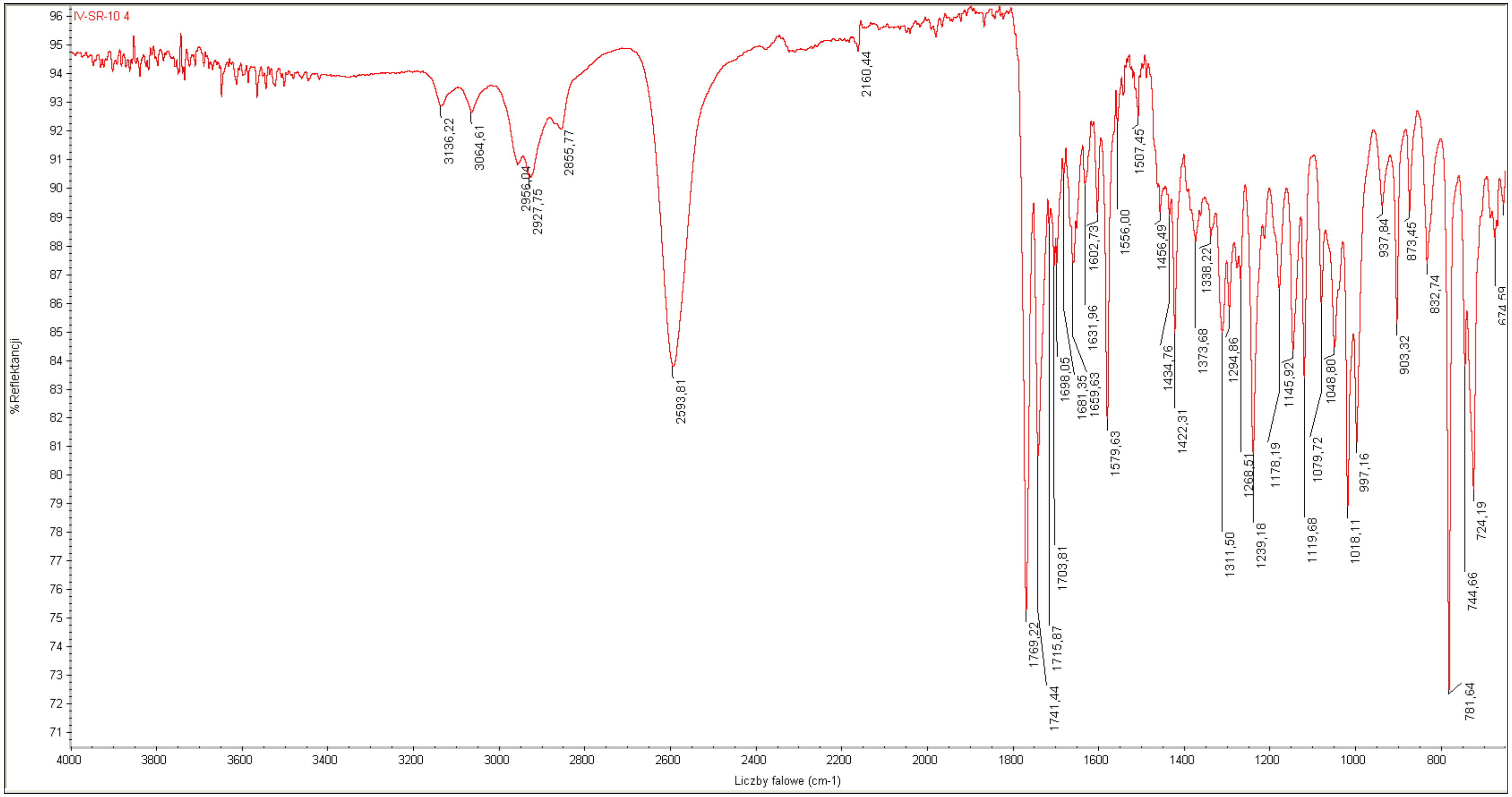


**Figure S12.** IR spectrum of **7**.

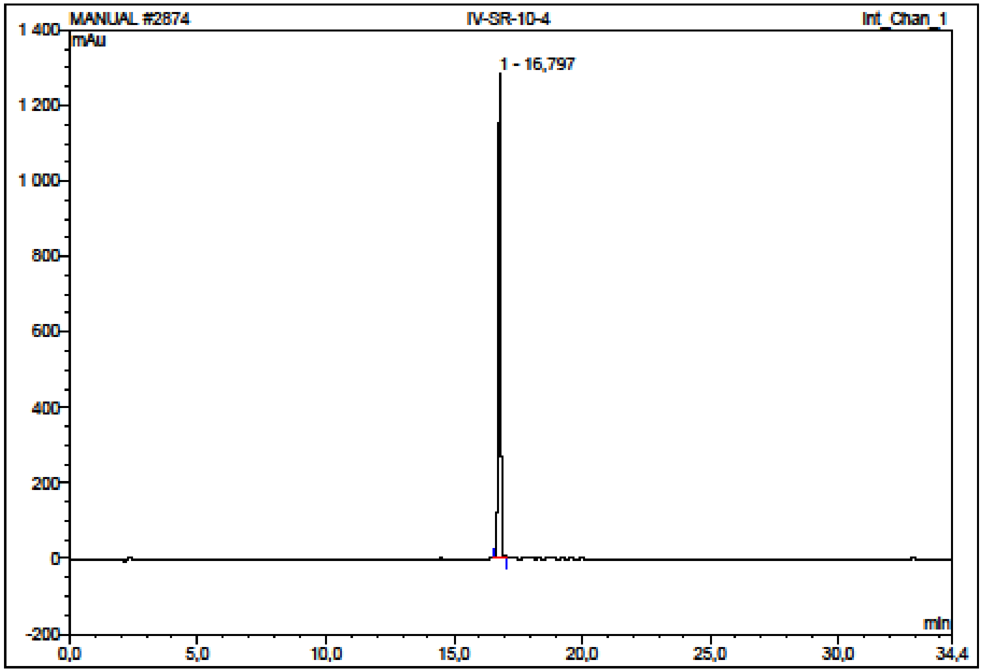


| No. | Ret.Time min | Peak Name | Height mAu | Area mAu*min | Rel.Area % | Amount | Type |
|---|---|---|---|---|---|---|---|
| 1 | 16,80 | n.a. | 1281,764 | 135,115 | 100,00 | n.a. | BMB |
| Total: | | | 1281,764 | 135,115 | 100,00 | 0,000 | |

**Figure S13**. HPLC analysis of **7**.

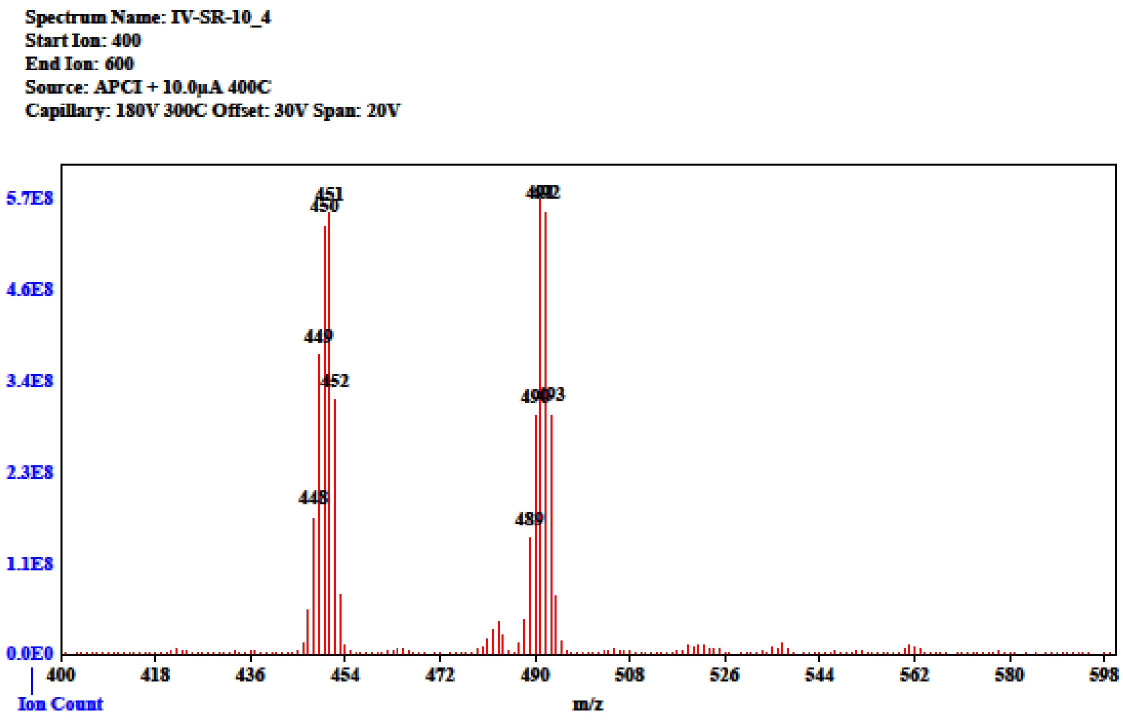


**Figure S14.** MS spectrum of **7**.

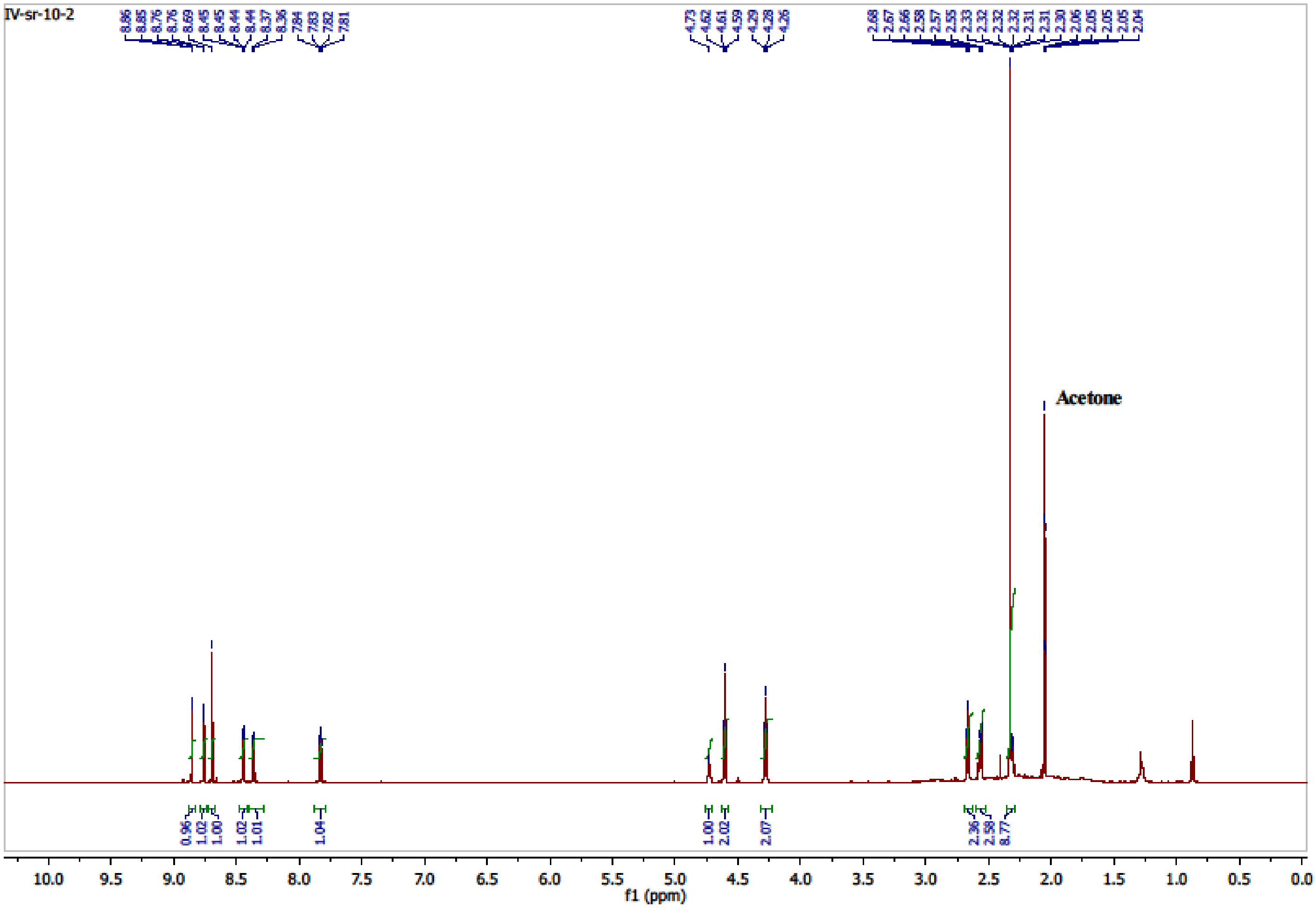


**Figure S15**. $^{1}$H NMR spectrum of **8**.

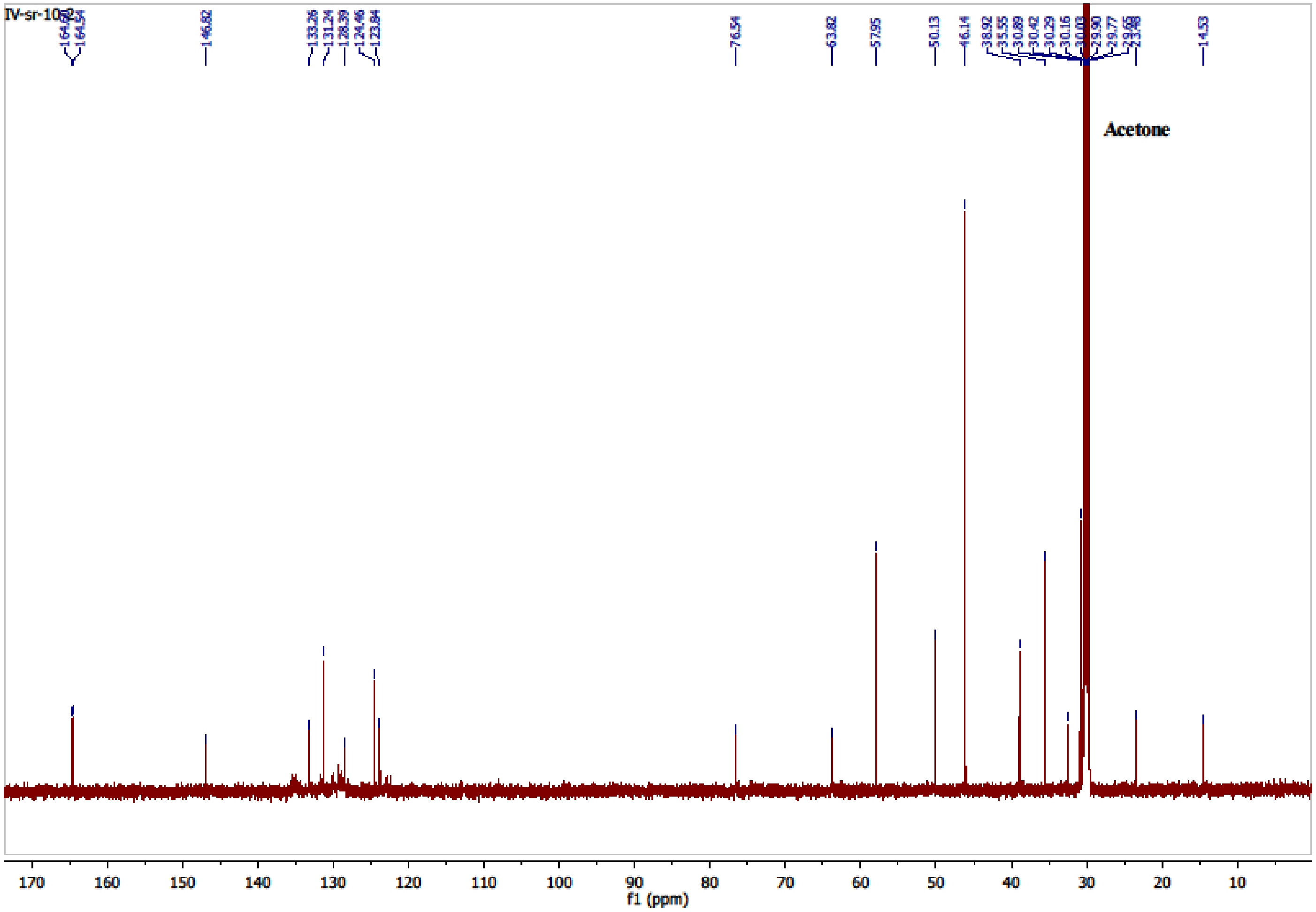


**Figure S16**. $^{13}$C NMR spectrum of **8**.

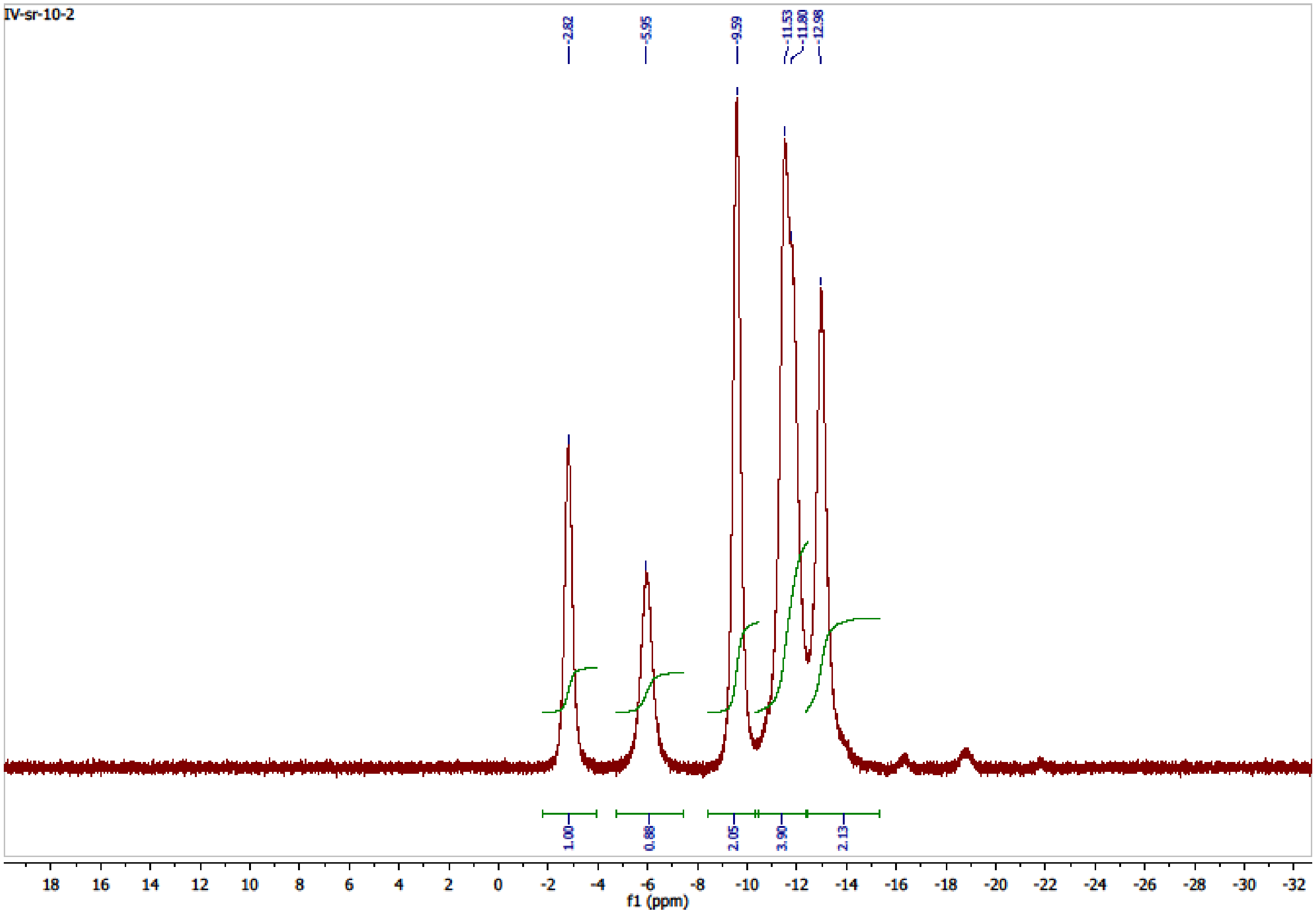


**Figure S17**. $^{11}B$ NMR {$^{1}H$ BB} spectrum of **8**.

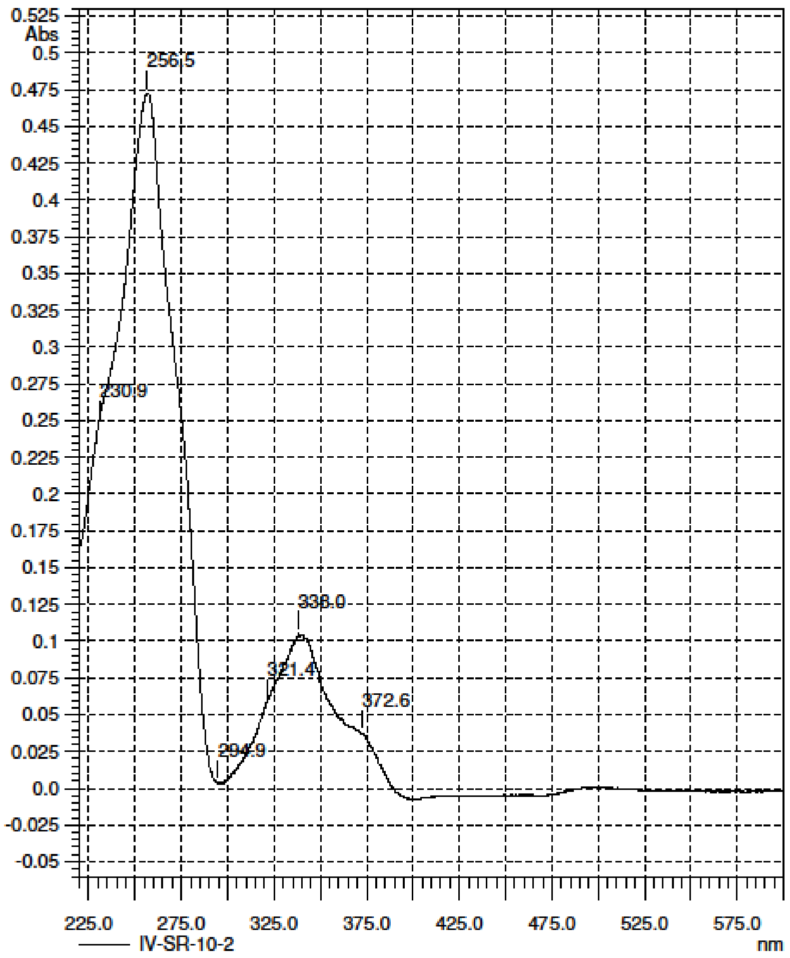


**Figure S18**. UV spectrum of **8**.

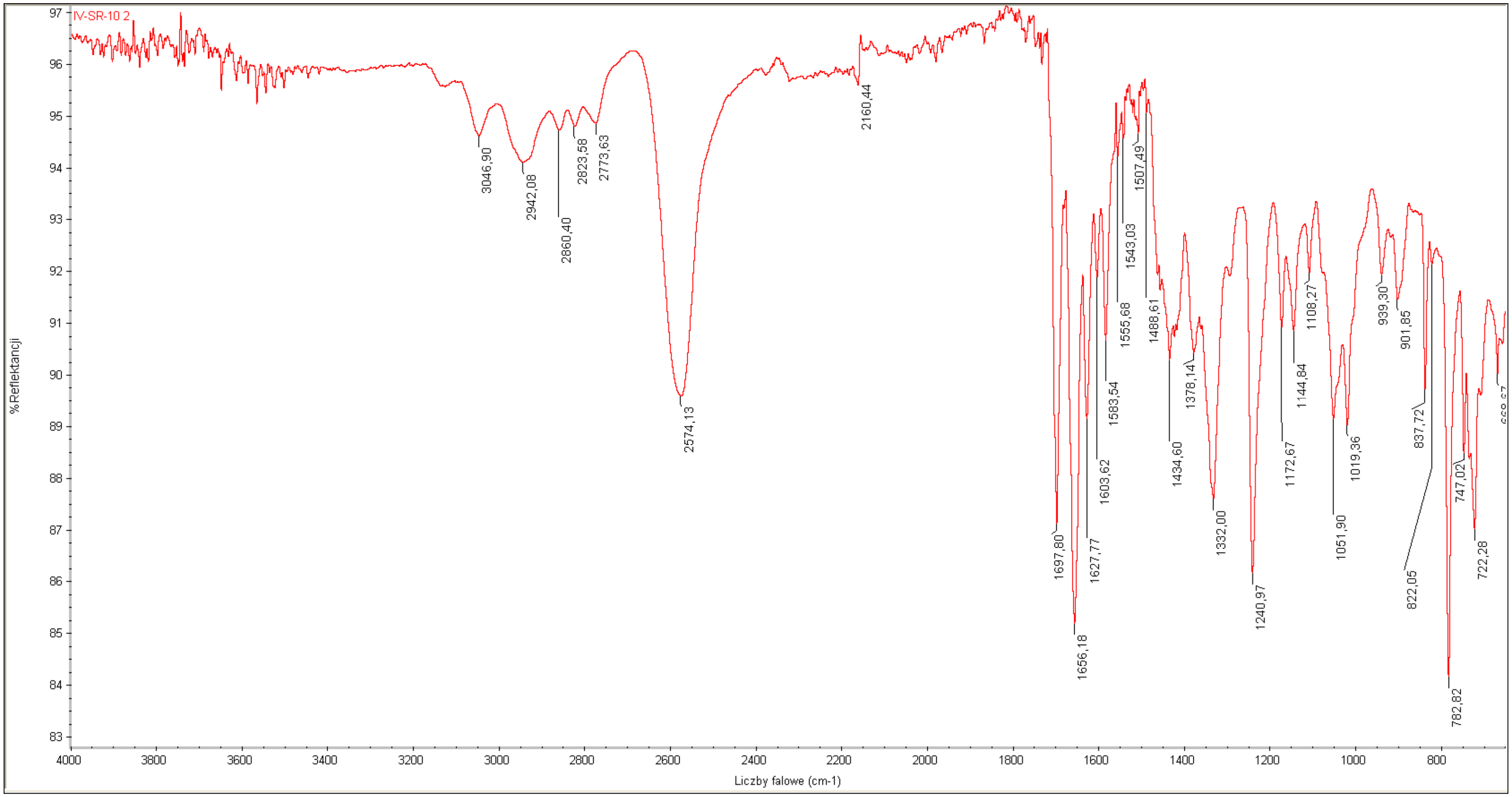


**Figure S19**. IR spectrum of **8**.

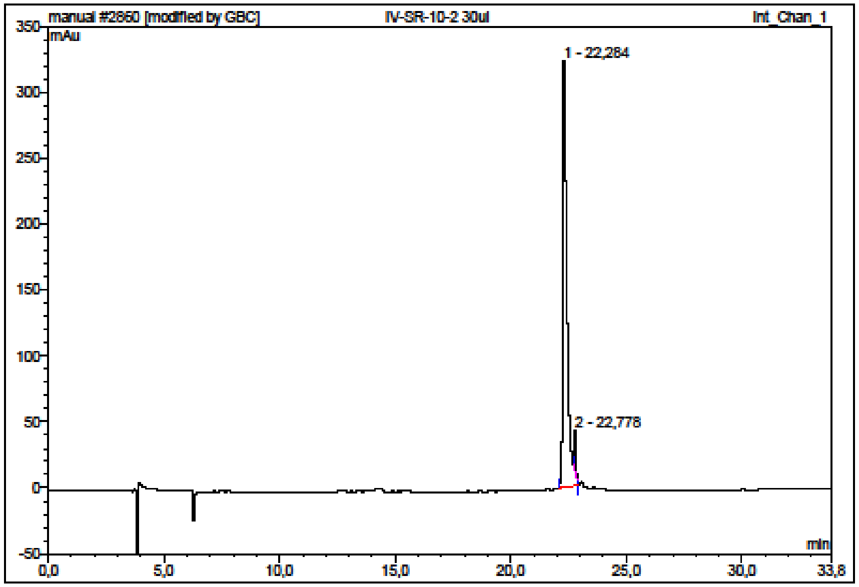


| No. | Ret.Time min | Peak Name | Height mAu | Area mAu*min | Rel.Area % | Amount | Type |
|---|---|---|---|---|---|---|---|
| 1 | 22,28 | n.a. | 324,260 | 69,327 | 96,50 | n.a. | BMB* |
| 2 | 22,78 | n.a. | 30,615 | 2,511 | 3,50 | n.a. | Rd* |
| Total: | | | 354,875 | 71,838 | 100,00 | 0,000 | |

**Figure S20**. HPLC analysis of **8**.

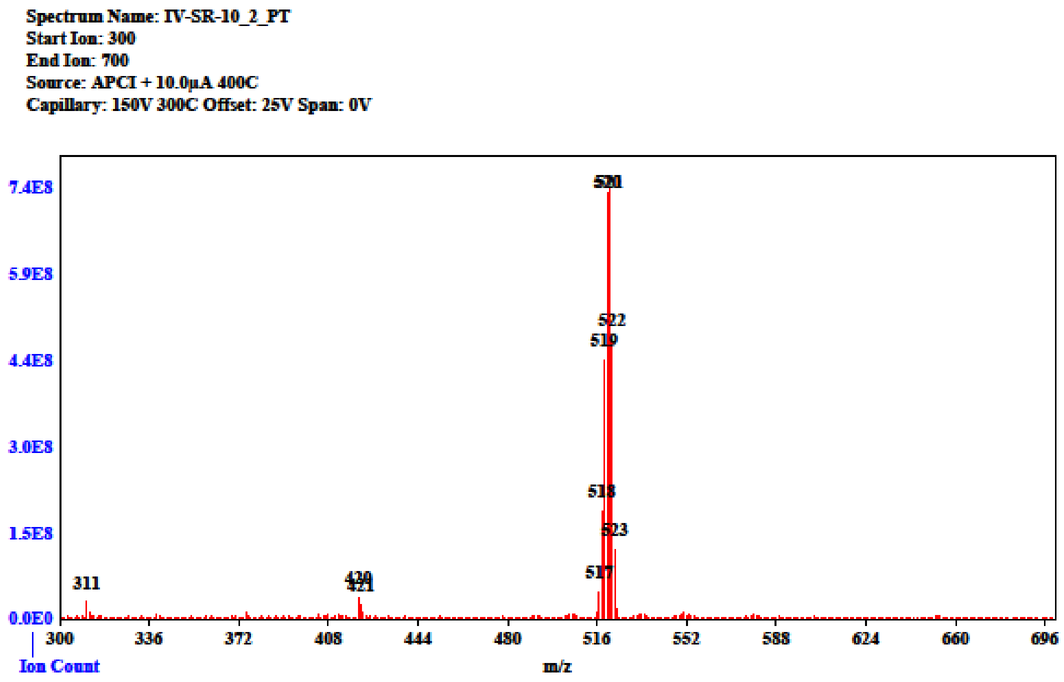


**Figure S21.** MS spectrum of **8**.

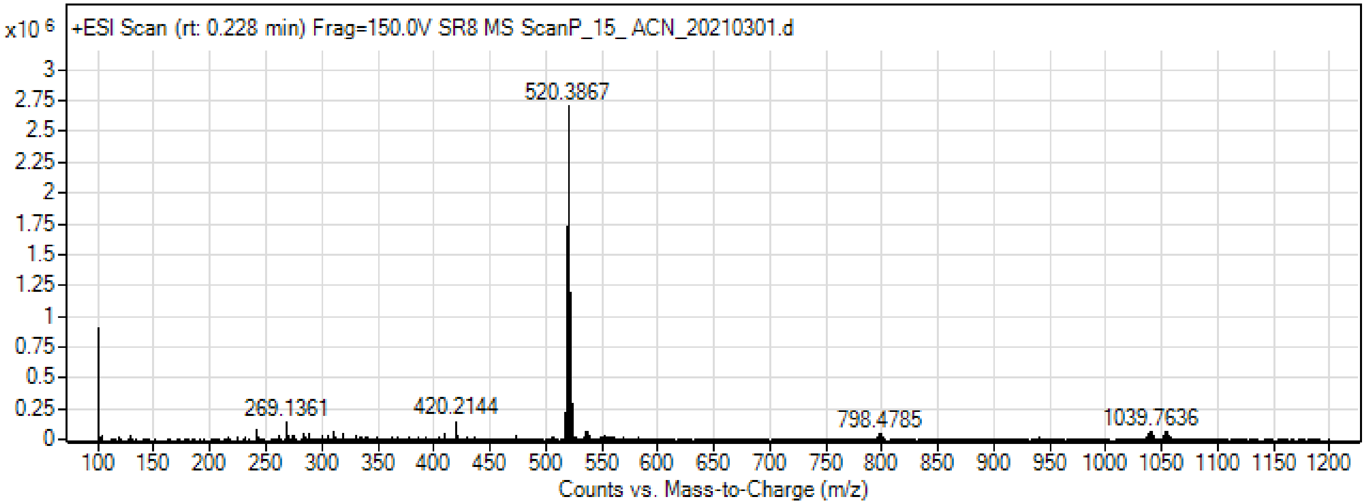


**Figure S22.** HRMS spectrum of **8**.

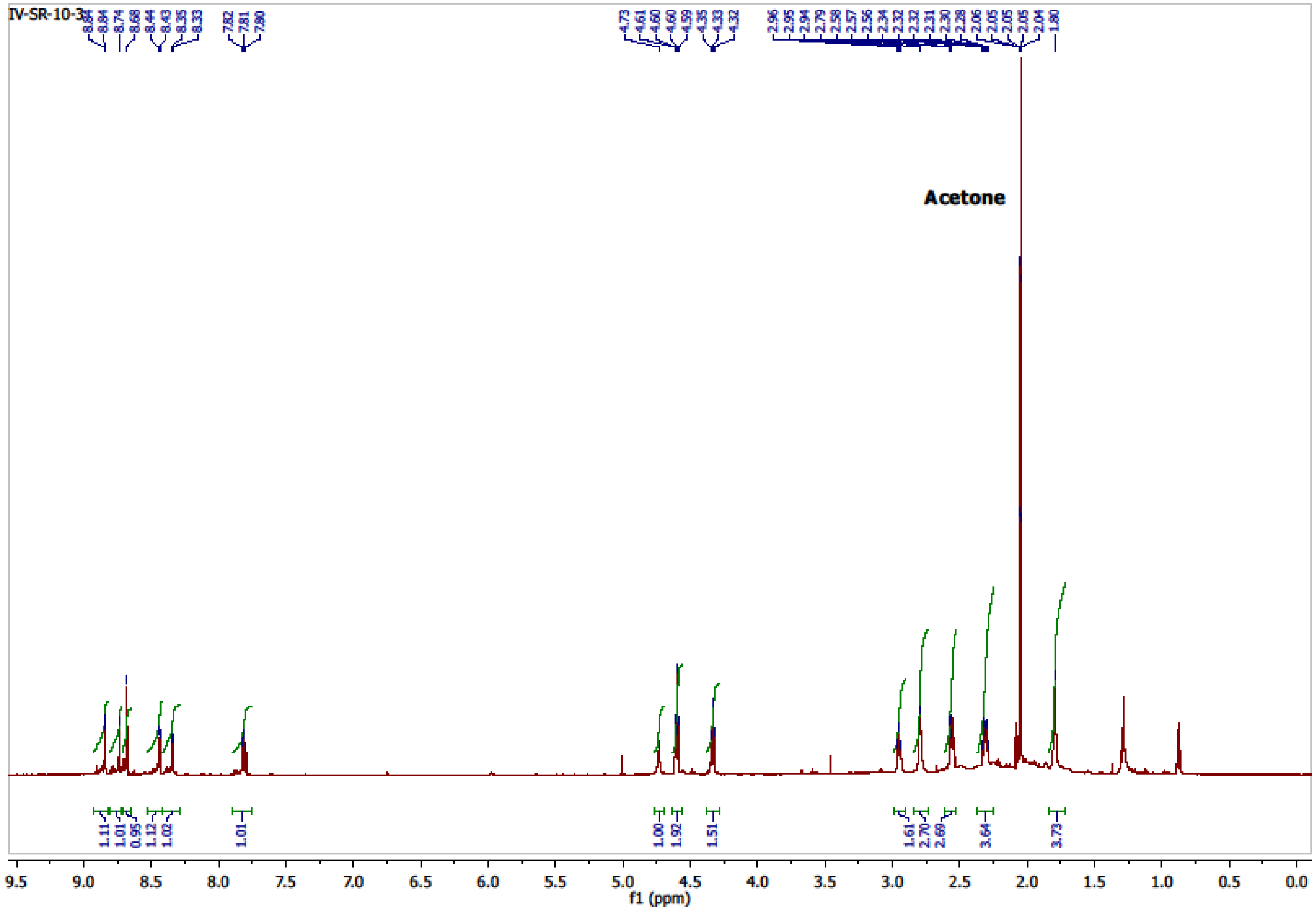


**Figure S23**. ¹H NMR spectrum of **9**.

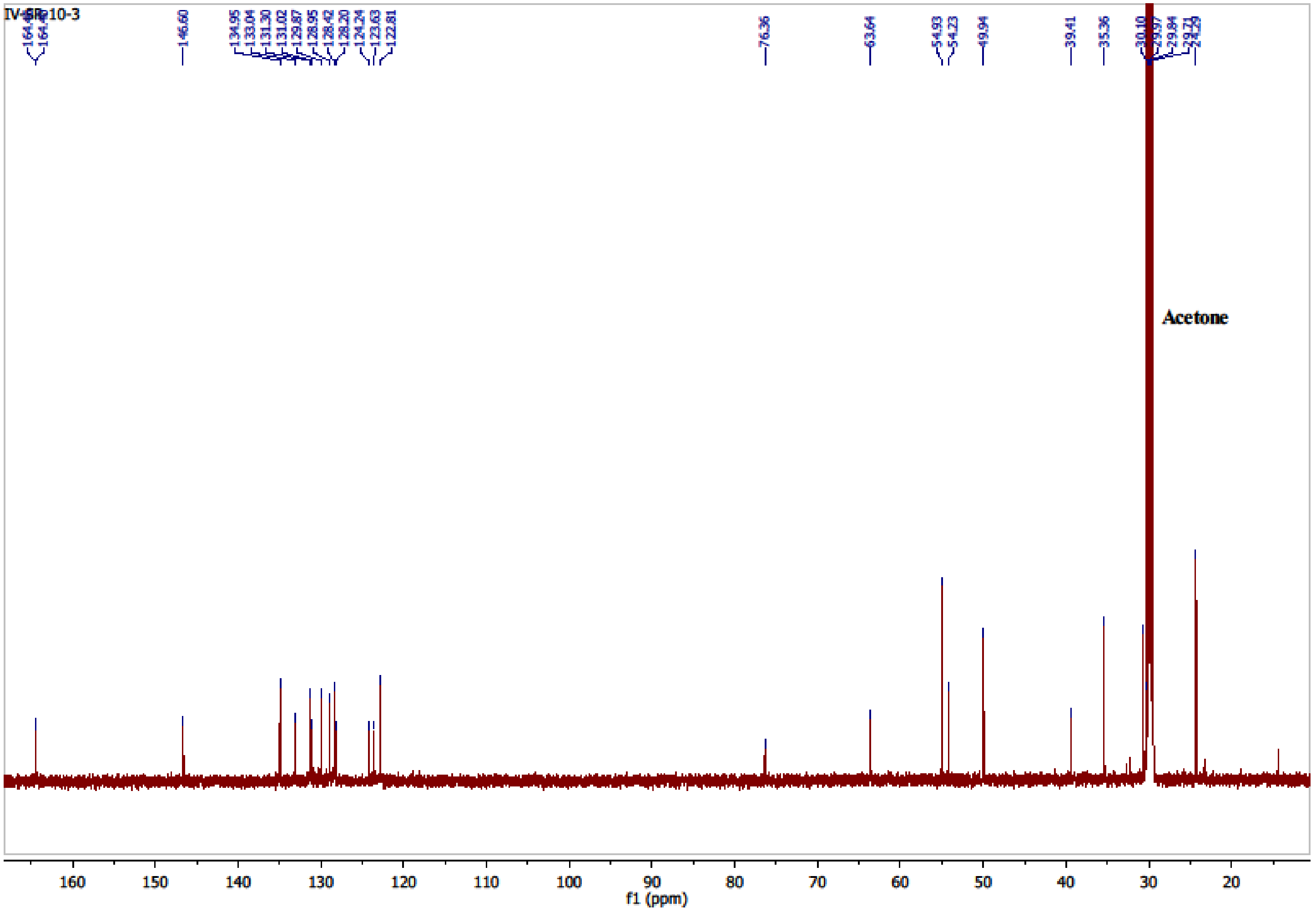


**Figure S24**. $^{13}C$ NMR spectrum of **9**.

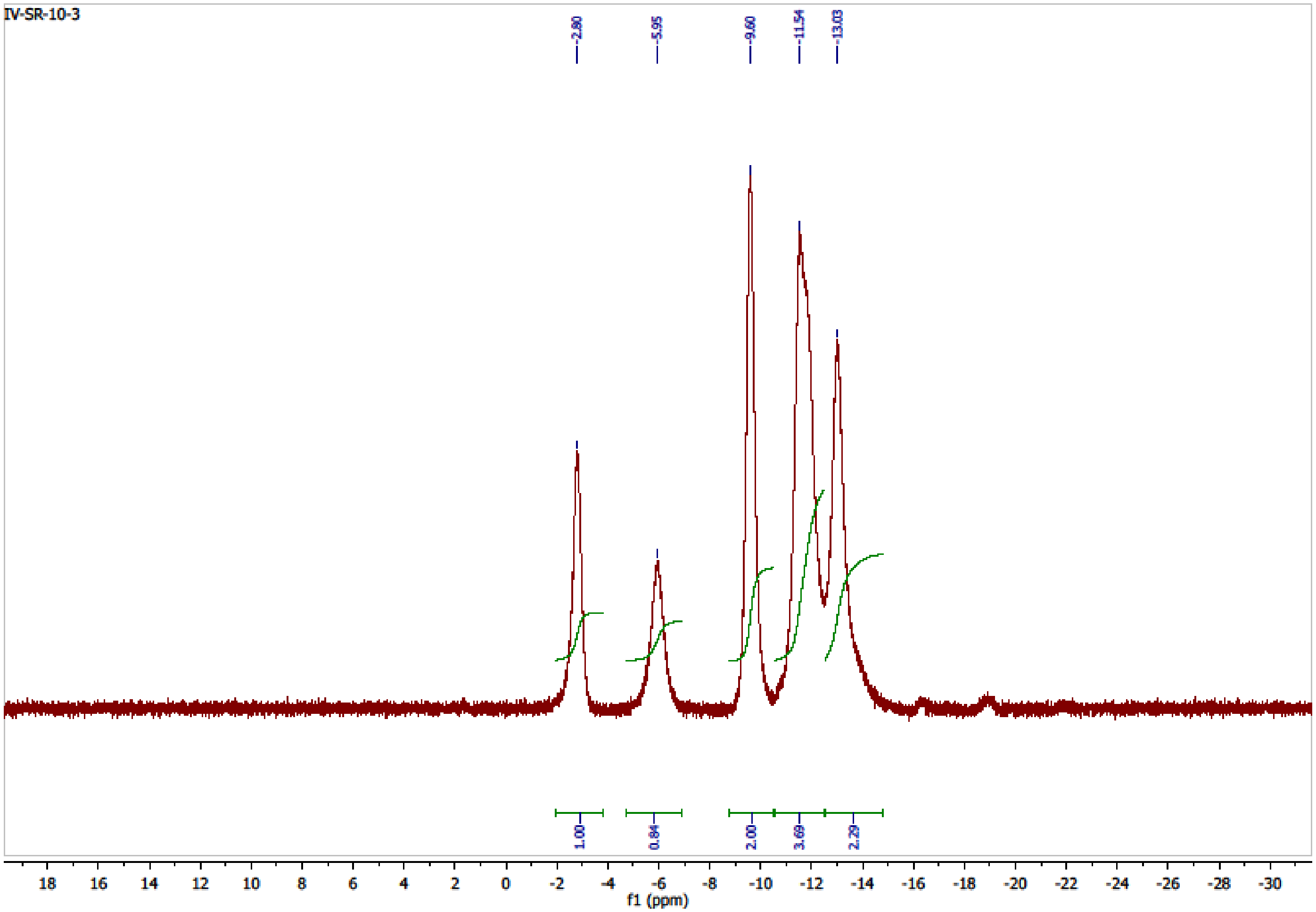


**Figure S25**. $^{11}B$ NMR {$^{1}H$ BB} spectrum of **9**.

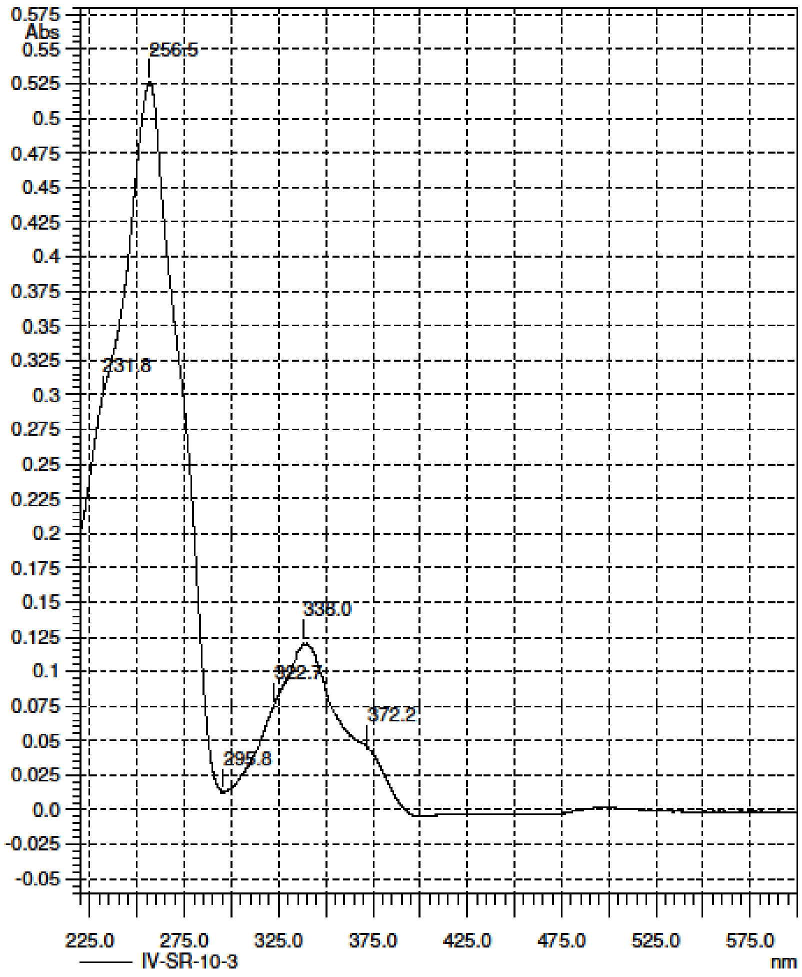


**Figure S26**. UV spectrum of **9**.

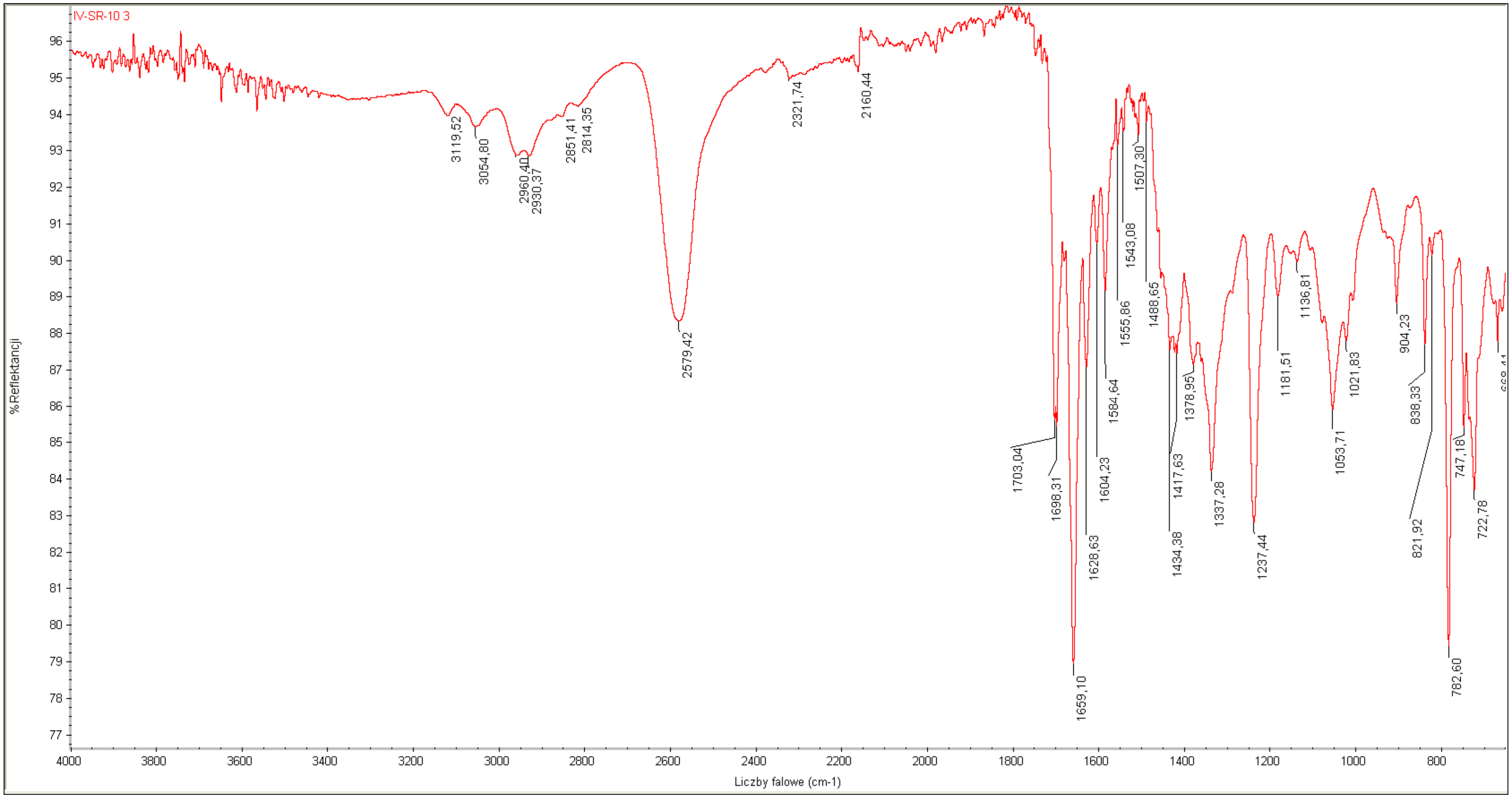


**Figure S27**. IR spectrum of **9**.

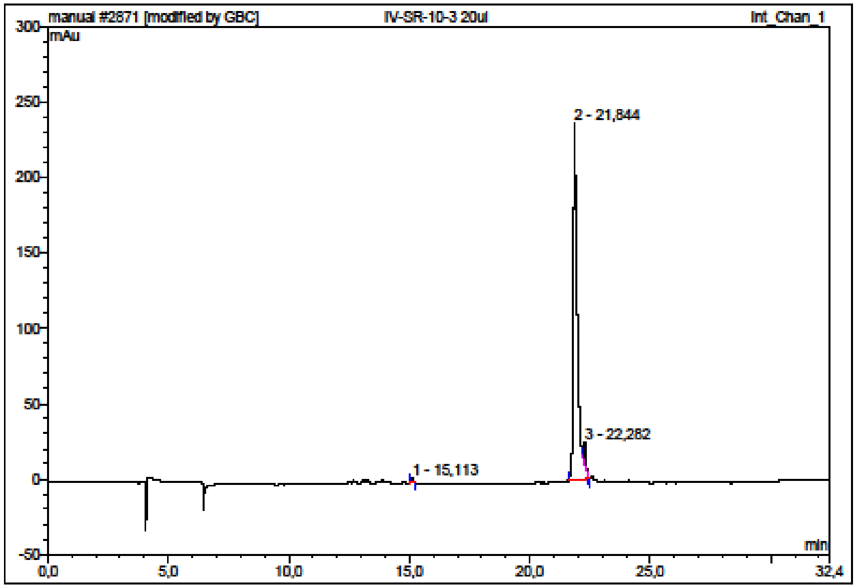


| No. | Ret.Time min | Peak Name | Height mAu | Area mAu*min | Rel.Area % | Amount | Type |
|---|---|---|---|---|---|---|---|
| 1 | 15,11 | n.a. | 3,035 | 0,348 | 0,72 | n.a. | BMB* |
| 2 | 21,84 | n.a. | 236,872 | 46,733 | 96,65 | n.a. | BMB* |
| 3 | 22,28 | n.a. | 15,135 | 1,272 | 2,63 | n.a. | Rd* |
| Total: | | | 255,042 | 48,353 | 100,00 | 0,000 | |

**Figure S28**. HPLC analysis of **9**.

Spectrum Name: IV-SR-10_3_PT
Start Ion: 300
End Ion: 700
Source: APCI + 10.0µA 400C
Capillary: 150V 300C Offset: 25V Span: 0V

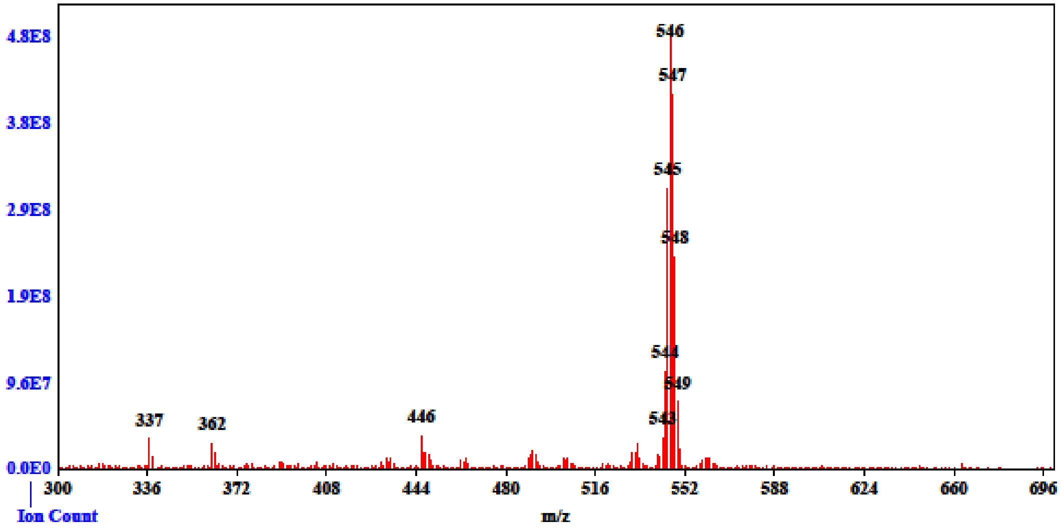


**Figure S29.** MS spectrum of **9**.

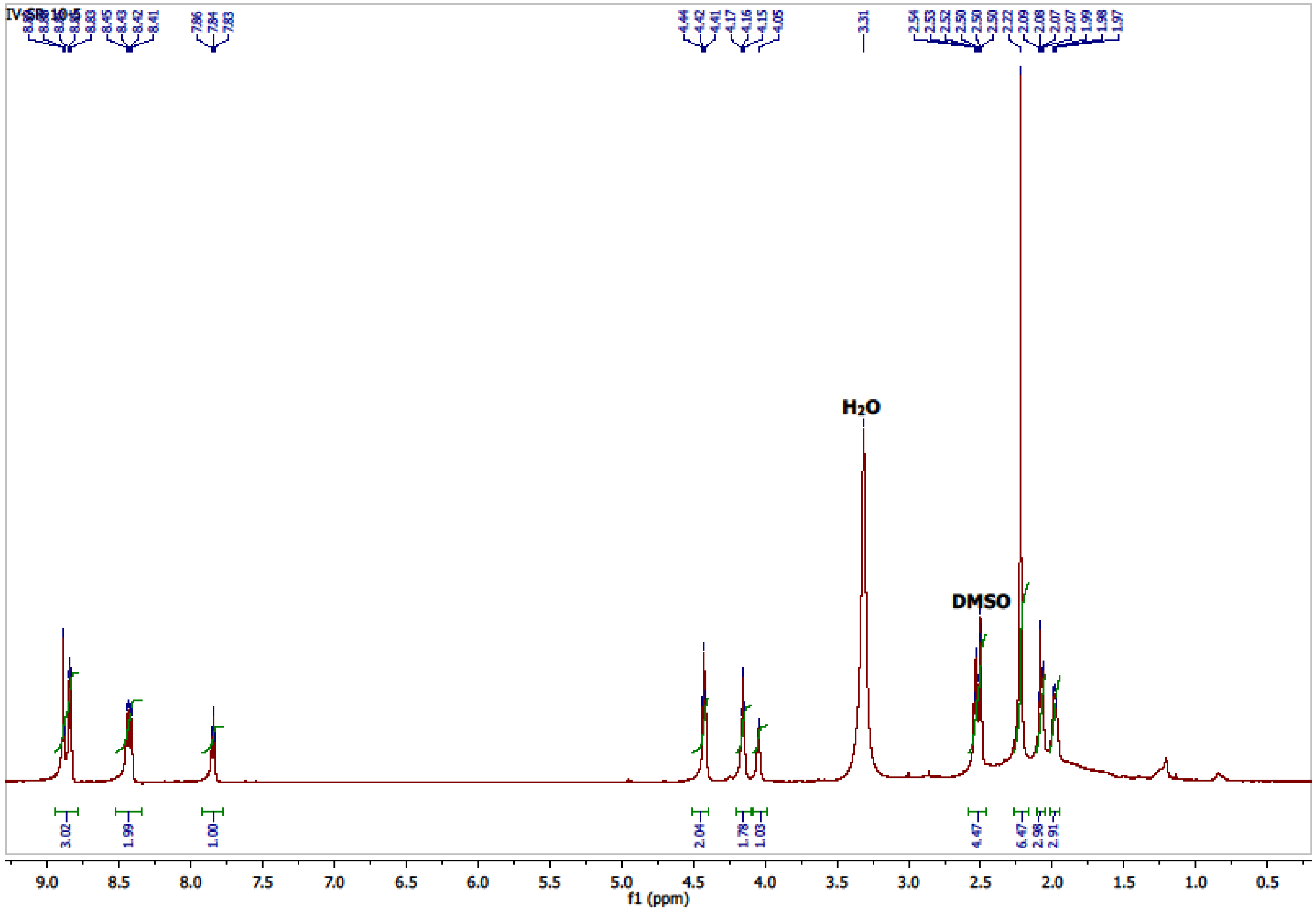


**Figure S30**. $^{1}H$ NMR spectrum of **10**.

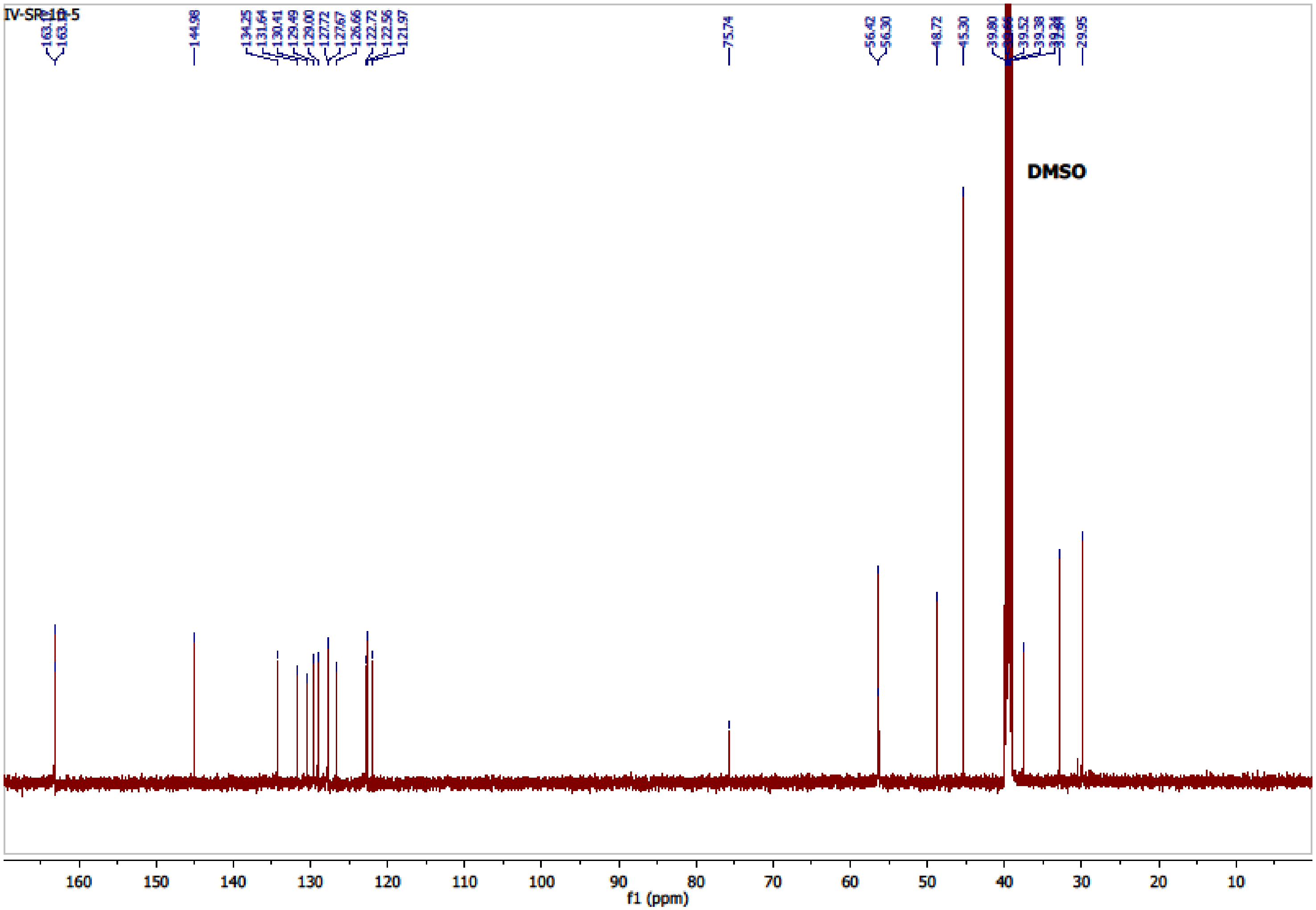

**Figure S31**. $^{13}C$ NMR spectrum of **10**.

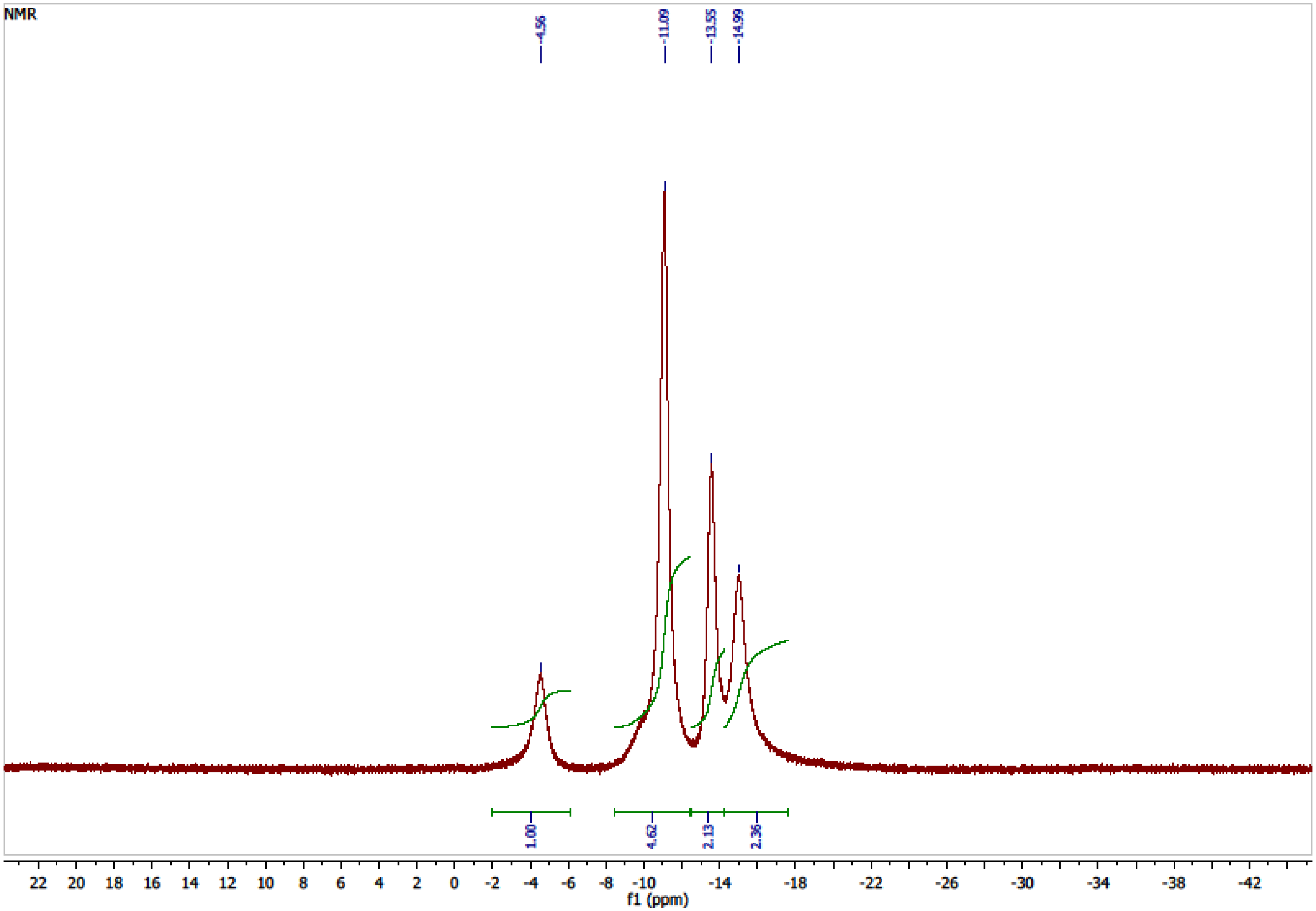


**Figure S32**. $^{11}B$ NMR {$^{1}H$ BB} spectrum of **10**.

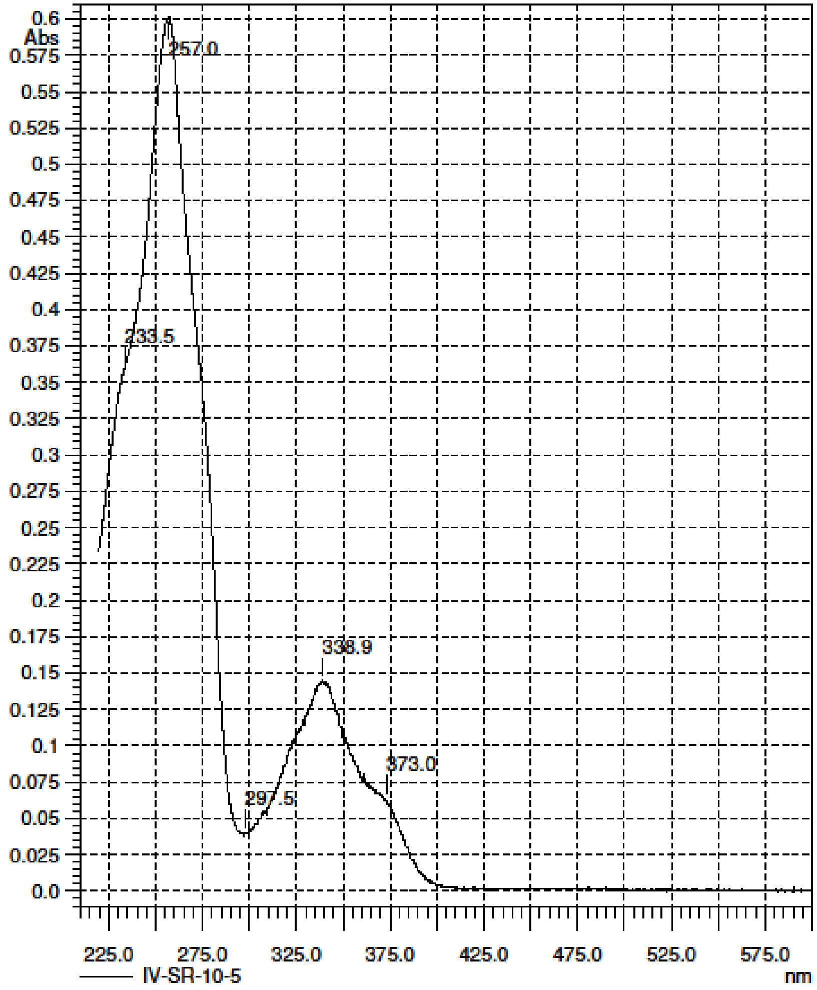


**Figure S33**. UV spectrum of **10**.

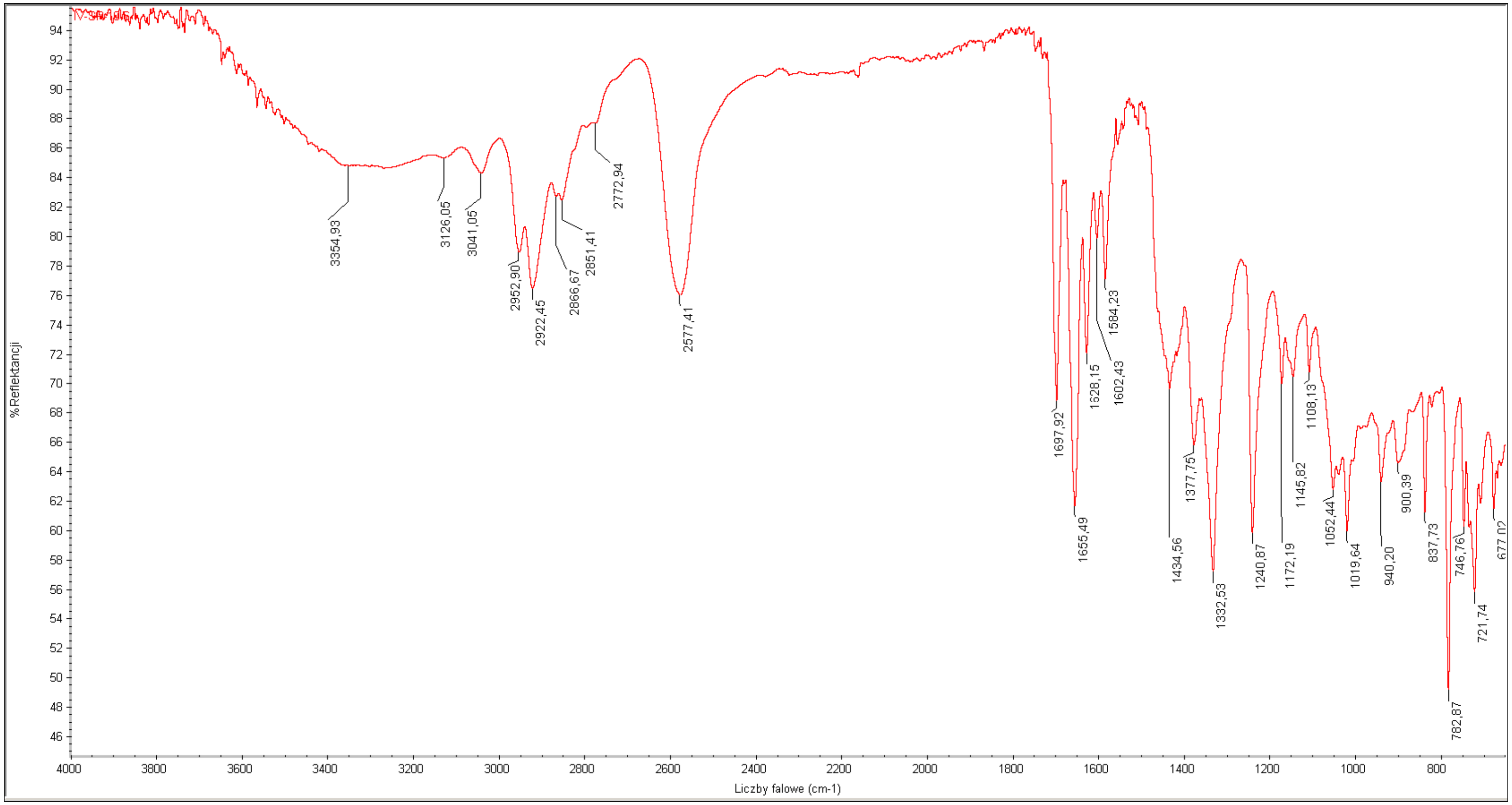


**Figure S34**. IR spectrum of **10**.

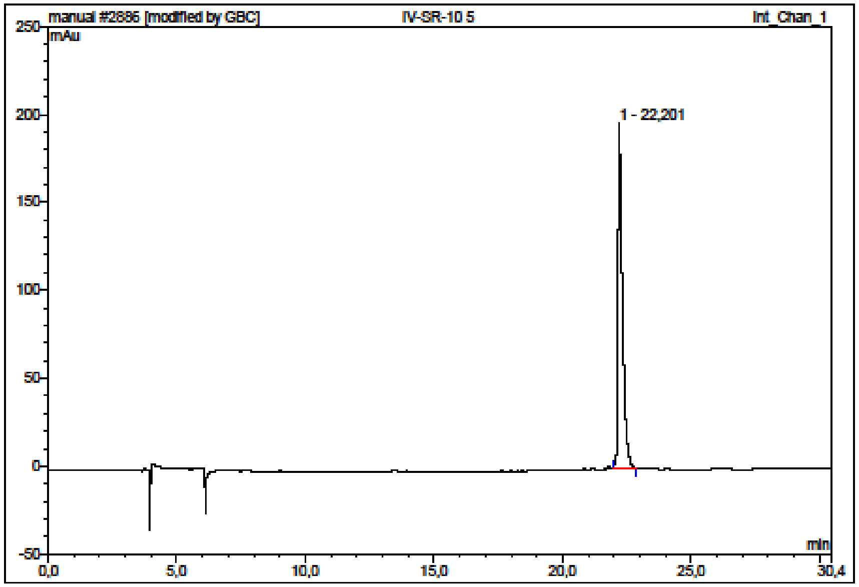


| No. | Ret.Time min | Peak Name | Height mAu | Area mAu*min | Rel.Area % | Amount | Type |
|---|---|---|---|---|---|---|---|
| 1 | 22,20 | n.a. | 196,209 | 39,594 | 100,00 | n.a. | BMB |
| Total: | | | 196,209 | 39,594 | 100,00 | 0,000 | |

**Figure S35**. HPLC analysis of **10**.

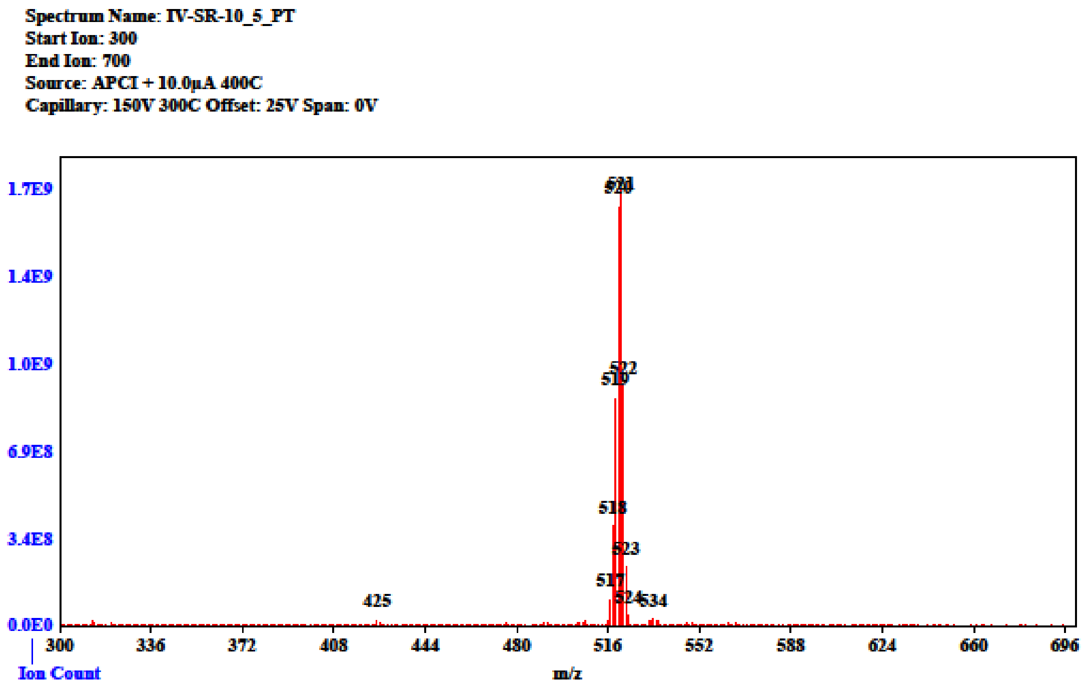


**Figure S36**. MS spectrum of **10**.

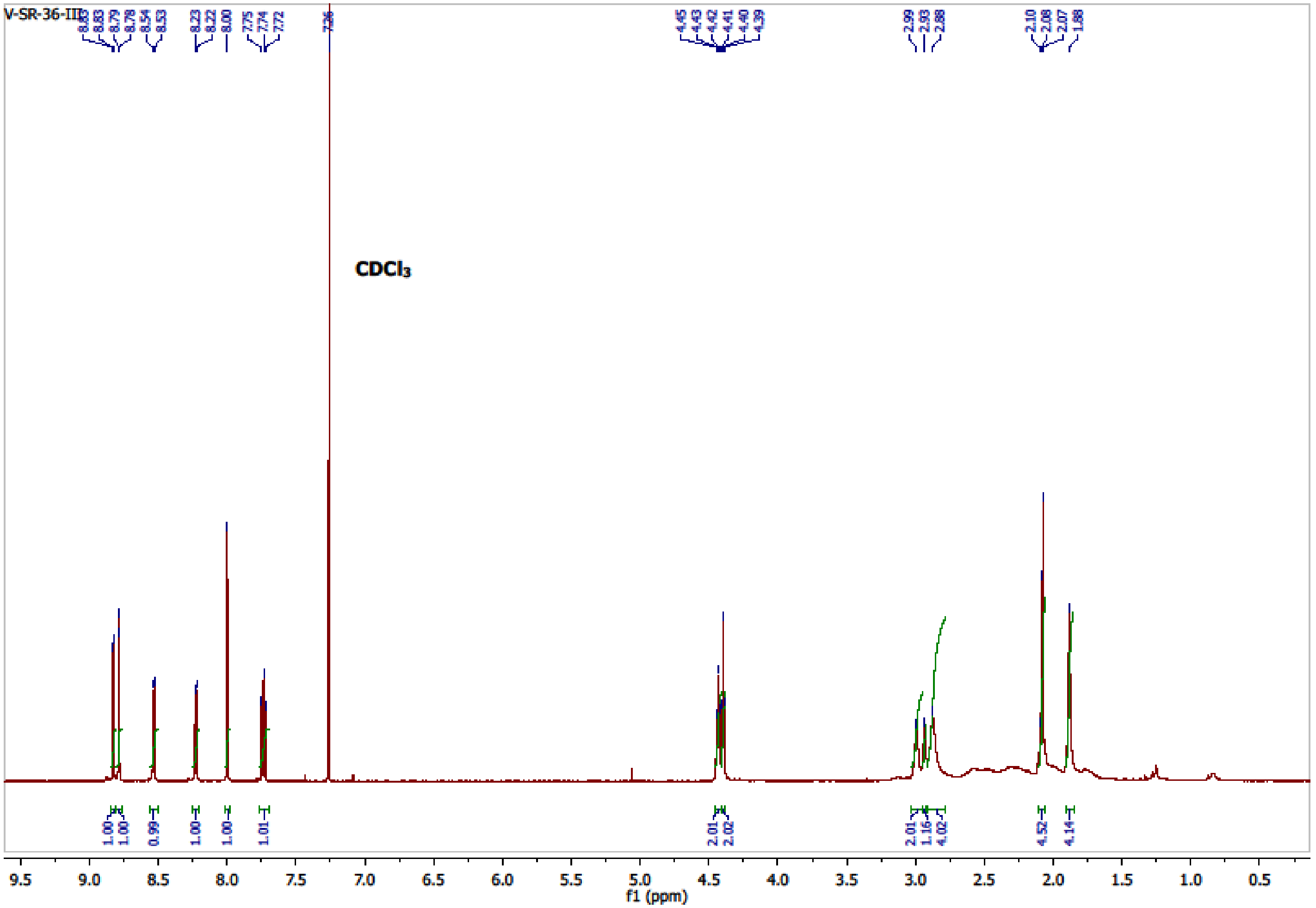


**Figure S37**. $^{1}$H NMR spectrum of **11**.

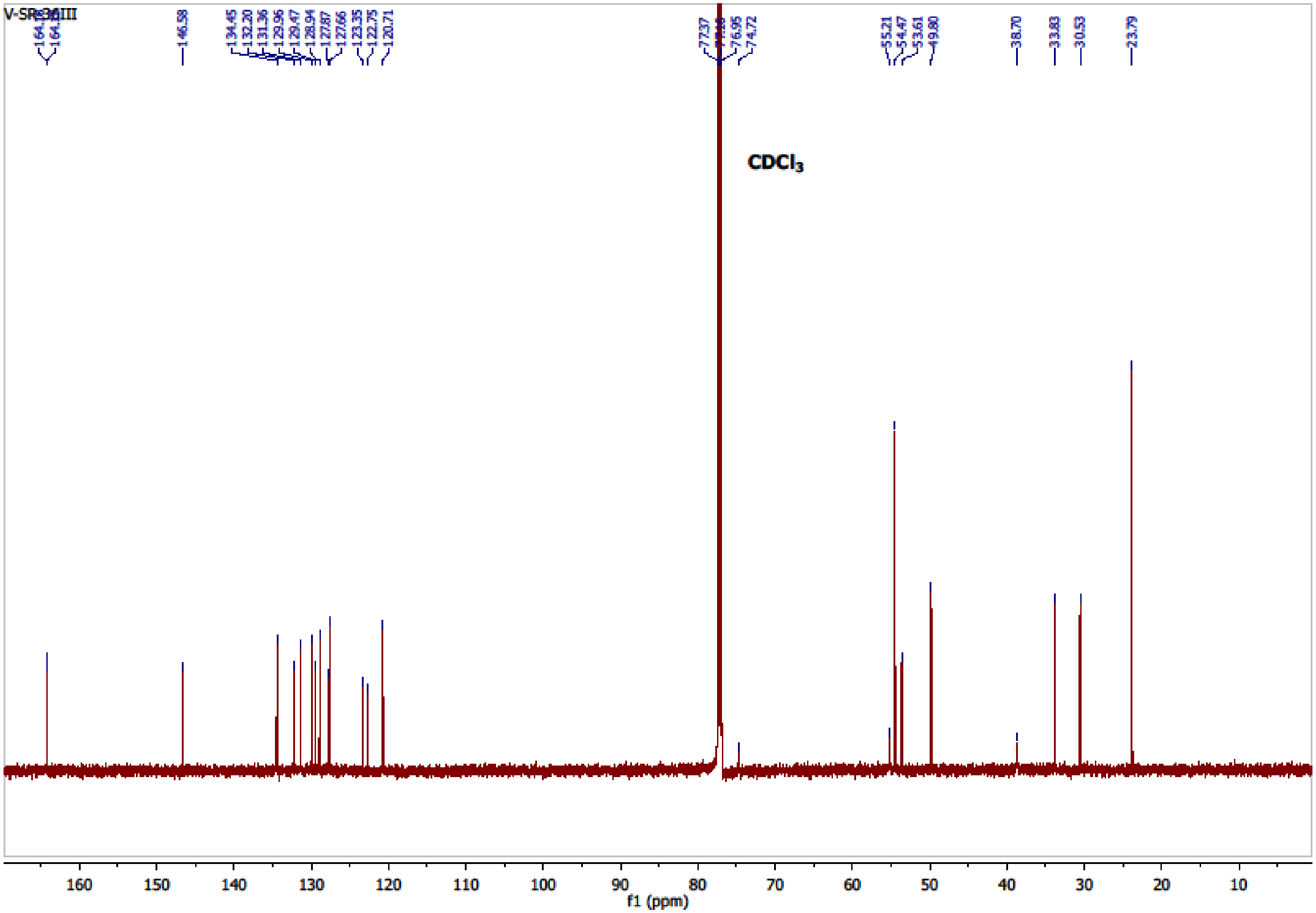


**Figure S38**. $^{13}C$ NMR spectrum of **11**.

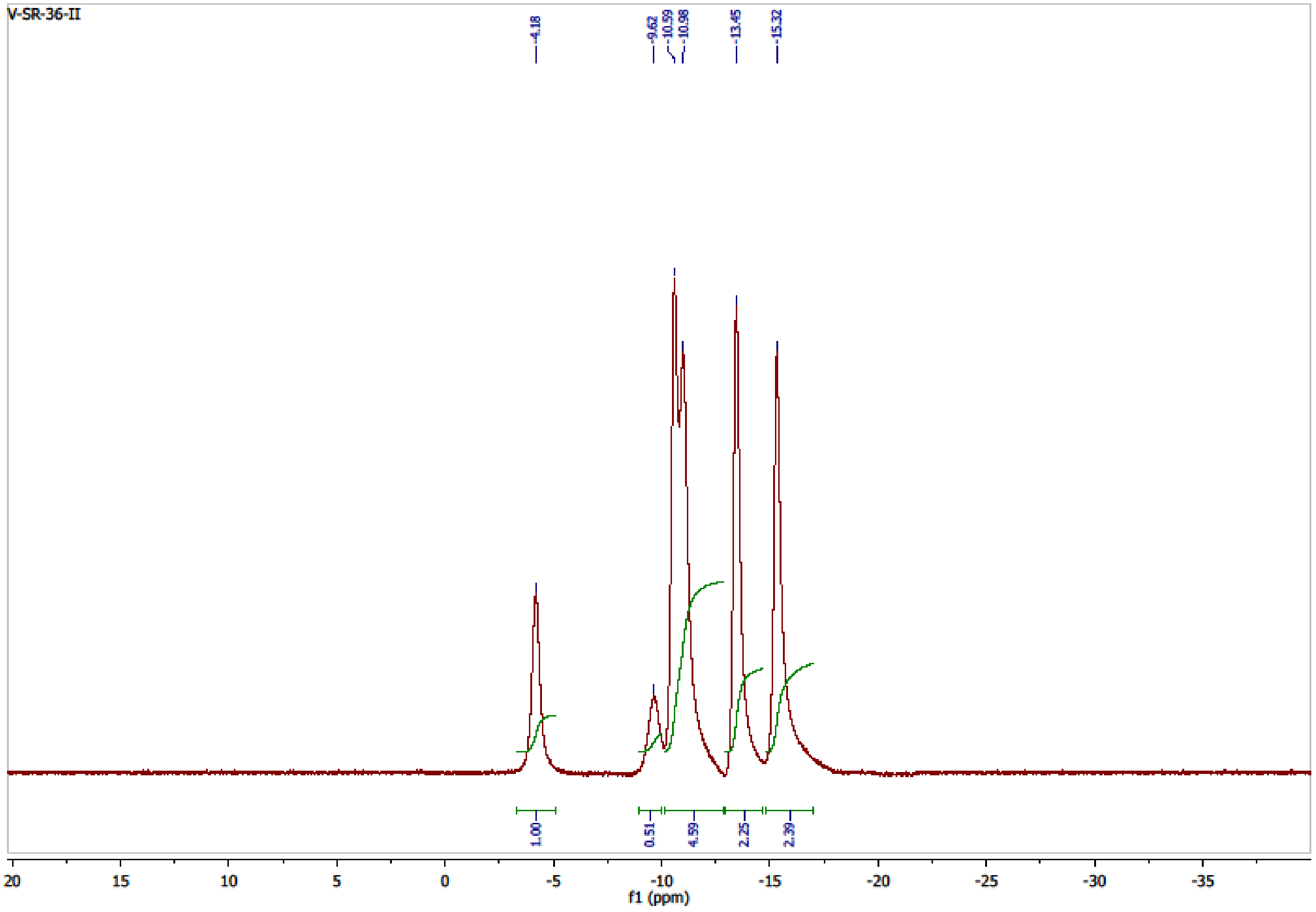


**Figure S39**. $^{11}B$ NMR {$^{1}H$ BB} spectrum of **11**.

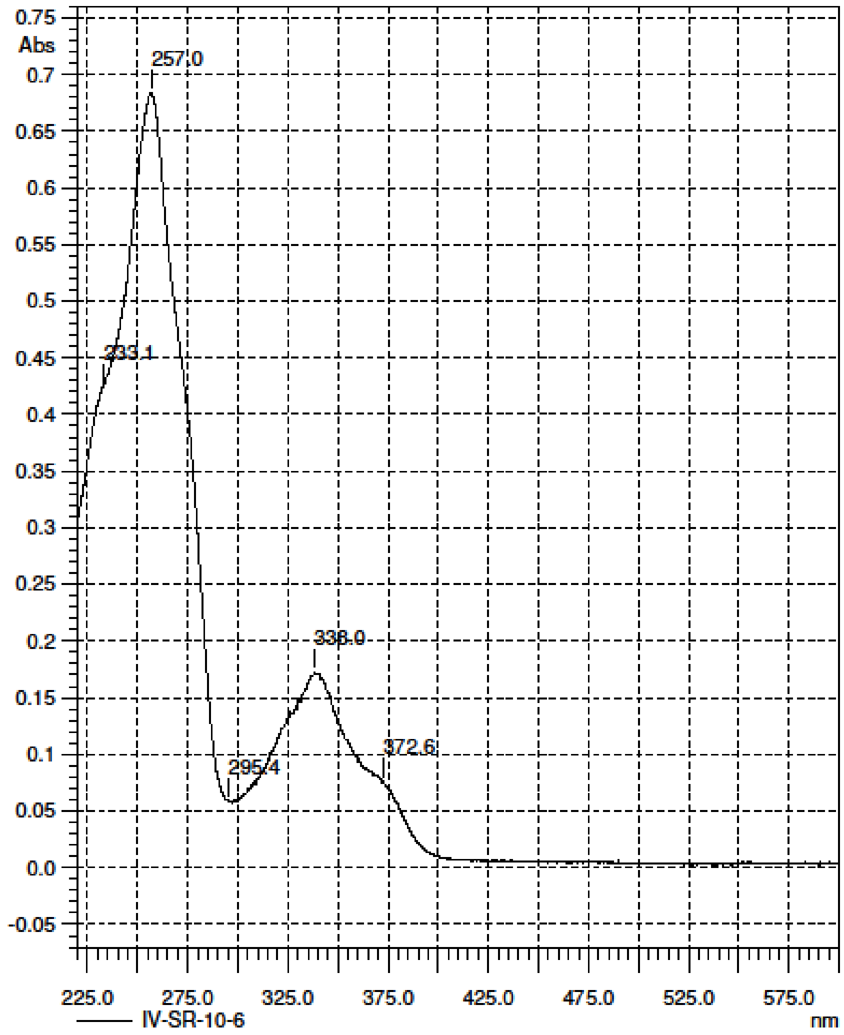


**Figure S40**. UV spectrum of **11**.

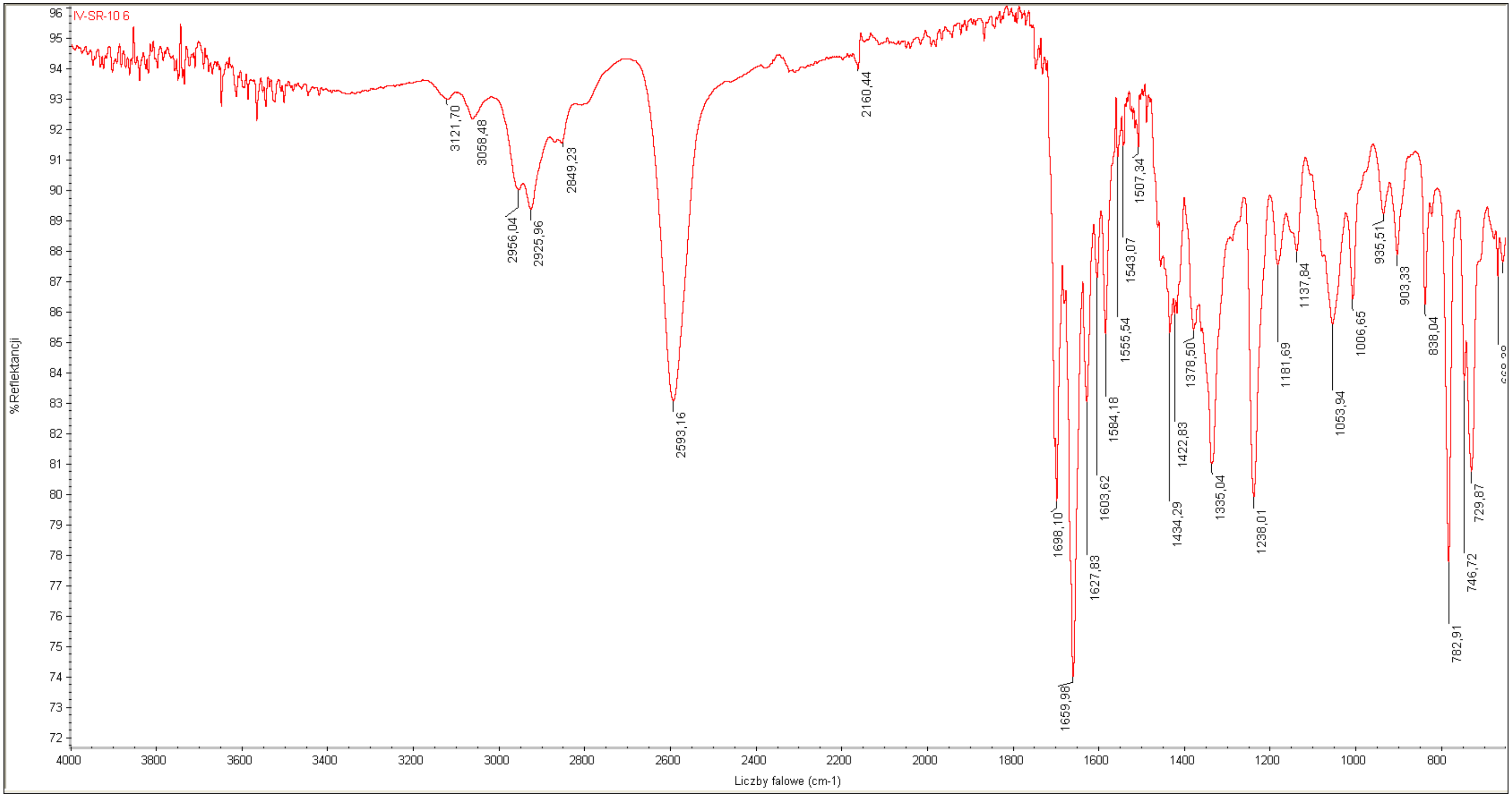


**Figure S41**. IR spectrum of **11**.

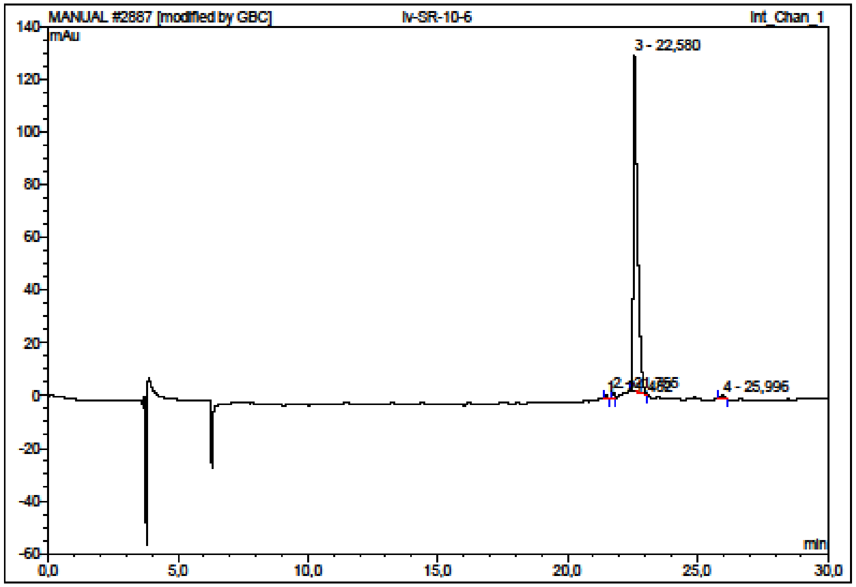


| No. | Ret.Time min | Peak Name | Height mAu | Area mAu*min | Rel.Area % | Amount | Type |
|---|---|---|---|---|---|---|---|
| 1 | 21,48 | n.a. | 0,983 | 0,114 | 0,46 | n.a. | BMB* |
| 2 | 21,76 | n.a. | 2,363 | 0,226 | 0,91 | n.a. | BMB* |
| 3 | 22,58 | n.a. | 127,847 | 24,210 | 97,63 | n.a. | BMB* |
| 4 | 26,00 | n.a. | 1,303 | 0,248 | 1,00 | n.a. | BMB* |
| Total: | | | 132,495 | 24,798 | 100,00 | 0,000 | |

**Figure S42**. HPLC analysis of **11**.

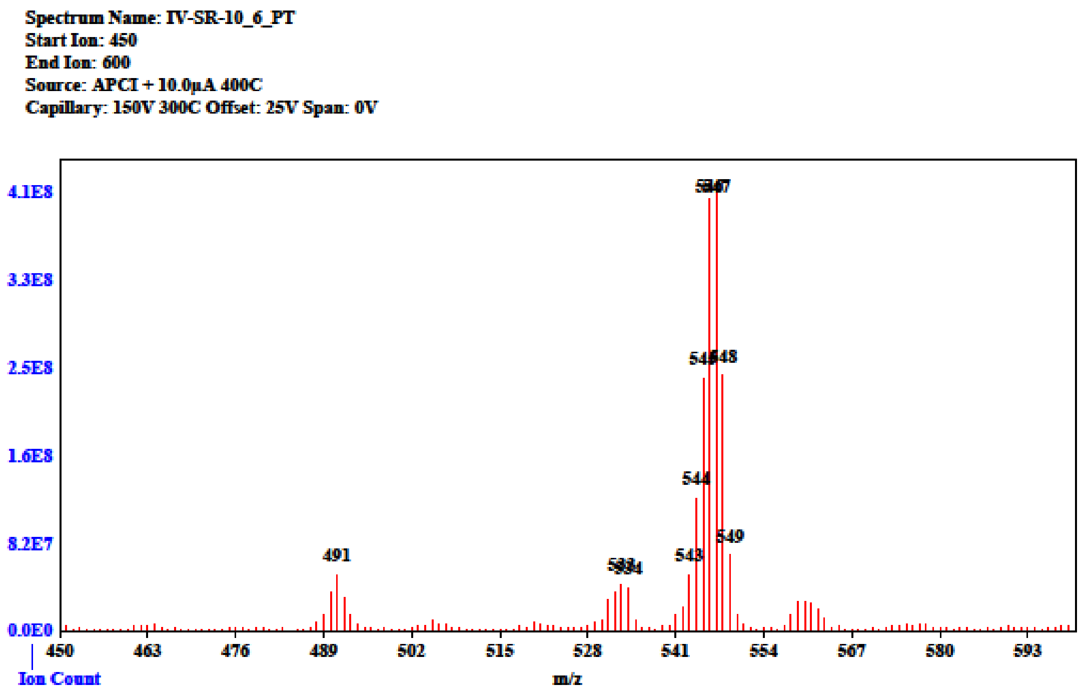


**Figure S43**. MS spectrum of **11**.

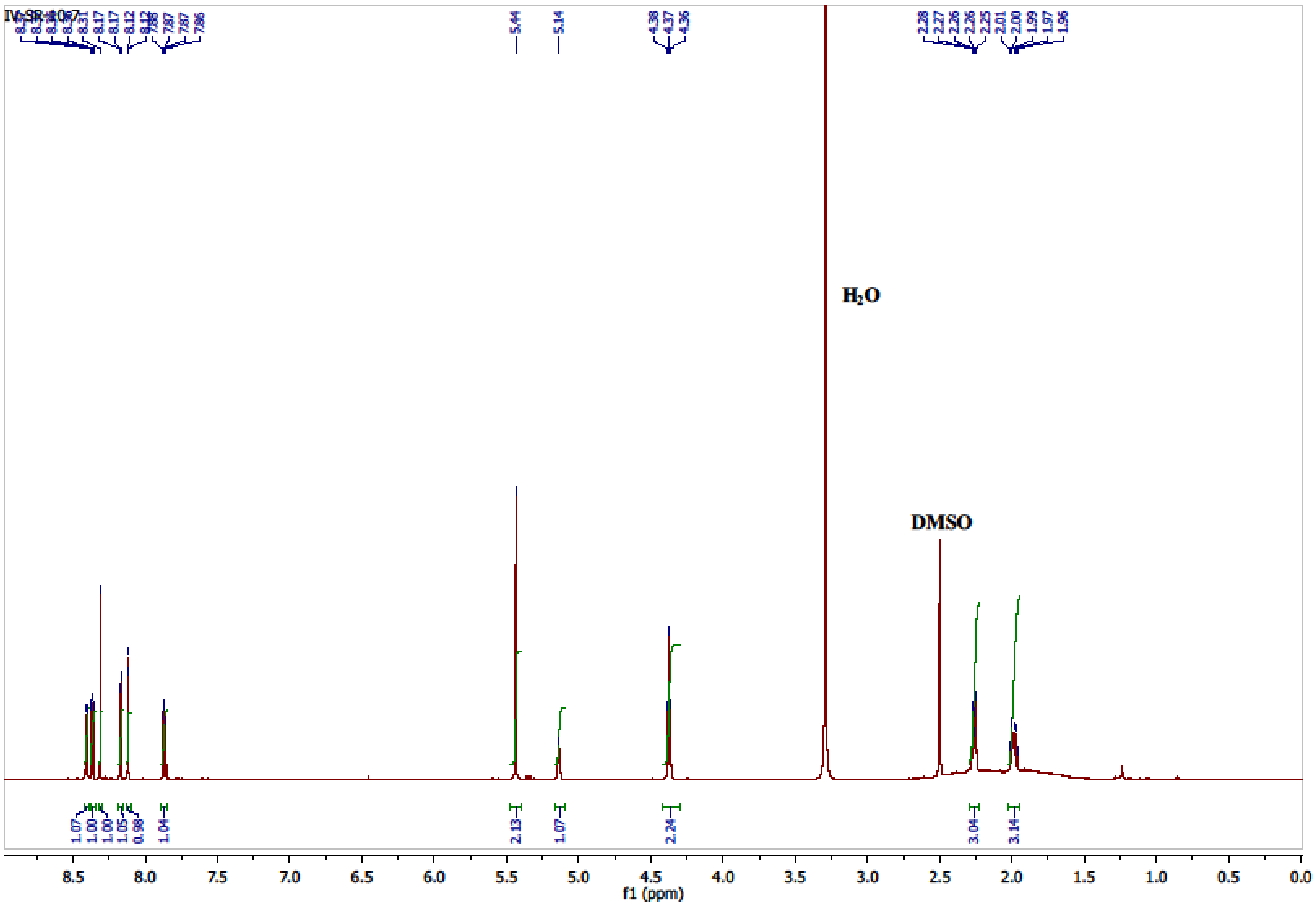


**Figure S44**. ¹H NMR spectrum of **15**.

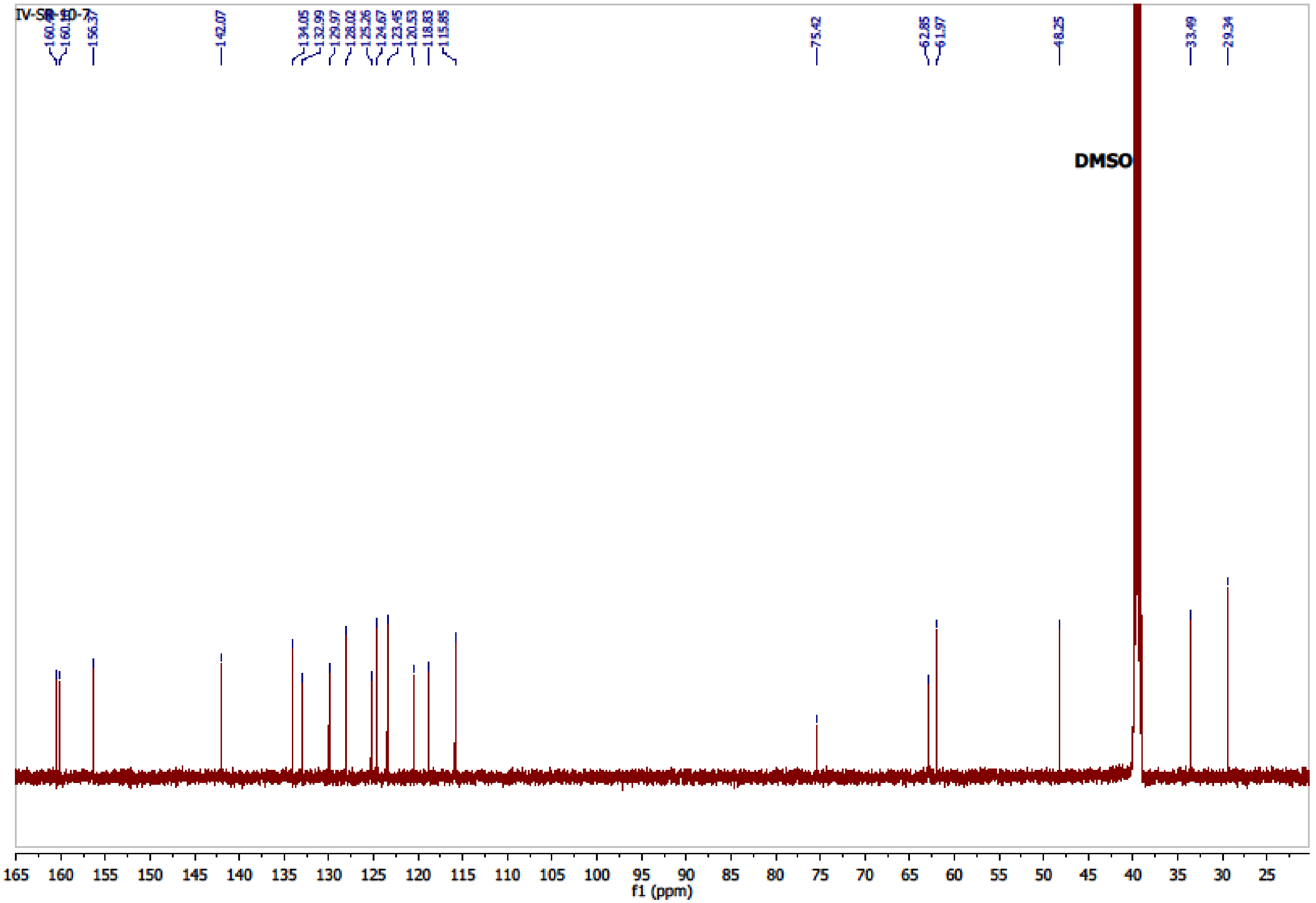


**Figure S45**. $^{13}C$ NMR spectrum of **15**.

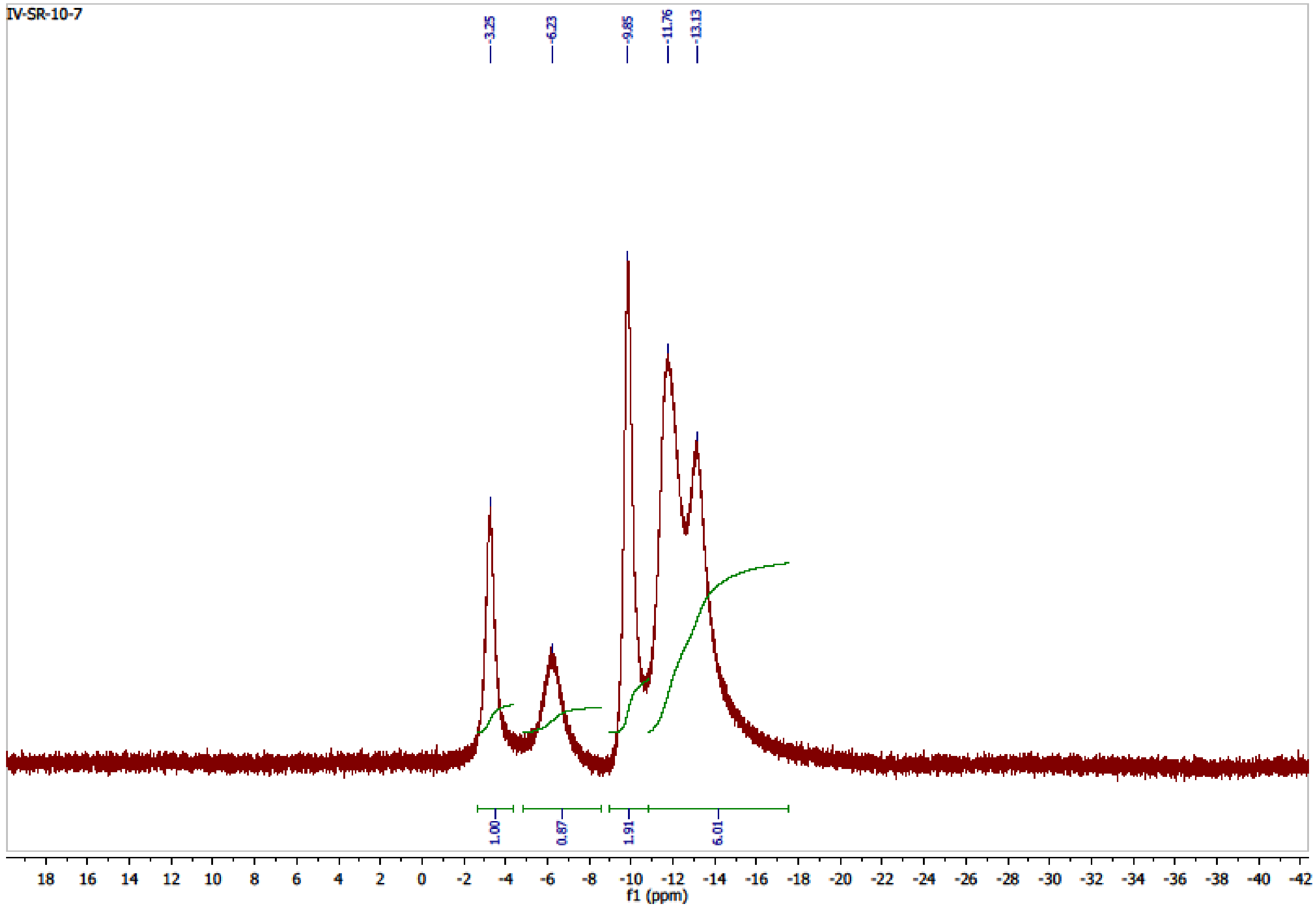


**Figure S46**. $^{11}$B NMR {$^{1}$H BB} spectrum of **15**.

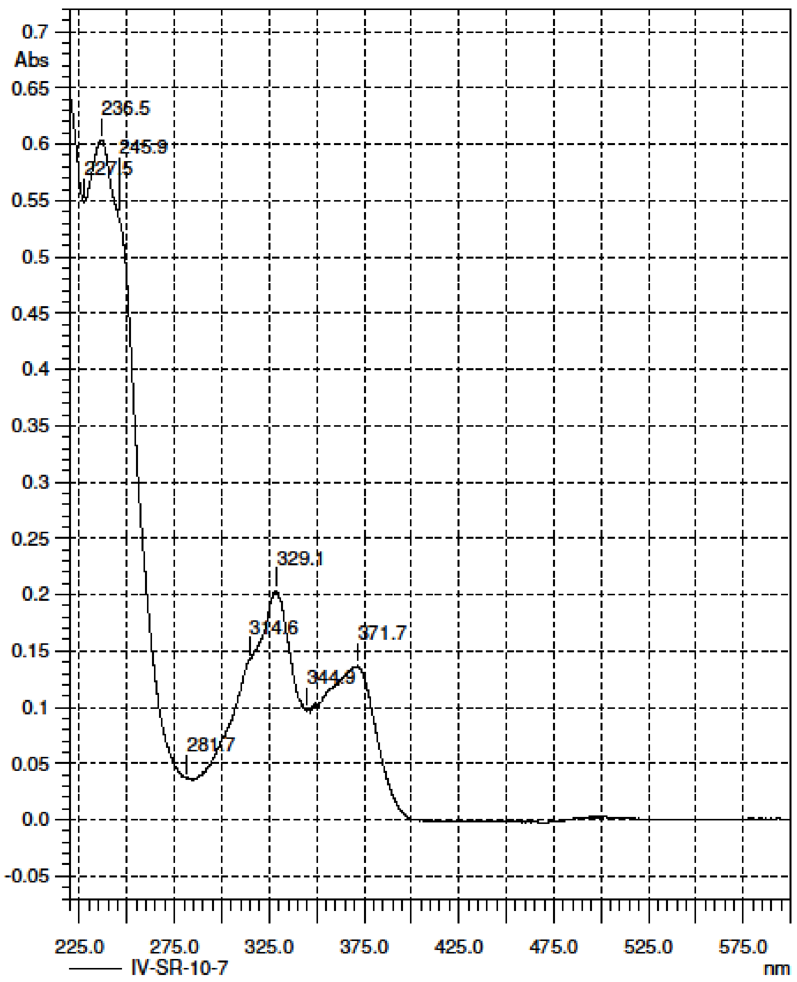


**Figure S47**. UV spectrum of **15**.

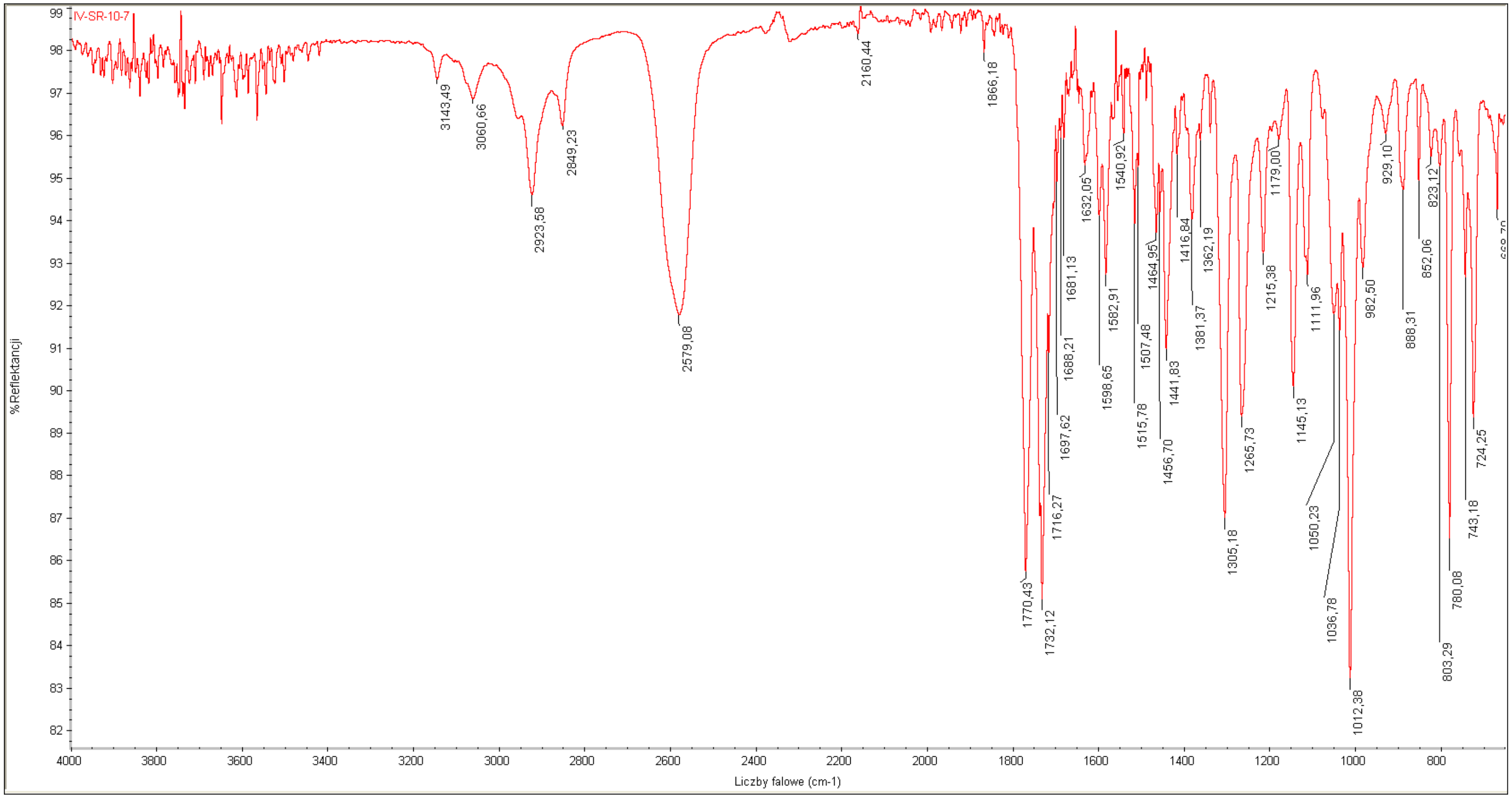


**Figure S48**. IR spectrum of **15**.

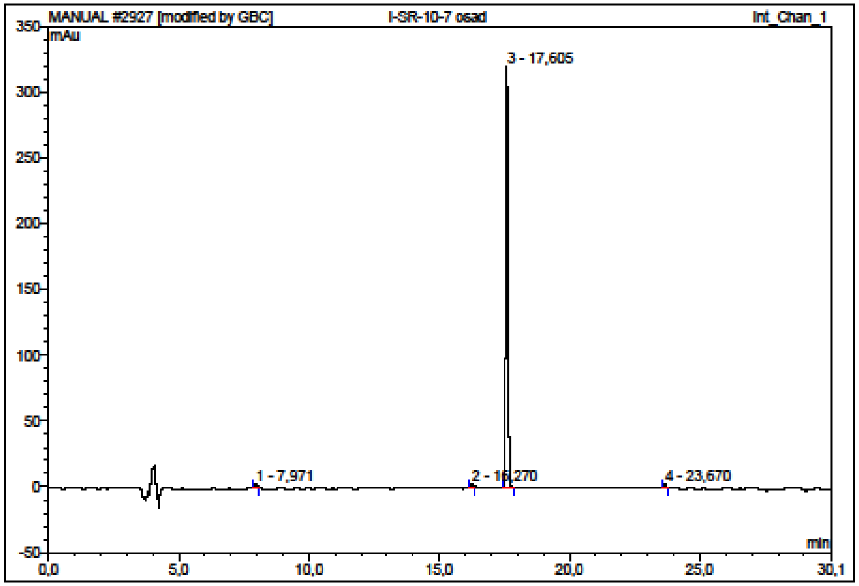


| No. | Ret.Time min | Peak Name | Height mAu | Area mAu*min | Rel.Area % | Amount | Type |
|---|---|---|---|---|---|---|---|
| 1 | 7,97 | n.a. | 2,487 | 0,249 | 0,75 | n.a. | BMB* |
| 2 | 16,27 | n.a. | 2,121 | 0,185 | 0,56 | n.a. | BMB* |
| 3 | 17,61 | n.a. | 320,685 | 32,546 | 98,09 | n.a. | BMB |
| 4 | 23,67 | n.a. | 2,109 | 0,199 | 0,60 | n.a. | BMB* |
| Total: | | | 327,402 | 33,179 | 100,00 | 0,000 | |

**Figure S49**. HPLC analysis of **15**.

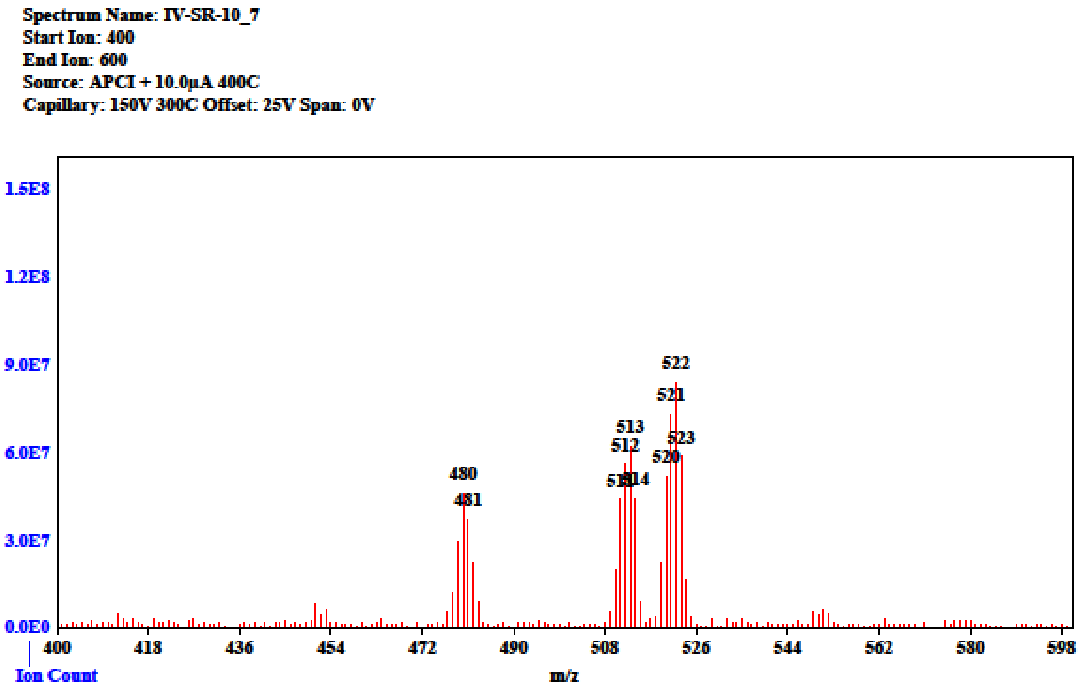


**Figure S50**. MS spectrum of **15**.

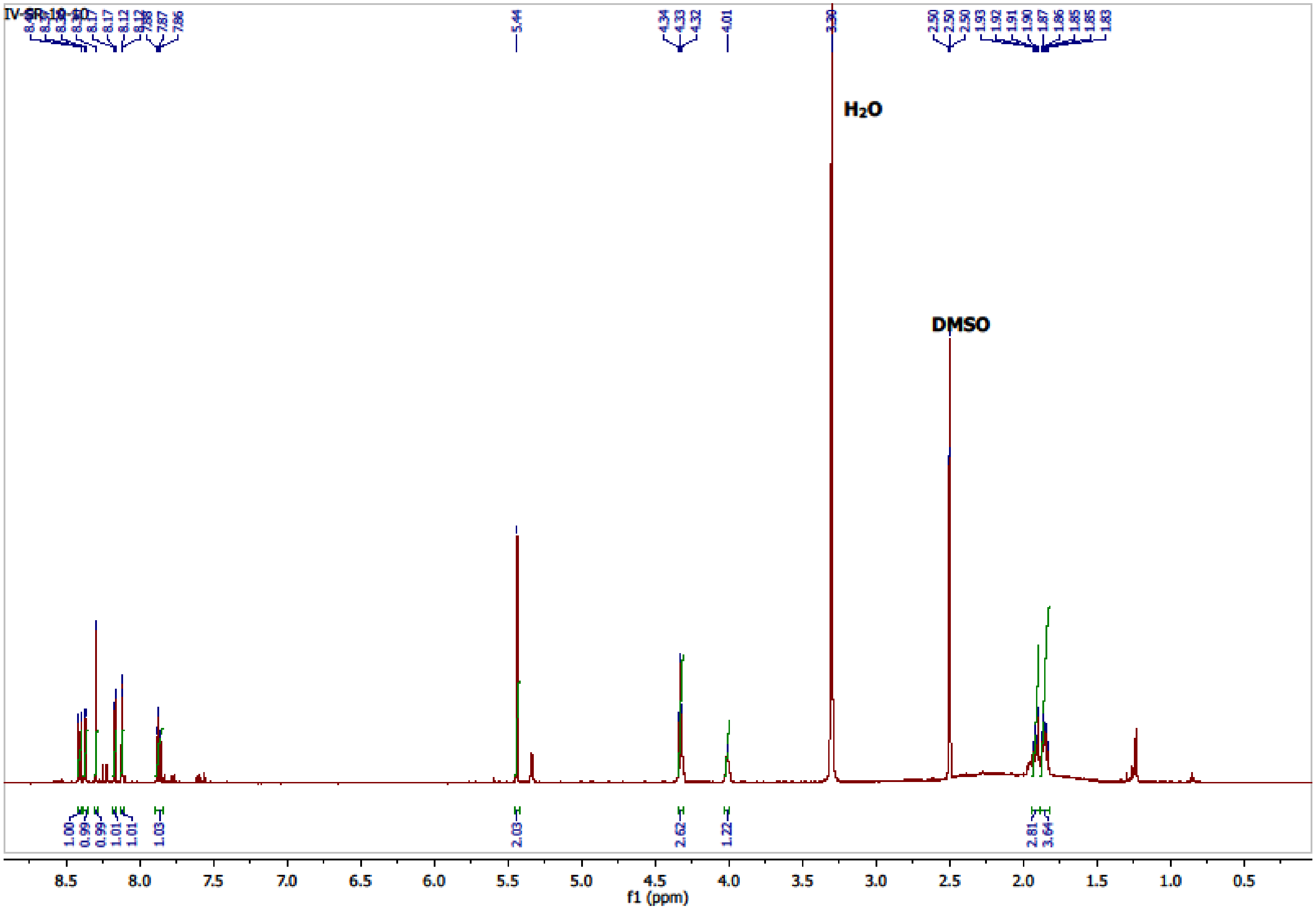

**Figure S51**. $^{1}H$ NMR spectrum of **16**.

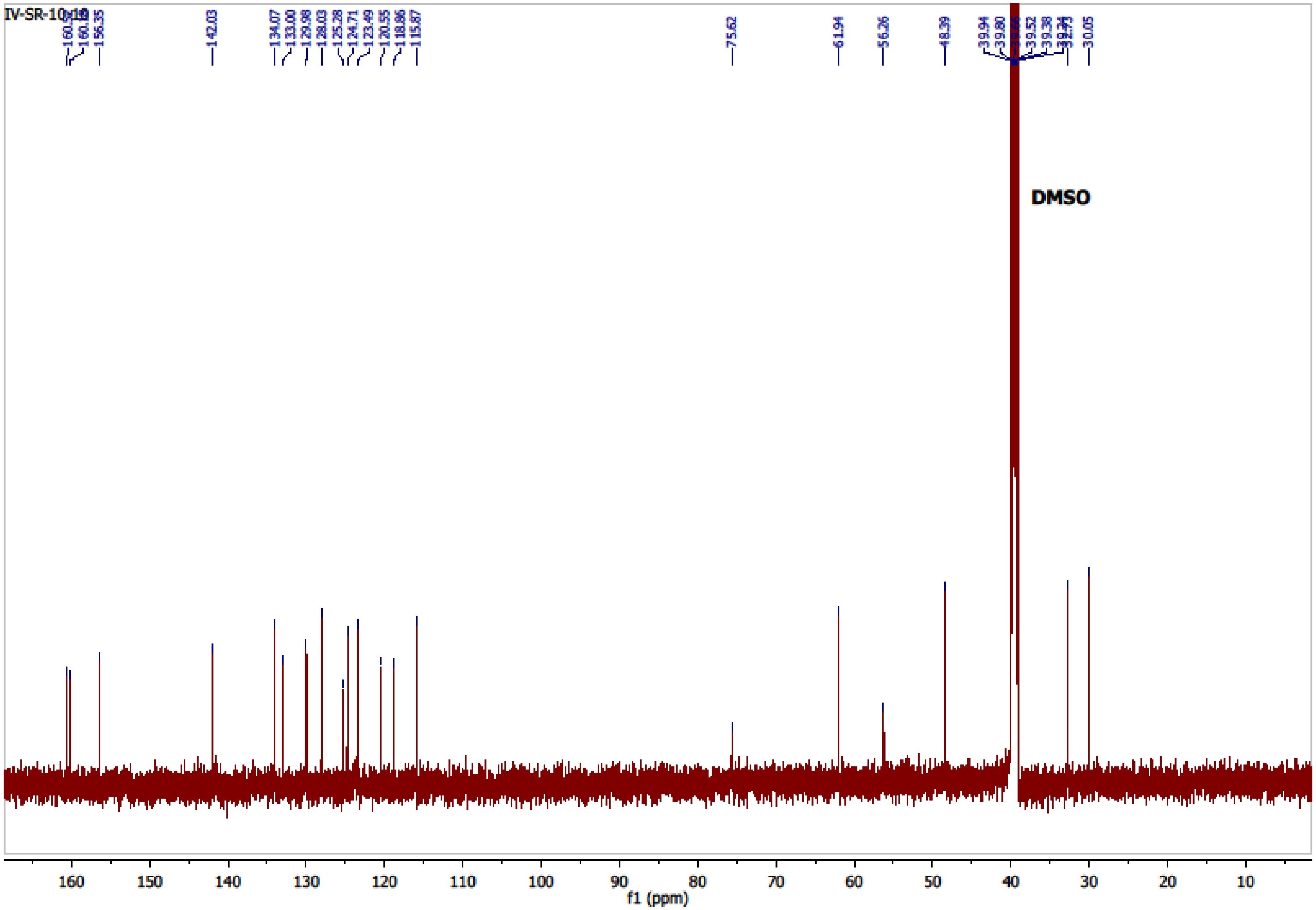


**Figure S52**. $^{13}C$ NMR spectrum of **16**.

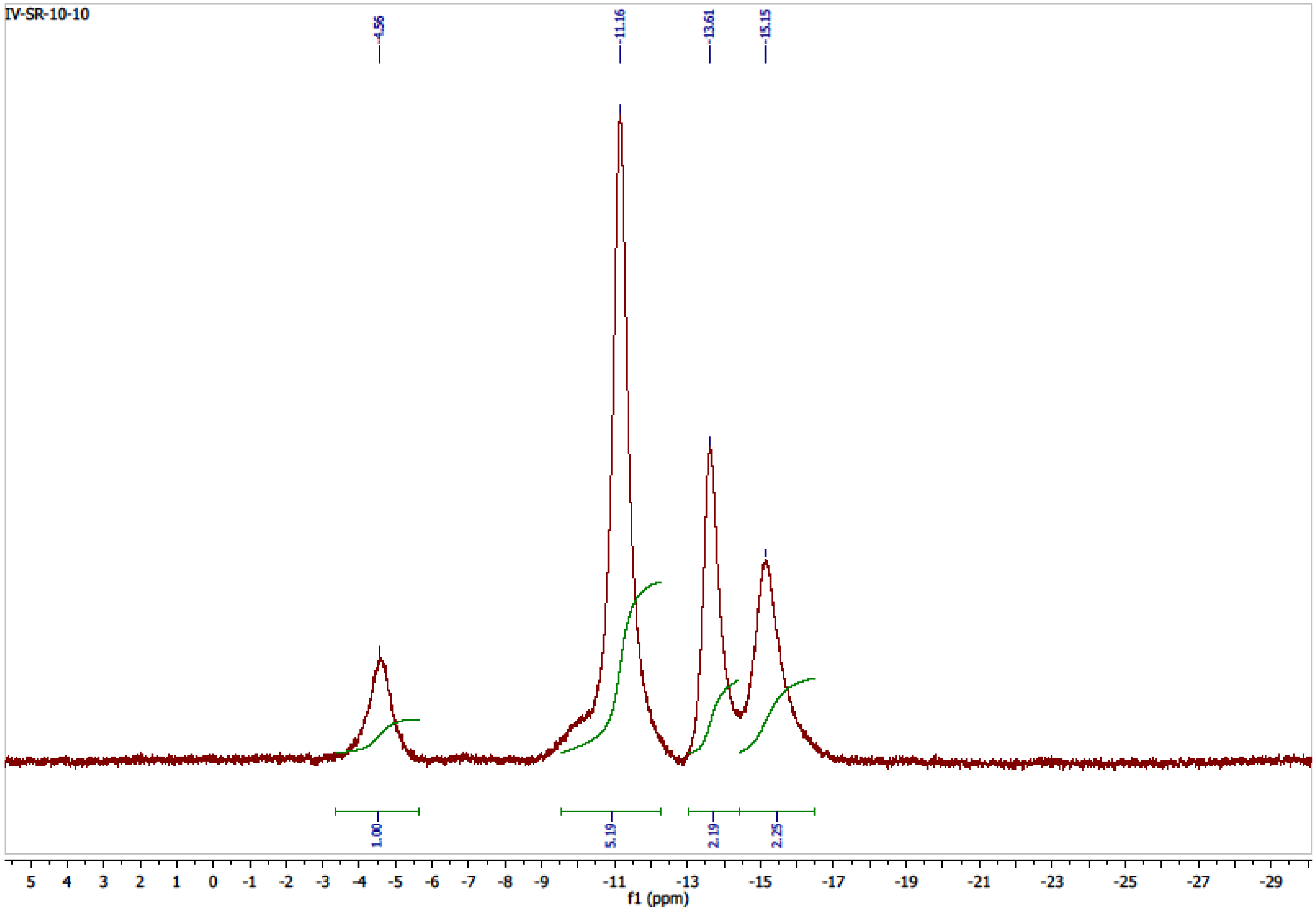


**Figure S53**. $^{11}$B NMR {$^{1}$H BB} spectrum of **16**.

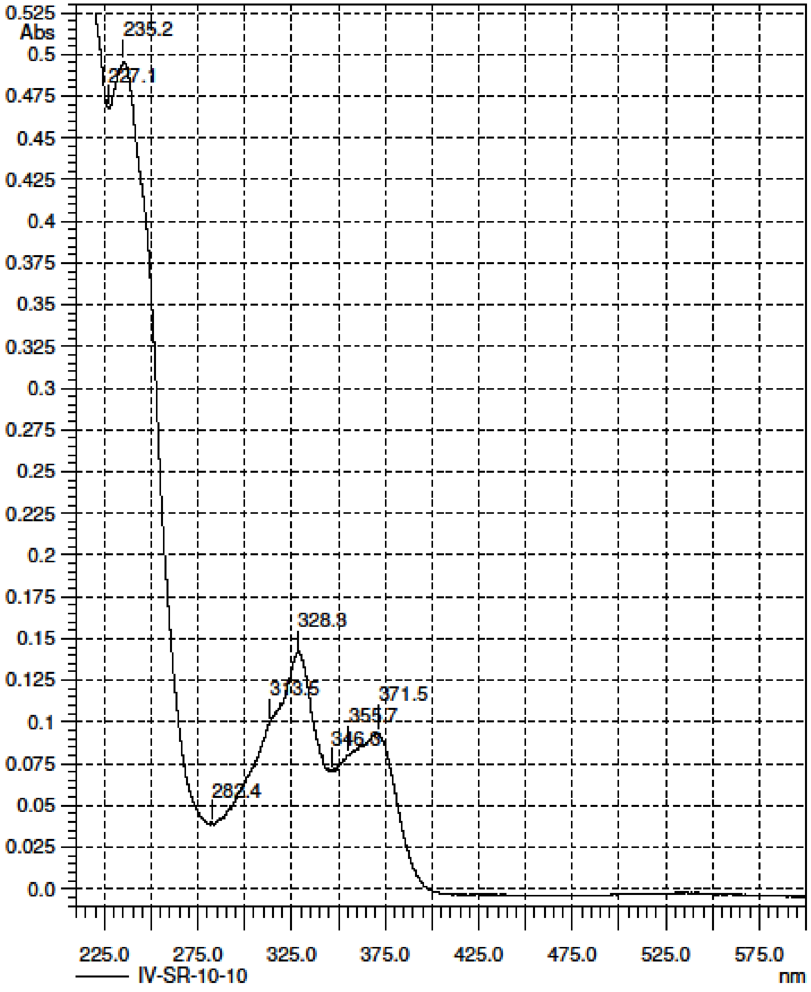


**Figure S54**. UV spectrum of **16**.

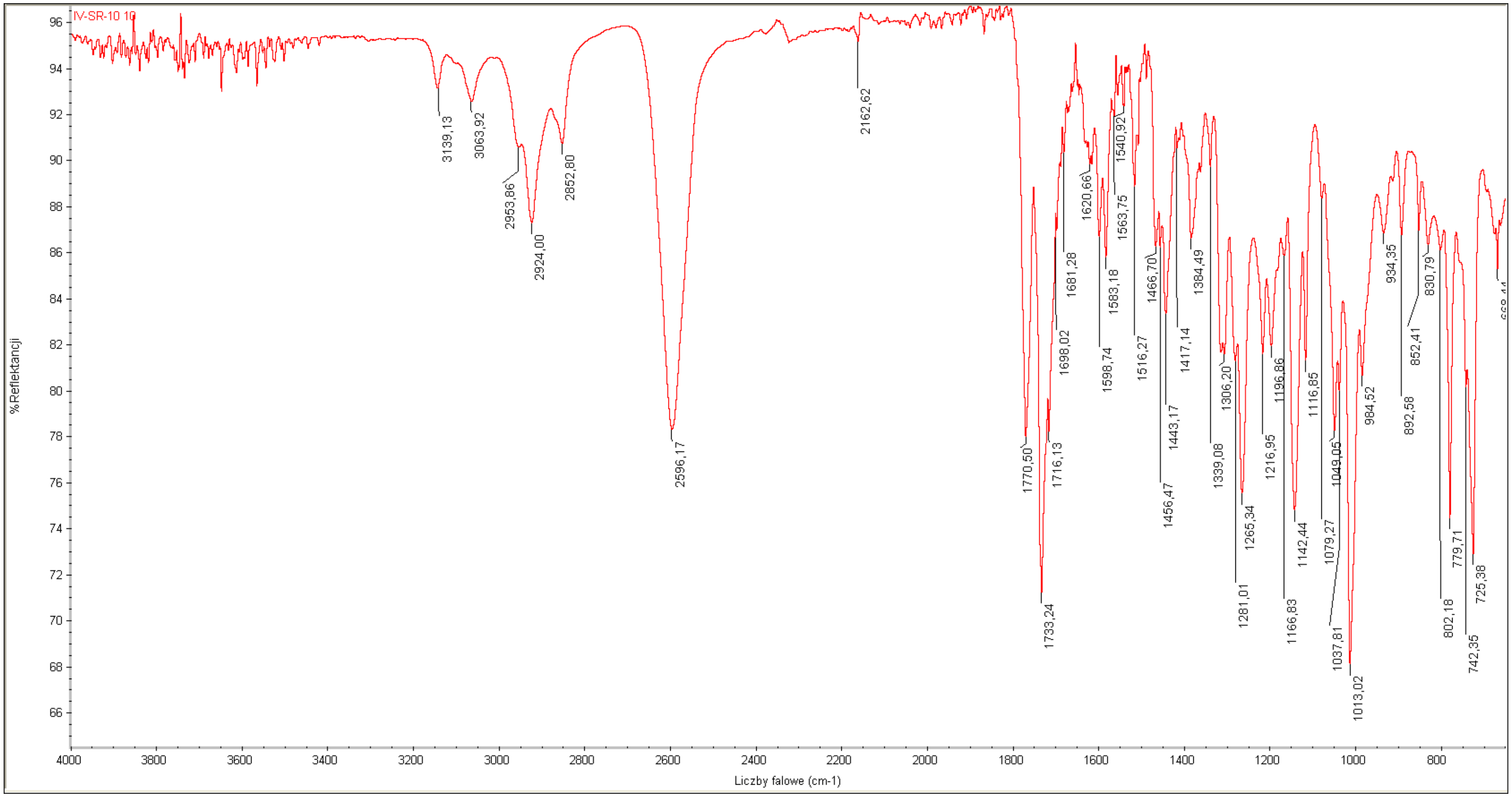

**Figure S55**. IR spectrum of **16**.

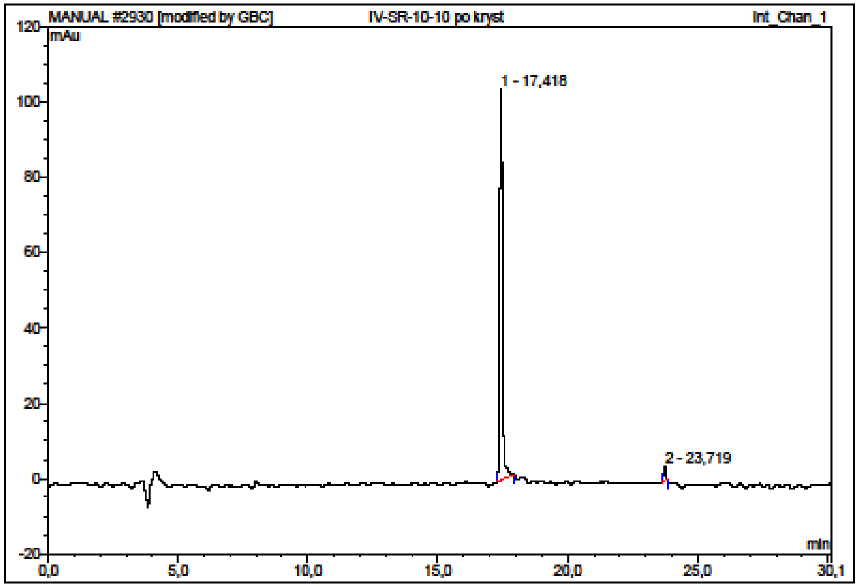


| No. | Ret.Time min | Peak Name | Height mAu | Area mAu*min | Rel.Area % | Amount | Type |
|---|---|---|---|---|---|---|---|
| 1 | 17,42 | n.a. | 103,998 | 11,152 | 96,15 | n.a. | BMB |
| 2 | 23,72 | n.a. | 4,200 | 0,446 | 3,85 | n.a. | BMB* |
| Total: | | | 108,198 | 11,598 | 100,00 | 0,000 | |

**Figure S56**. HPLC analysis of **16**.

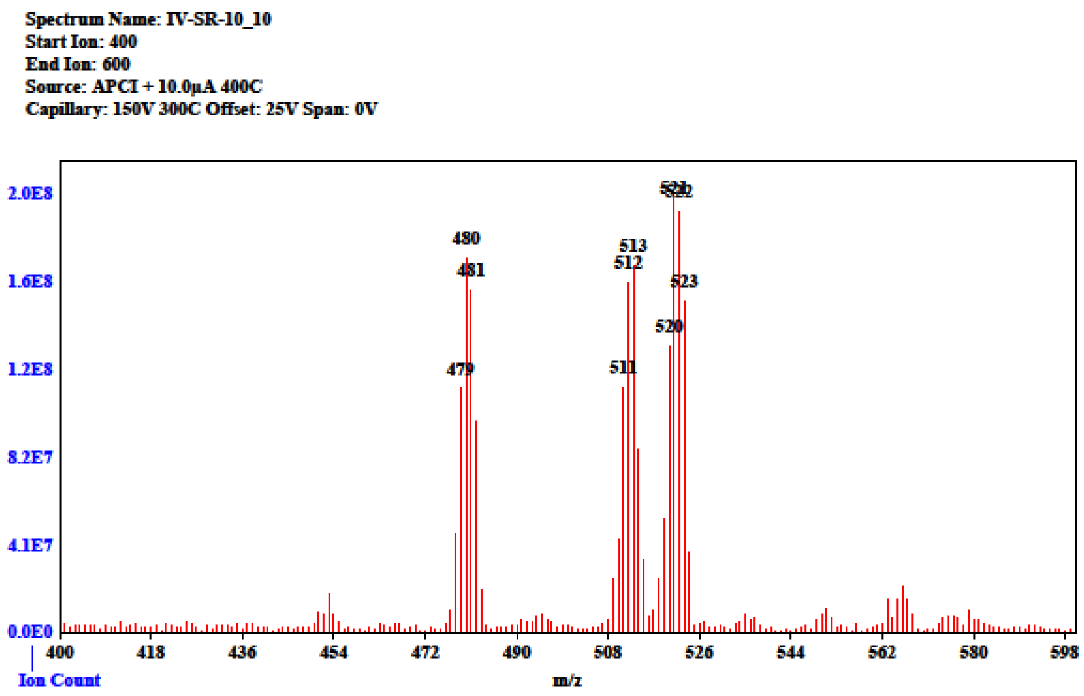


**Figure S57**. MS spectrum of **16**.

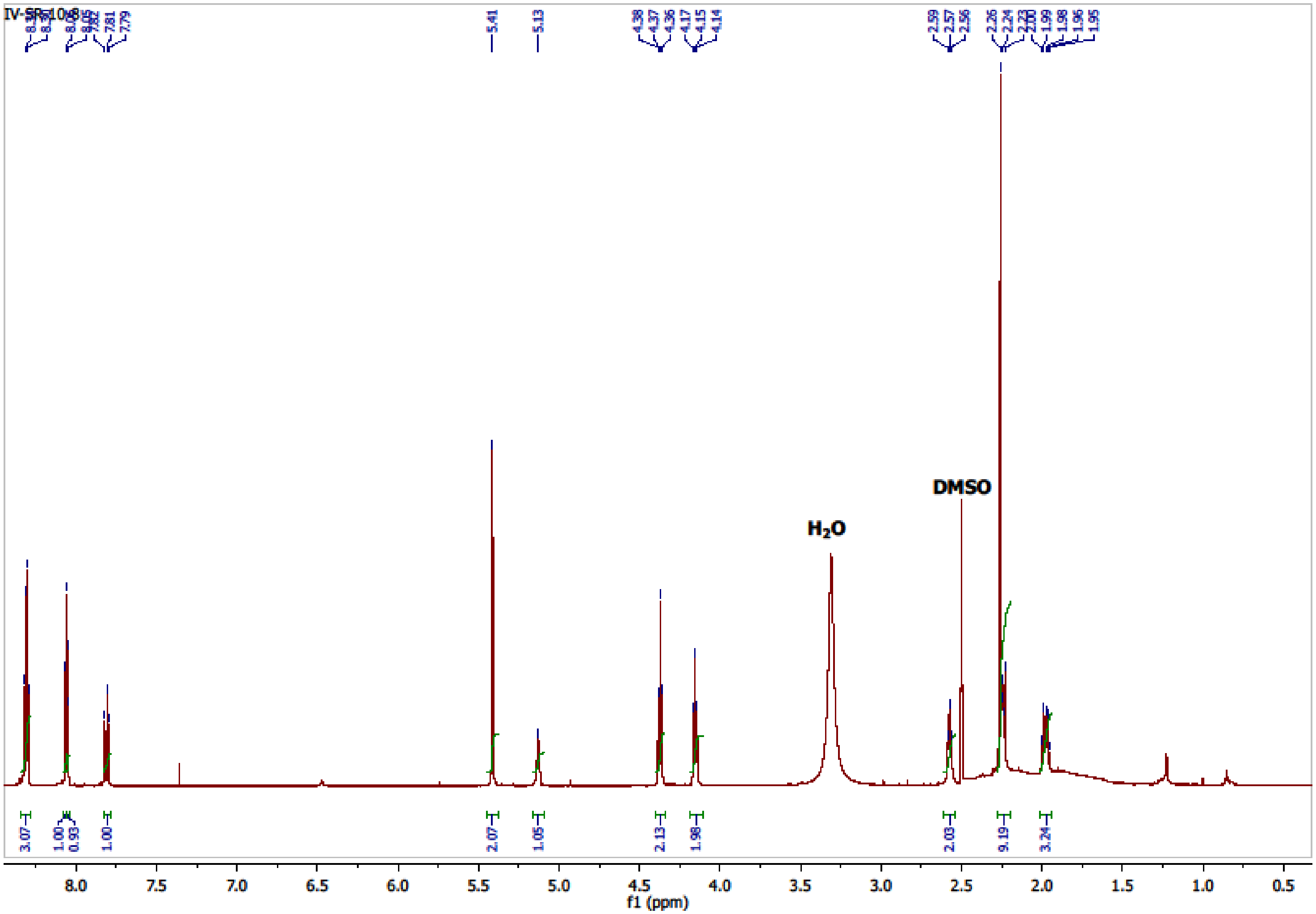


**Figure S58**. ¹H NMR spectrum of **17**.

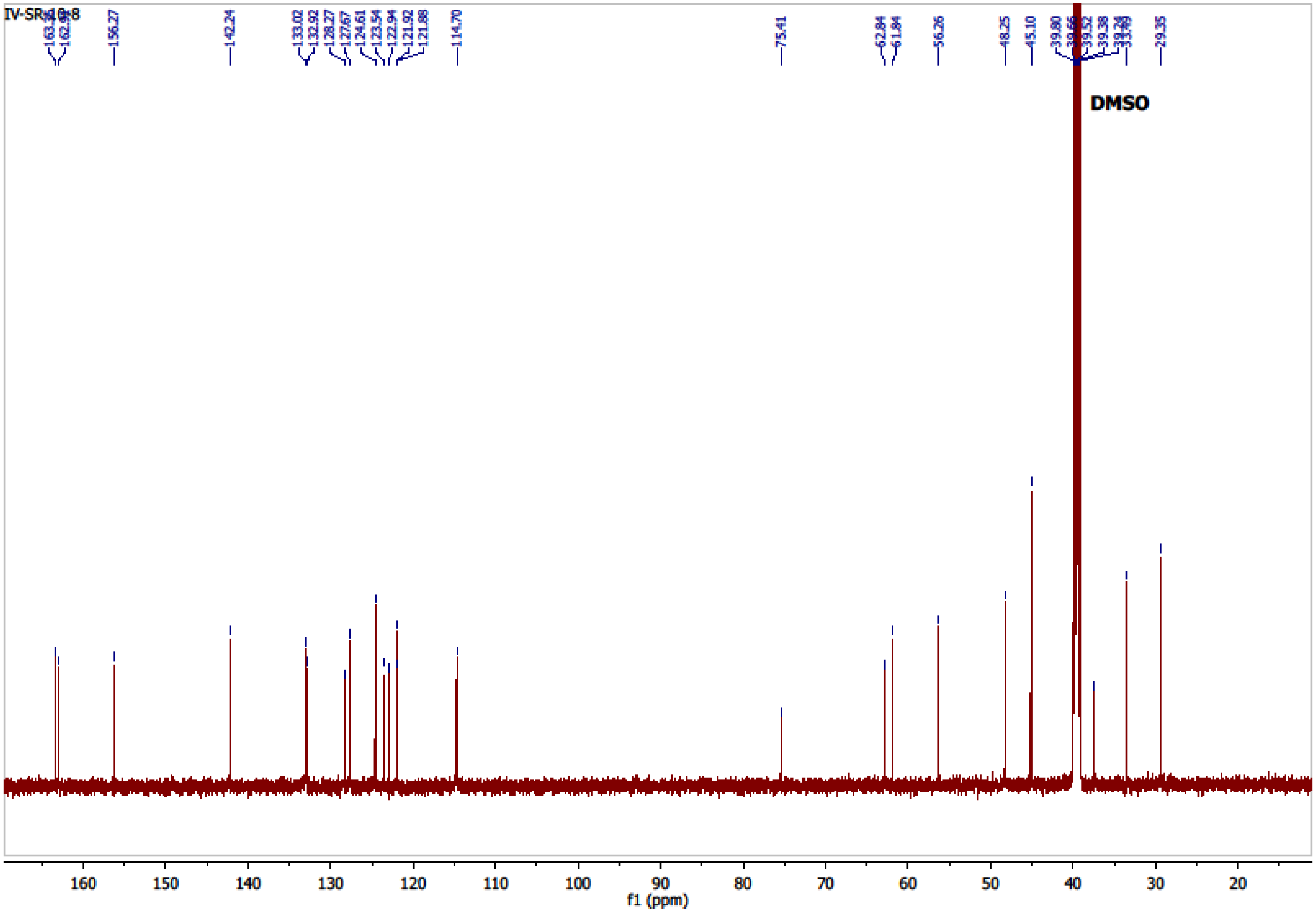


**Figure S59**. $^{13}$C NMR spectrum of **17**.

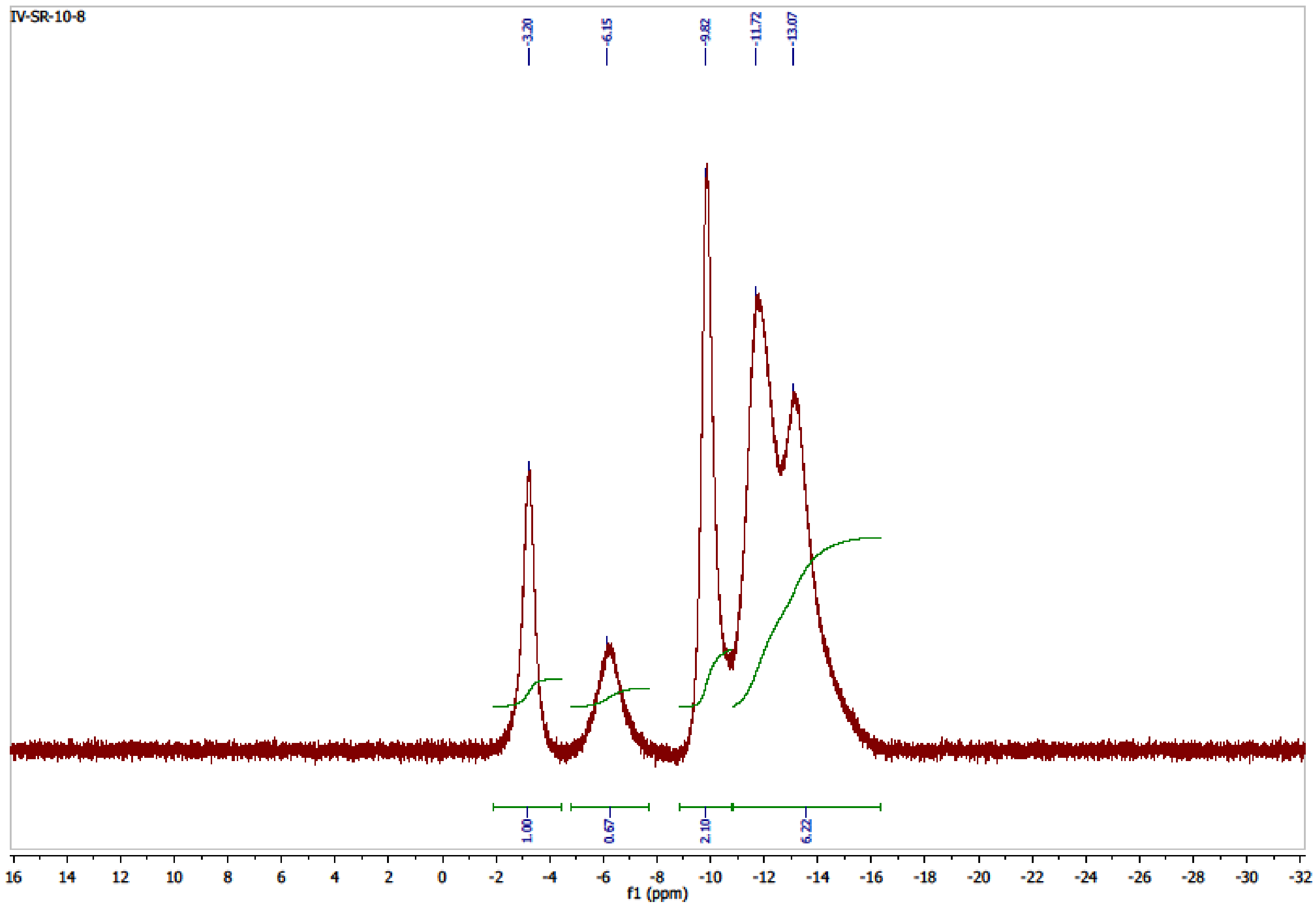


**Figure S60**. $^{11}B$ NMR {$^{1}H$ BB} spectrum of **17**.

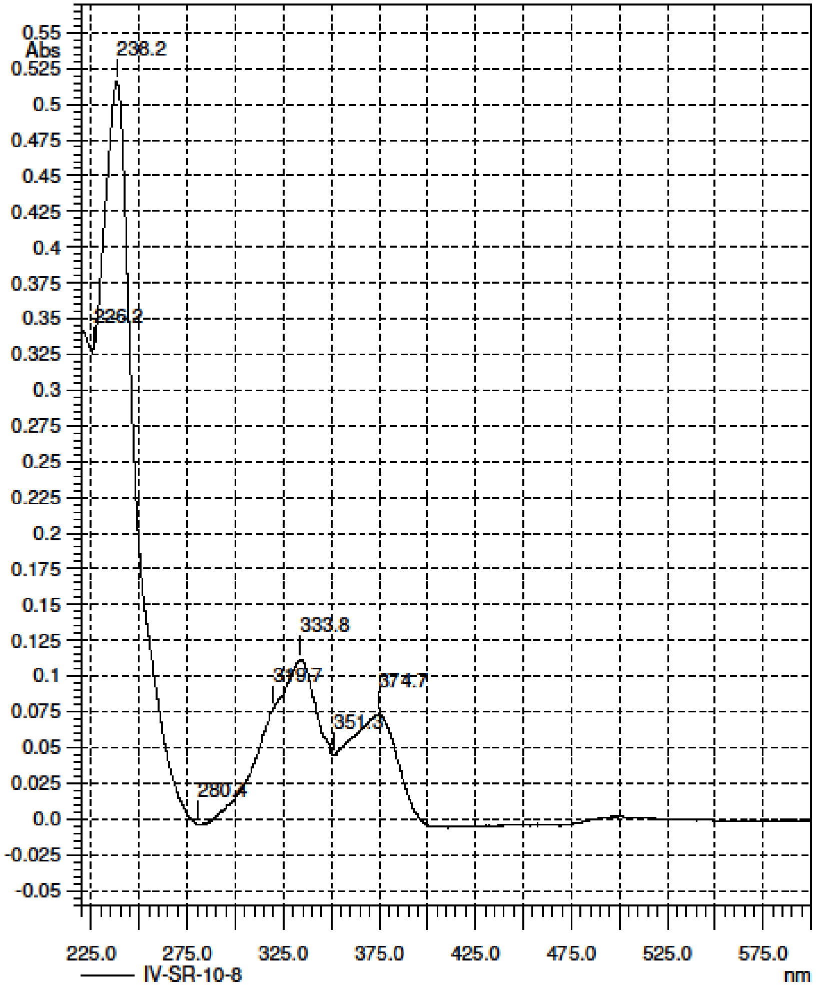


**Figure S61**. UV spectrum of **17**.

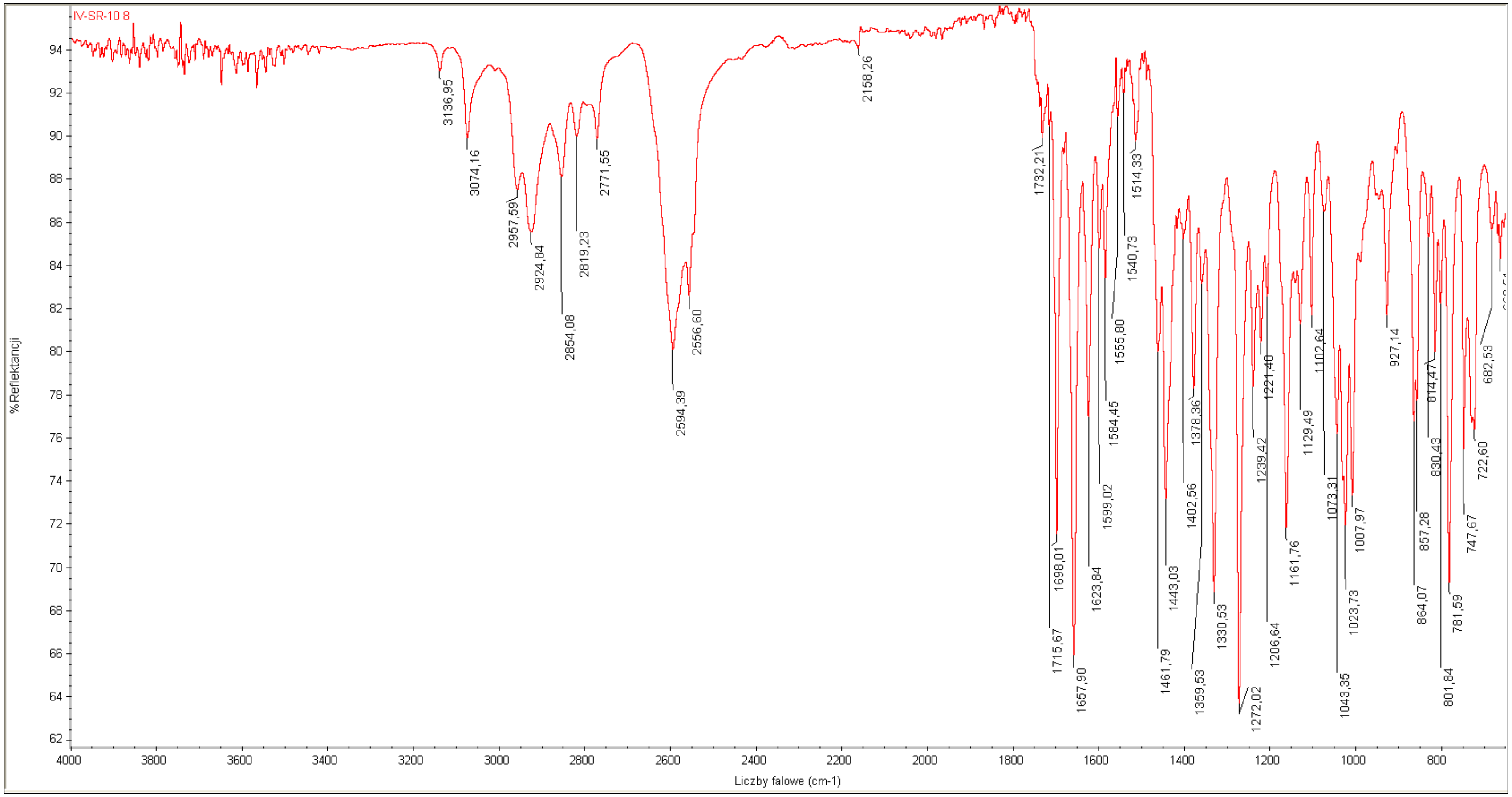


**Figure S62**. IR spectrum of **17**.

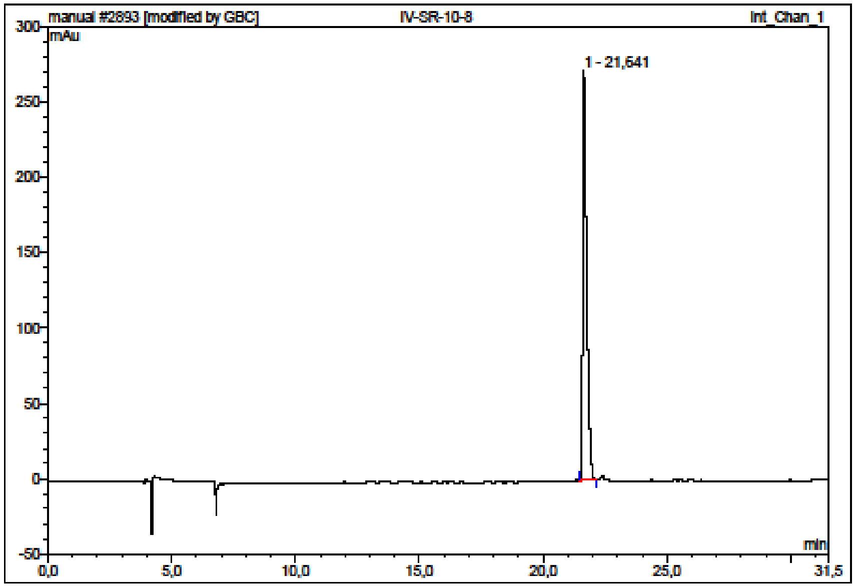


| No. | Ret.Time min | Peak Name | Height mAu | Area mAu*min | Rel.Area % | Amount | Type |
|---|---|---|---|---|---|---|---|
| 1 | 21,64 | n.a. | 272,839 | 51,467 | 100,00 | n.a. | BMB |
| Total: | | | 272,839 | 51,467 | 100,00 | 0,000 | |

**Figure S63**. HPLC analysis of **17**.

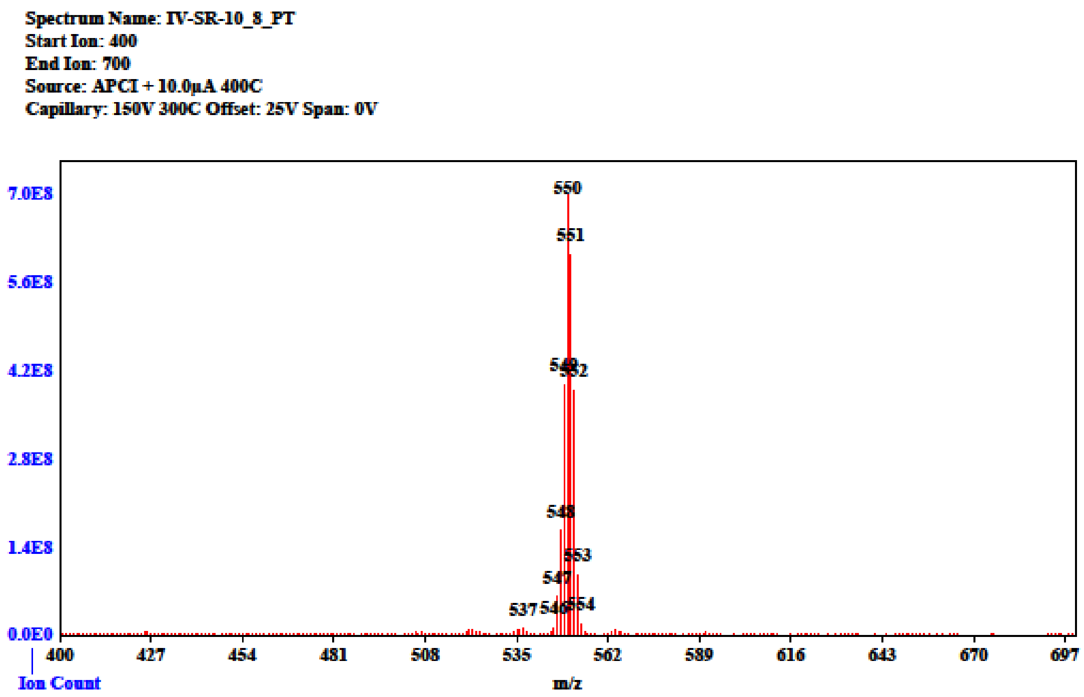


**Figure S64**. MS spectrum of **17**.

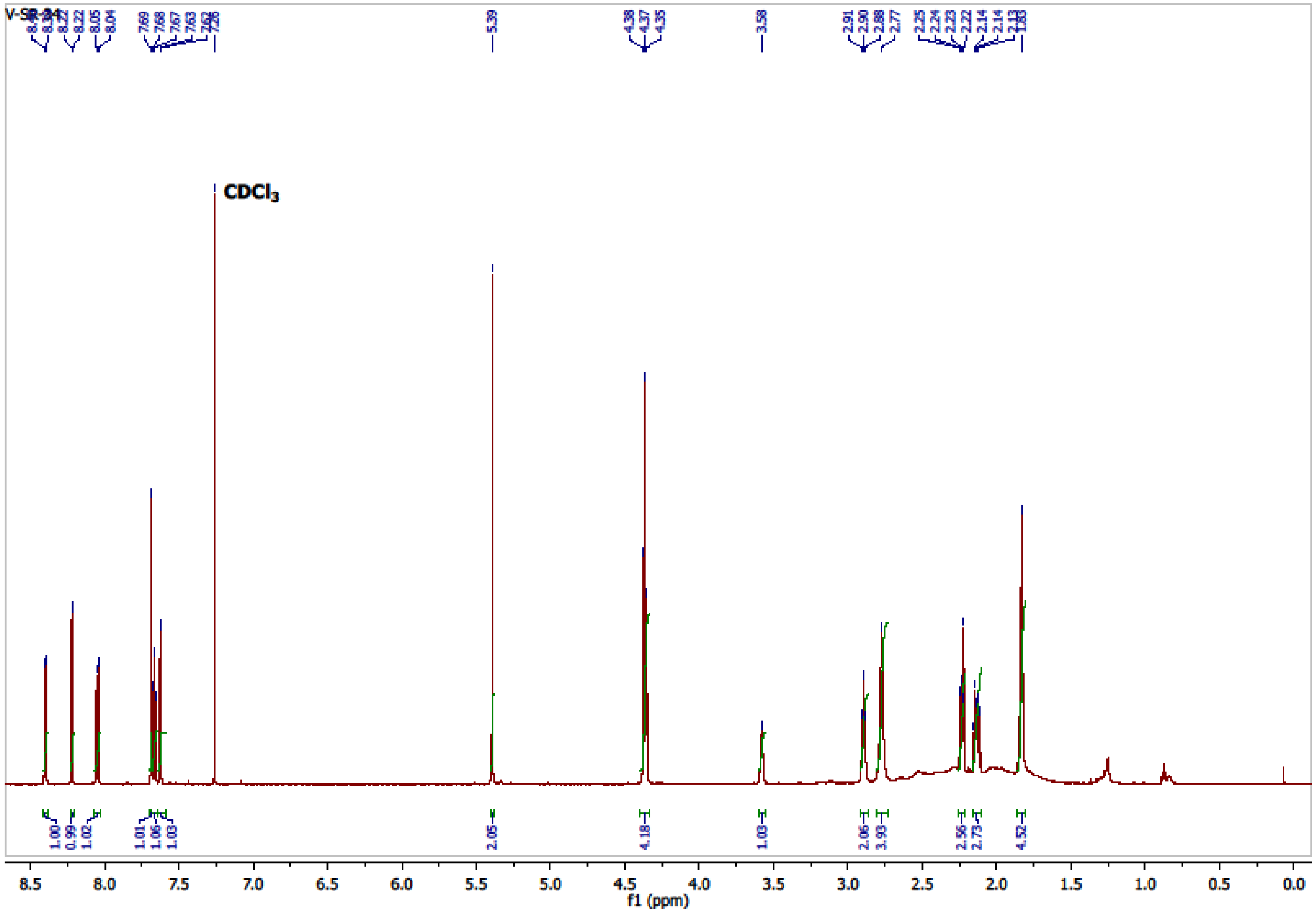

**Figure S65**. $^{1}$H NMR spectrum of **18**.

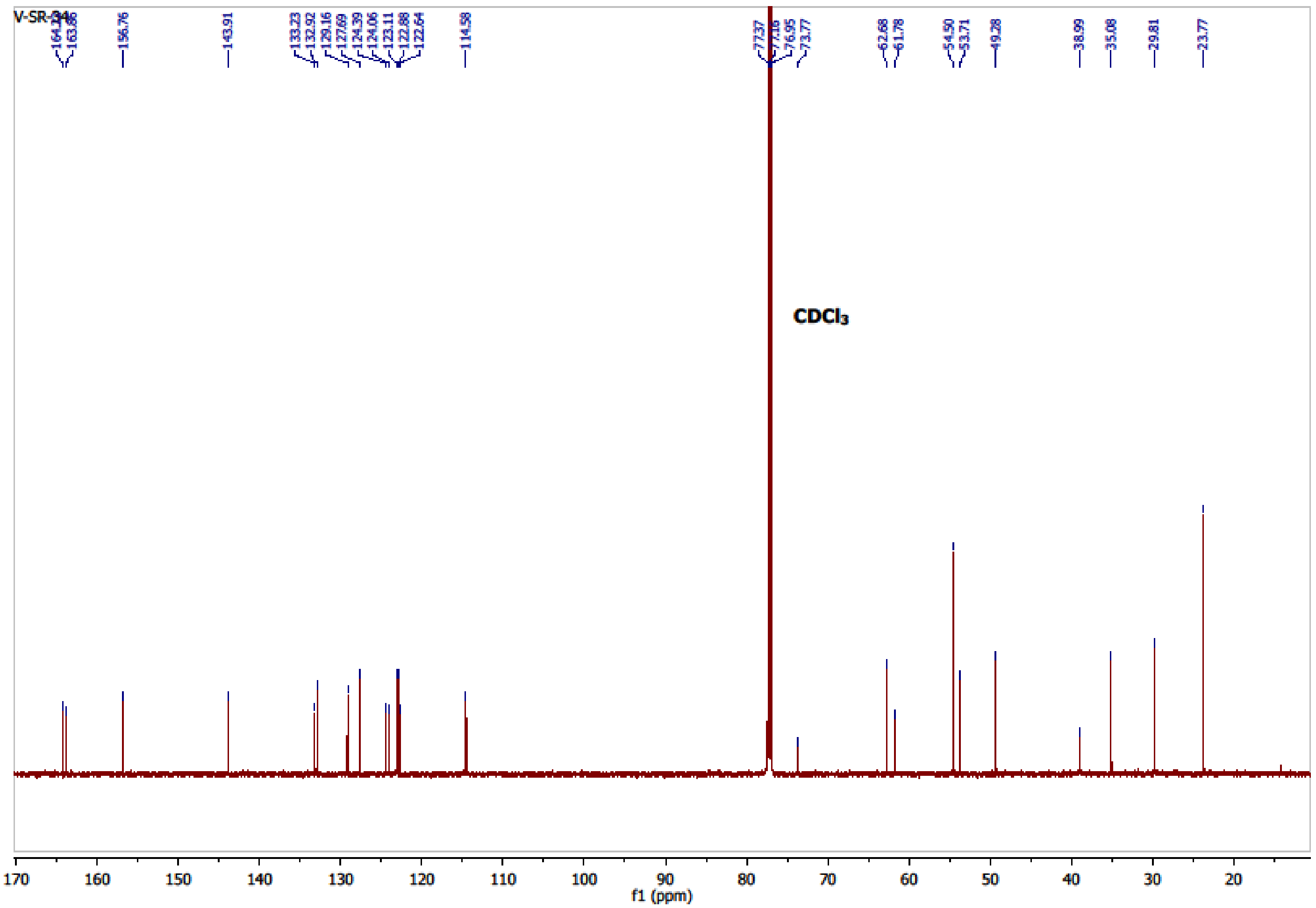


**Figure S66**. $^{13}$C NMR spectrum of **18**.

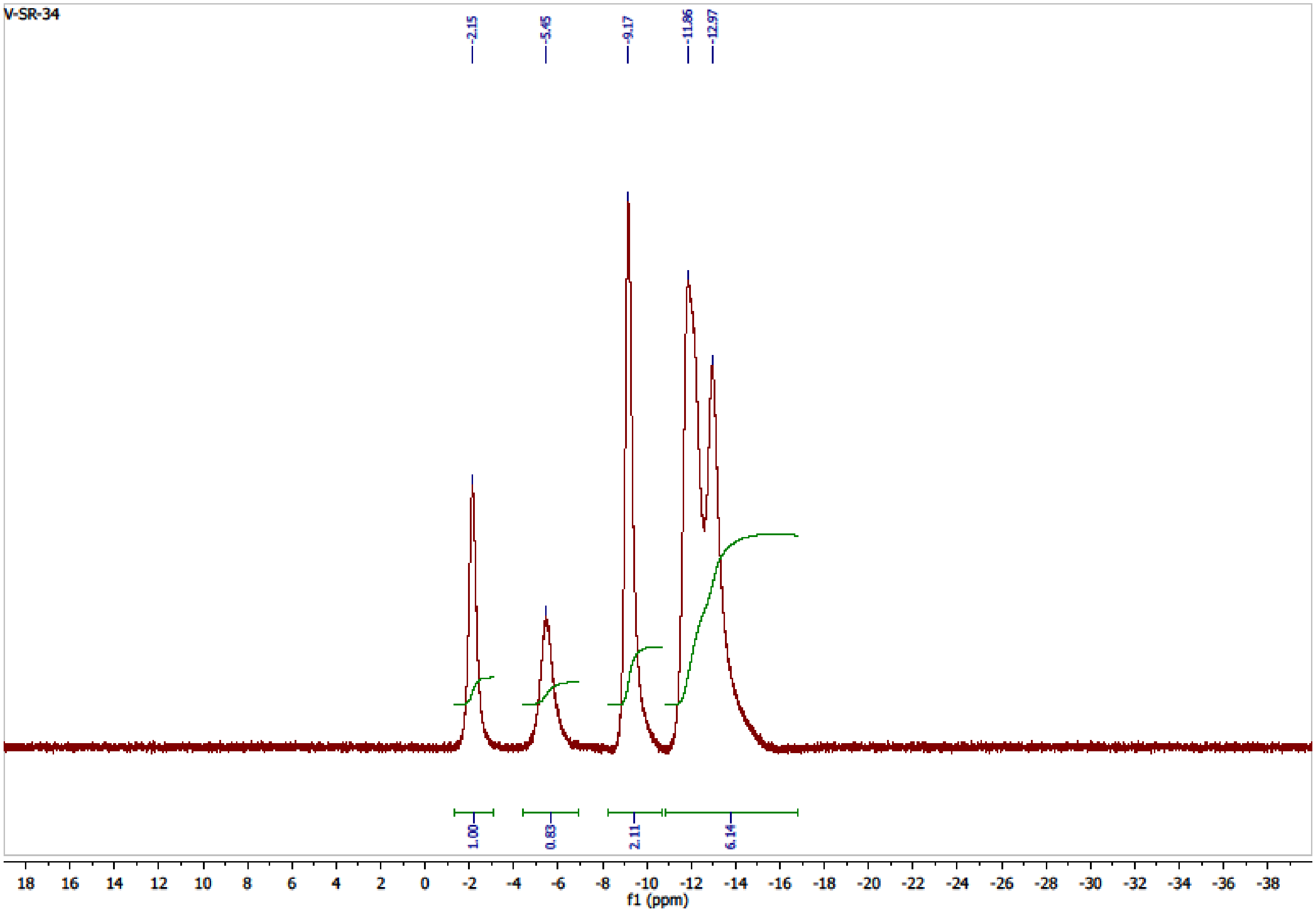


**Figure S67**. $^{11}B$ NMR {$^{1}H$ BB} spectrum of **18**.

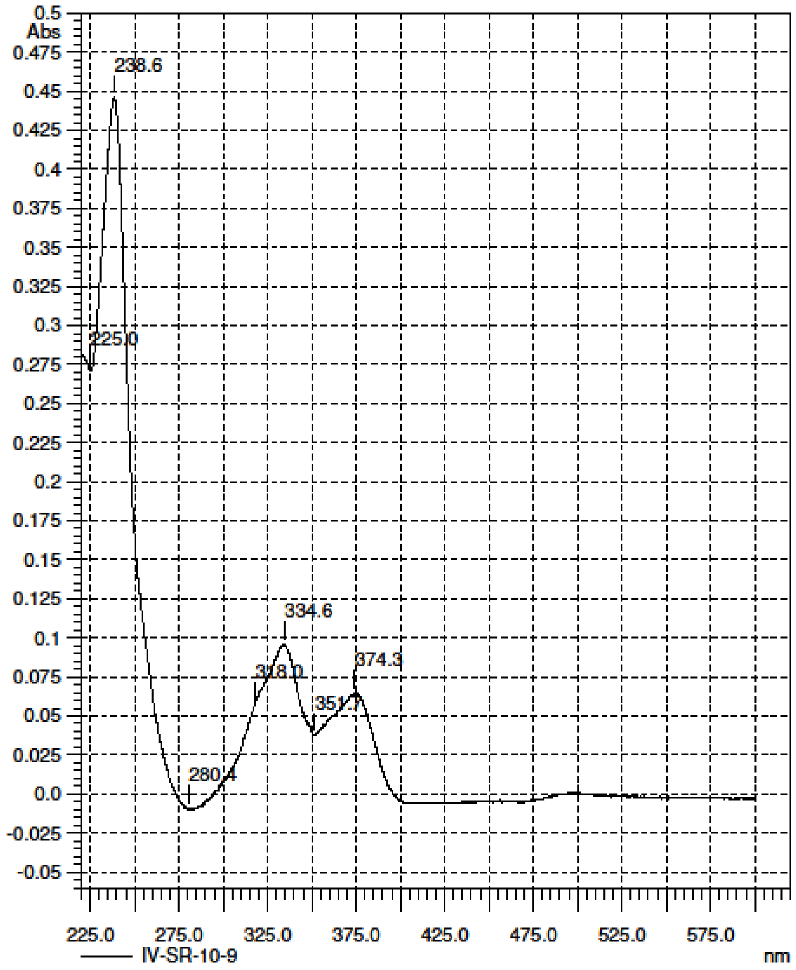


**Figure S68**. UV spectrum of **18**.

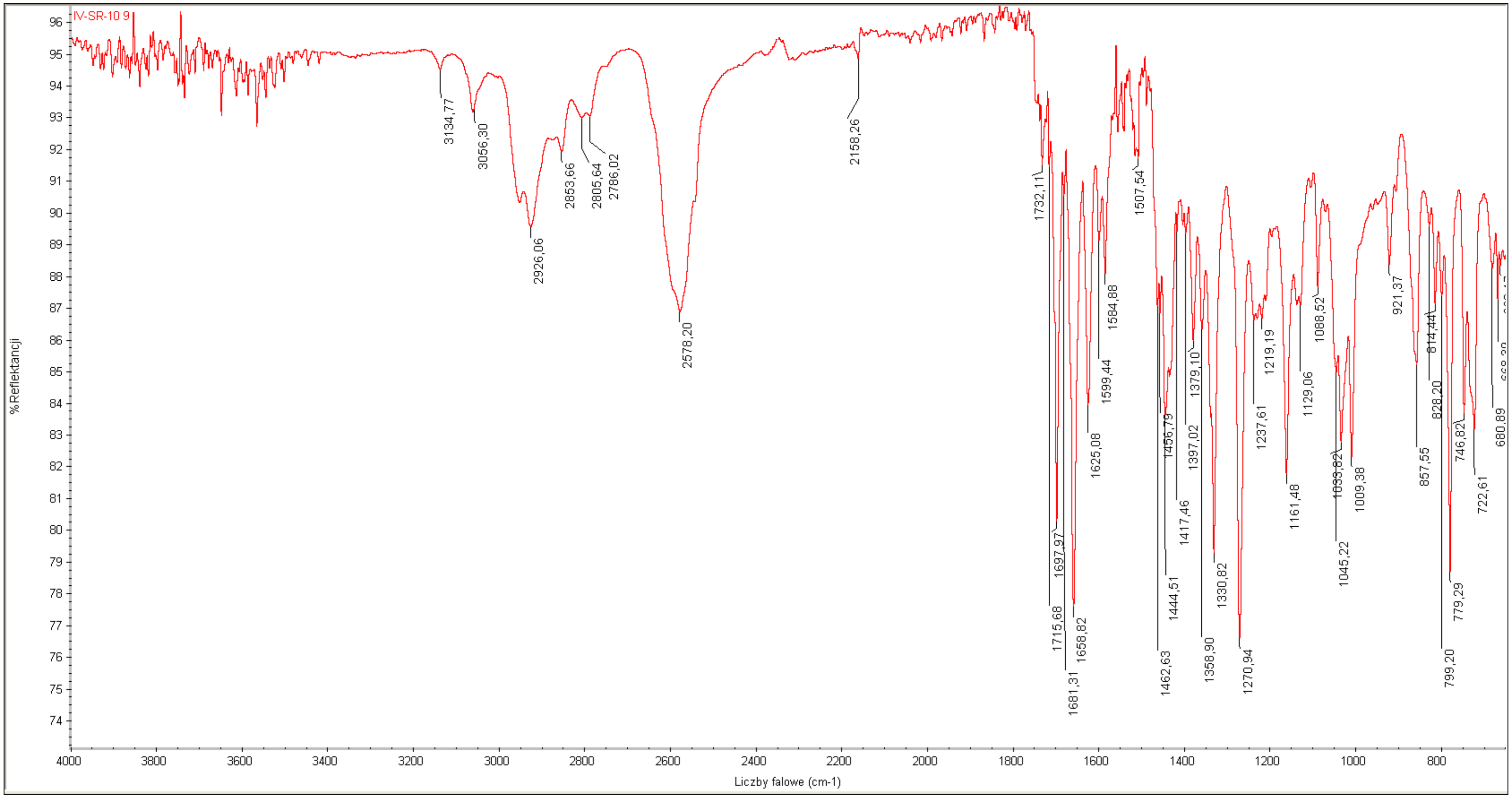


**Figure S69**. IR spectrum of **18**.

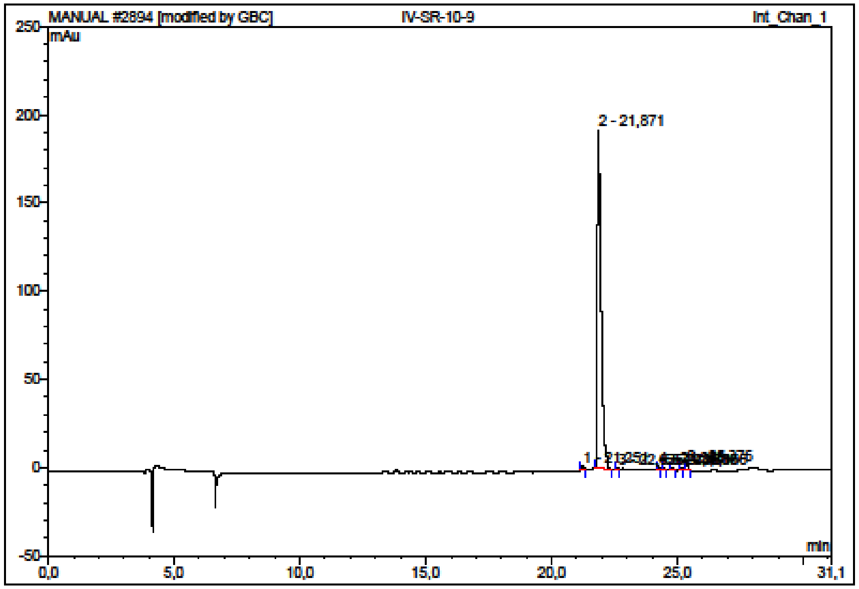


| No. | Ret.Time min | Peak Name | Height mAu | Area mAu*min | Rel.Area % | Amount | Type |
|---|---|---|---|---|---|---|---|
| 1 | 21,25 | n.a. | 1,985 | 0,193 | 0,56 | n.a. | BMB* |
| 2 | 21,87 | n.a. | 191,502 | 33,011 | 96,79 | n.a. | BMB |
| 3 | 22,64 | n.a. | 0,708 | 0,071 | 0,21 | n.a. | BMB* |
| 4 | 24,27 | n.a. | 1,989 | 0,186 | 0,54 | n.a. | BMB* |
| 5 | 24,48 | n.a. | 0,728 | 0,065 | 0,19 | n.a. | BMB* |
| 6 | 24,80 | n.a. | 1,215 | 0,127 | 0,37 | n.a. | BMB* |
| 7 | 25,17 | n.a. | 0,822 | 0,078 | 0,23 | n.a. | BMB* |
| 8 | 25,38 | n.a. | 2,904 | 0,375 | 1,10 | n.a. | BMB* |
| Total: | | | 201,851 | 34,106 | 100,00 | 0,000 | |

**Figure S70**. HPLC analysis of **18**.

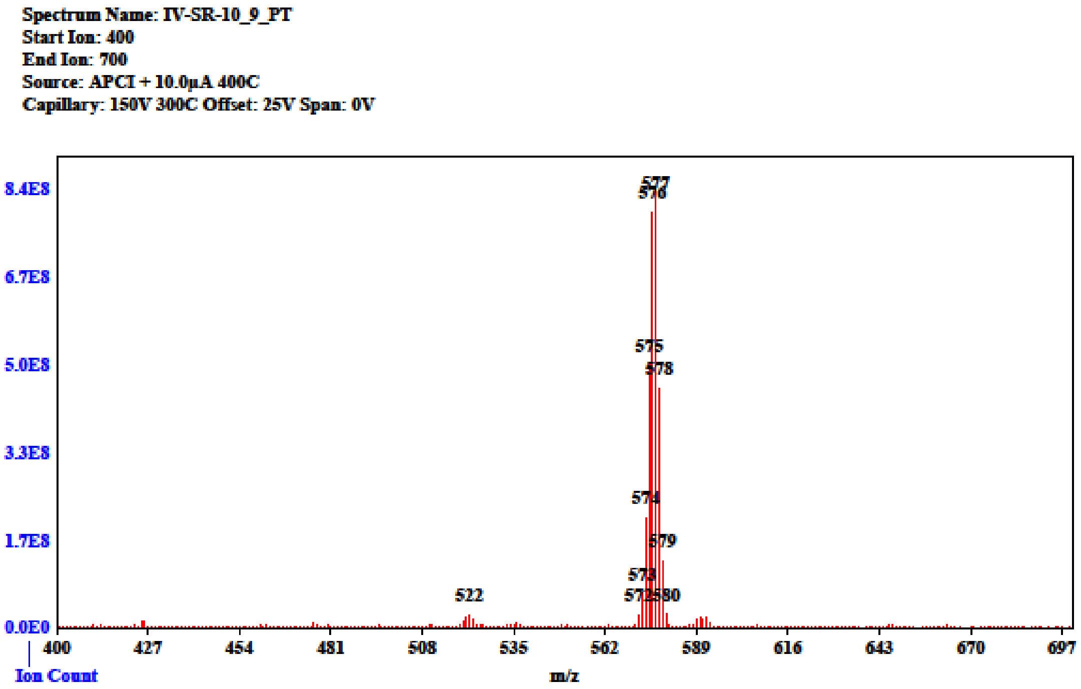


**Figure S71**. MS spectrum of **18**.

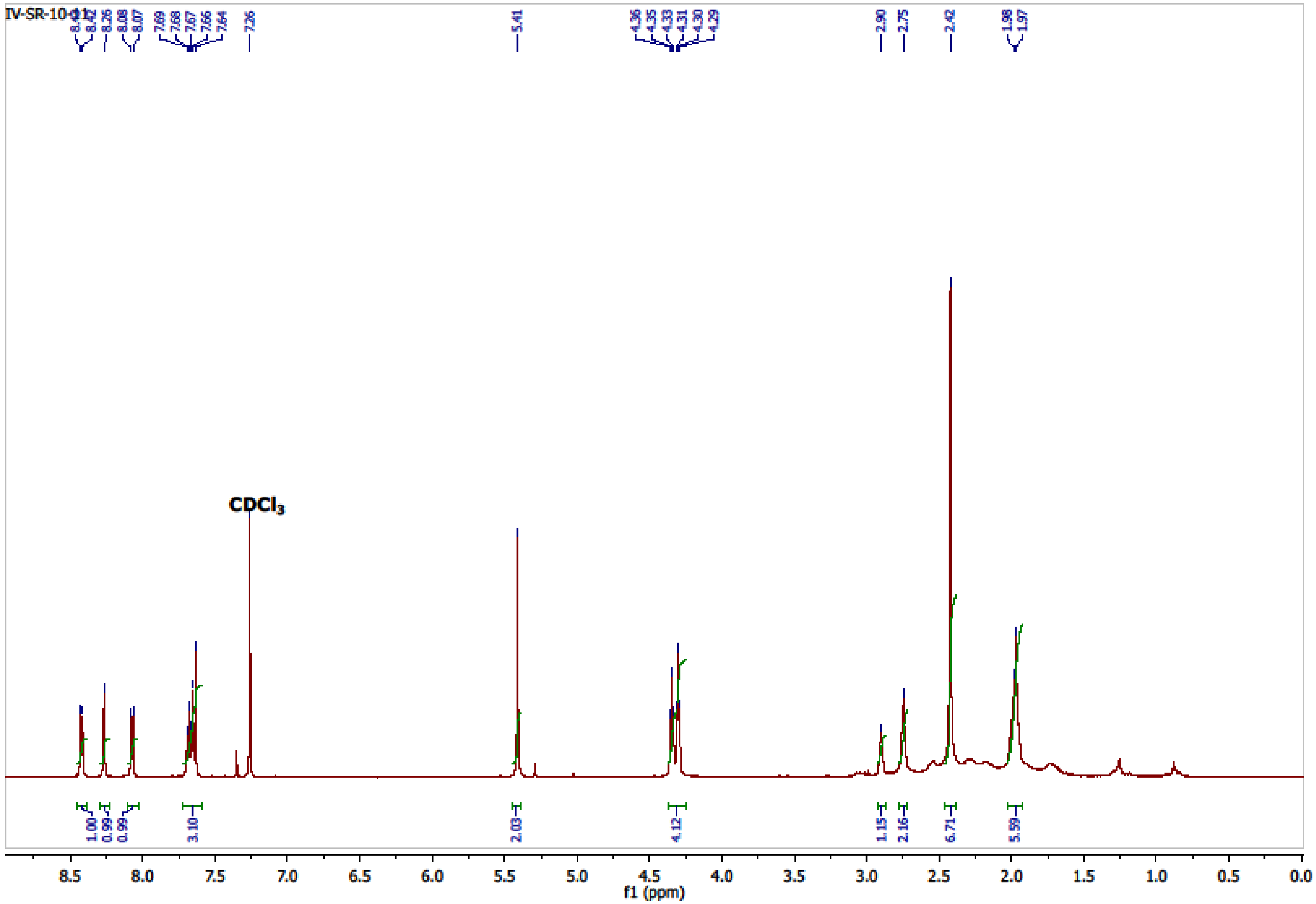


**Figure S72**. $^{1}$H NMR spectrum of **19**.

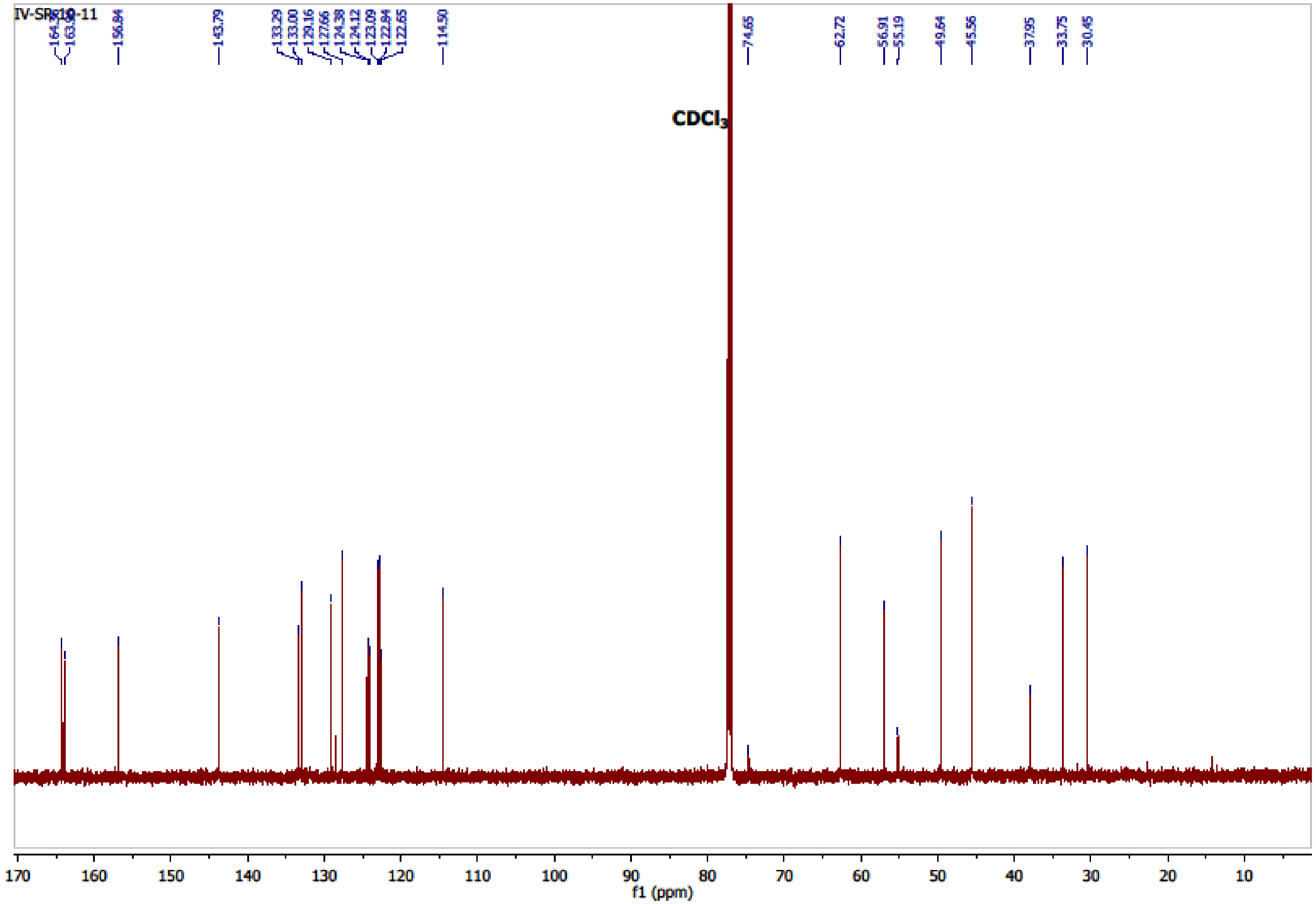


**Figure S73**. $^{13}C$ NMR spectrum of **19**.

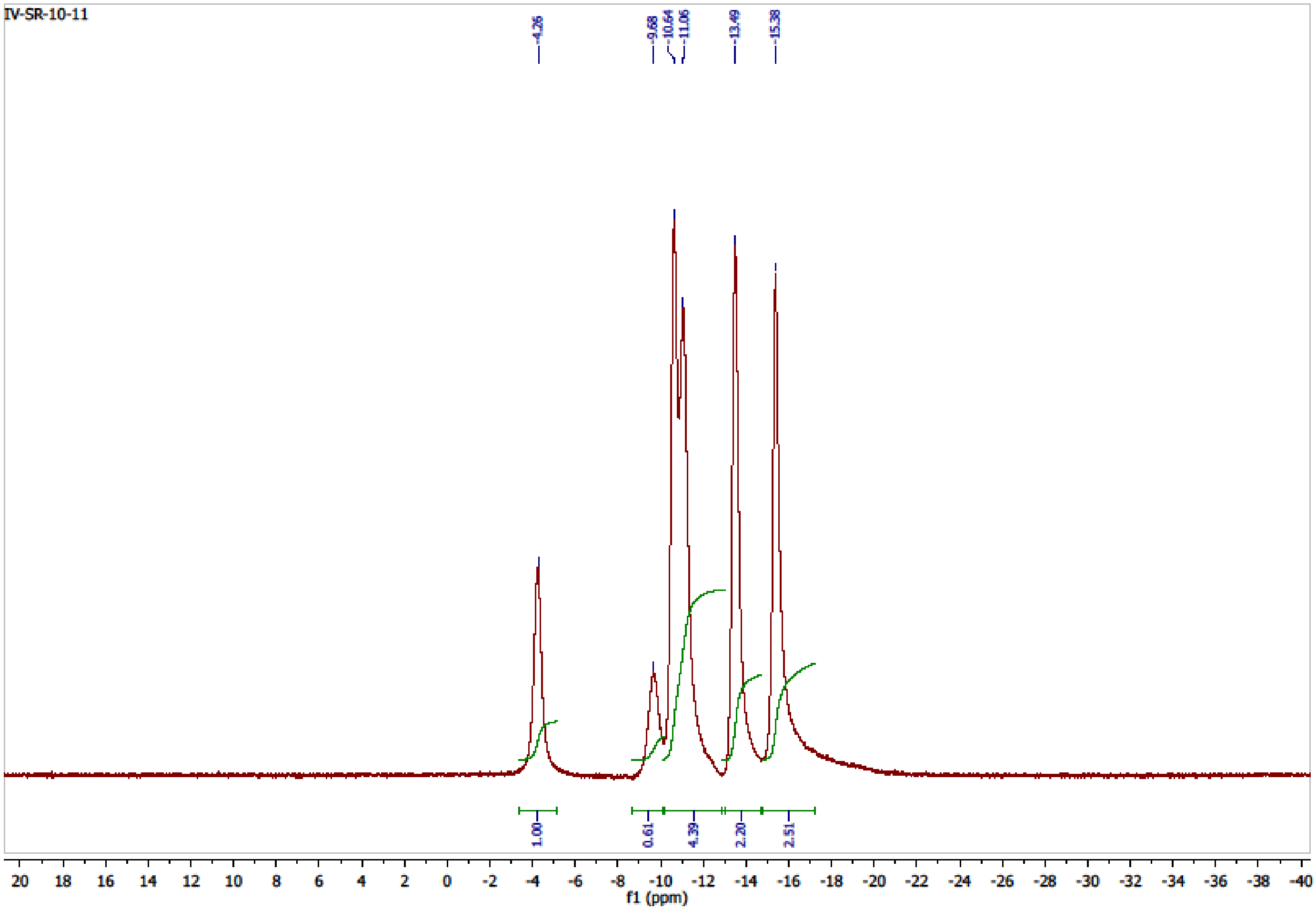


**Figure S74**. $^{11}B$ NMR {$^{1}H$ BB} spectrum of **19**.

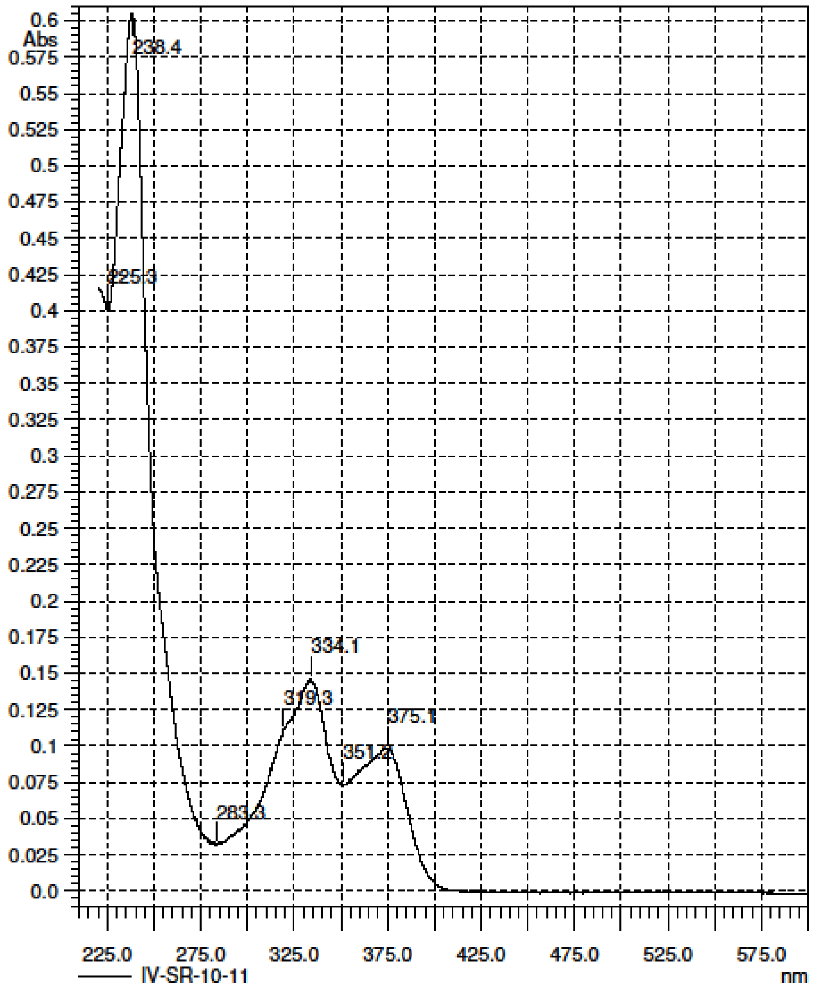


**Figure S75**. UV spectrum of **19**.

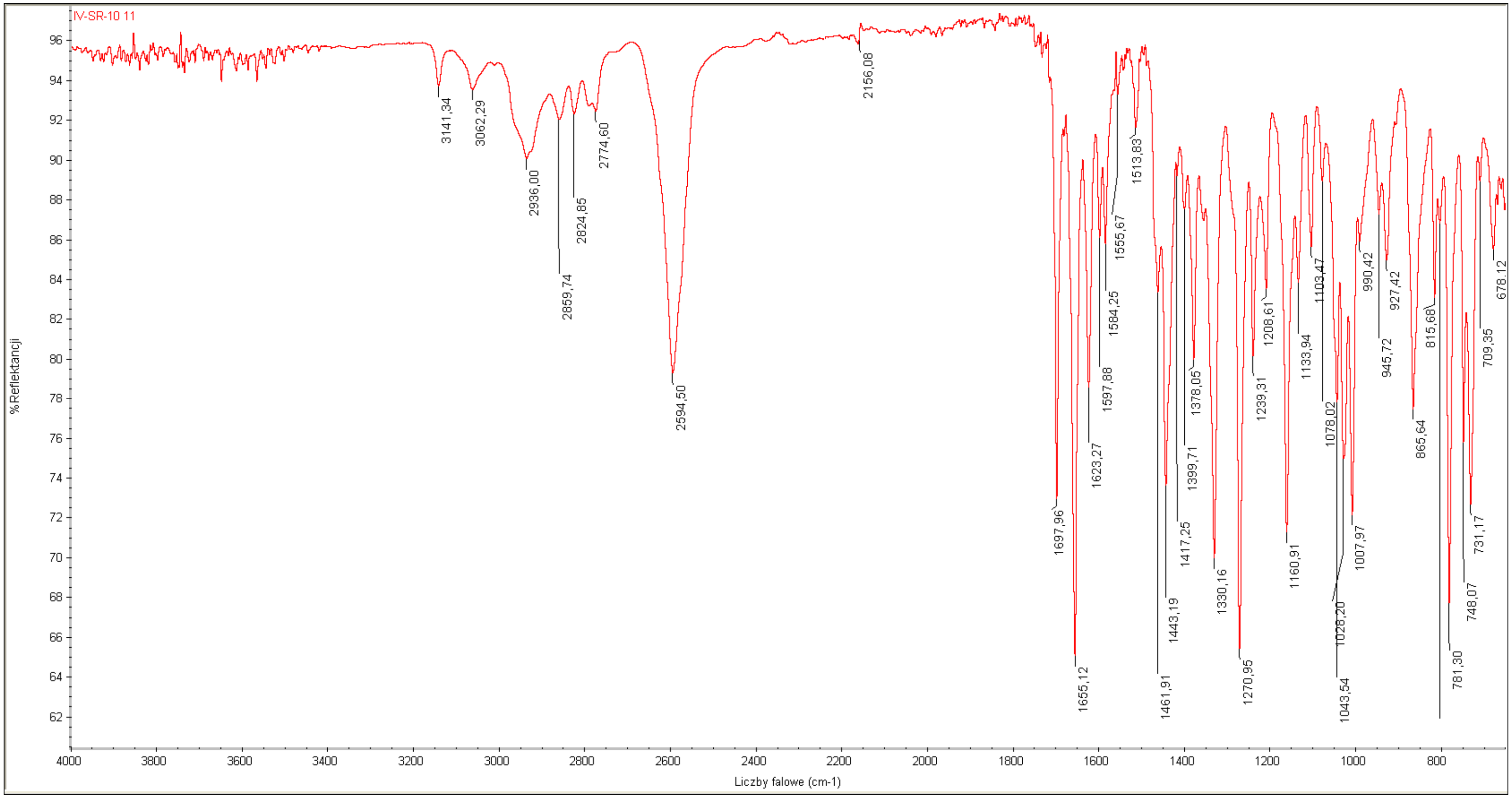


**Figure S76**. IR spectrum of **19**.

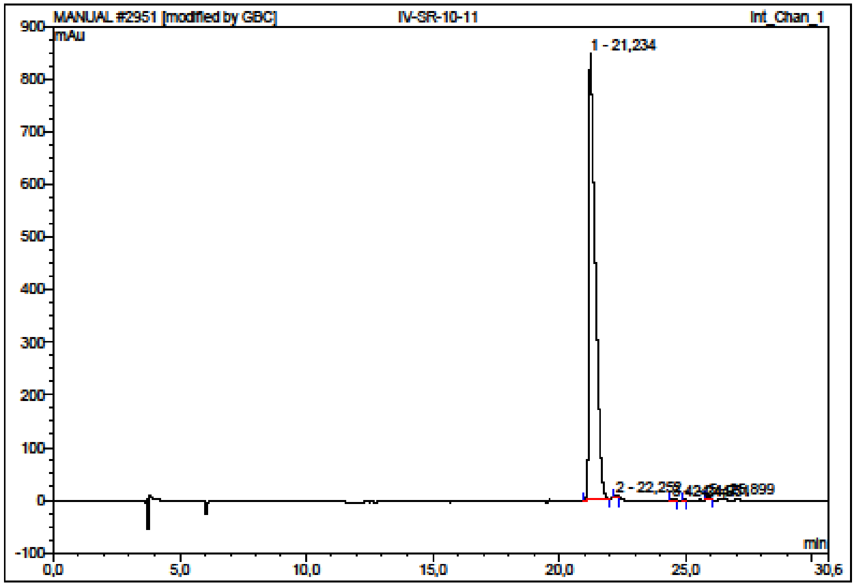


| No. | Ret.Time min | Peak Name | Height mAu | Area mAu*min | Rel.Area % | Amount | Type |
|---|---|---|---|---|---|---|---|
| 1 | 21,23 | n.a. | 849,952 | 248,891 | 99,30 | n.a. | BMB |
| 2 | 22,25 | n.a. | 4,583 | 0,672 | 0,27 | n.a. | BM * |
| 3 | 24,45 | n.a. | 1,821 | 0,243 | 0,10 | n.a. | BMB* |
| 4 | 24,95 | n.a. | 2,623 | 0,272 | 0,11 | n.a. | BMB* |
| 5 | 25,90 | n.a. | 4,032 | 0,565 | 0,23 | n.a. | BMB* |
| Total: | | | 863,010 | 250,642 | 100,00 | 0,000 | |

**Figure S77**. HPLC analysis of **19**.

**Spectrum Name: IV-SR-10_11_PT**
**Start Ion: 400**
**End Ion: 700**
**Source: APCI + 10.0µA 400C**
**Capillary: 150V 300C Offset: 25V Span: 0V**

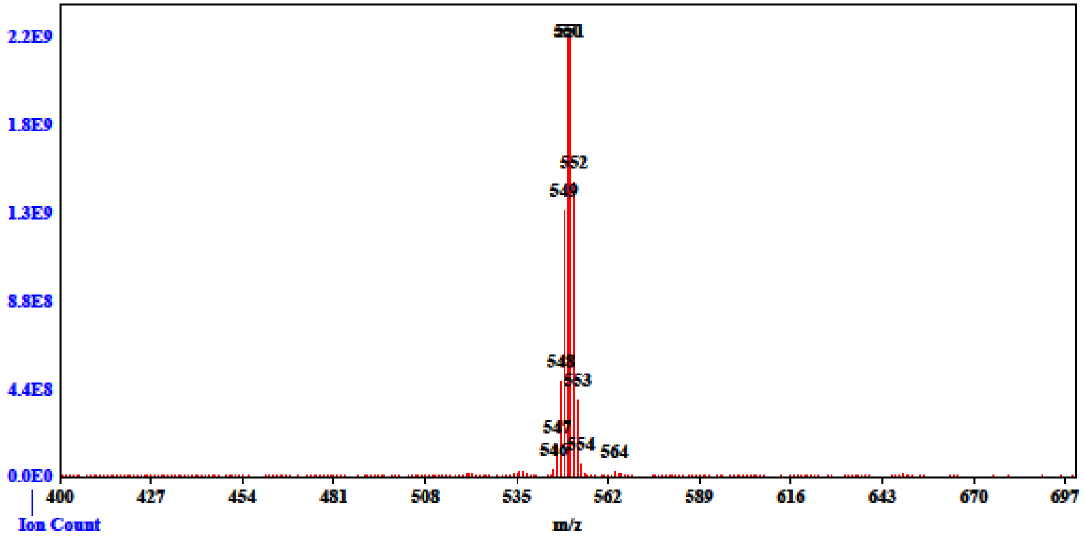


**Figure S78**. MS spectrum of **19**.

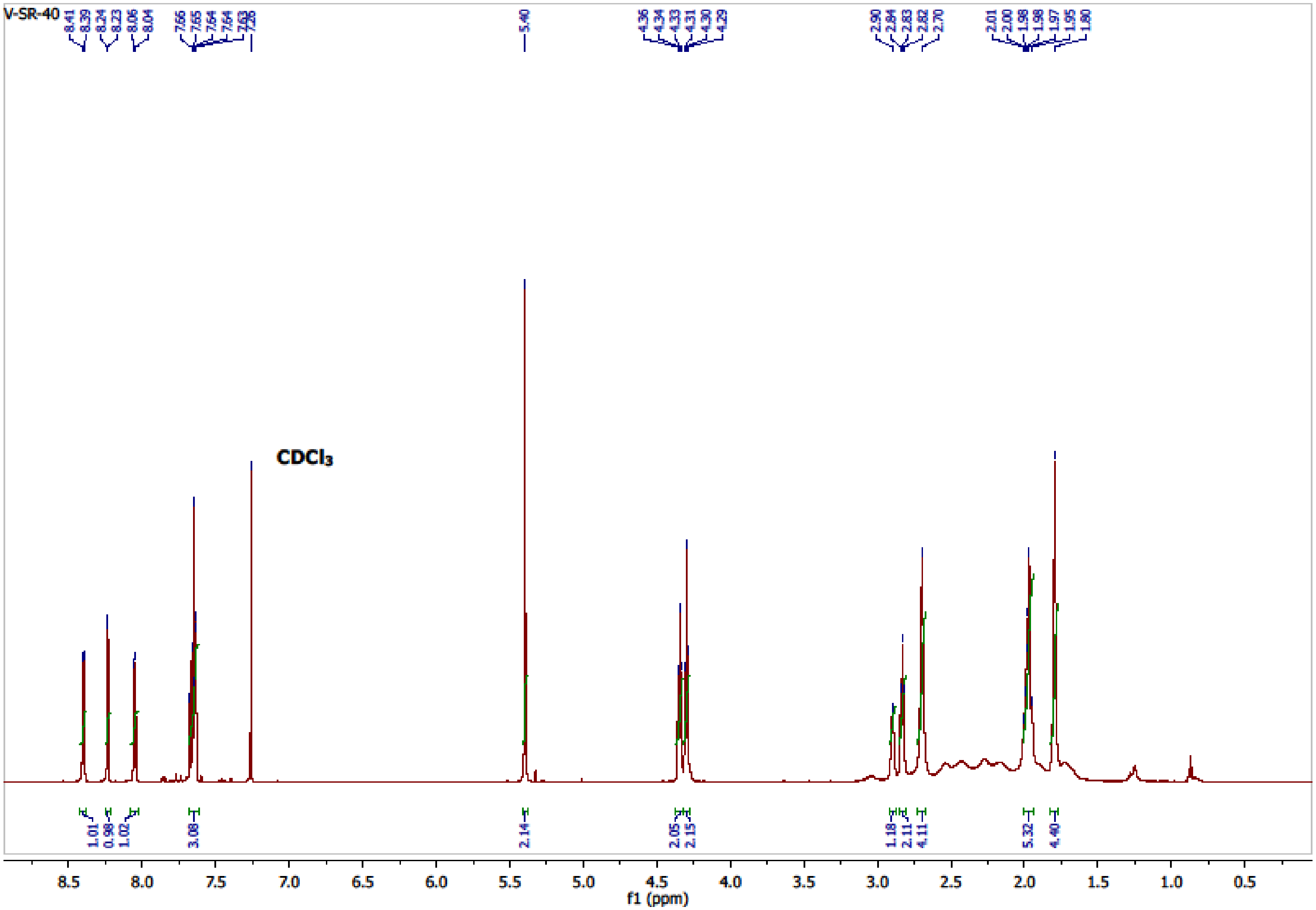


**Figure S79**. $^{1}$H NMR spectrum of **20**.

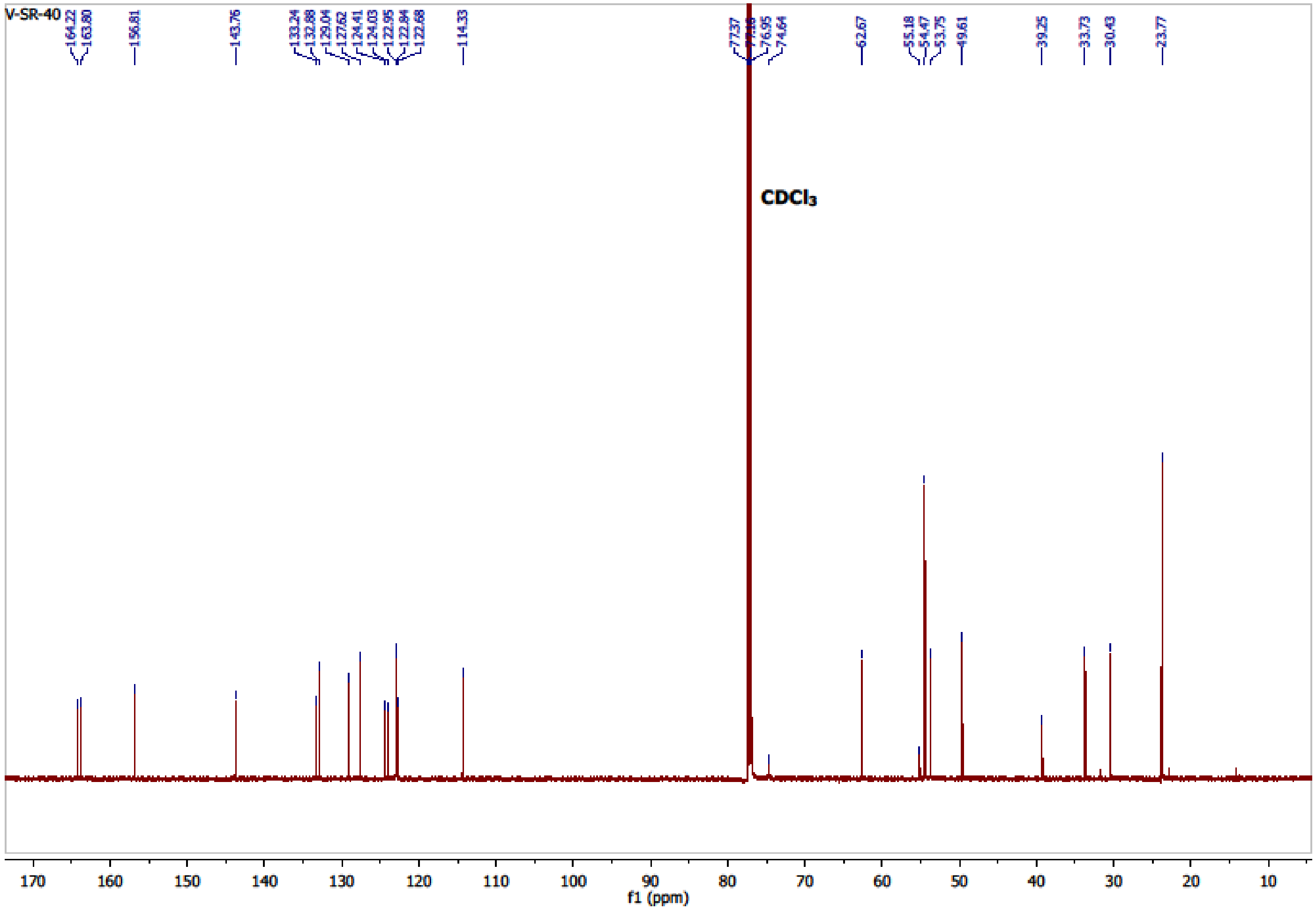


**Figure S80**. $^{13}C$ NMR spectrum of **20**.

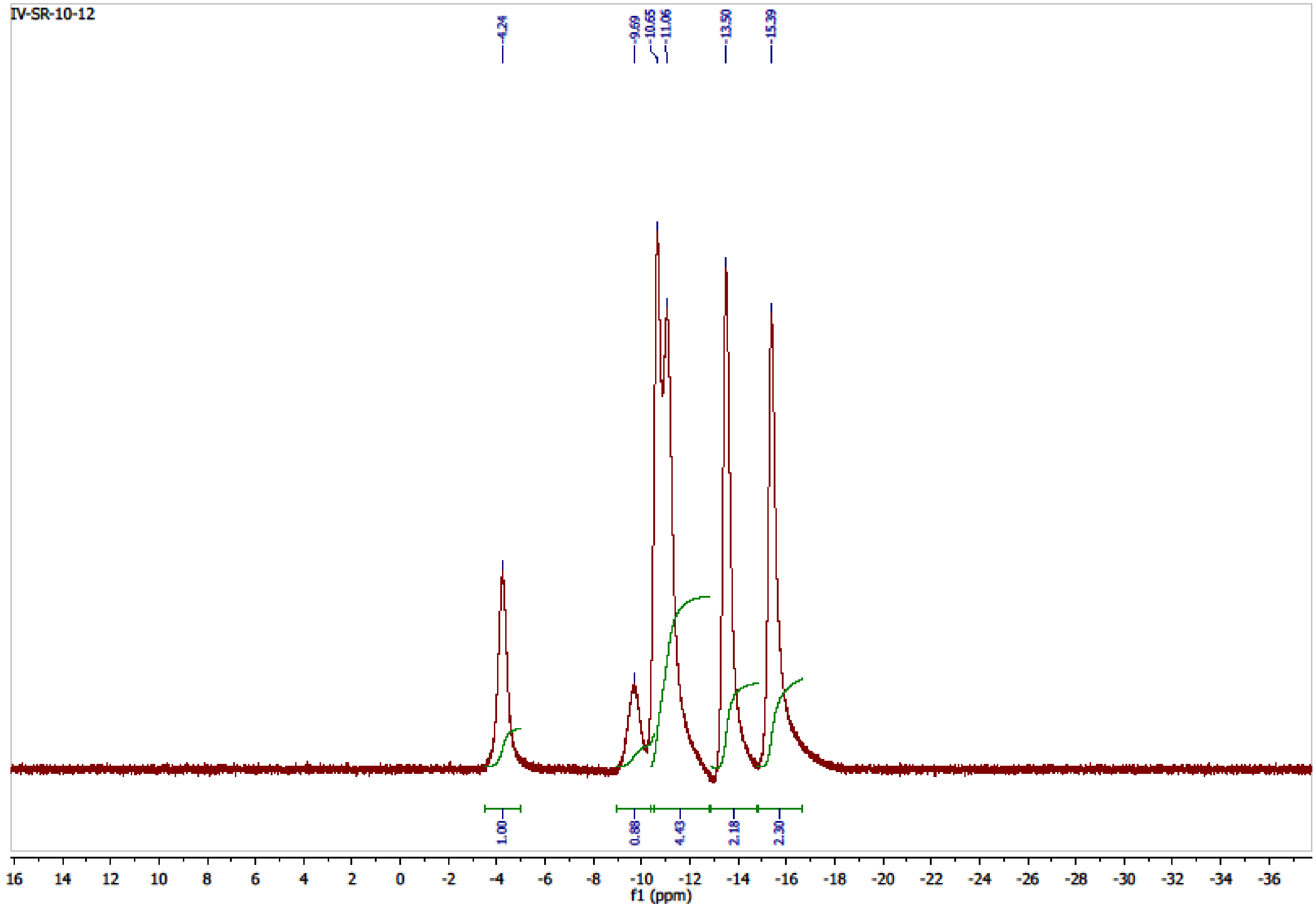


**Figure S81**. $^{11}B$ NMR {$^{1}H$ BB} spectrum of **20**.

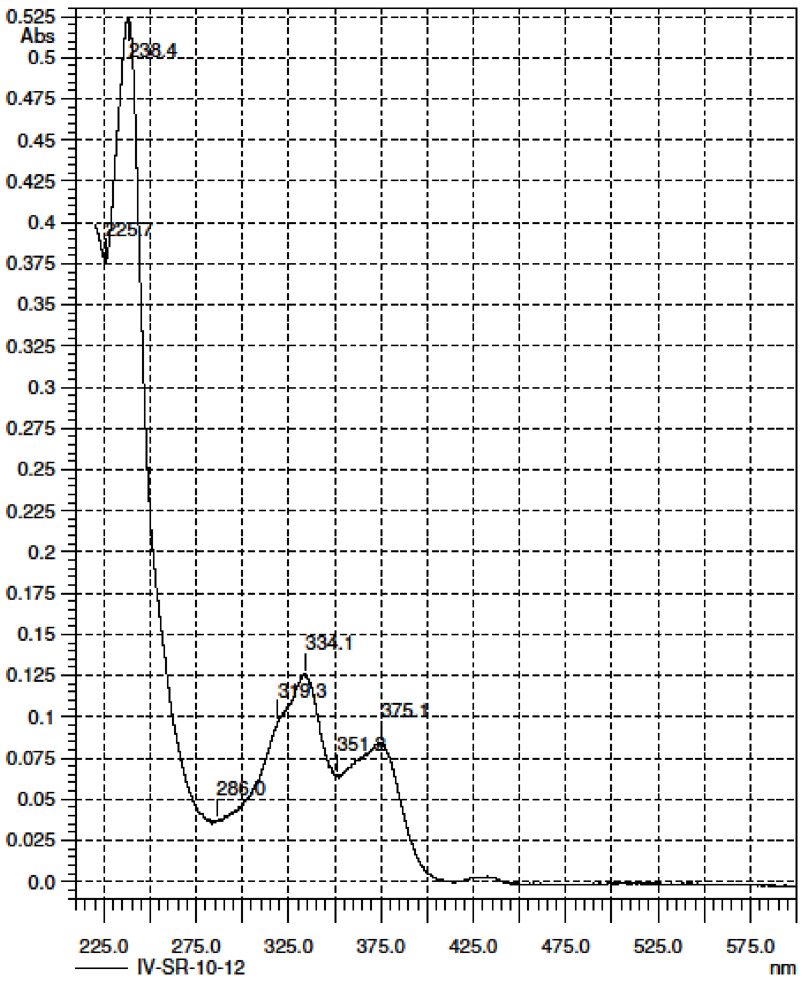


**Figure S82**. UV spectrum of **20**.

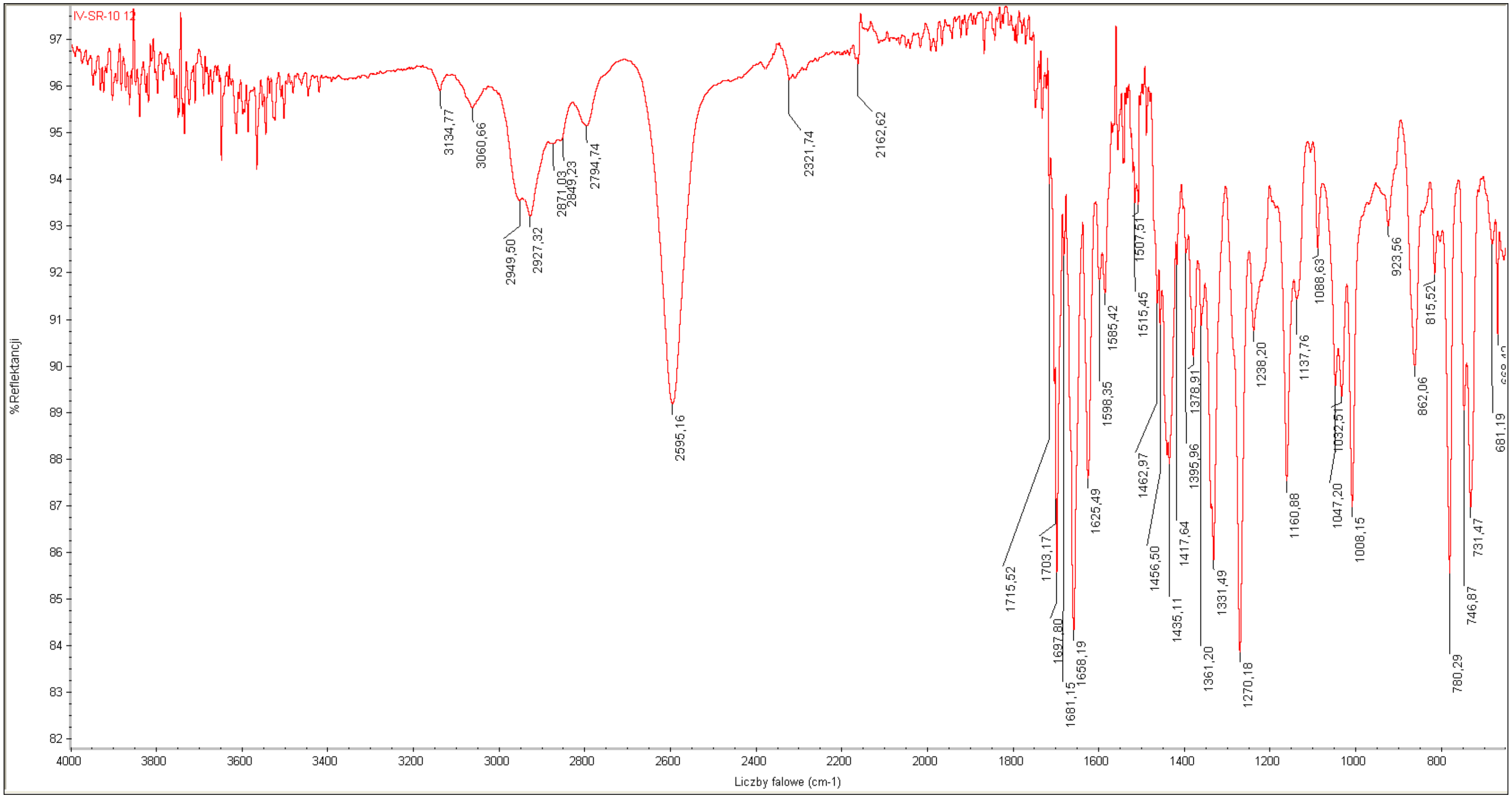


**Figure S83**. IR spectrum of **20**.

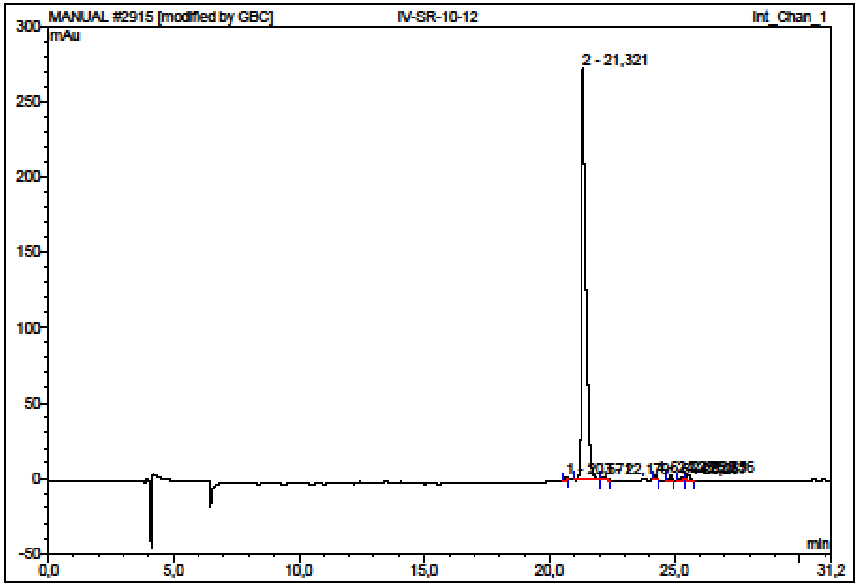


| No. | Ret.Time min | Peak Name | Height mAu | Area mAu*min | Rel.Area % | Amount | Type |
|---|---|---|---|---|---|---|---|
| 1 | 20,67 | n.a. | 2,376 | 0,234 | 0,40 | n.a. | BMB* |
| 2 | 21,32 | n.a. | 272,962 | 56,512 | 96,19 | n.a. | BMB* |
| 3 | 22,18 | n.a. | 1,980 | 0,366 | 0,62 | n.a. | BMB* |
| 4 | 24,25 | n.a. | 2,976 | 0,286 | 0,49 | n.a. | BMB* |
| 5 | 24,82 | n.a. | 2,954 | 0,408 | 0,69 | n.a. | BMB* |
| 6 | 25,27 | n.a. | 2,065 | 0,283 | 0,48 | n.a. | BMb* |
| 7 | 25,56 | n.a. | 3,923 | 0,659 | 1,12 | n.a. | bMB* |
| Total: | | | 289,235 | 58,748 | 100,00 | 0,000 | |

**Figure S84**. HPLC analysis of **20**.

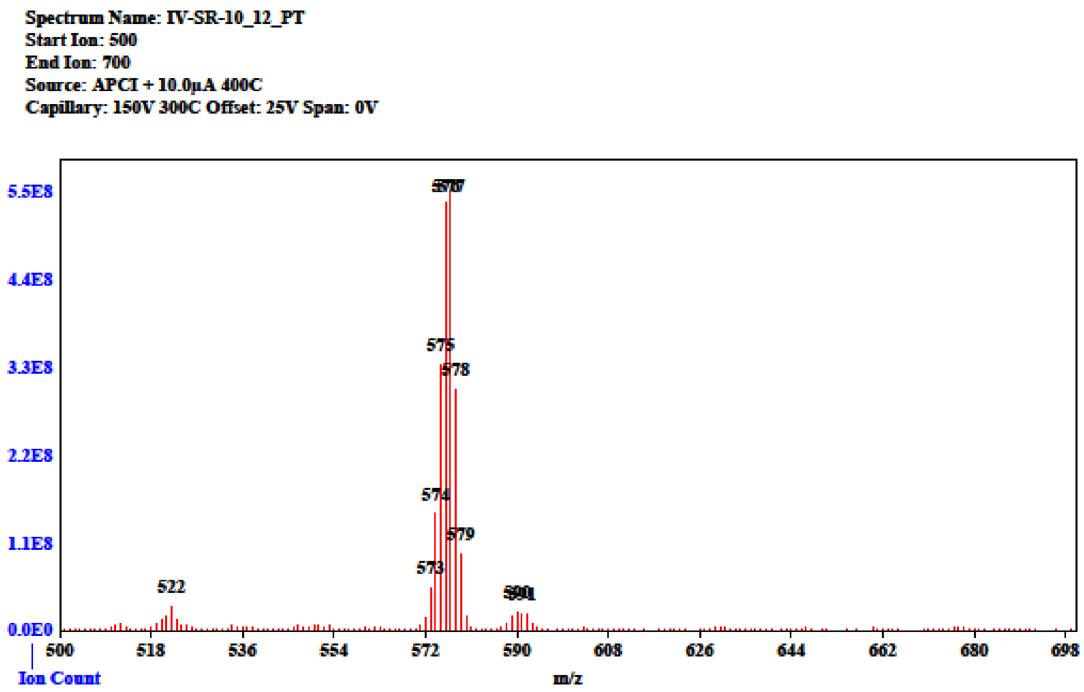


**Figure S85**. MS spectrum of **20**.

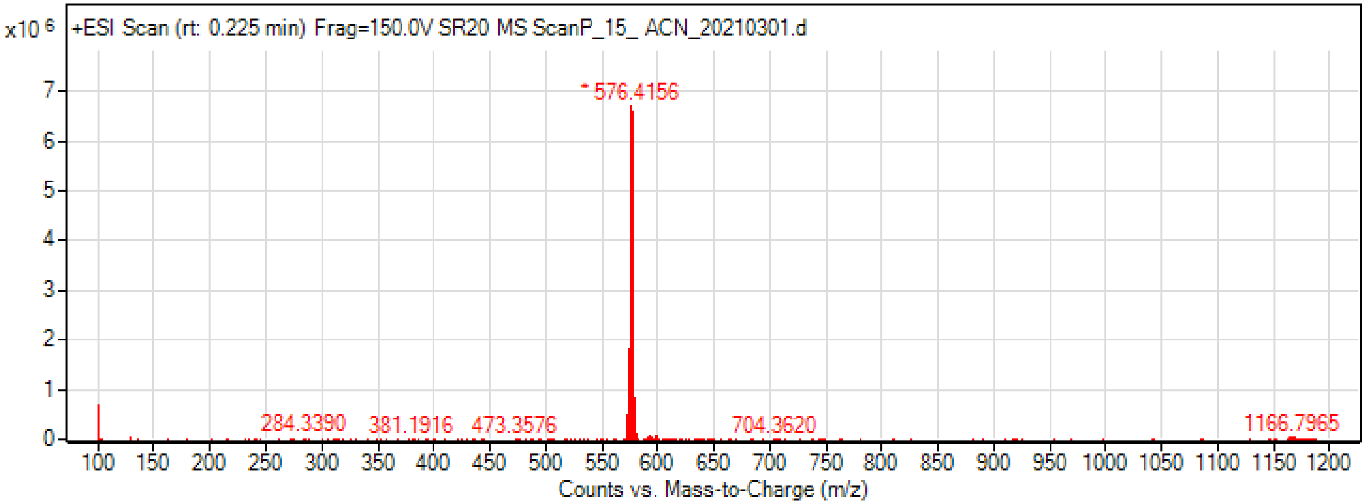


**Figure S86**. HRMS spectrum of **20**.

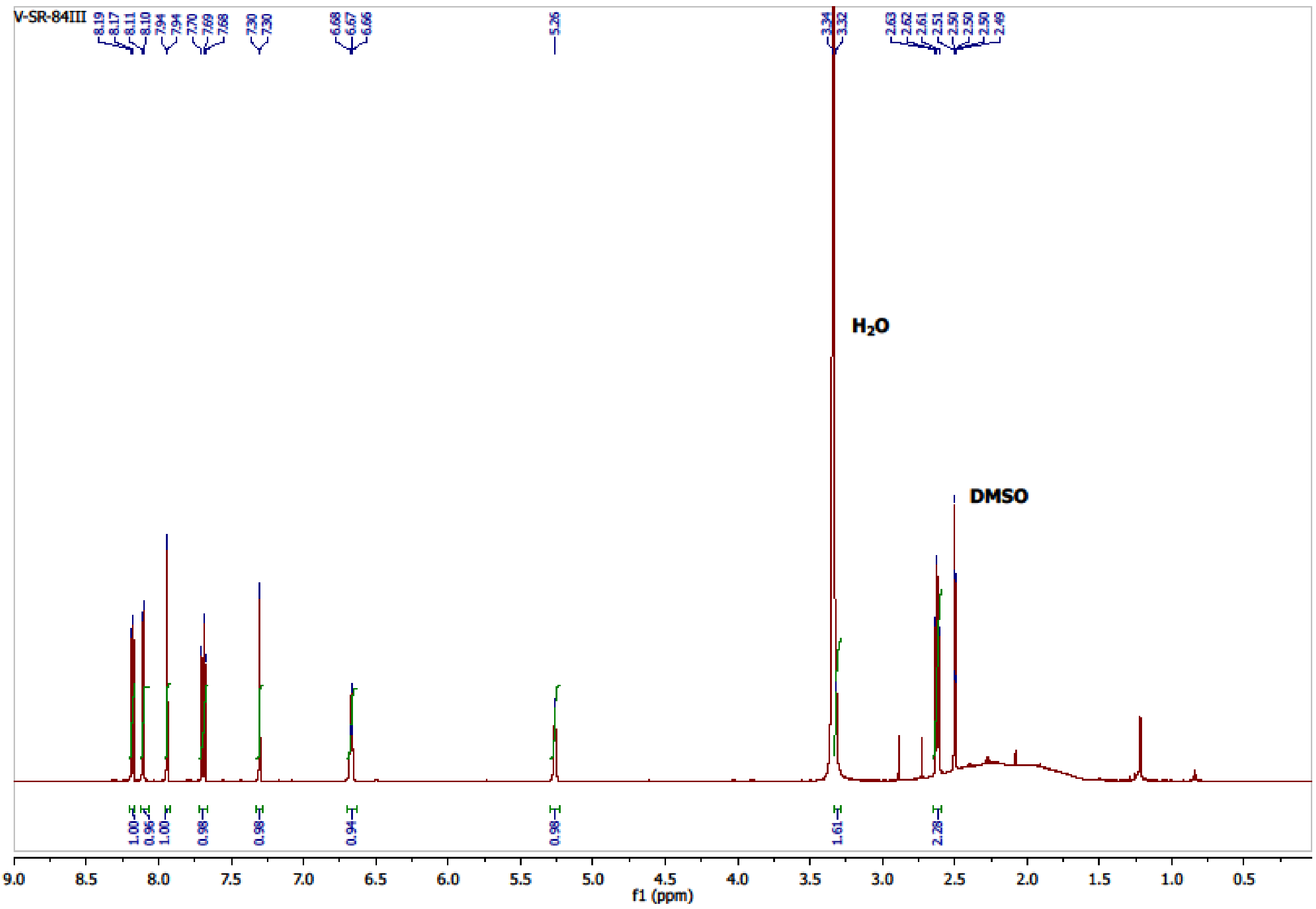


**Figure S87**. $^{1}$H NMR spectrum of **31**.

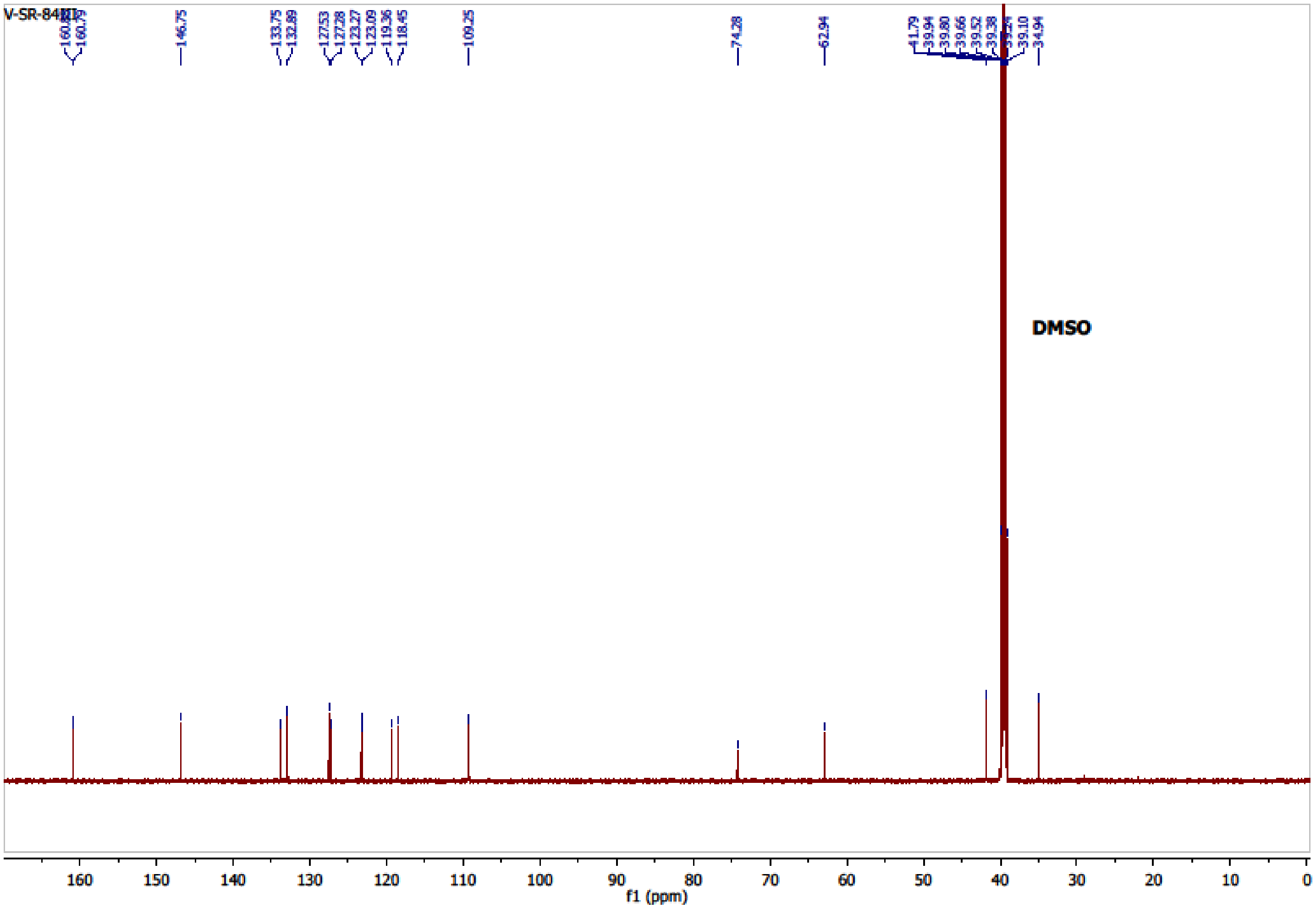


**Figure S88**. $^{13}$C NMR spectrum of **31**.

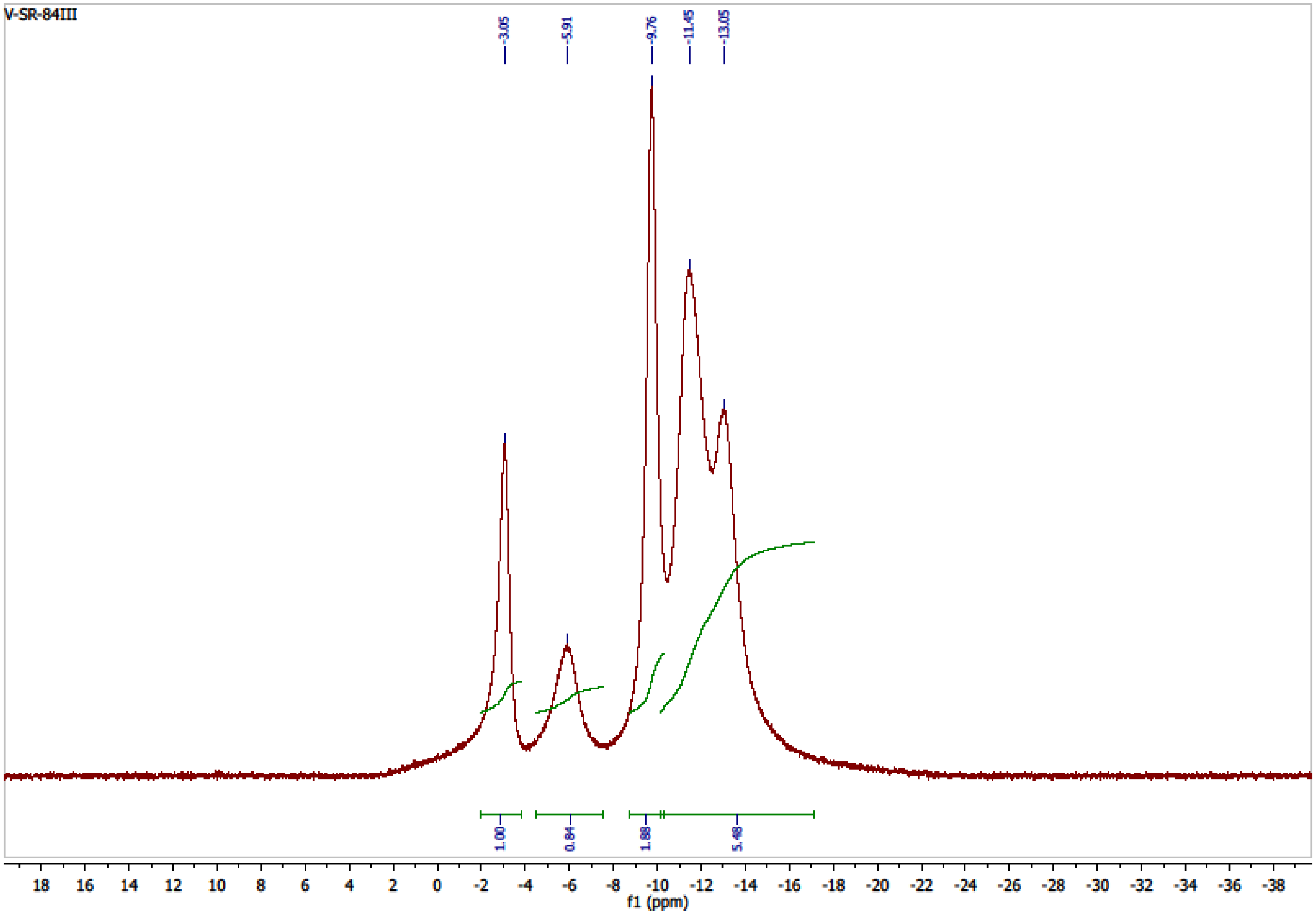


**Figure S89**. $^{11}B$ NMR {$^{1}H$ BB} spectrum of **31**.

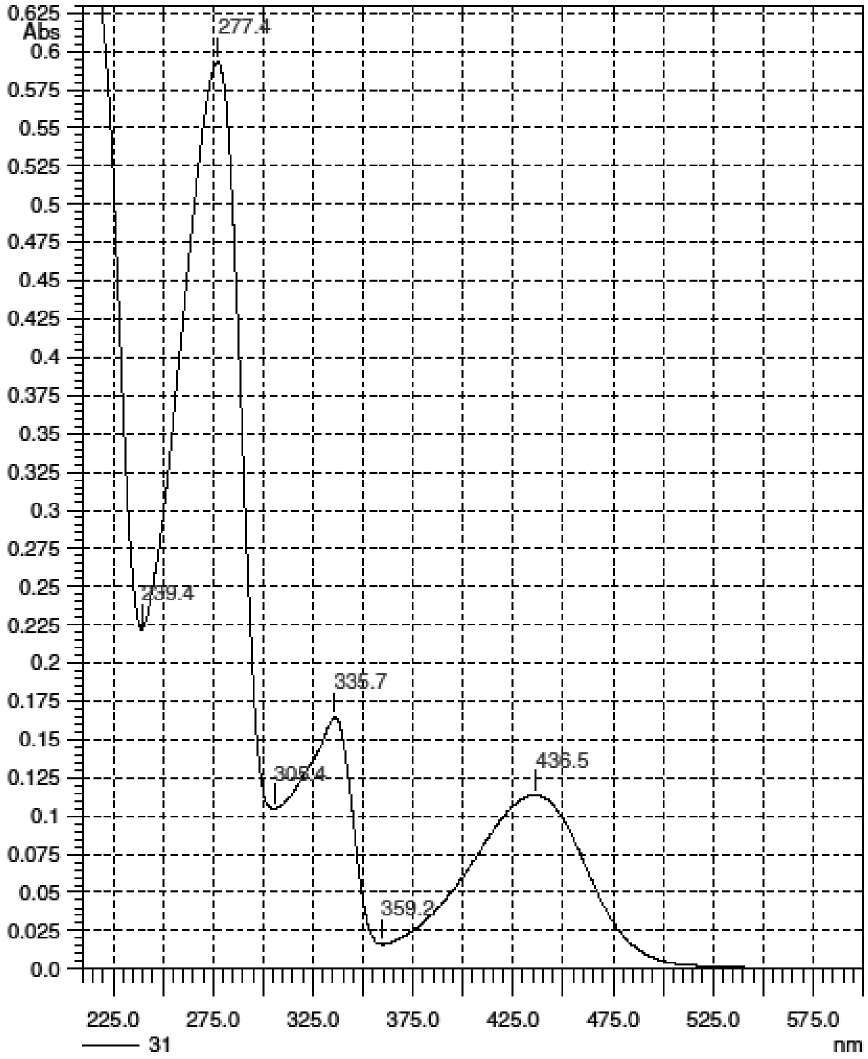


**Figure S90**. UV spectrum of **31**.

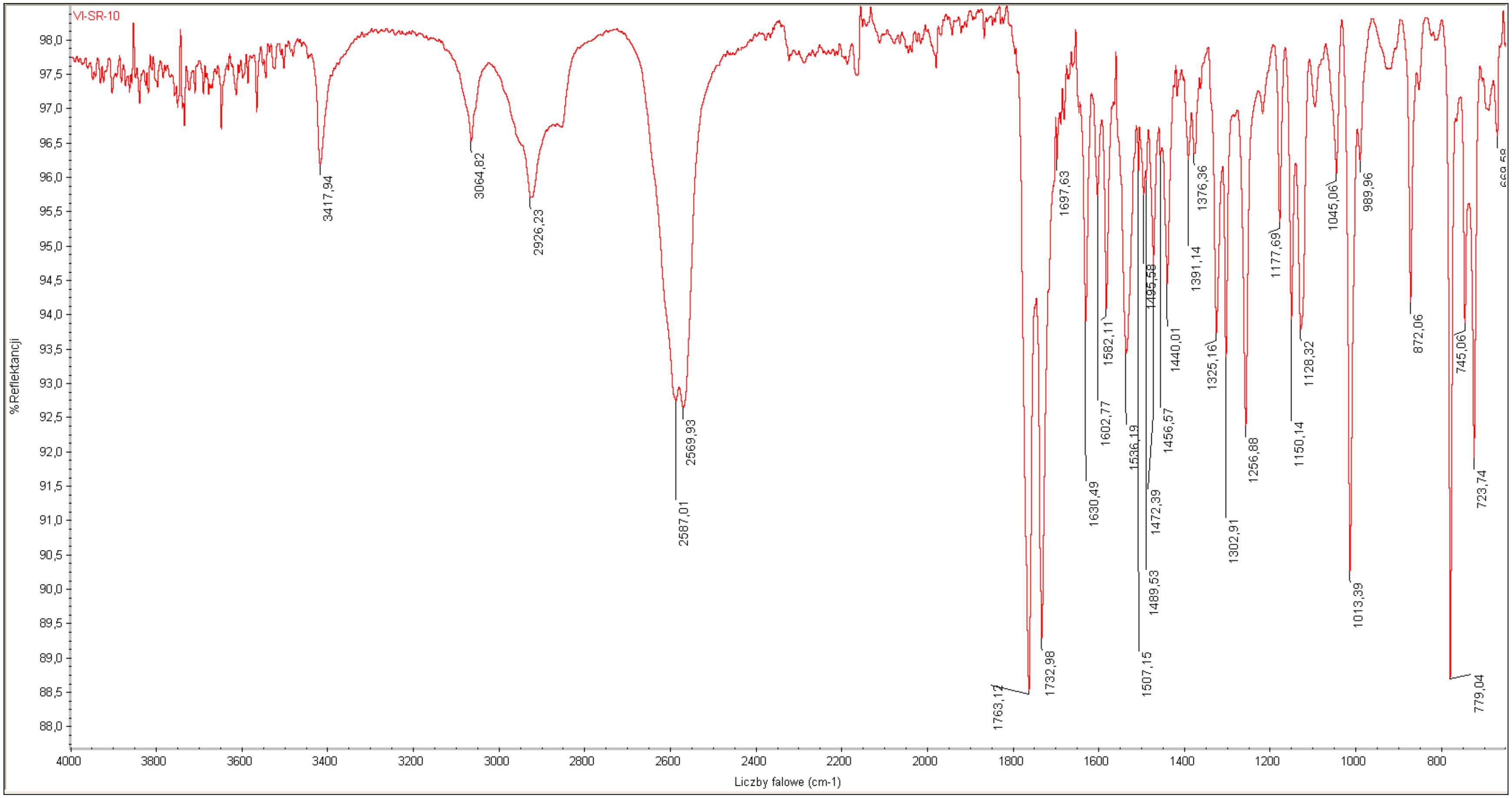


**Figure S91**. IR spectrum of **31**.

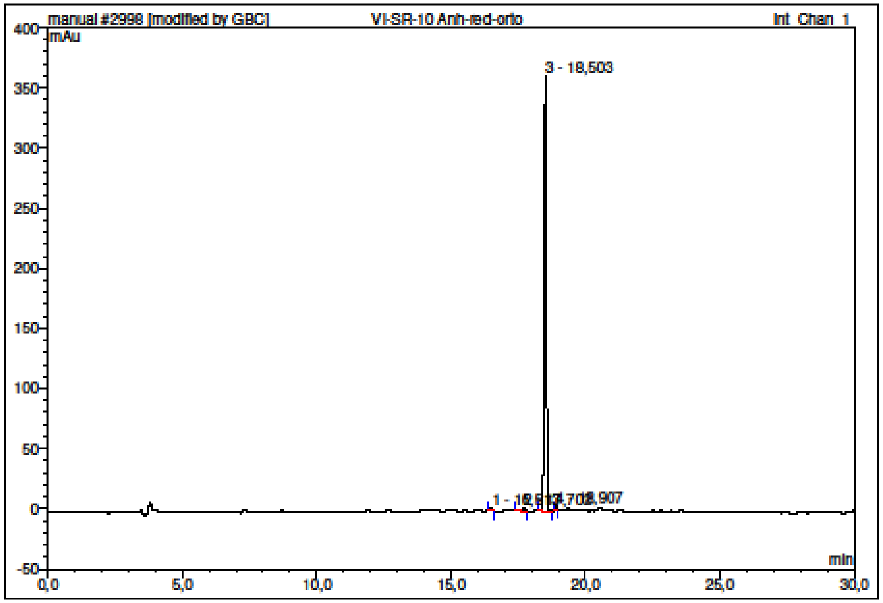


| No. | Ret.Time min | Peak Name | Height mAu | Area mAu*min | Rel.Area % | Amount | Type |
|---|---|---|---|---|---|---|---|
| 1 | 16,51 | n.a. | 1,476 | 0,158 | 0,49 | n.a. | BMB* |
| 2 | 17,70 | n.a. | 1,962 | 0,465 | 1,44 | n.a. | BMB* |
| 3 | 18,50 | n.a. | 361,479 | 31,441 | 97,33 | n.a. | BMB |
| 4 | 18,91 | n.a. | 3,089 | 0,240 | 0,74 | n.a. | BMB* |
| Total: | | | 368,005 | 32,304 | 100,00 | 0,000 | |

**Figure S92**. HPLC analysis of **31**.

Spectrum Name: VI-SR-10_pr
Start Ion: 300
End Ion: 500
Source: APCI + 10.0µA 400C
Capillary: 180V 300C Offset: 30V Span: 20V

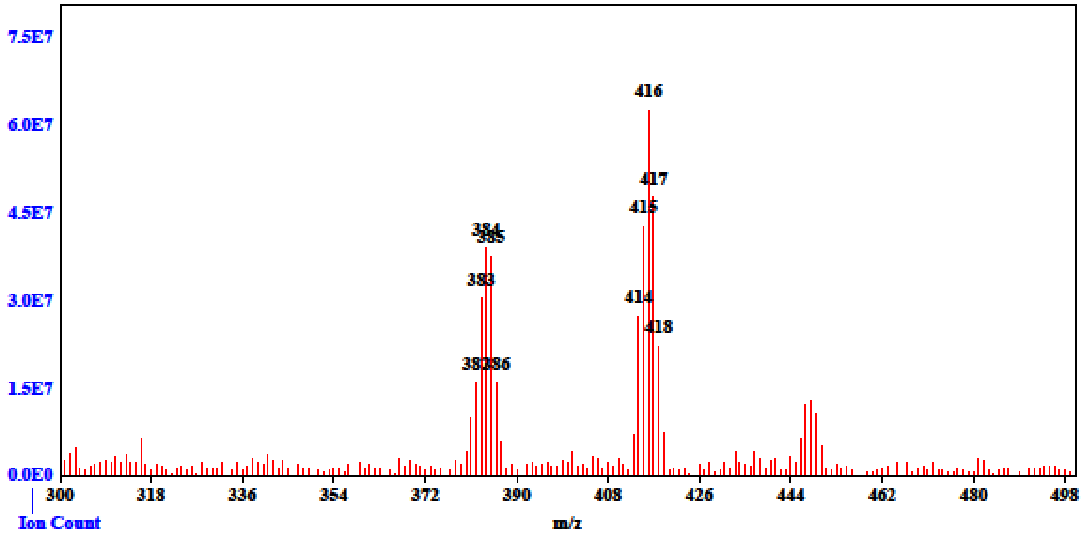


Figure S93. MS spectrum of 31.

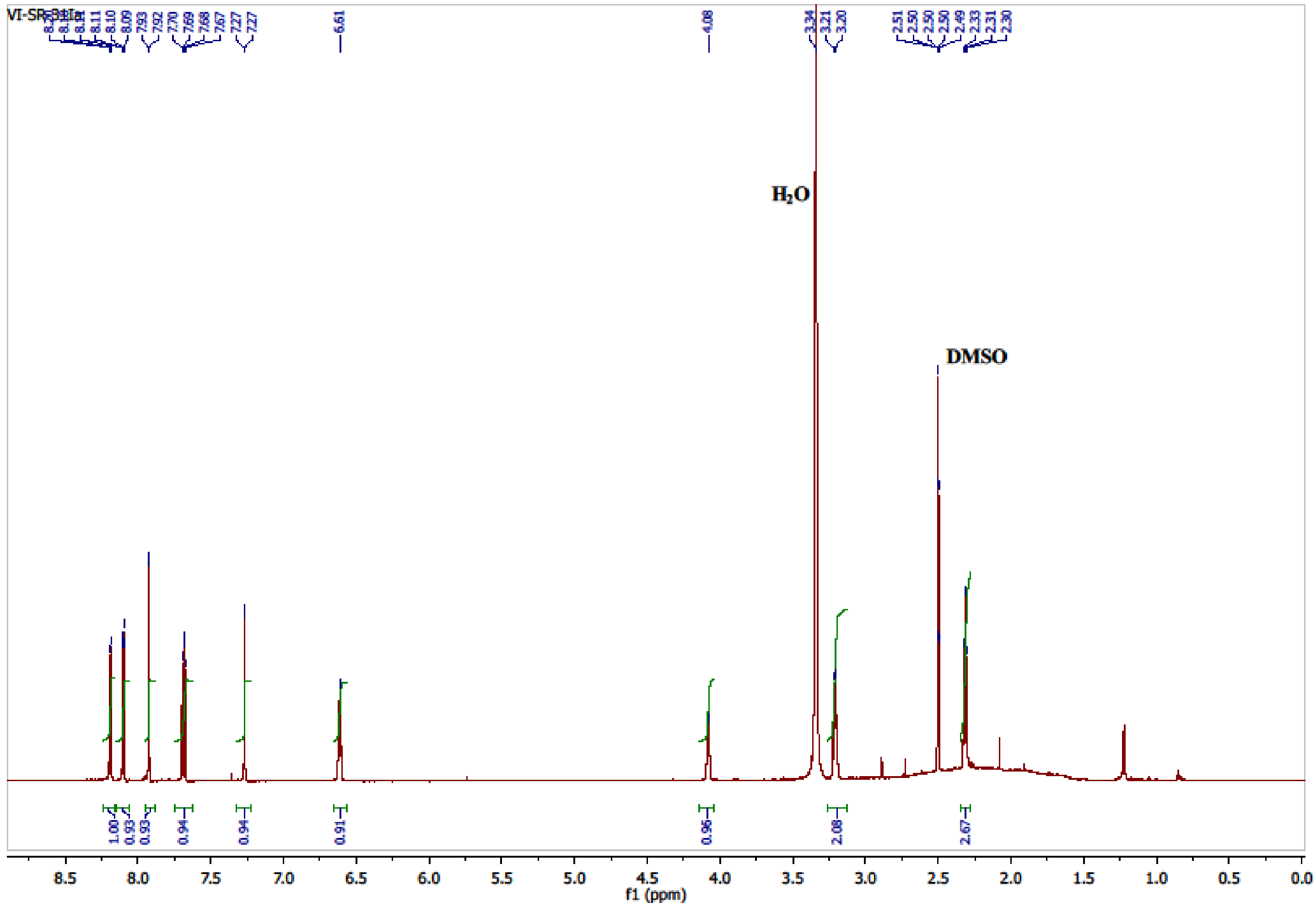


**Figure S94**. $^{1}$H NMR spectrum of **32**.

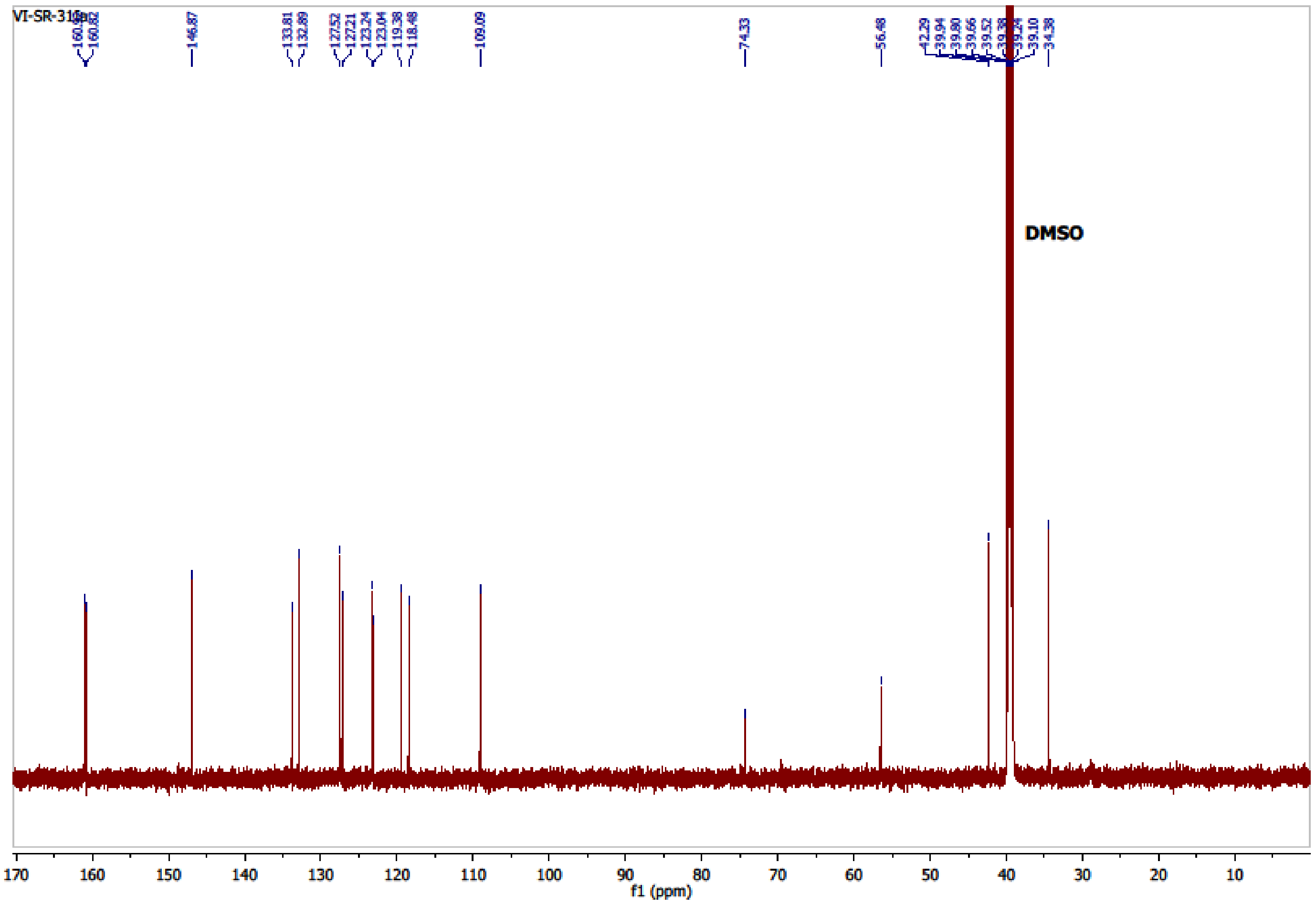


**Figure S95**. $^{13}$C NMR spectrum of **32**.

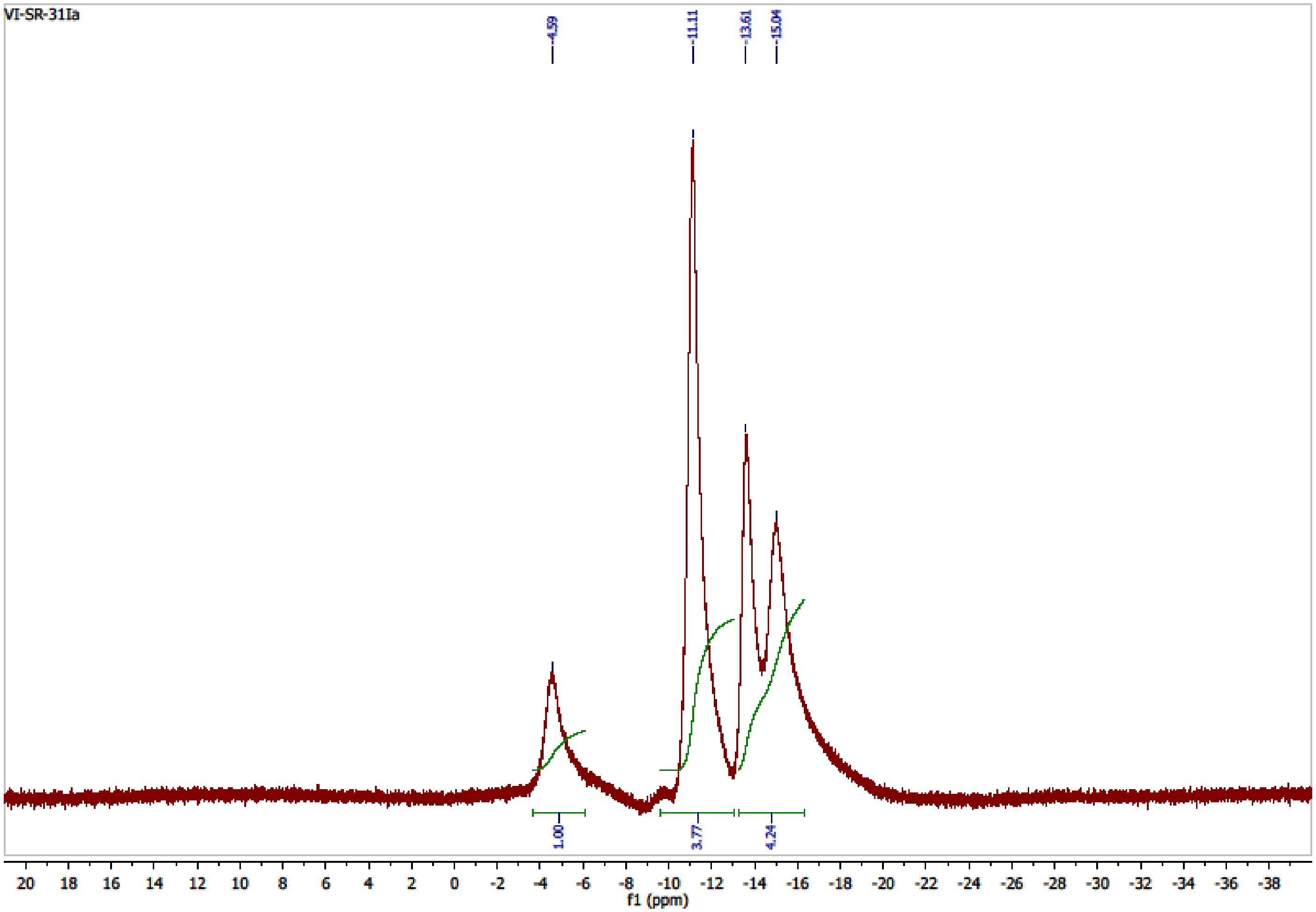


**Figure S96**. $^{11}B$ NMR {$^{1}H$ BB} spectrum of **32**.

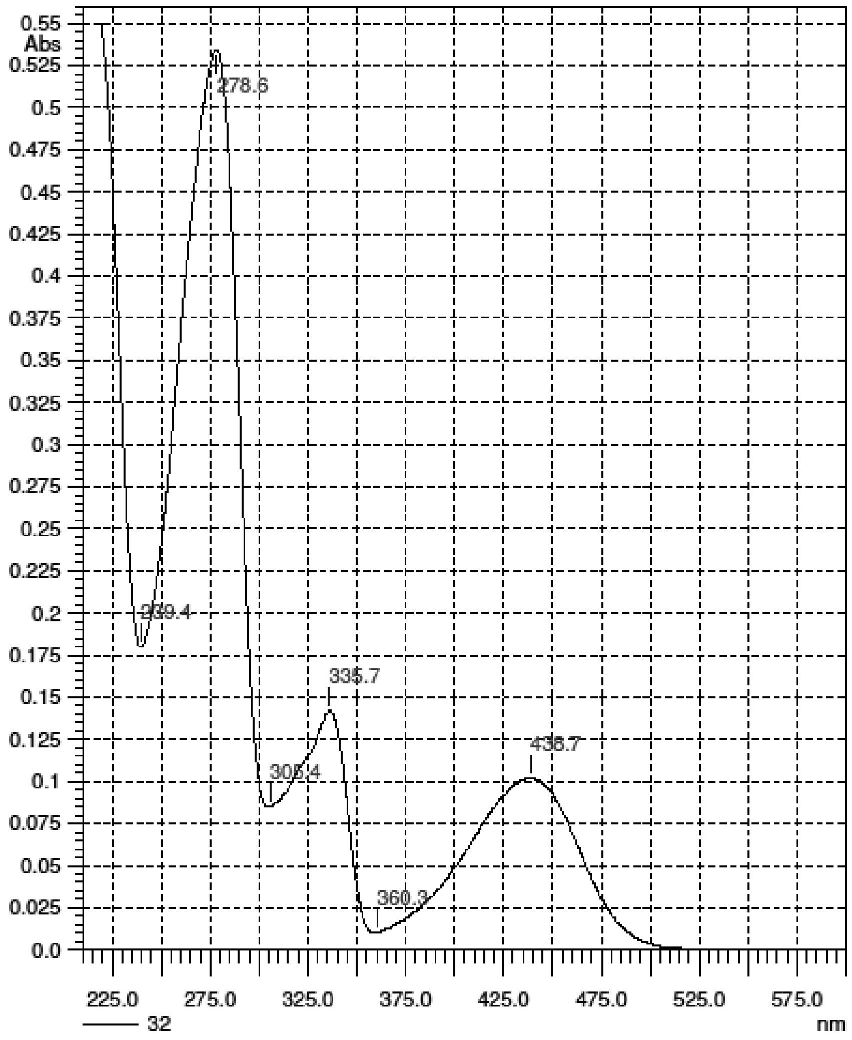


**Figure S97**. UV spectrum of **32**.

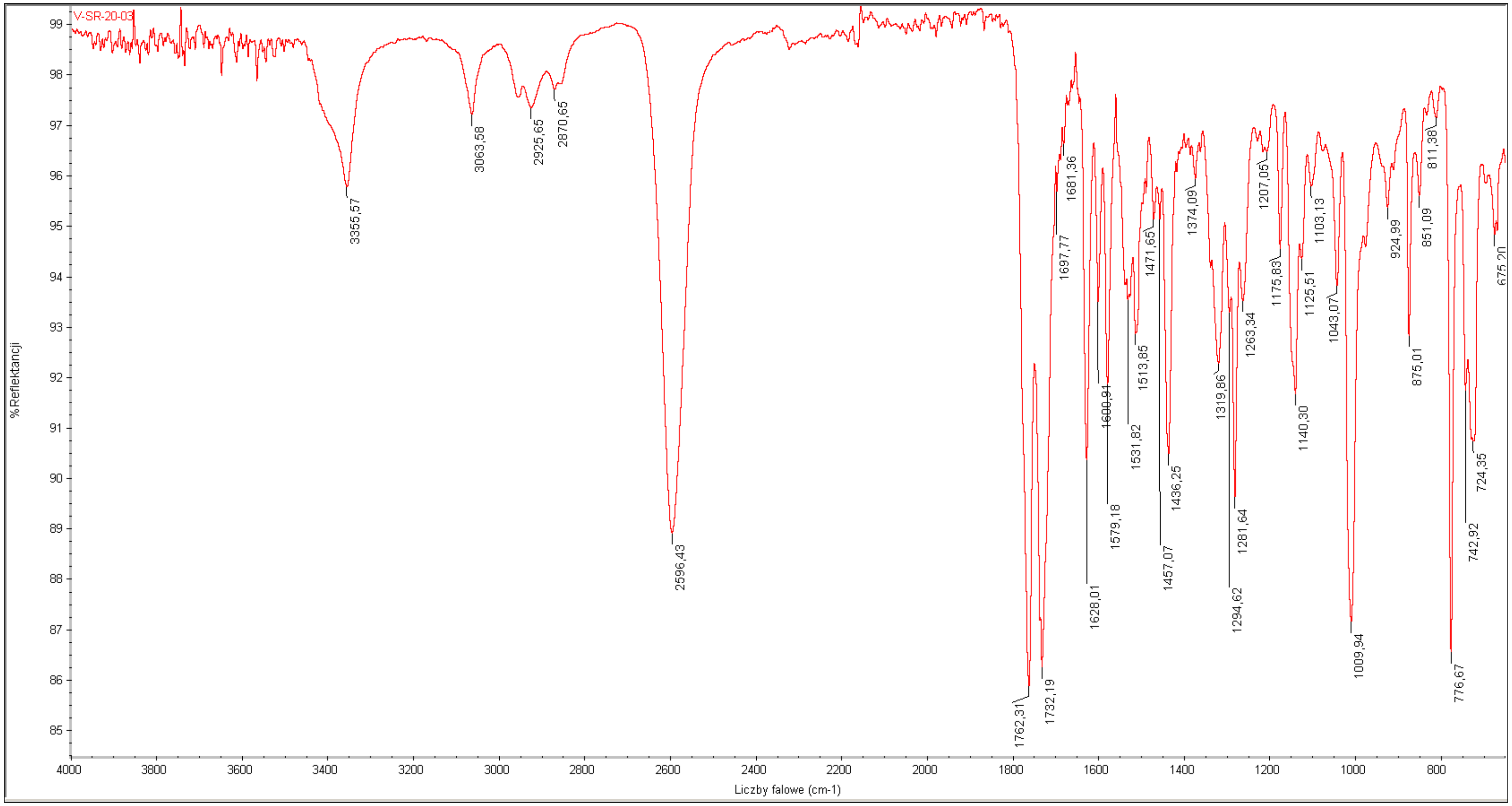


**Figure S98**. IR spectrum of **32**.

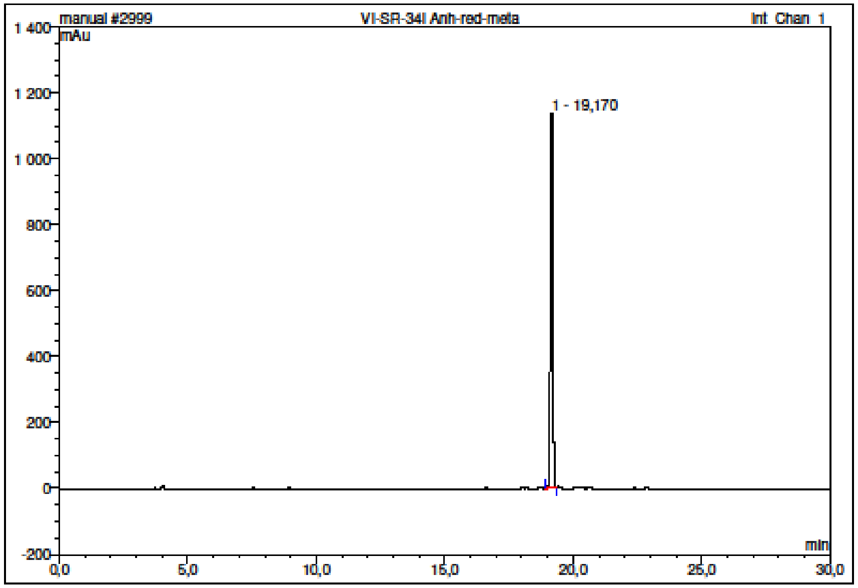


| No. | Ret.Time min | Peak Name | Height mAu | Area mAu*min | Rel.Area % | Amount | Type |
|---|---|---|---|---|---|---|---|
| 1 | 19,17 | n.a. | 1137,513 | 110,542 | 100,00 | n.a. | BMB |
| Total: | | | 1137,513 | 110,542 | 100,00 | 0,000 | |

**Figure S99**. HPLC analysis of **32**.

Spectrum Name: V-SR-20-03_PT
Start Ion: 300
End Ion: 500
Source: APCI + 10.0µA 400C
Capillary: 150V 300C Offset: 25V Span: 0V

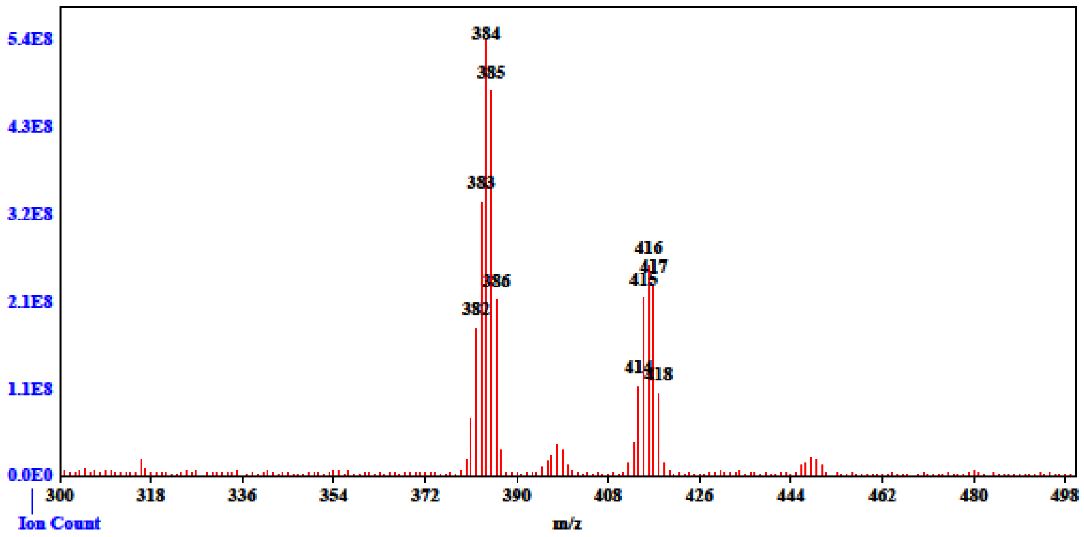


**Figure S100**. MS spectrum of **32**.

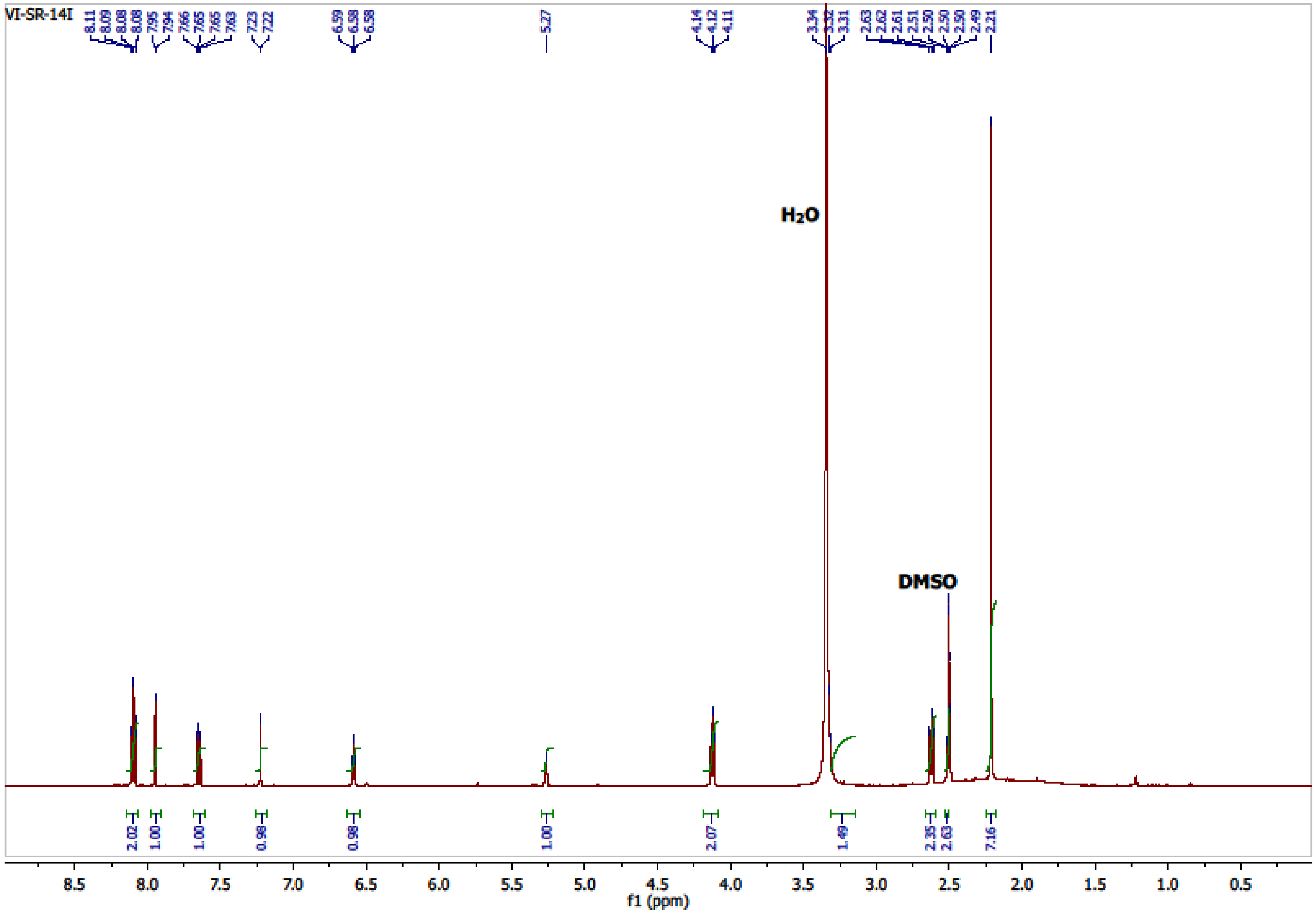


**Figure S101**. $^1$H NMR spectrum of **33**.

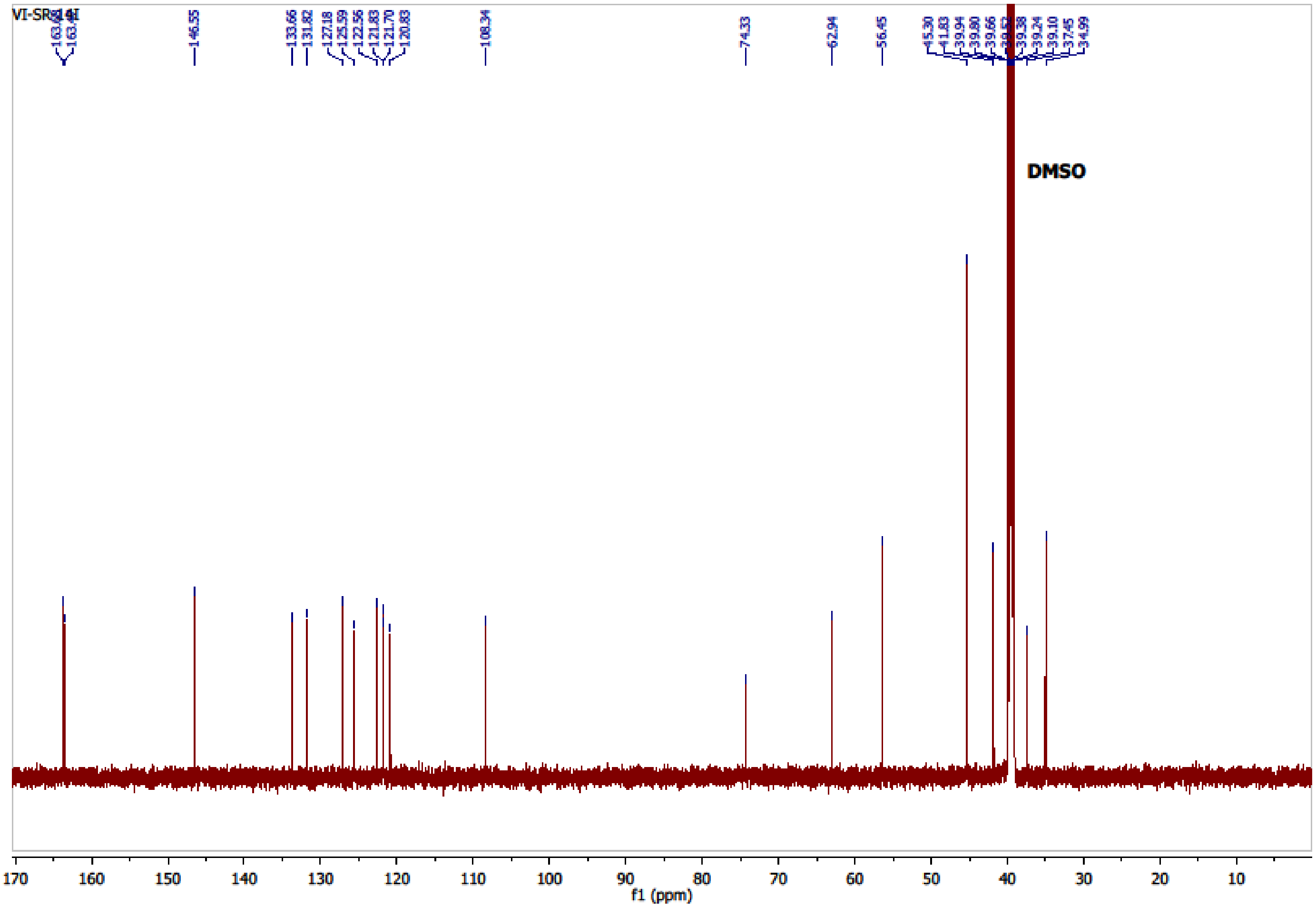


**Figure S102**. $^{13}C$ NMR spectrum of **33**.

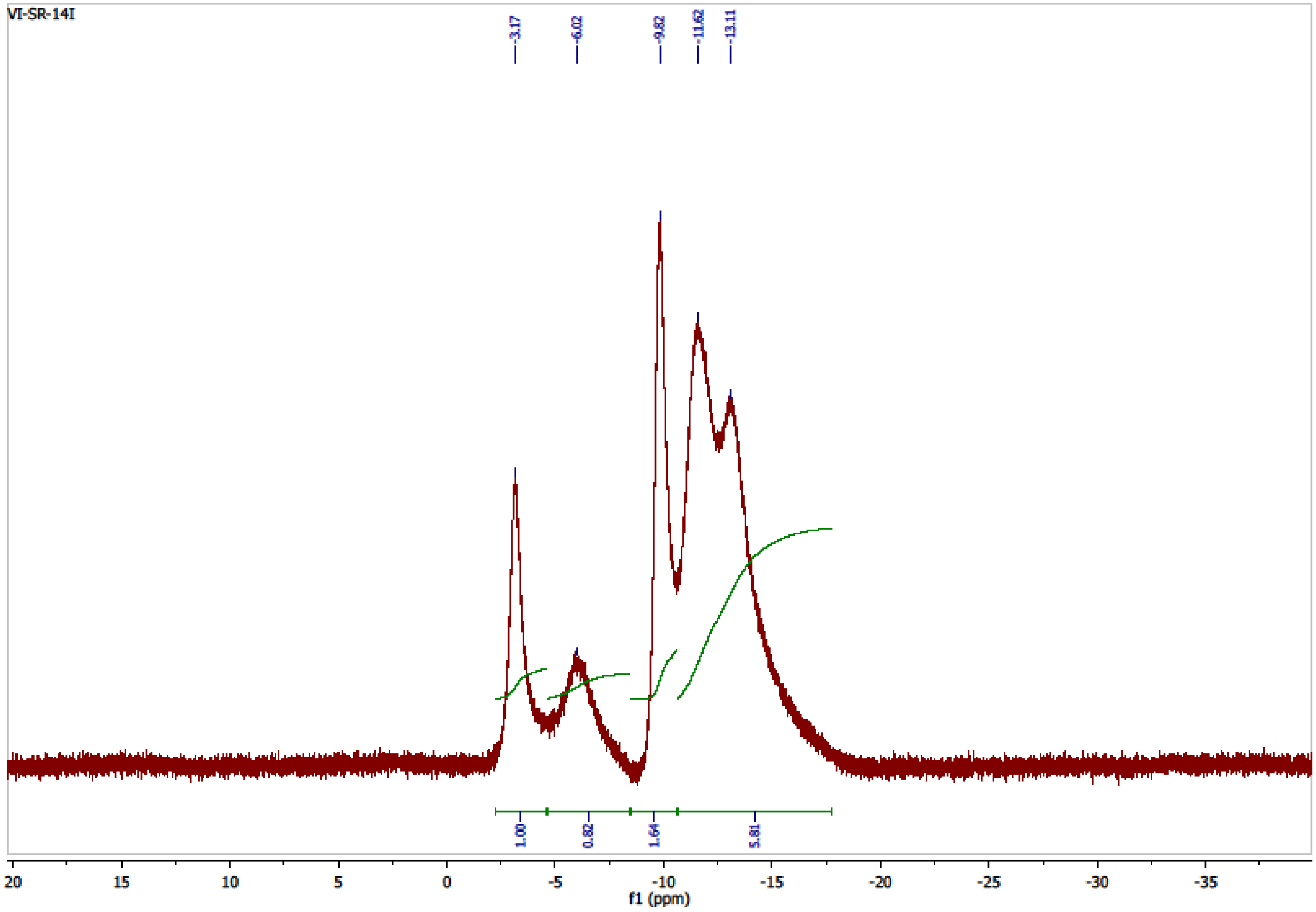


**Figure S103**. $^{11}B$ NMR {$^{1}H$ BB} spectrum of **33**.

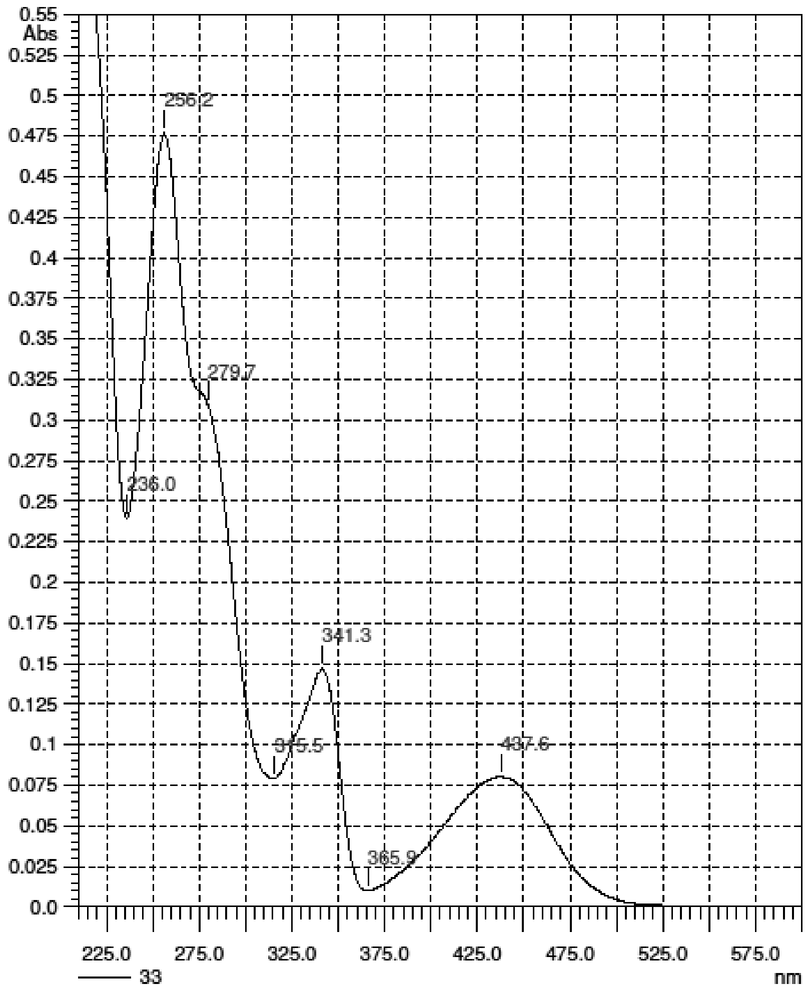


**Figure S104**. UV spectrum of **33**.

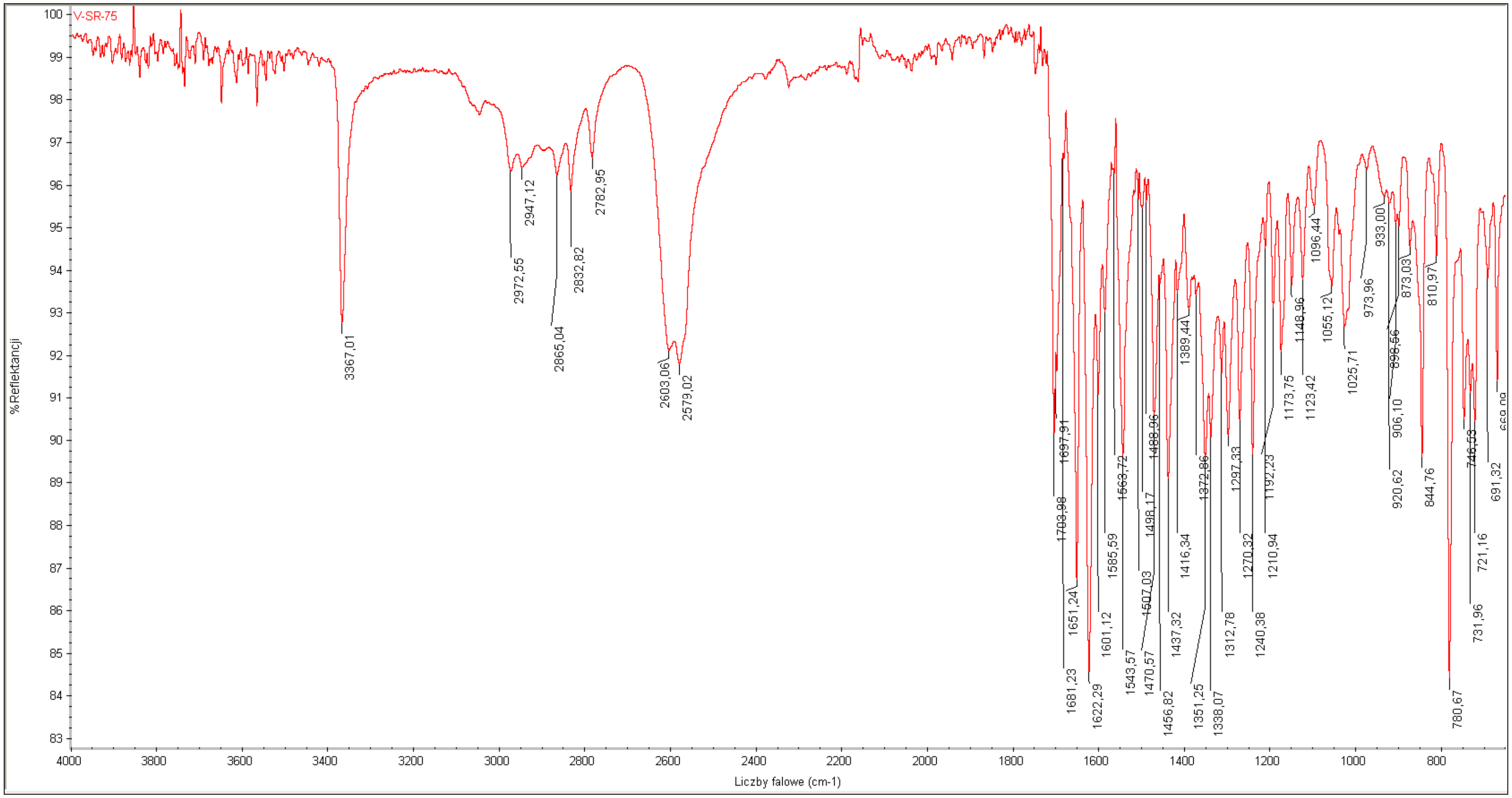


**Figure S105**. IR spectrum of **33**.

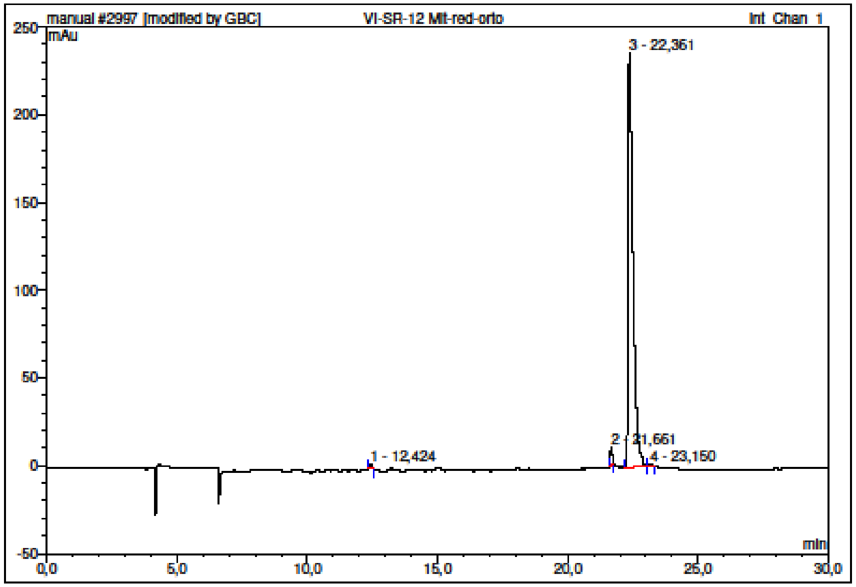


| No. | Ret.Time min | Peak Name | Height mAu | Area mAu*min | Rel.Area % | Amount | Type |
|---|---|---|---|---|---|---|---|
| 1 | 12,42 | n.a. | 2,295 | 0,298 | 0,58 | n.a. | BMB* |
| 2 | 21,66 | n.a. | 10,037 | 0,950 | 1,85 | n.a. | BMB* |
| 3 | 22,36 | n.a. | 235,948 | 49,811 | 97,15 | n.a. | BMB* |
| 4 | 23,15 | n.a. | 1,591 | 0,215 | 0,42 | n.a. | BMB* |
| Total: | | | 249,872 | 51,275 | 100,00 | 0,000 | |

**Figure S106**. HPLC analysis of **33**.

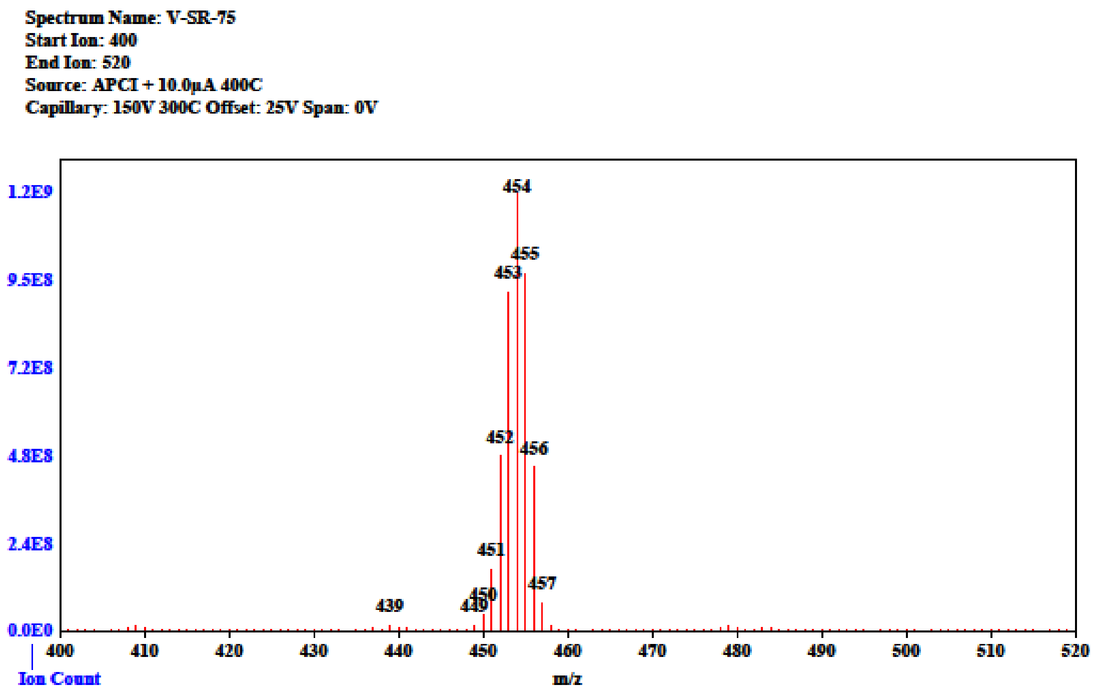


**Figure S107**. MS spectrum of **33**.

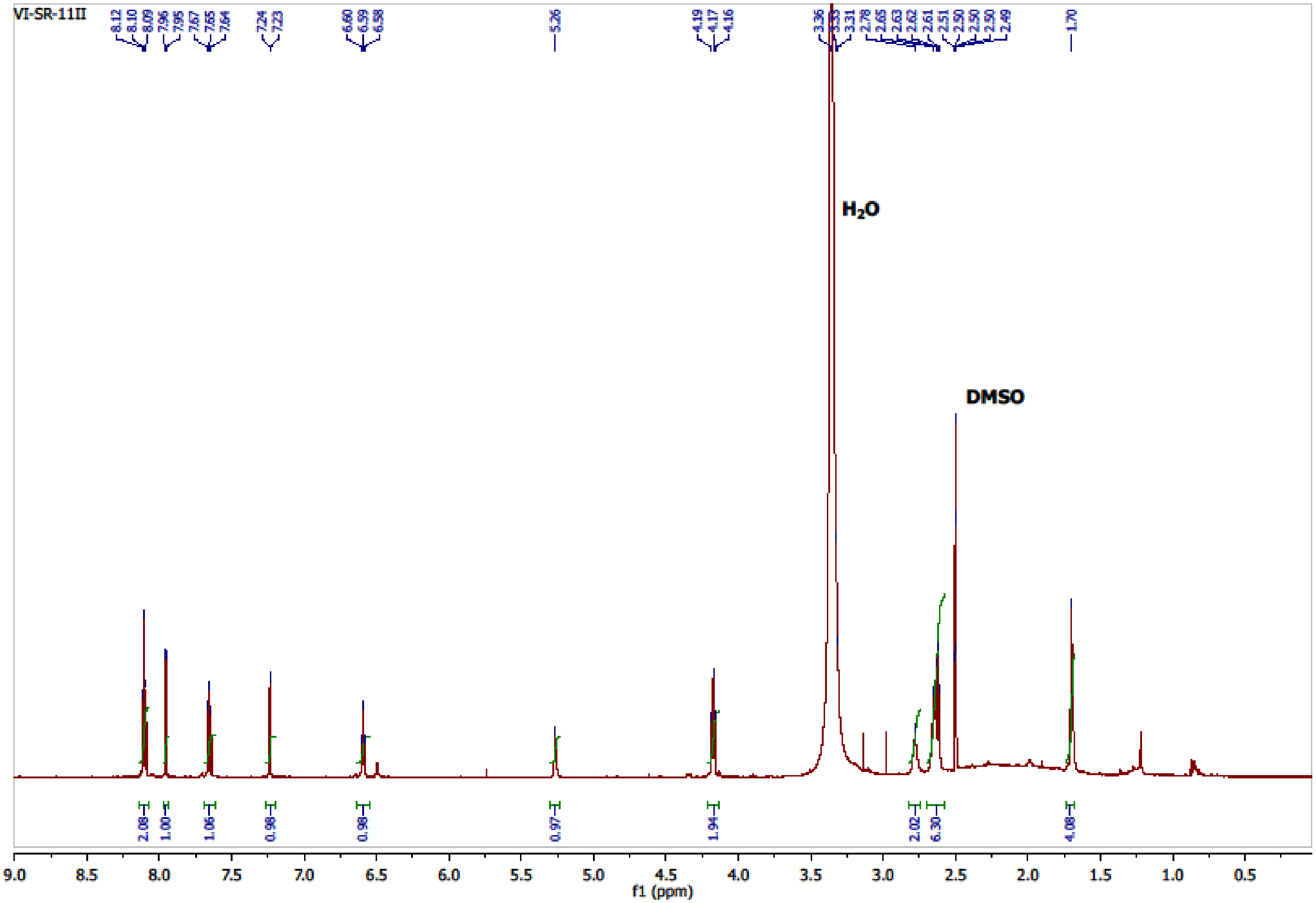


**Figure S108**. $^{1}$H NMR spectrum of **34**.

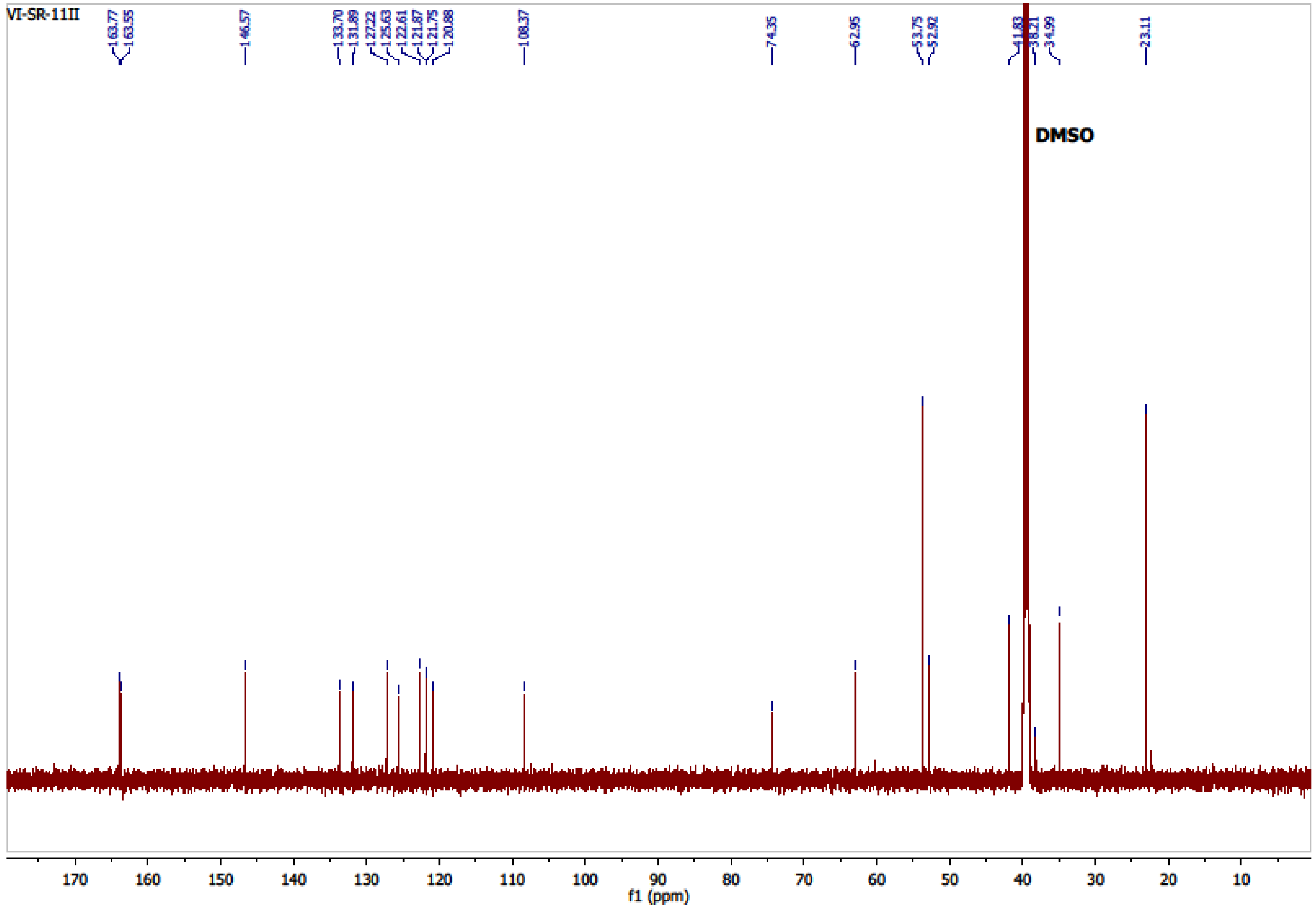


**Figure S109**. $^{13}C$ NMR spectrum of **34**.

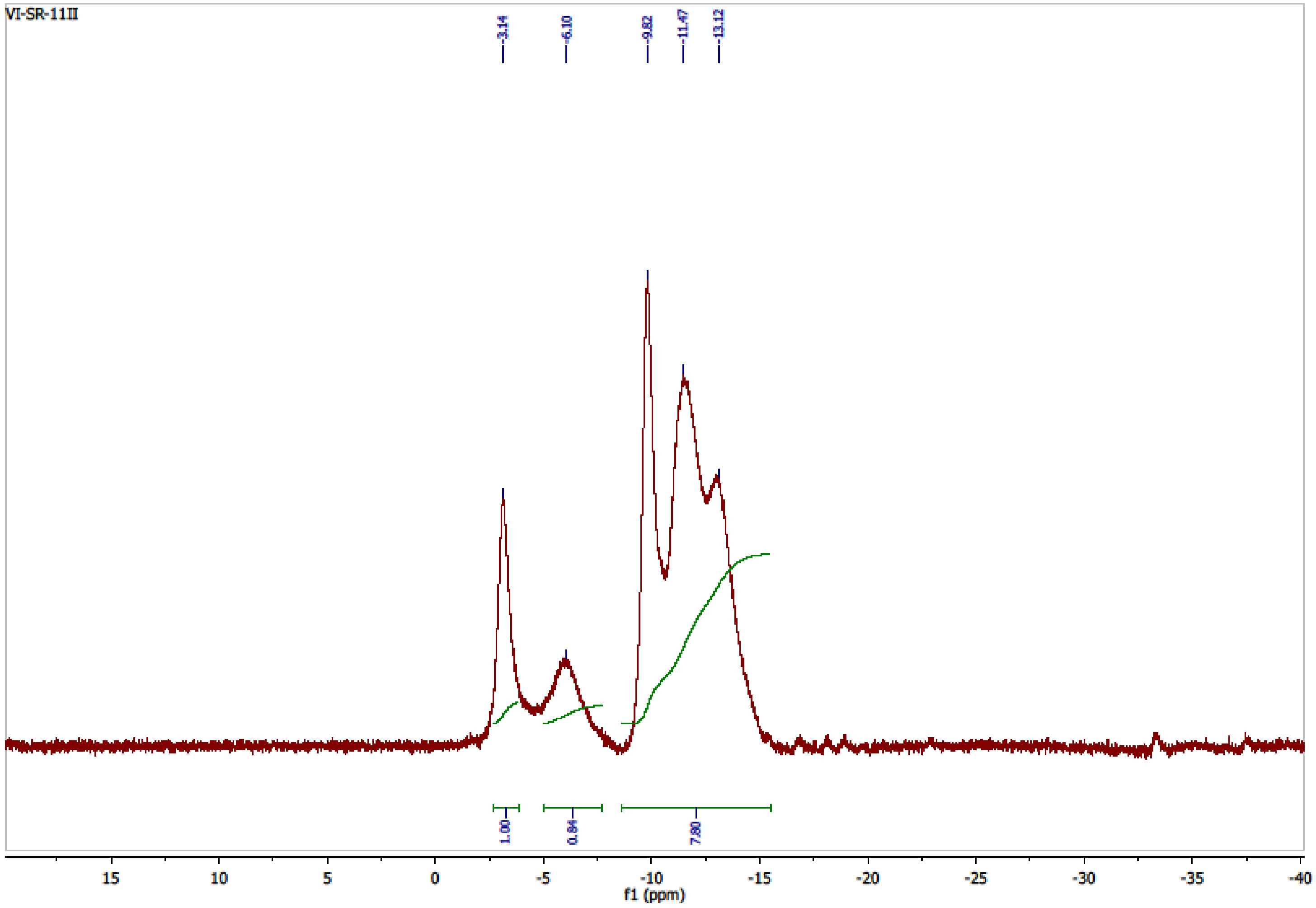


**Figure S110**. $^{11}B$ NMR {$^1H$ BB} spectrum of **34**.

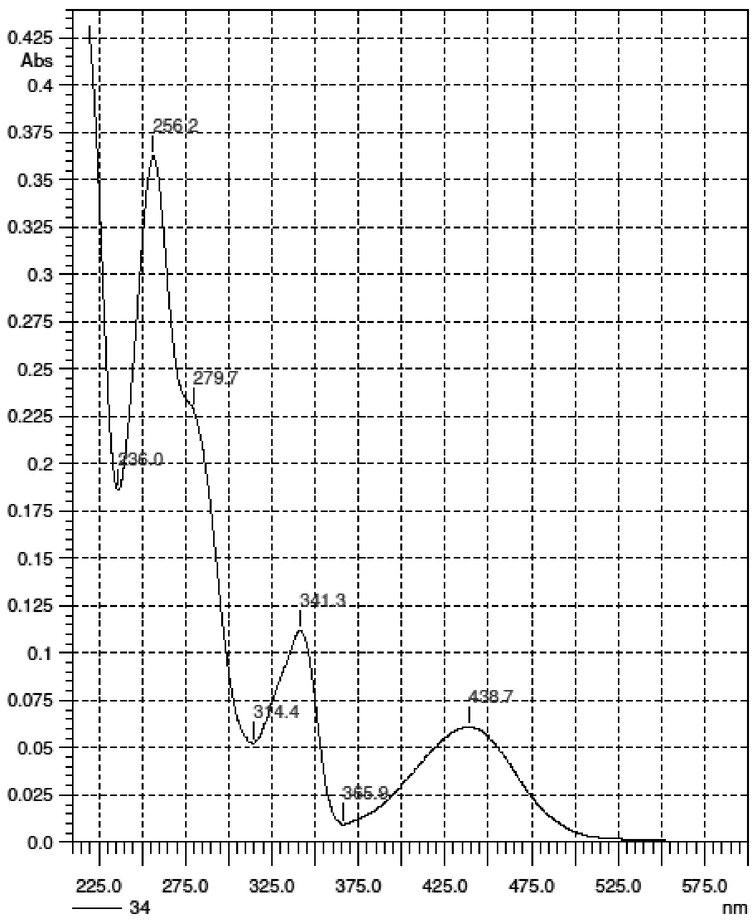


**Figure S111**. UV spectrum of **34**.

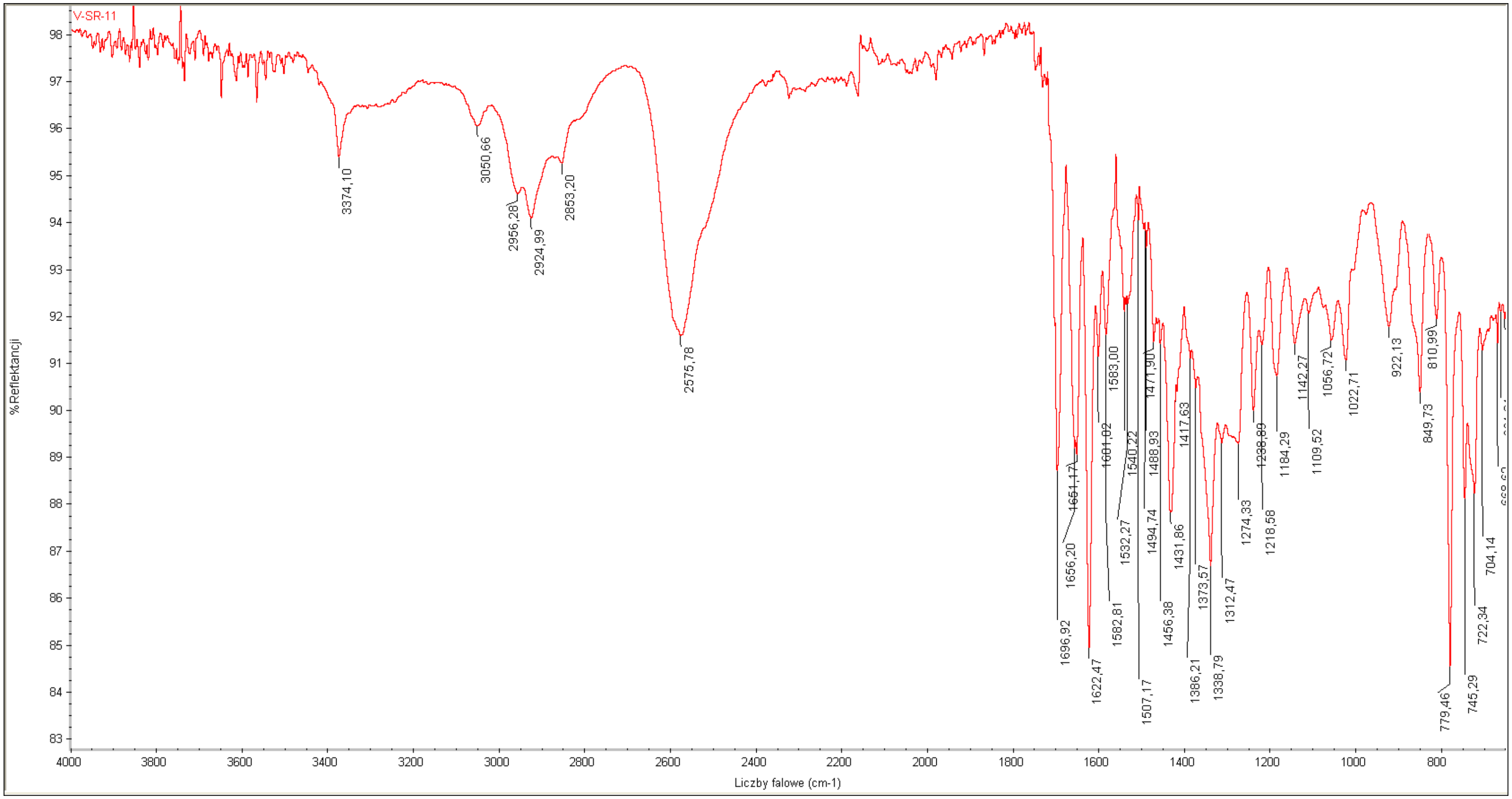


**Figure S112**. IR spectrum of **34**.

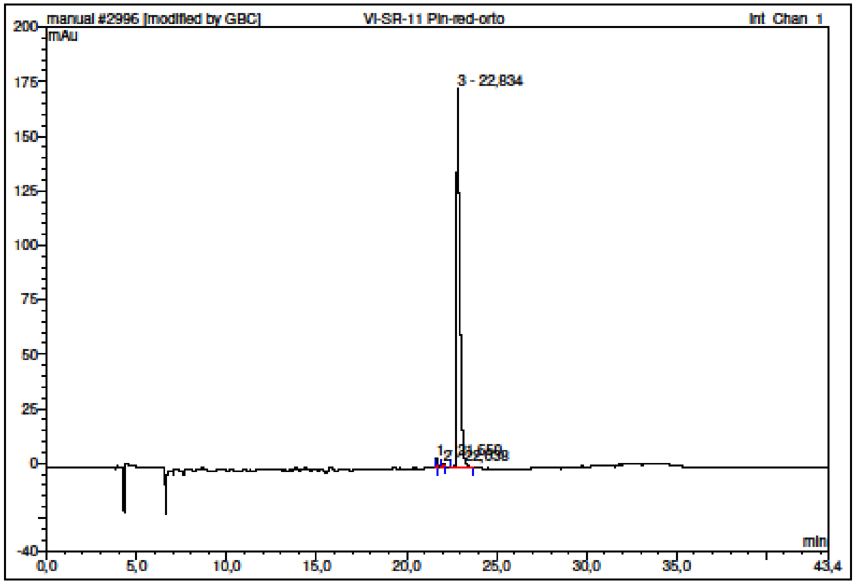


| No. | Ret.Time min | Peak Name | Height mAu | Area mAu*min | Rel.Area % | Amount | Type |
|---|---|---|---|---|---|---|---|
| 1 | 21,65 | n.a. | 3,876 | 0,366 | 1,01 | n.a. | BMB* |
| 2 | 22,04 | n.a. | 1,402 | 0,149 | 0,41 | n.a. | BMB* |
| 3 | 22,83 | n.a. | 173,815 | 35,568 | 98,57 | n.a. | BMB |
| Total: | | | 179,093 | 36,083 | 100,00 | 0,000 | |

**Figure S113**. HPLC analysis of **34**.

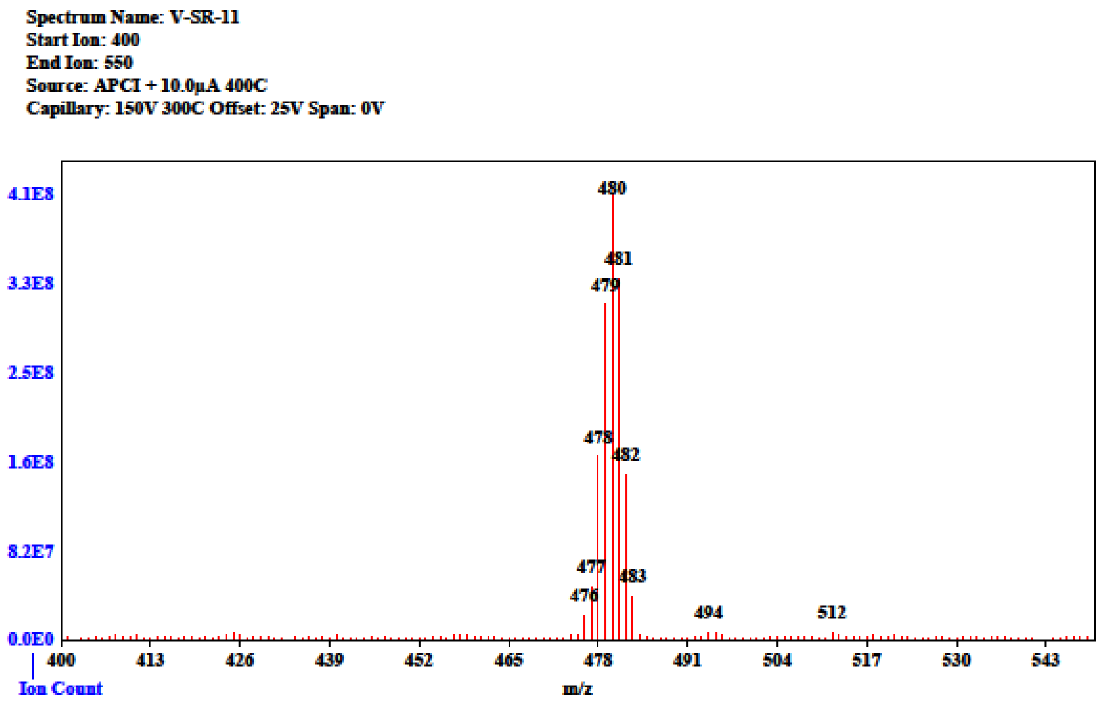


**Figure S114**. MS spectrum of **34**.

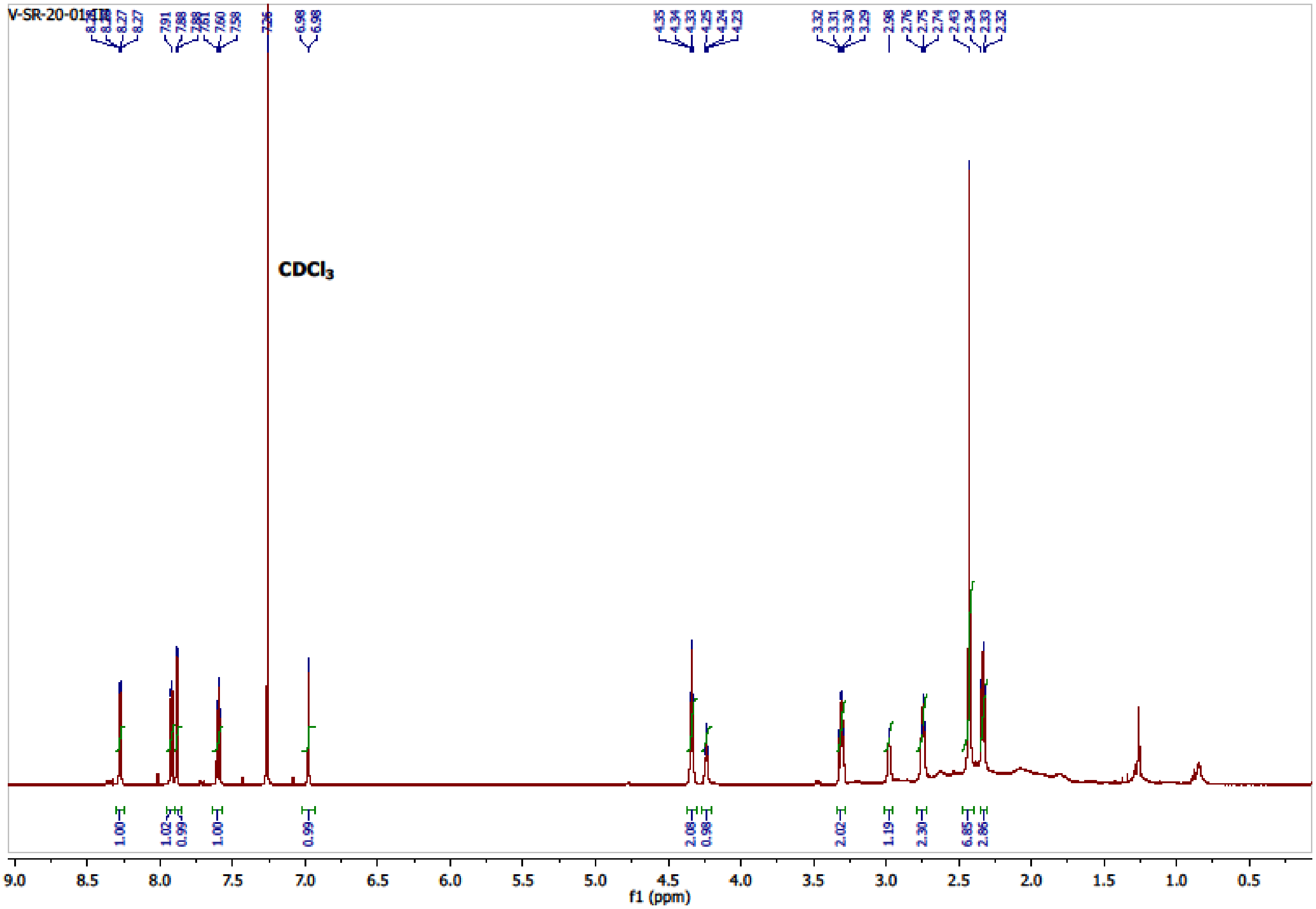


**Figure S115**. $^{1}$H NMR spectrum of **35**.

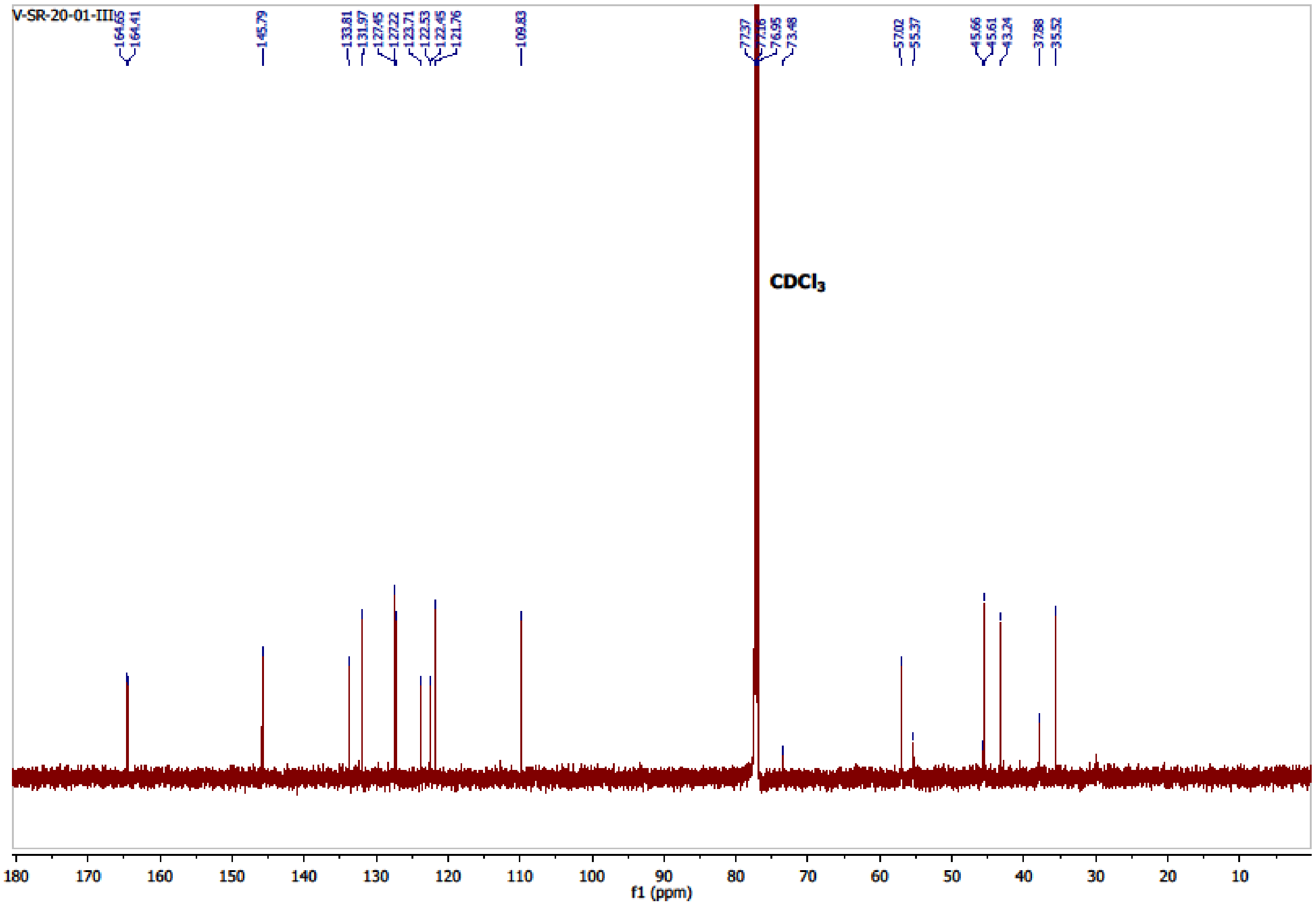


**Figure S116**. $^{13}$C NMR spectrum of **35**.

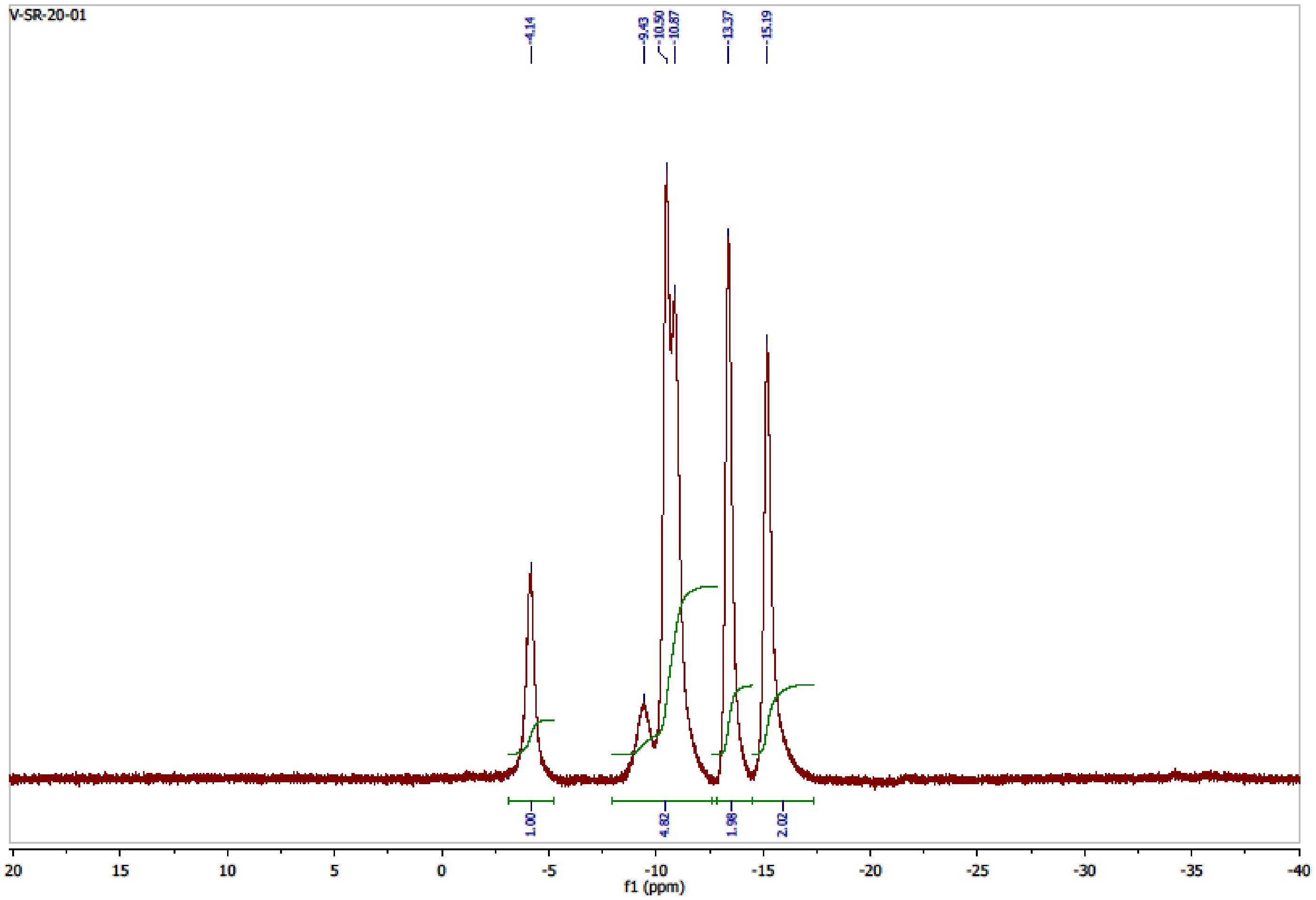


**Figure S117**. $^{11}B$ NMR {$^{1}H$ BB} spectrum of **35**.

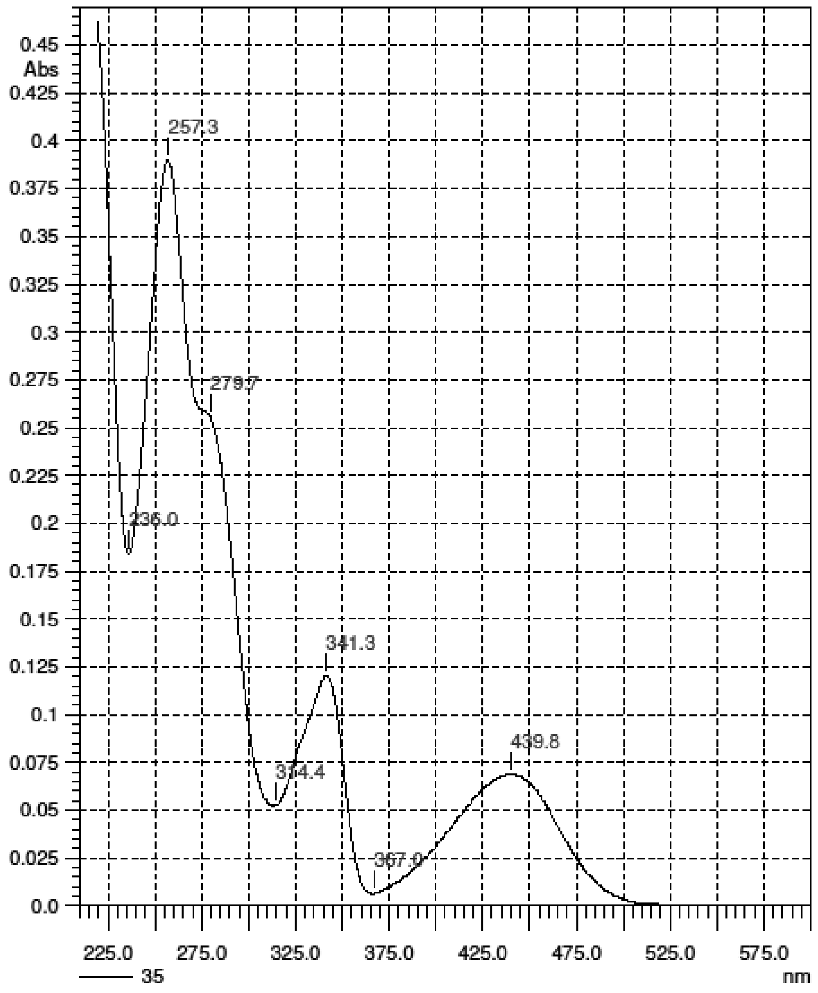


**Figure S118**. UV spectrum of **35**.

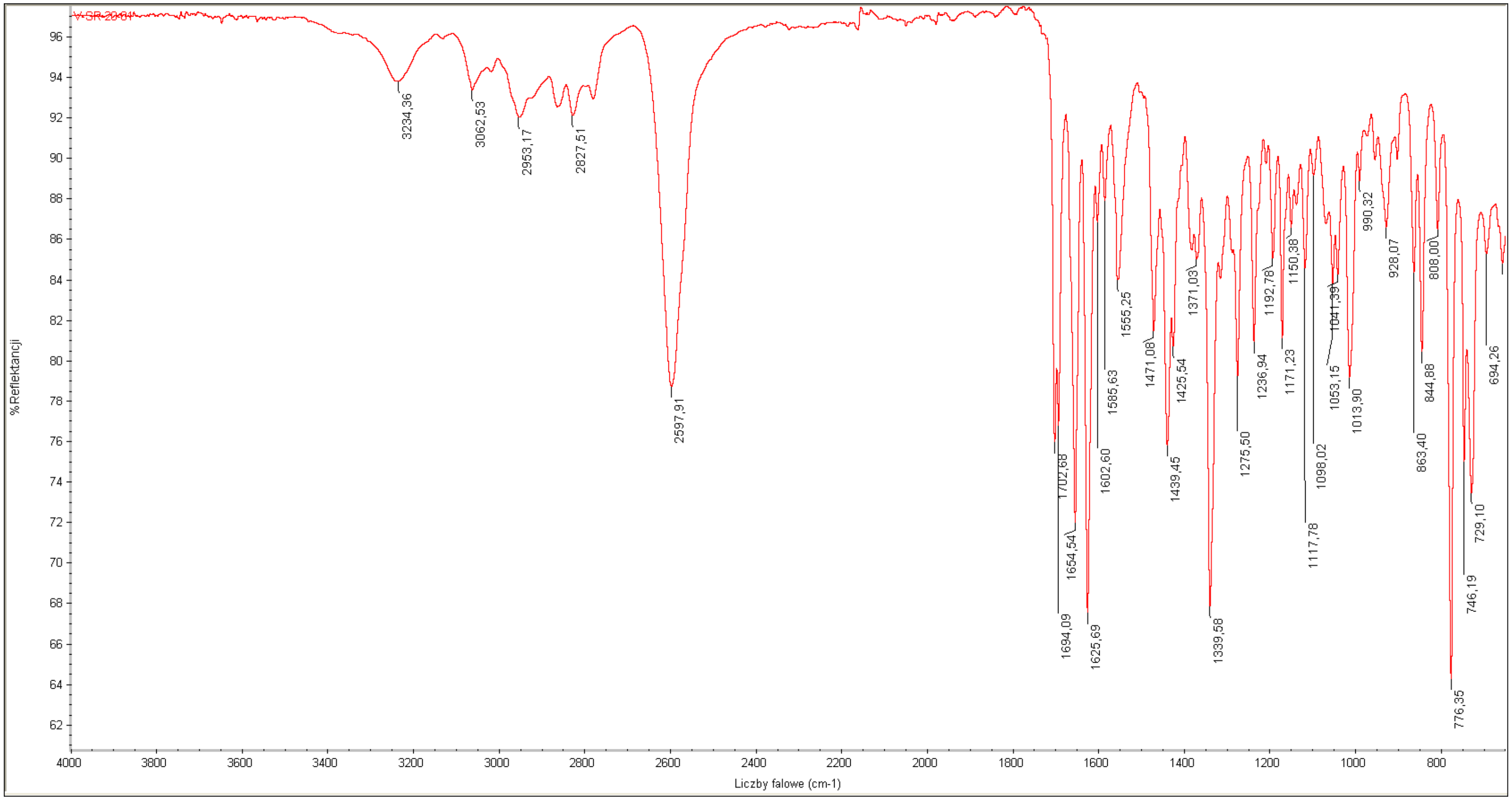


**Figure S119**. IR spectrum of **35**.

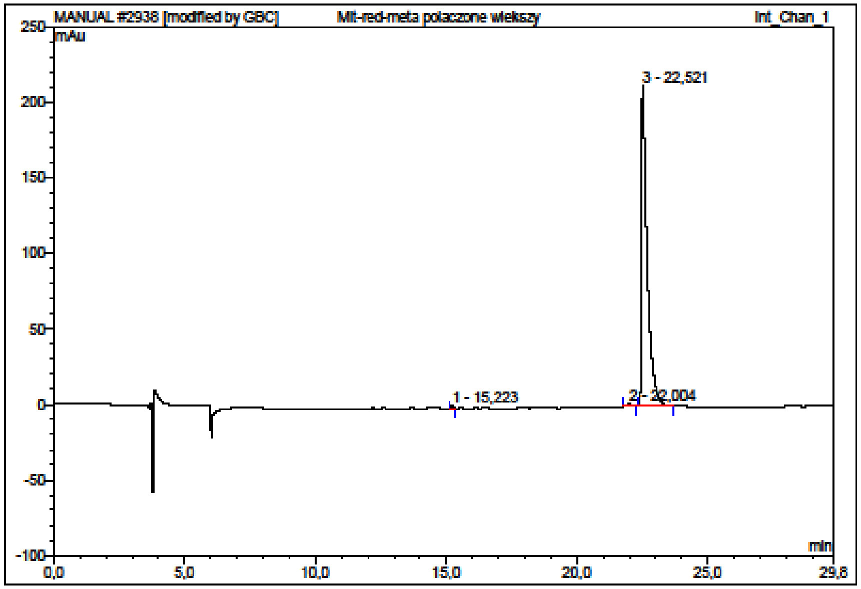


| No. | Ret.Time min | Peak Name | Height mAu | Area mAu*min | Rel.Area % | Amount | Type |
|---|---|---|---|---|---|---|---|
| 1 | 15,22 | n.a. | 1,554 | 0,181 | 0,34 | n.a. | BMB* |
| 2 | 22,00 | n.a. | 1,349 | 0,437 | 0,82 | n.a. | BMB* |
| 3 | 22,52 | n.a. | 211,994 | 52,373 | 98,83 | n.a. | BMB |
| Total: | | | 214,897 | 52,991 | 100,00 | 0,000 | |

**Figure S120**. HPLC analysis of **35**.

Spectrum Name: V-SR-20-01
Start Ion: 350
End Ion: 550
Source: APCI + 10.0µA 400C
Capillary: 150V 300C Offset: 25V Span: 0V

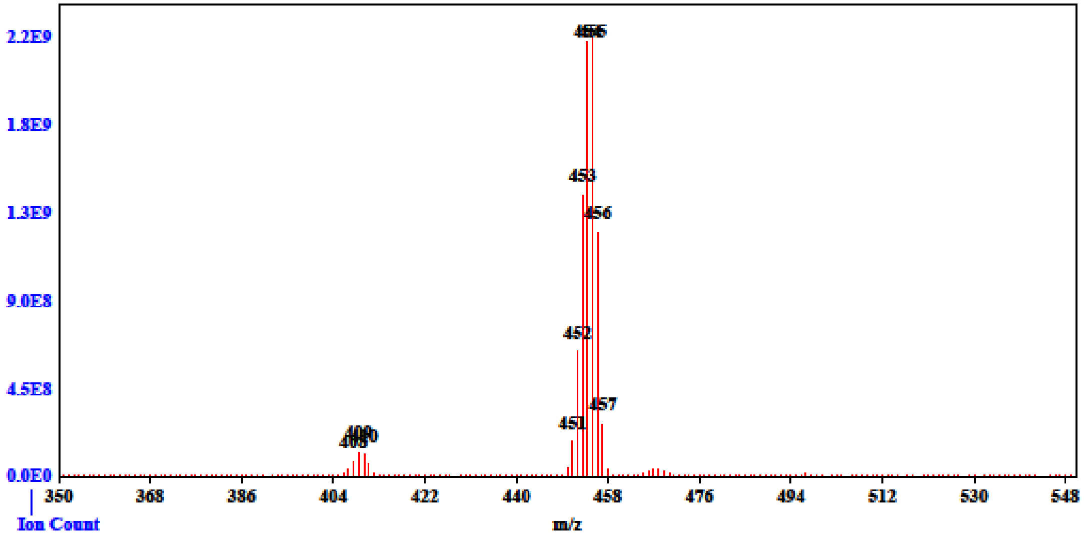


**Figure S121**. MS spectrum of **35**.

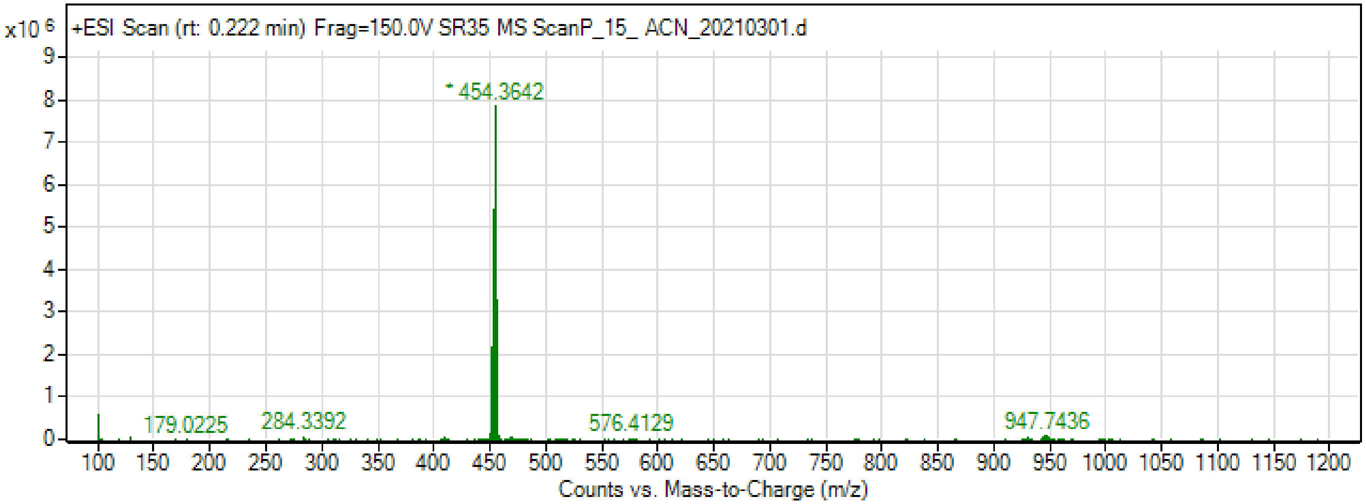


**Figure S122**. HRMS spectrum of **35**.

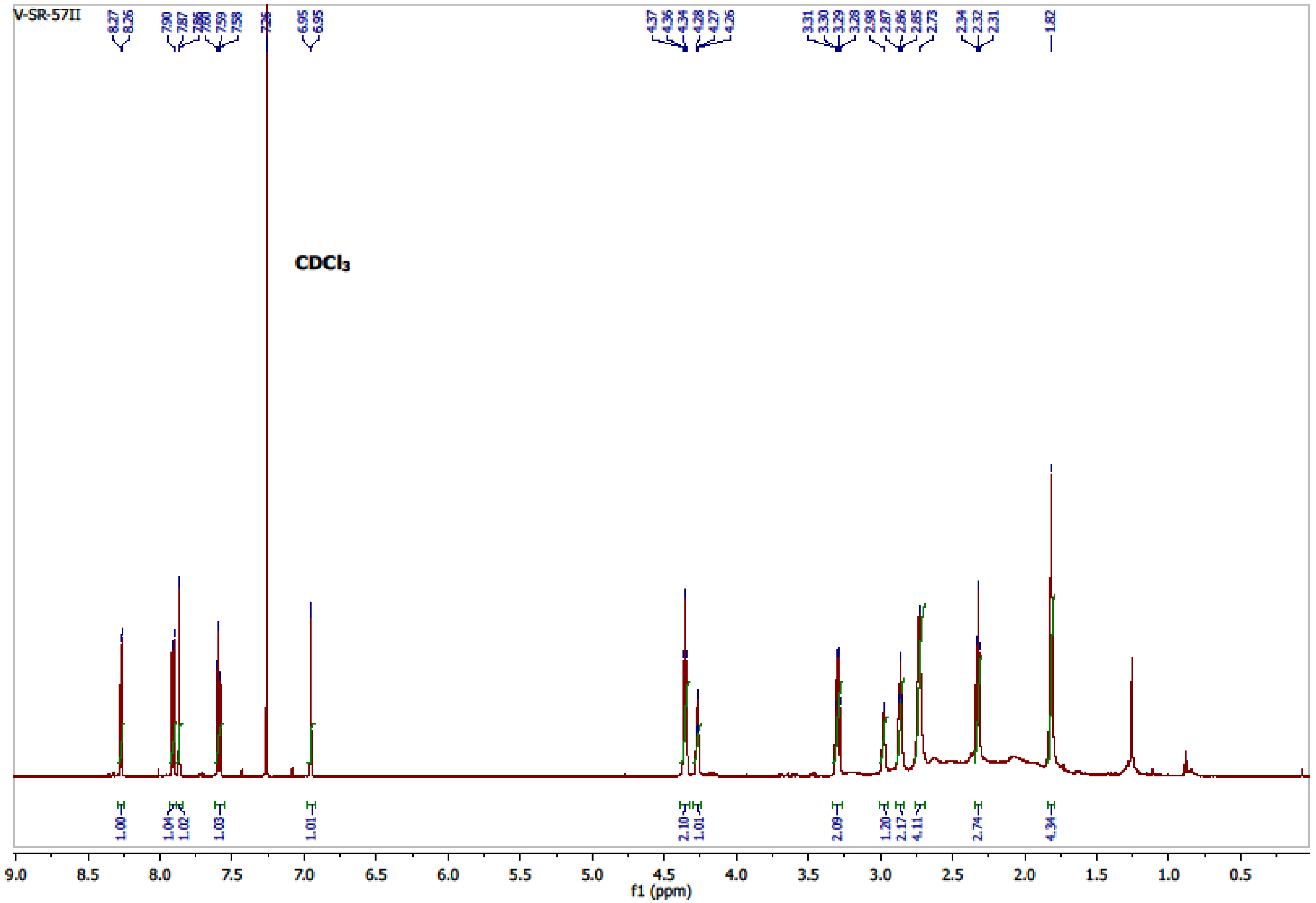


**Figure S123**. $^{1}$H NMR spectrum of **36**.

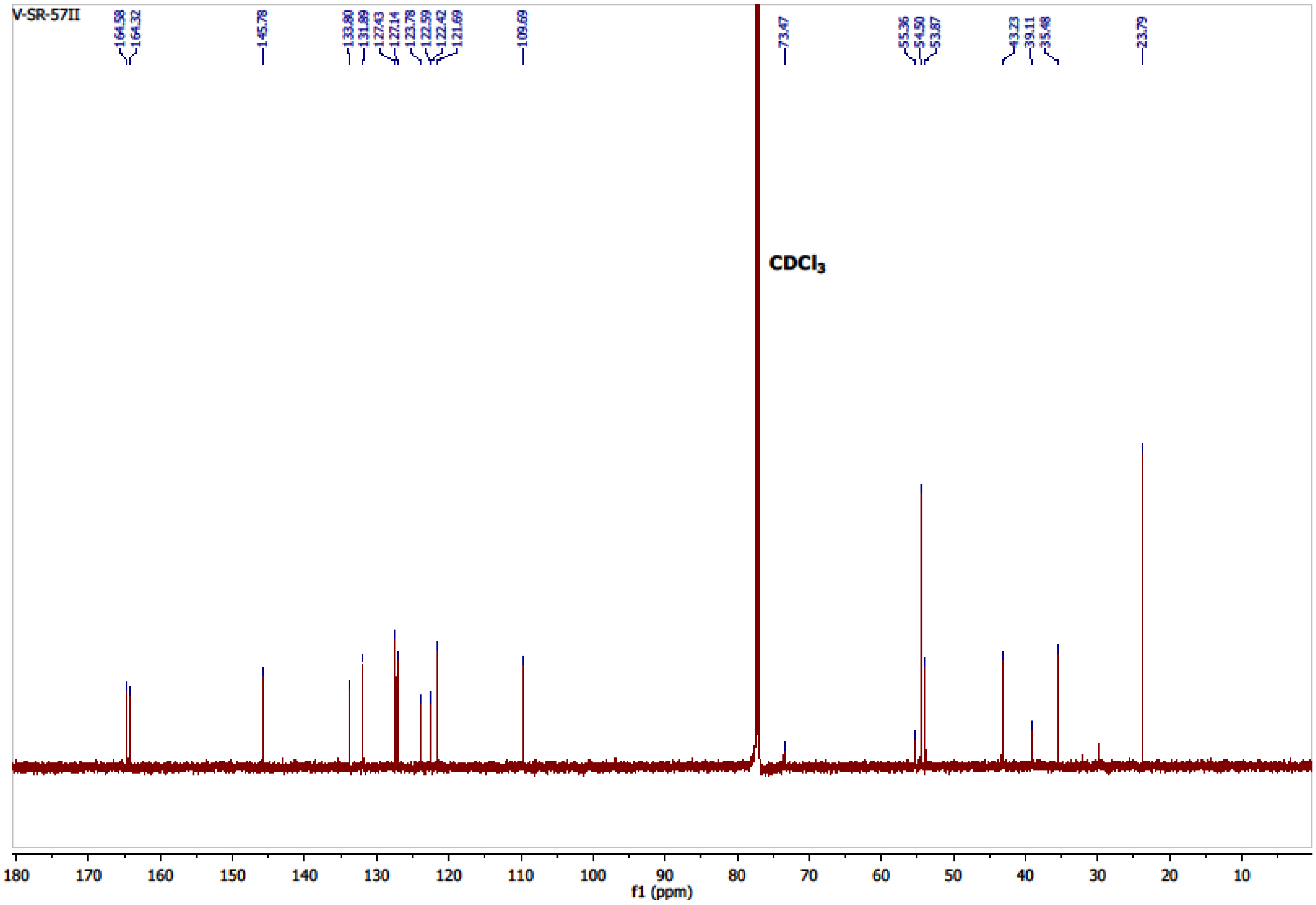


**Figure S124**. $^{13}$C NMR spectrum of **36**.

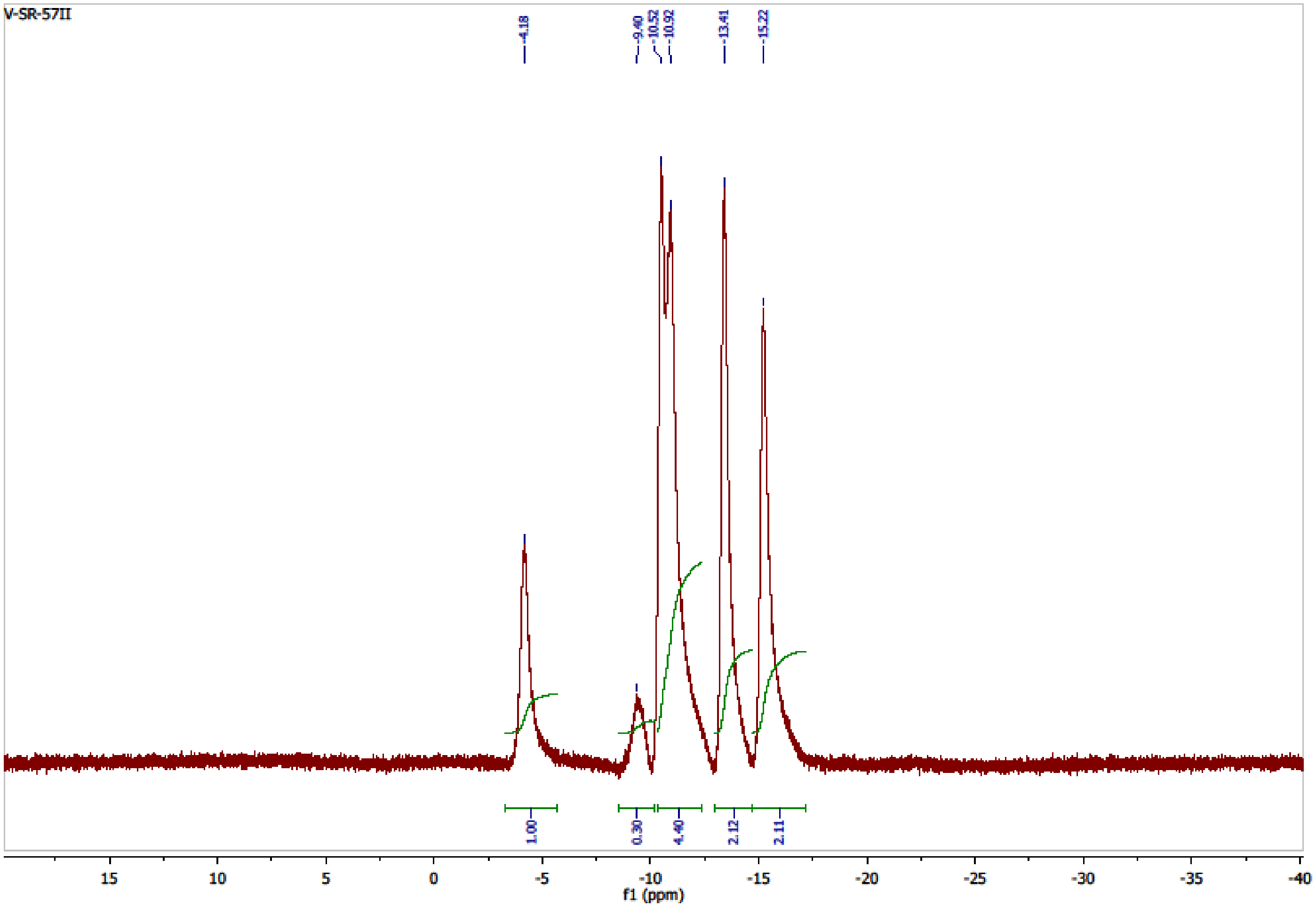


**Figure S125**. $^{11}B$ NMR {$^{1}H$ BB} spectrum of **36**.

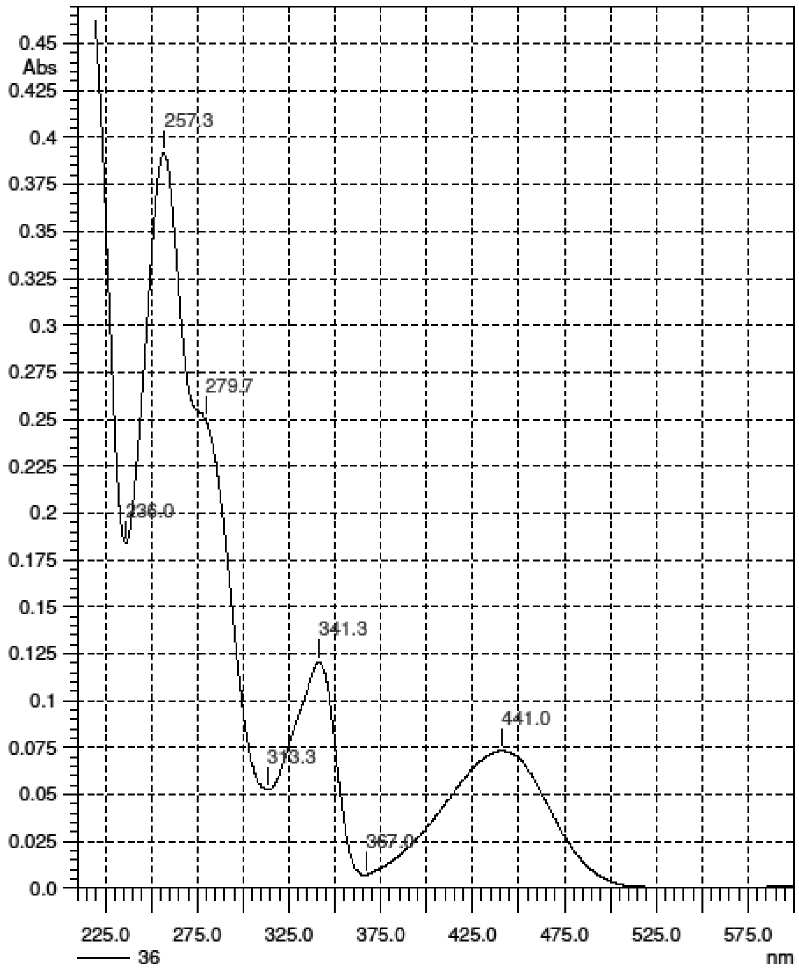


**Figure S126**. UV spectrum of **36**.

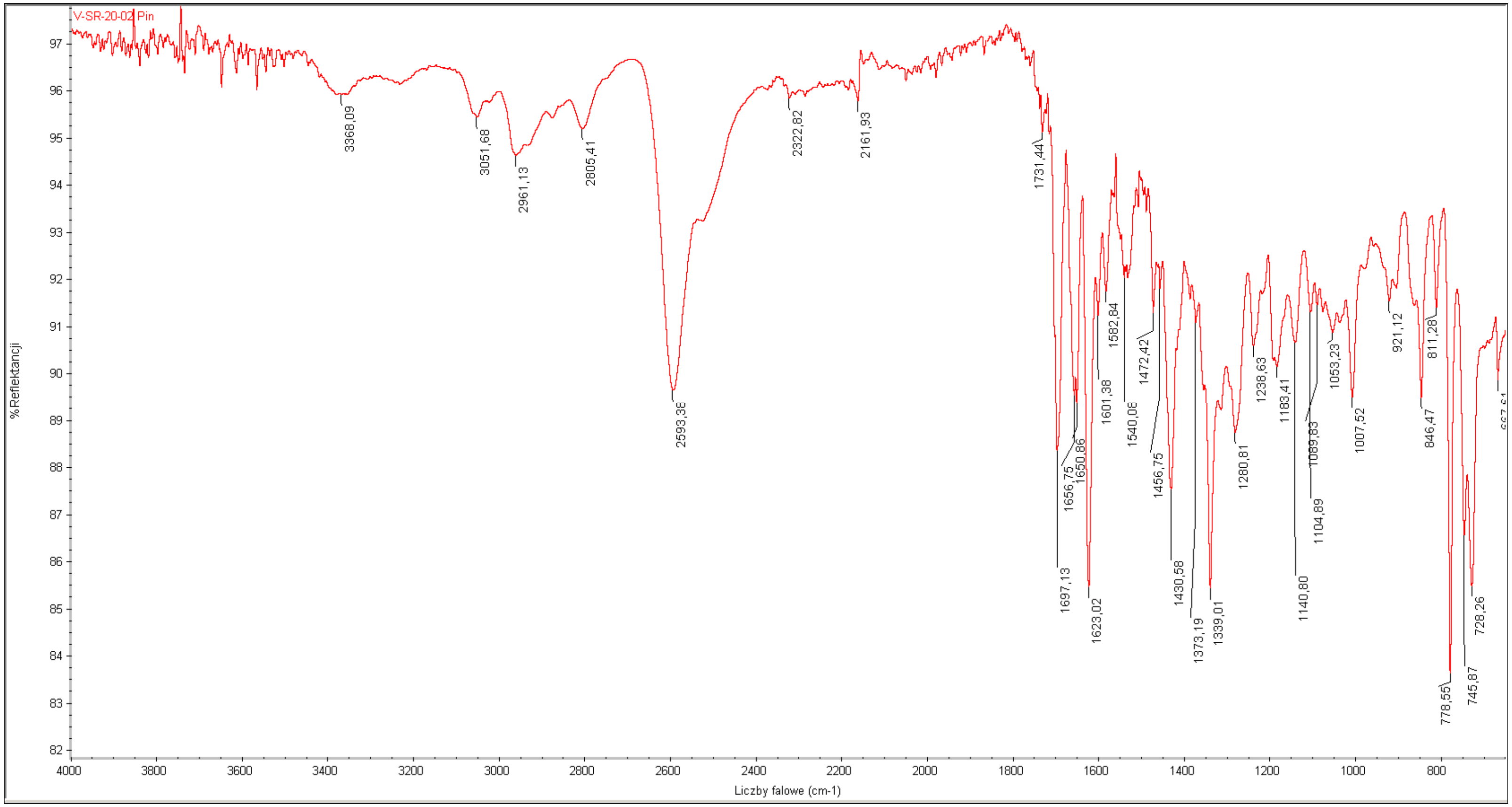


**Figure S127**. IR spectrum of **36**.

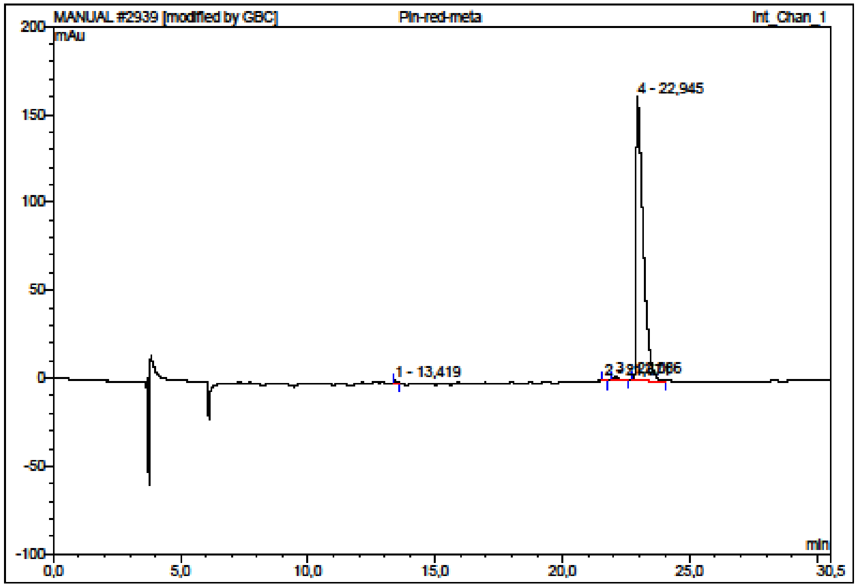


| No. | Ret.Time min | Peak Name | Height mAu | Area mAu*min | Rel.Area % | Amount | Type |
|---|---|---|---|---|---|---|---|
| 1 | 13,42 | n.a. | 1,566 | 0,193 | 0,36 | n.a. | BMB* |
| 2 | 21,67 | n.a. | 0,901 | 0,100 | 0,19 | n.a. | BMB* |
| 3 | 22,09 | n.a. | 2,307 | 0,591 | 1,12 | n.a. | BMB* |
| 4 | 22,95 | n.a. | 161,591 | 52,121 | 98,33 | n.a. | BMB |
| Total: | | | 166,365 | 53,005 | 100,00 | 0,000 | |

**Figure S128**. HPLC analysis of **36**.

Spectrum Name: V-SR-57
Start Ion: 400
End Ion: 600
Source: APCI + 10.0µA 400C
Capillary: 150V 300C Offset: 25V Span: 0V

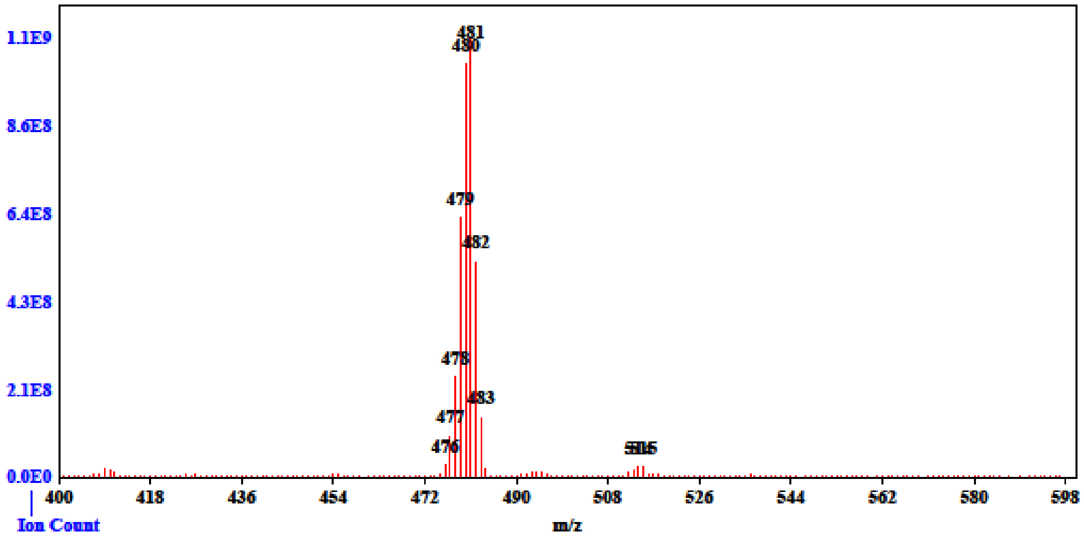


**Figure S129**. MS spectrum of **36**.

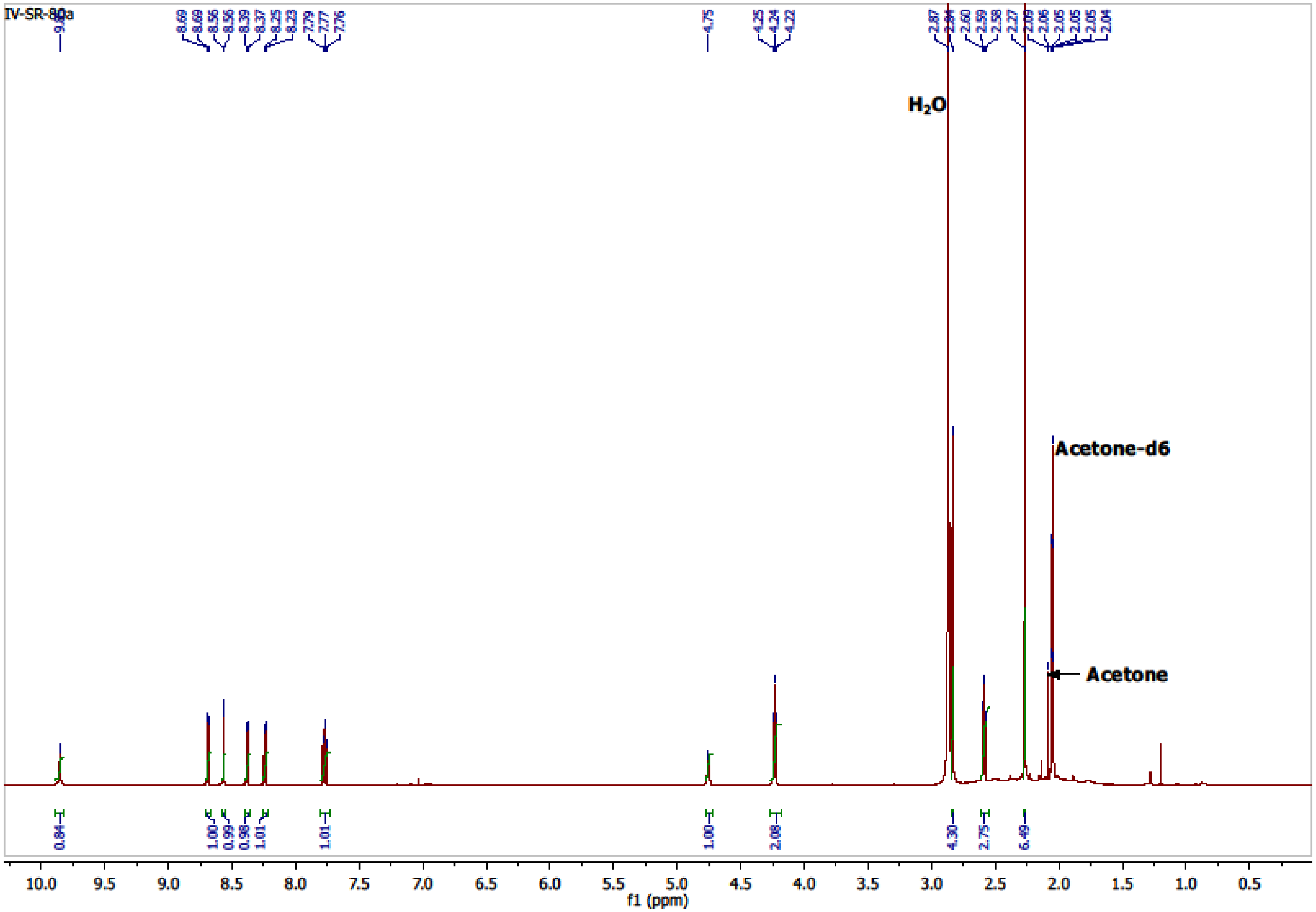


**Figure S130**. $^{1}$H NMR spectrum of **39**.

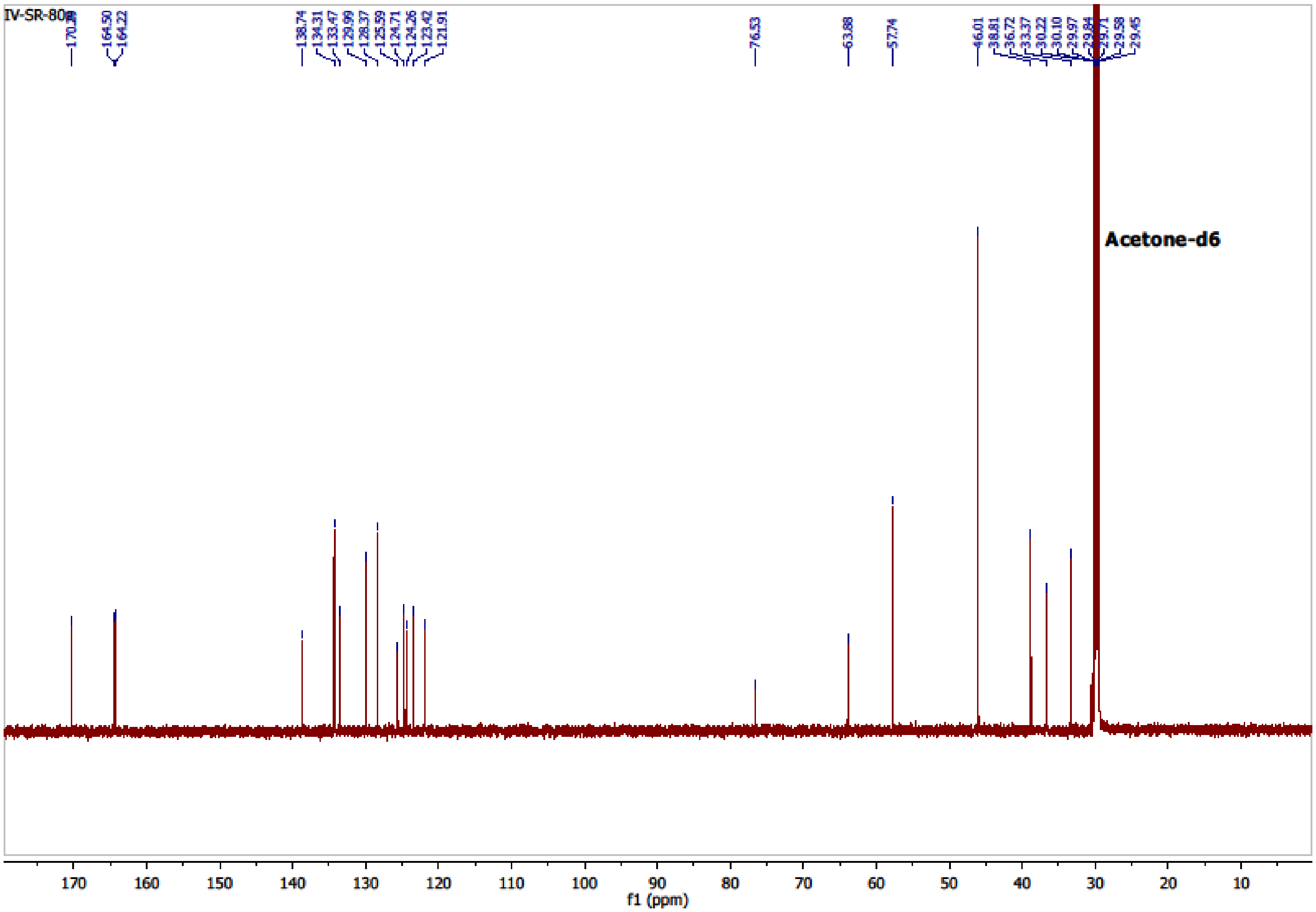


**Figure S131**. $^{13}$C NMR spectrum of **39**.

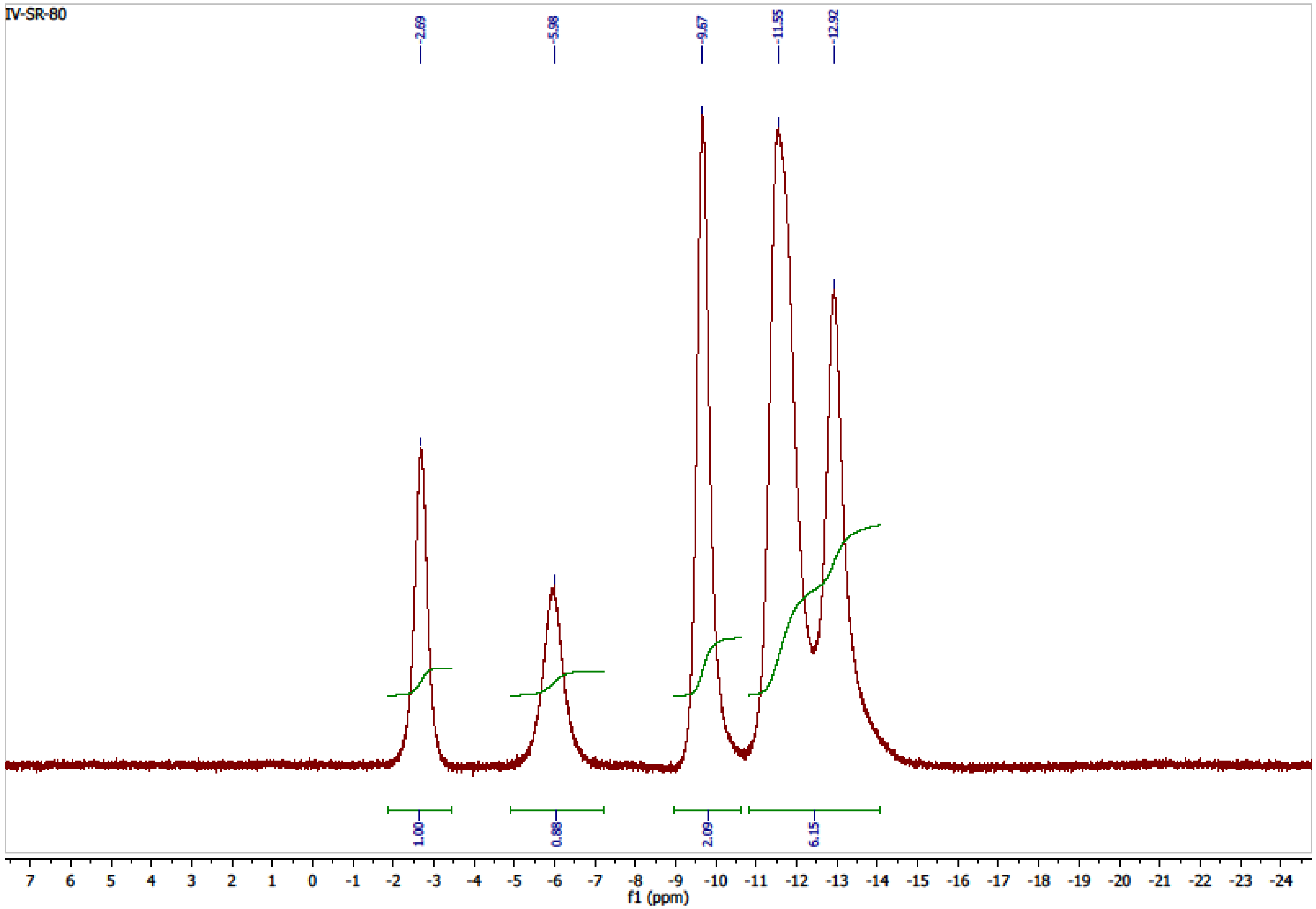


**Figure S132**. $^{11}B$ NMR {$^{1}H$ BB} spectrum of **39**.

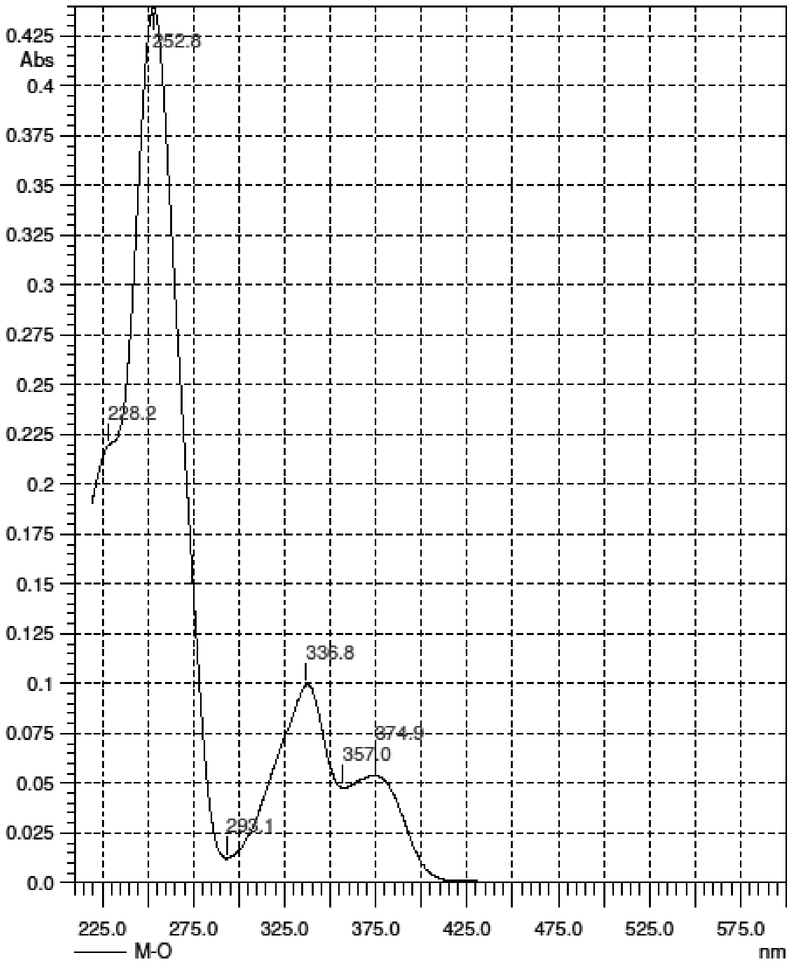


**Figure S133**. UV spectrum of **39**.

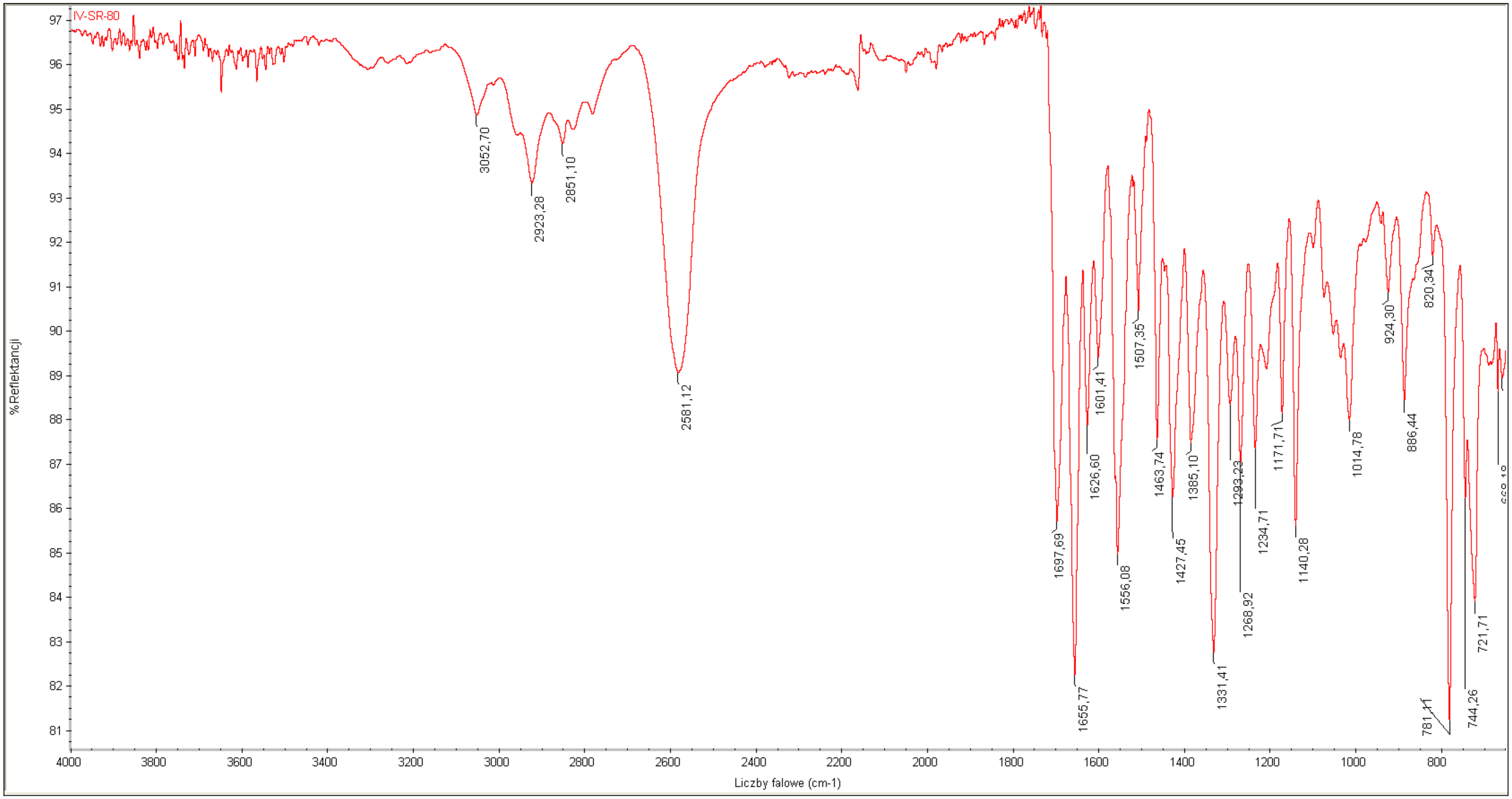


**Figure S134**. IR spectrum of **39**.

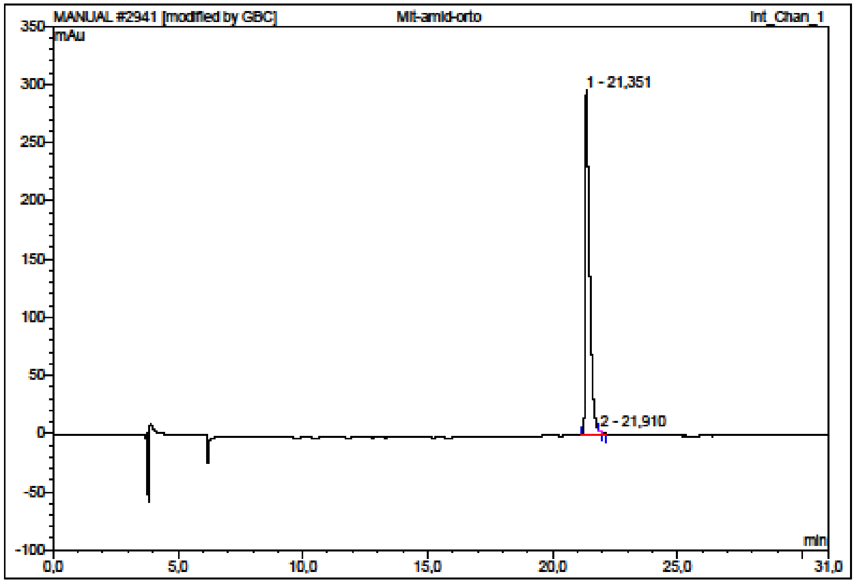


| No. | Ret.Time min | Peak Name | Height mAu | Area mAu*min | Rel.Area % | Amount | Type |
|---|---|---|---|---|---|---|---|
| 1 | 21,35 | n.a. | 296,387 | 60,775 | 99,89 | n.a. | BMB* |
| 2 | 21,91 | n.a. | 0,740 | 0,068 | 0,11 | n.a. | Rd* |
| Total: | | | 297,127 | 60,843 | 100,00 | 0,000 | |

**Figure S135**. HPLC analysis of **39**.

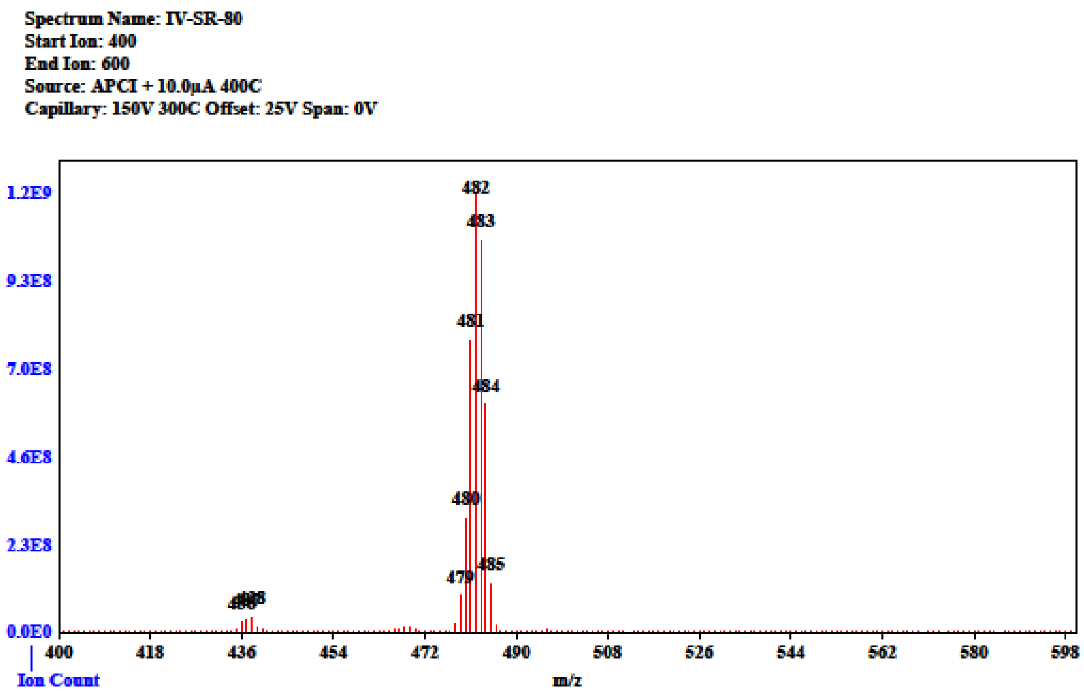


**Figure S136**. MS spectrum of **39**.

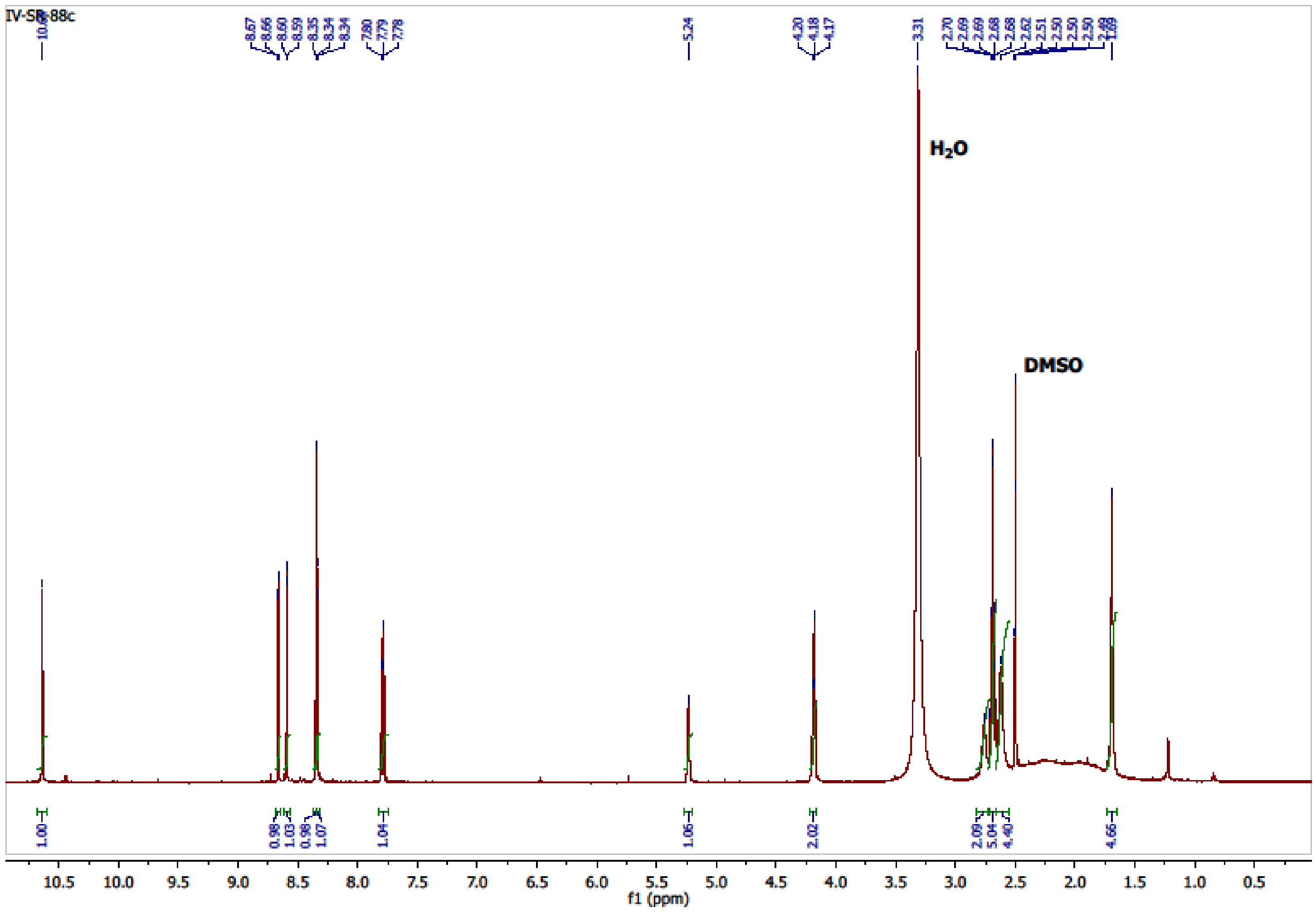


**Figure S137**. $^{1}$H NMR spectrum of **40**.

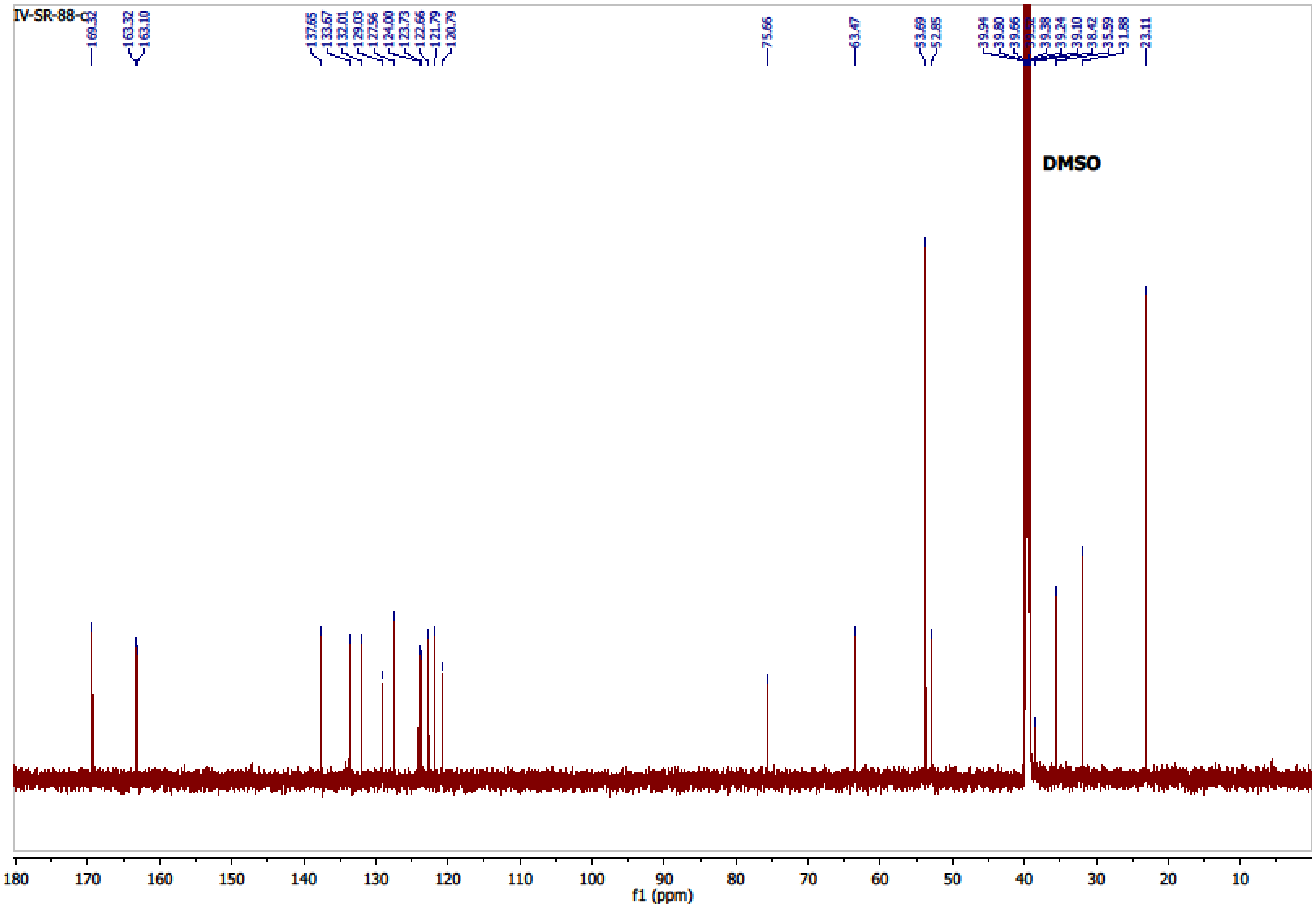


**Figure S138**. $^{13}C$ NMR spectrum of **40**.

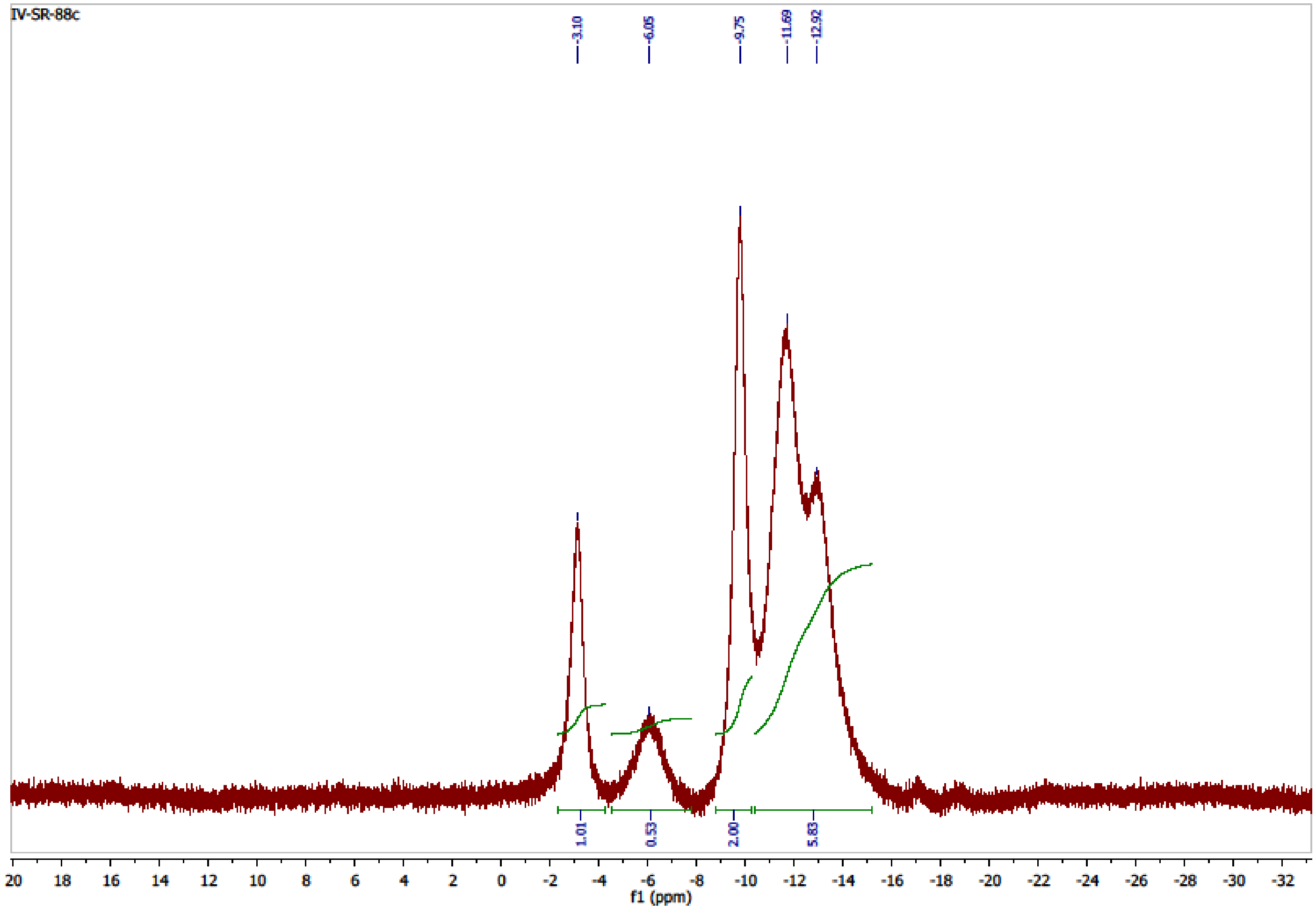


**Figure S139**. $^{11}B$ NMR {$^{1}H$ BB} spectrum of **40**.

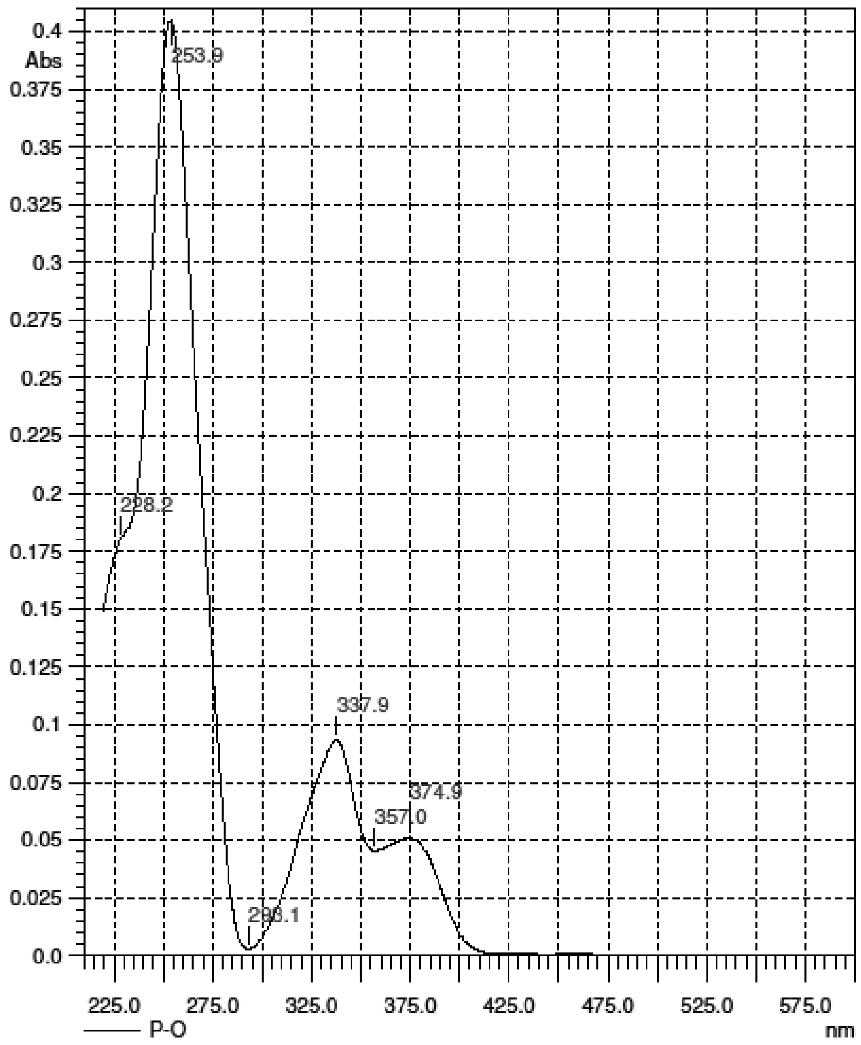


**Figure S140**. UV spectrum of **40**.

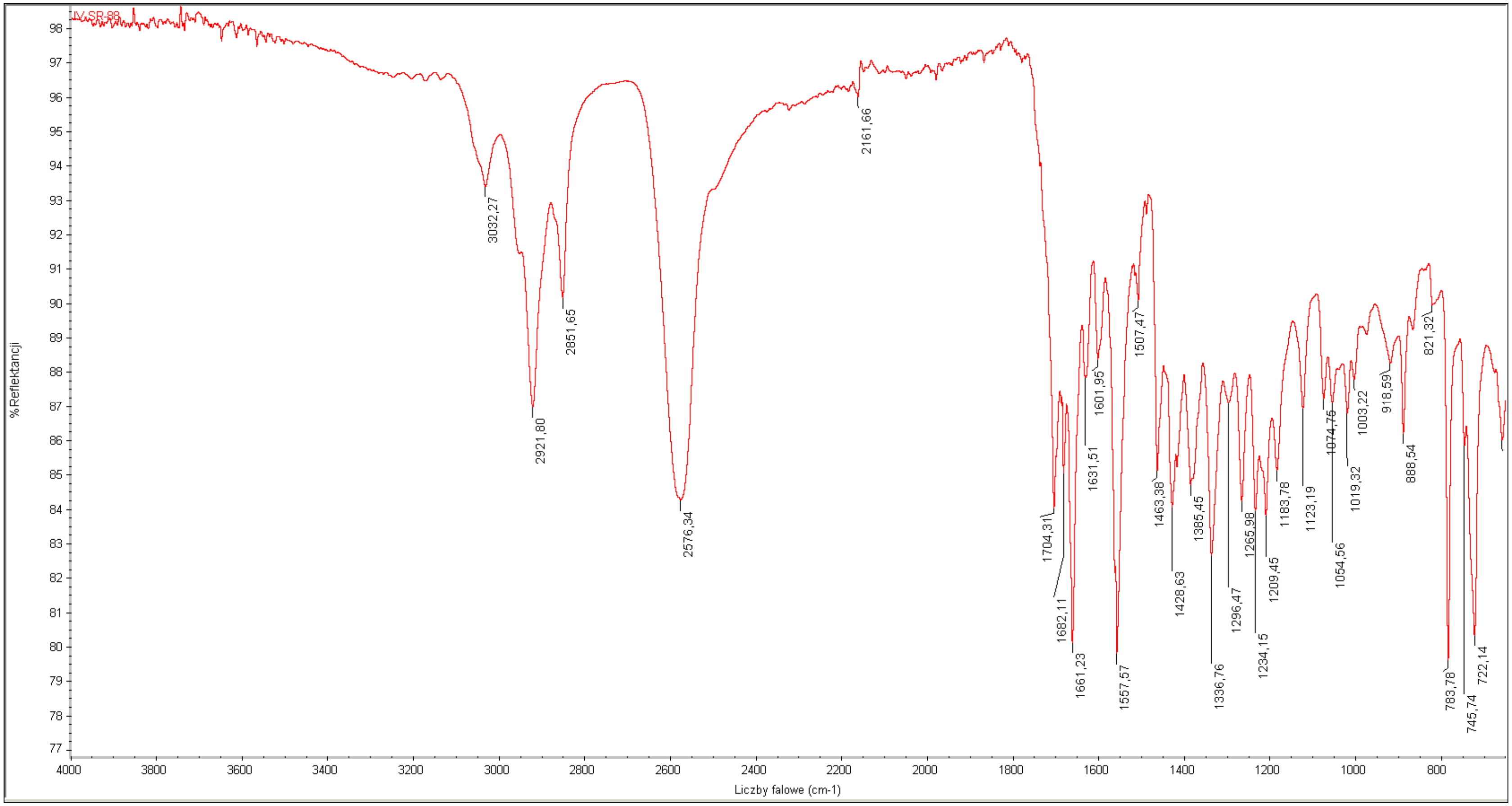


**Figure S141**. IR spectrum of **40**.

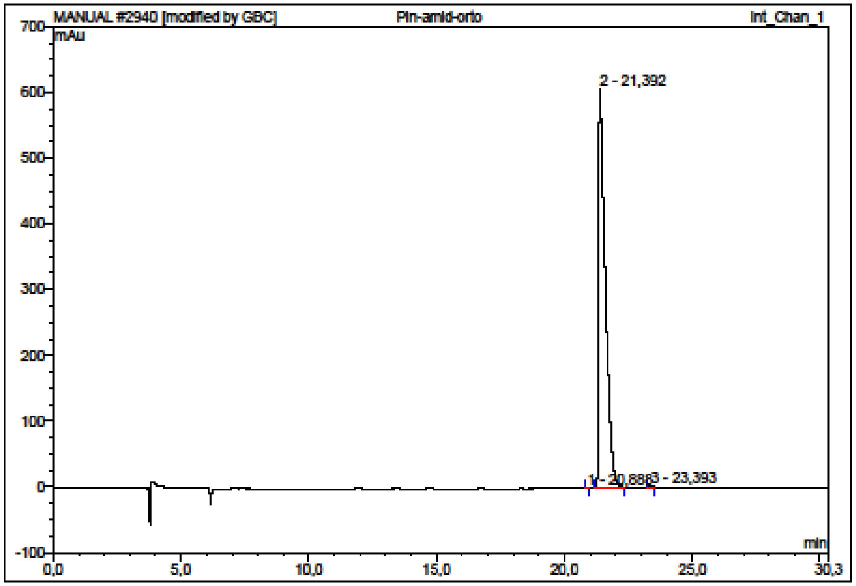


| No. | Ret.Time min | Peak Name | Height mAu | Area mAu*min | Rel.Area % | Amount | Type |
|---|---|---|---|---|---|---|---|
| 1 | 20,89 | n.a. | 0,743 | 0,064 | 0,03 | n.a. | BMB* |
| 2 | 21,39 | n.a. | 606,469 | 181,277 | 99,65 | n.a. | BMB |
| 3 | 23,39 | n.a. | 3,461 | 0,571 | 0,31 | n.a. | BMB* |
| Total: | | | 610,674 | 181,911 | 100,00 | 0,000 | |

**Figure S142**. HPLC analysis of **40**.

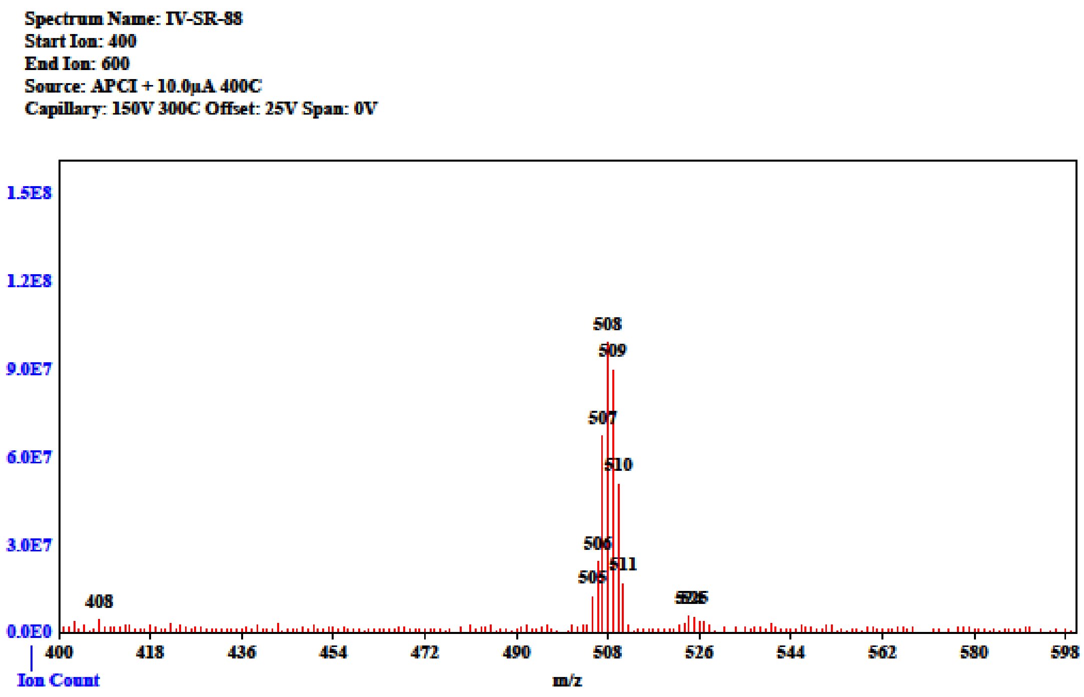


**Figure S143**. MS spectrum of **40**.

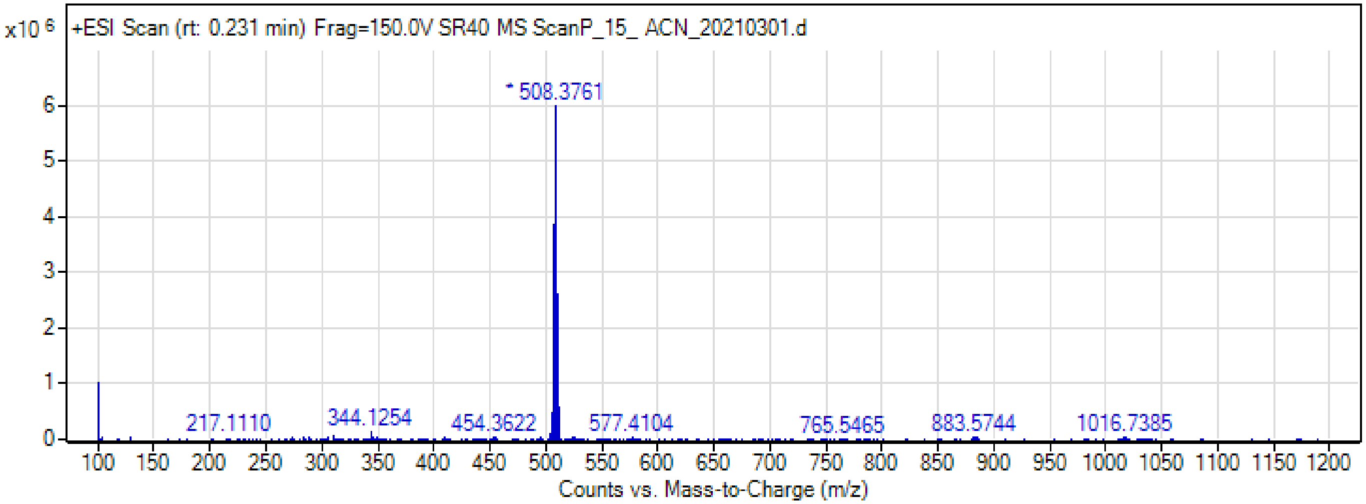


**Figure S144**. HRMS spectrum of **40**.

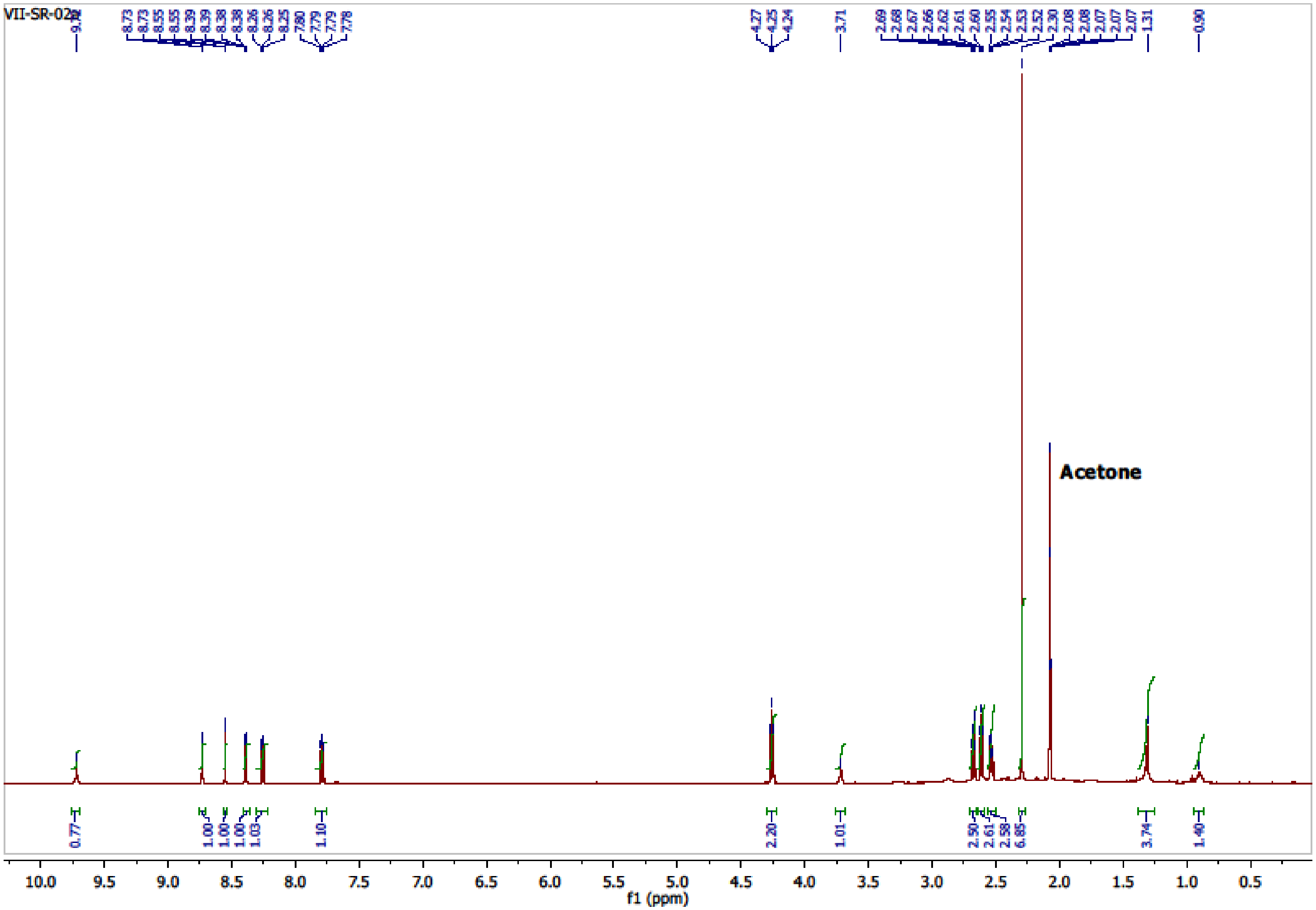


**Figure S145**. $^{1}$H NMR spectrum of **41**.

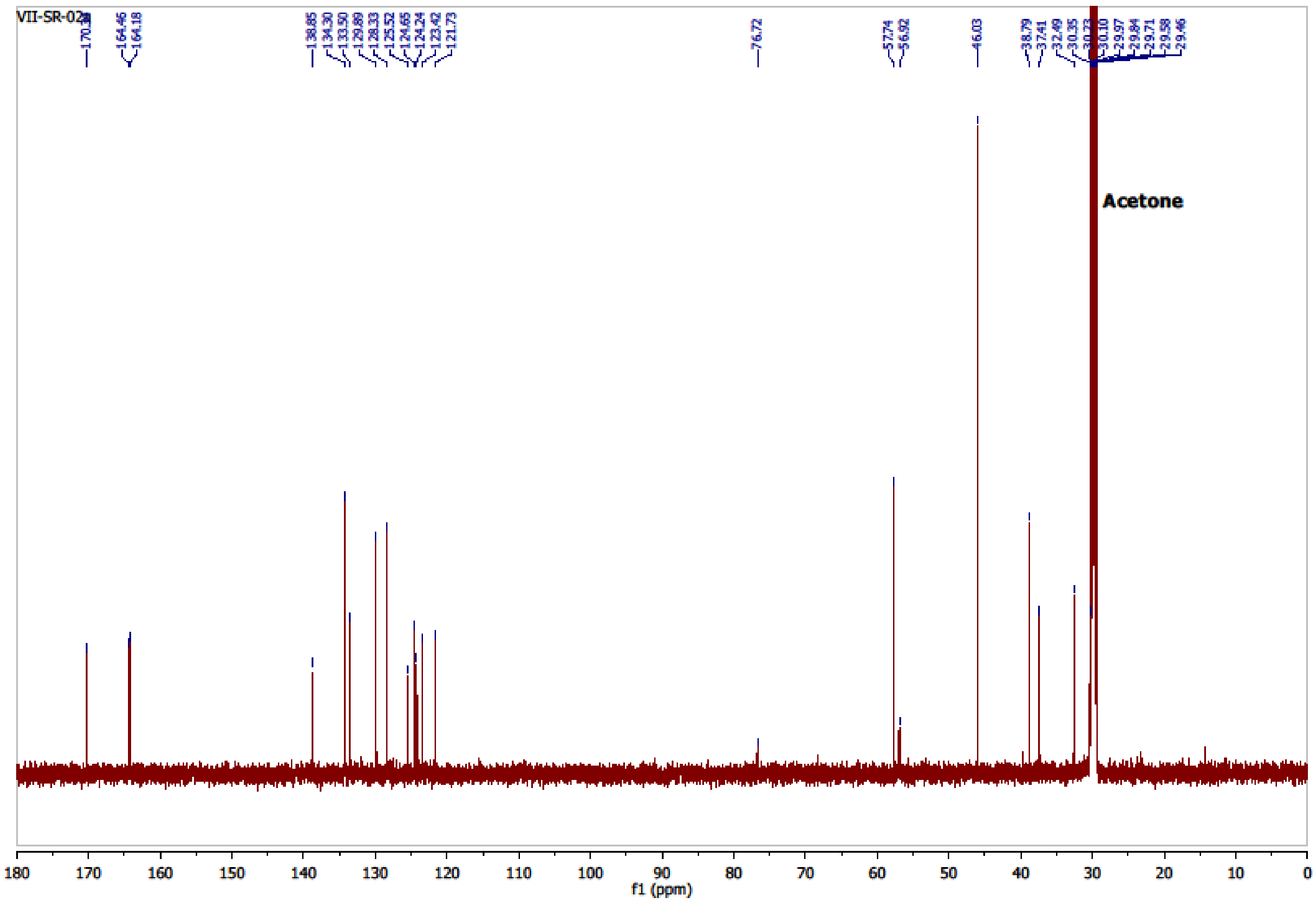


**Figure S146**. $^{13}C$ NMR spectrum of **41**.

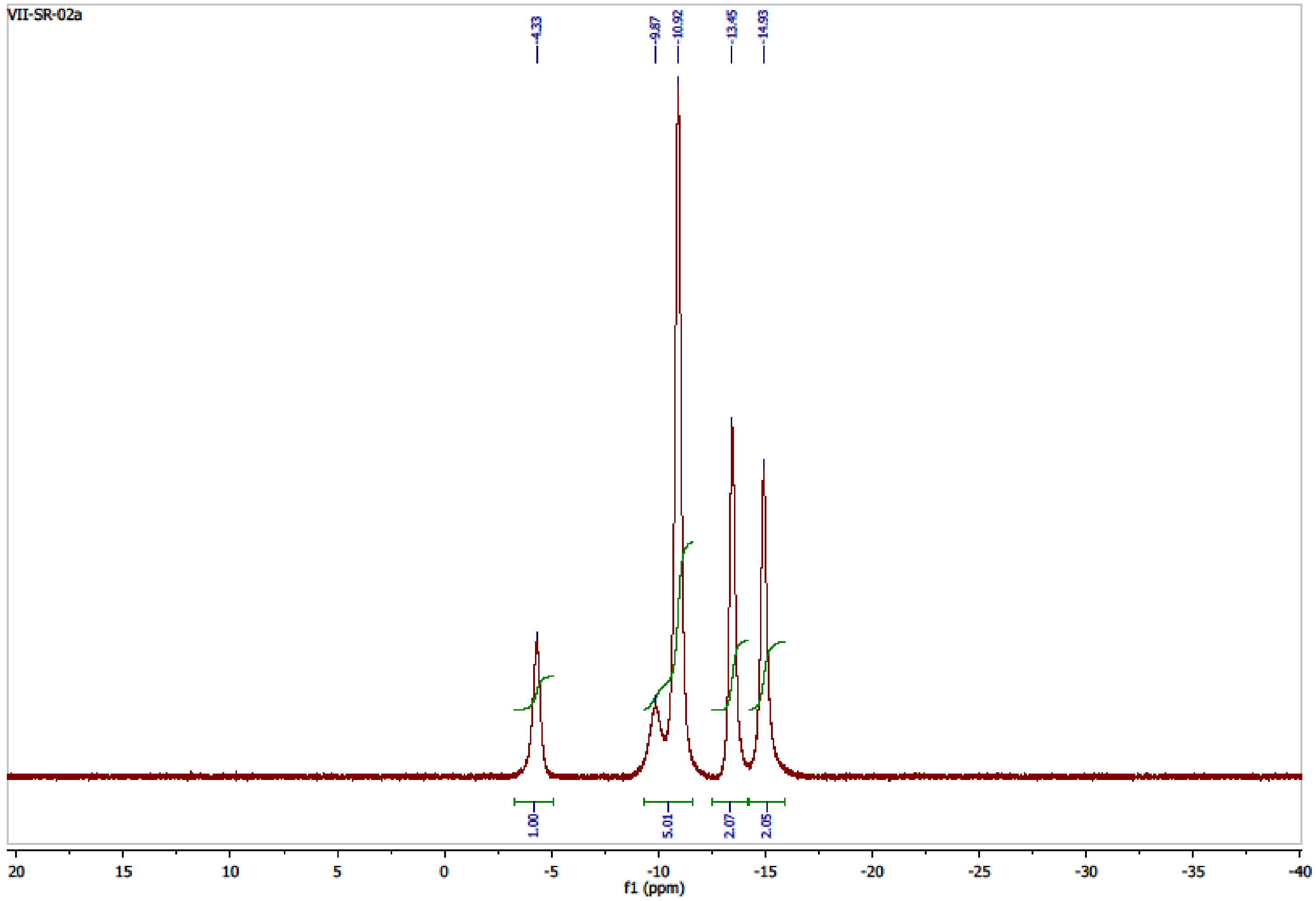


**Figure S147**. IR spectrum of **41**.

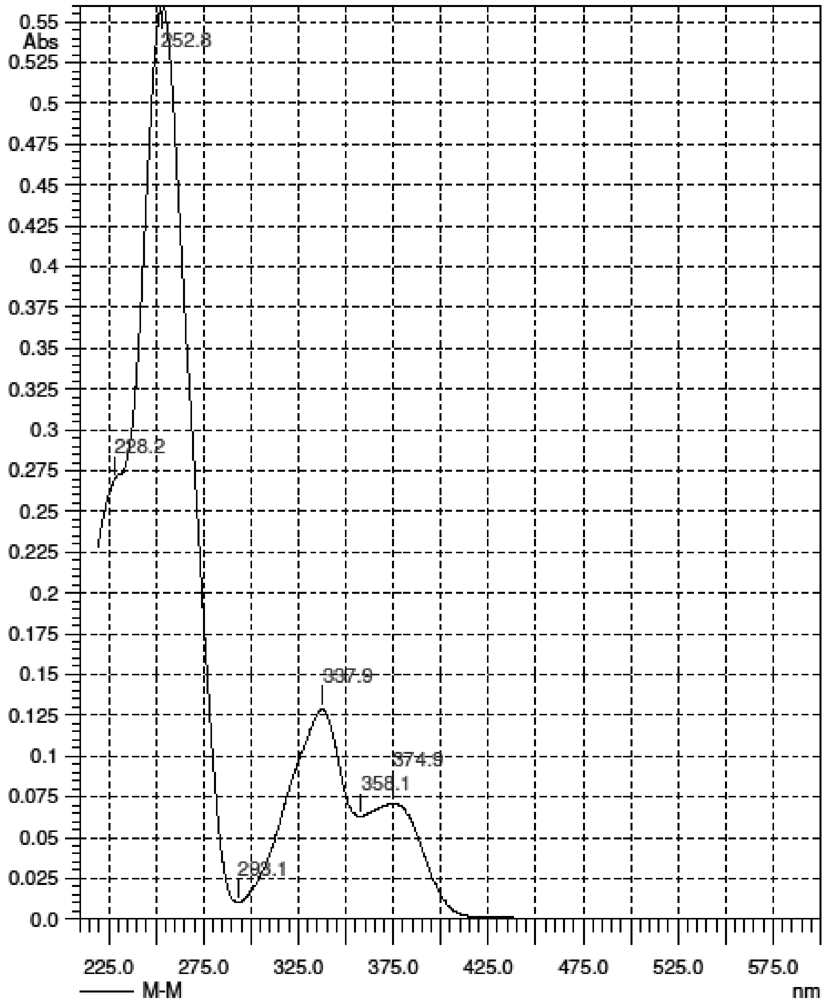


**Figure S148**. UV spectrum of **41**.

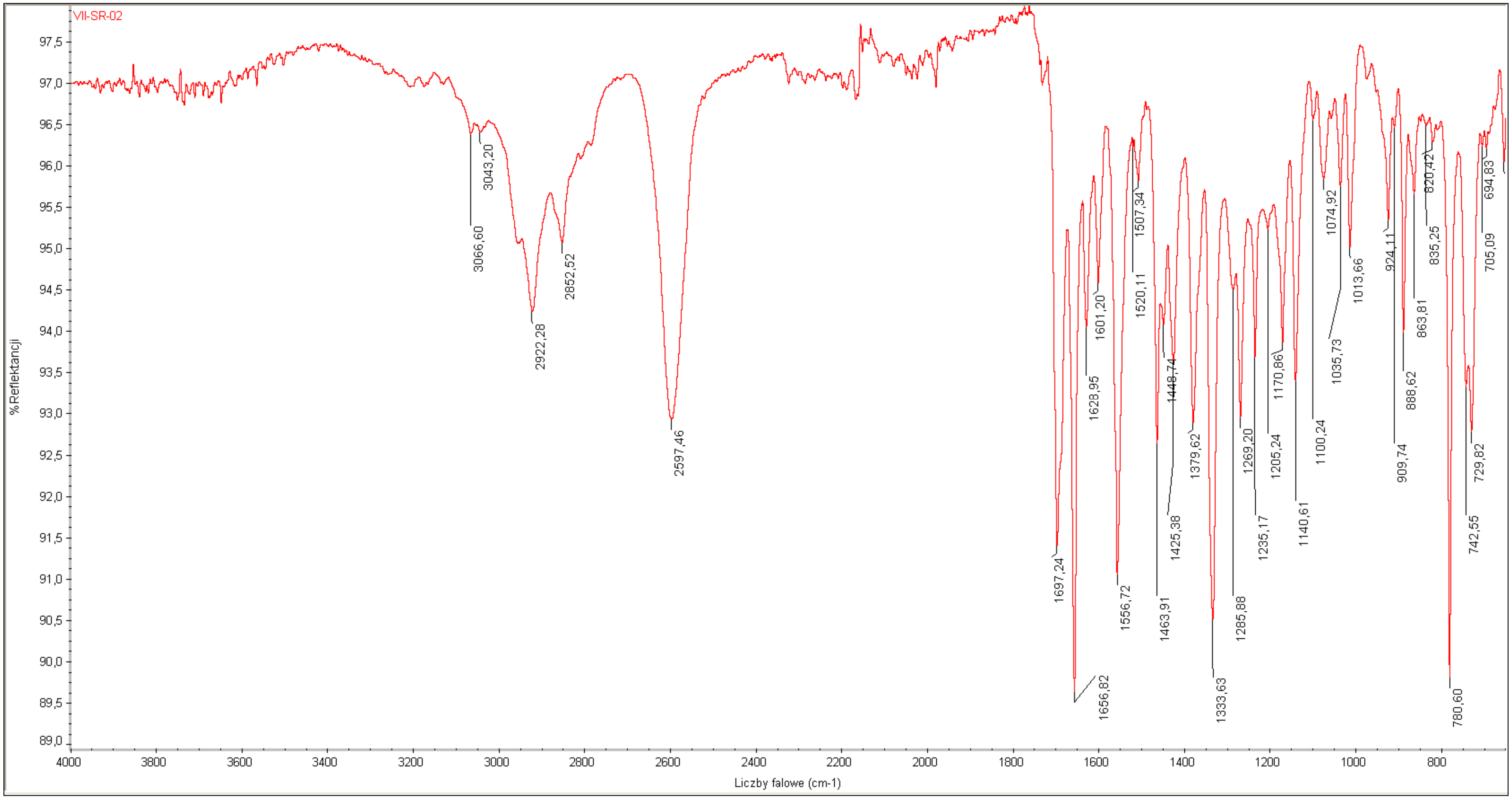


**Figure S149**. IR spectrum of **41**.

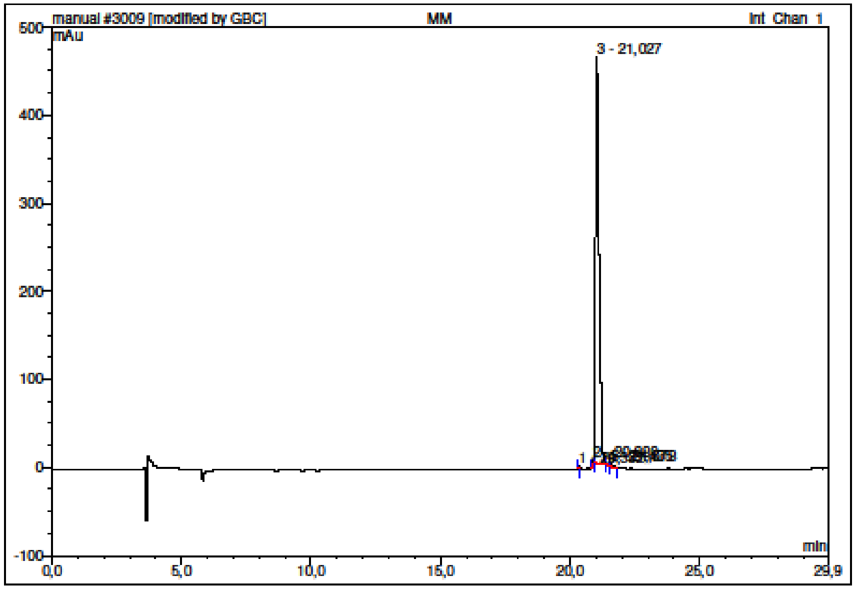


| No. | Ret.Time min | Peak Name | Height mAu | Area mAu*min | Rel.Area % | Amount | Type |
|---|---|---|---|---|---|---|---|
| 1 | 20,33 | n.a. | 3,809 | 0,284 | 0,39 | n.a. | BMB* |
| 2 | 20,88 | n.a. | 6,404 | 0,384 | 0,52 | n.a. | BMb* |
| 3 | 21,03 | n.a. | 460,312 | 72,780 | 98,54 | n.a. | bMB* |
| 4 | 21,44 | n.a. | 1,057 | 0,068 | 0,09 | n.a. | BMB* |
| 5 | 21,58 | n.a. | 2,437 | 0,339 | 0,46 | n.a. | BMB* |
| Total: | | | 474,019 | 73,856 | 100,00 | 0,000 | |

**Figure S150**. HPLC analysis of **41**.

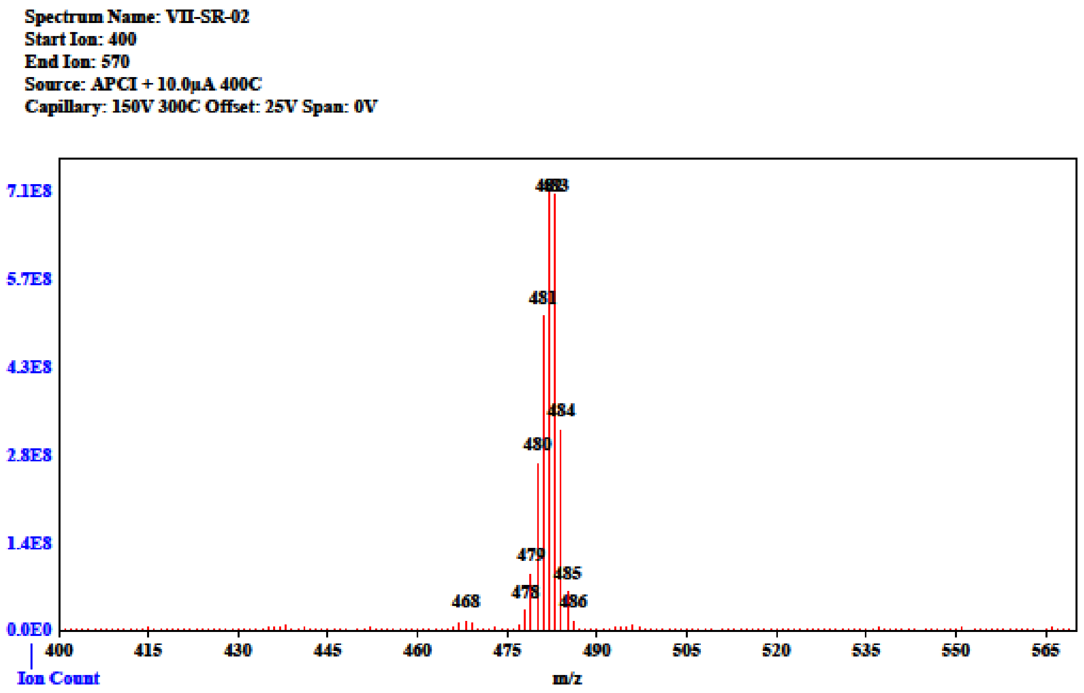


**Figure S151**. MS spectrum of **41**.

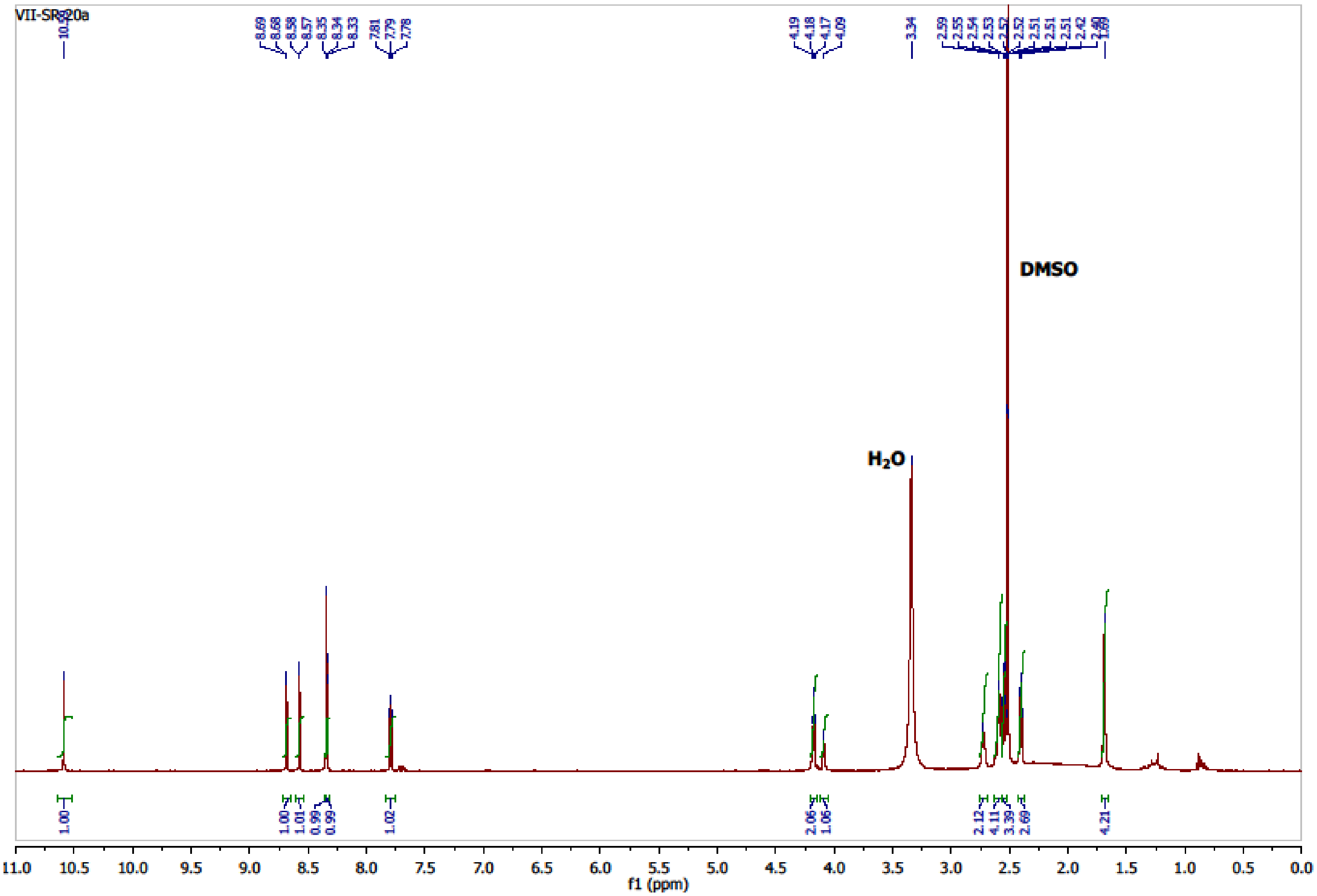

**Figure S152**. $^{1}$H NMR spectrum of **42**.

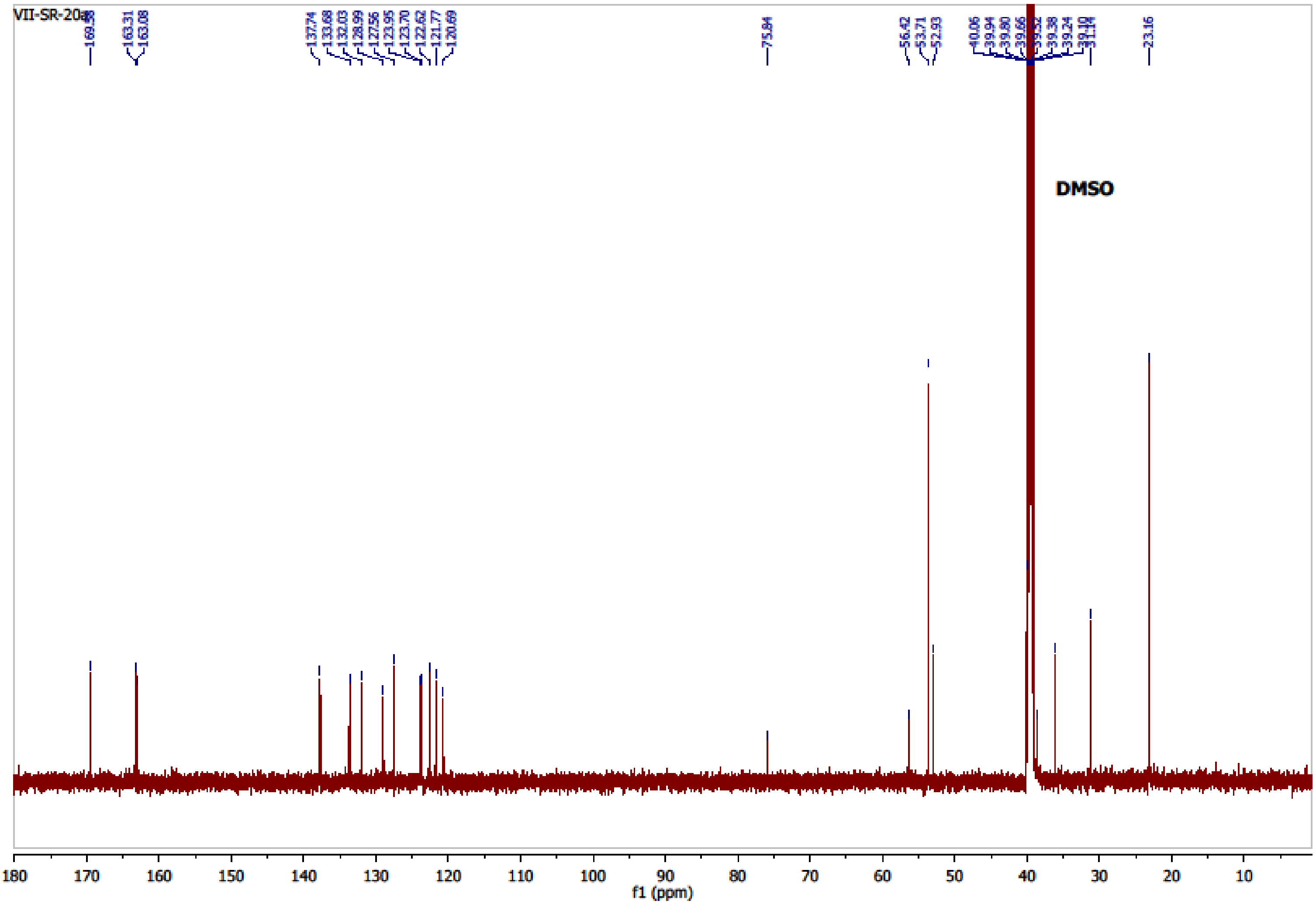


**Figure S153**. $^{13}$C NMR spectrum of **42**.

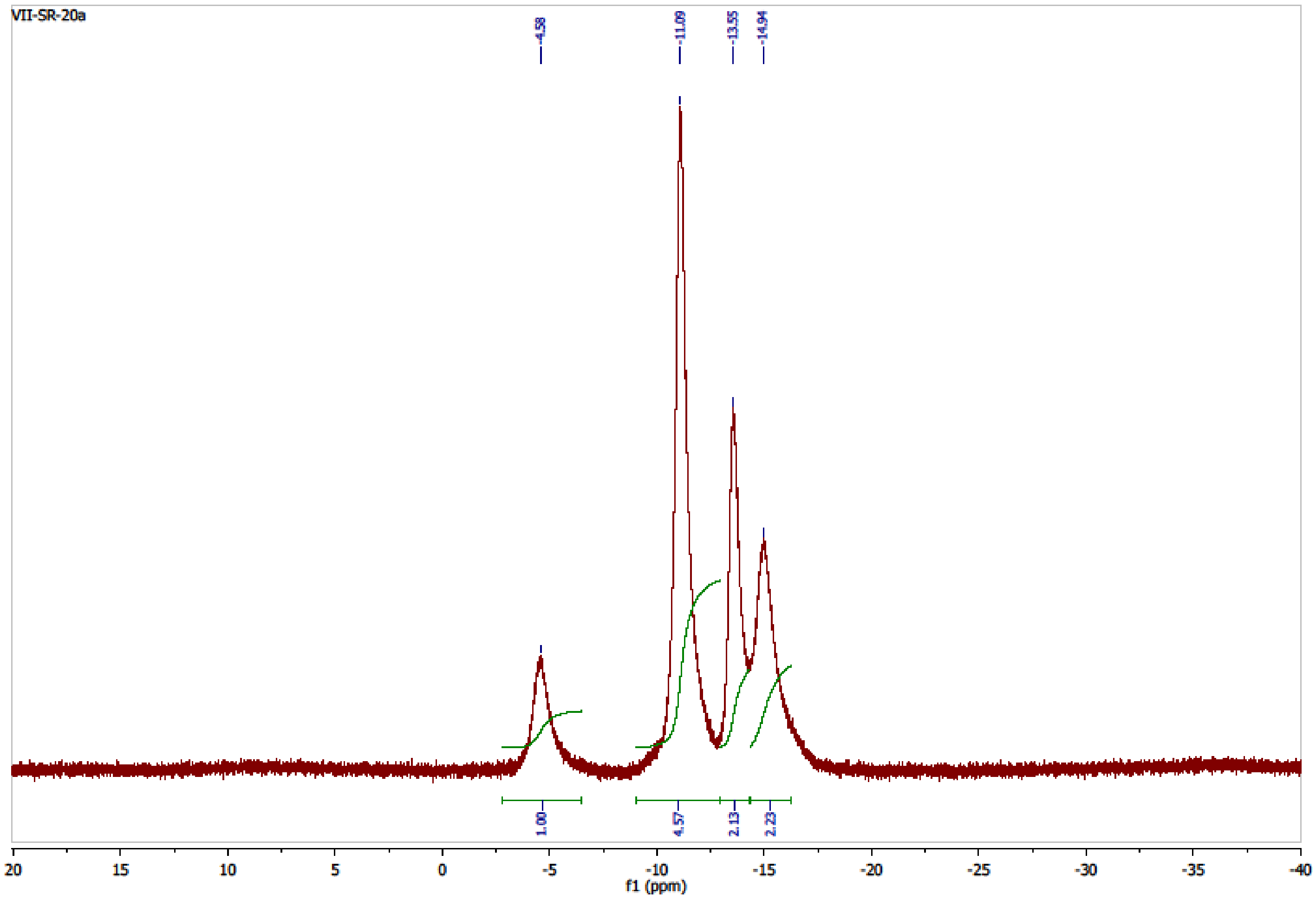


**Figure S154**. IR spectrum of **42**.

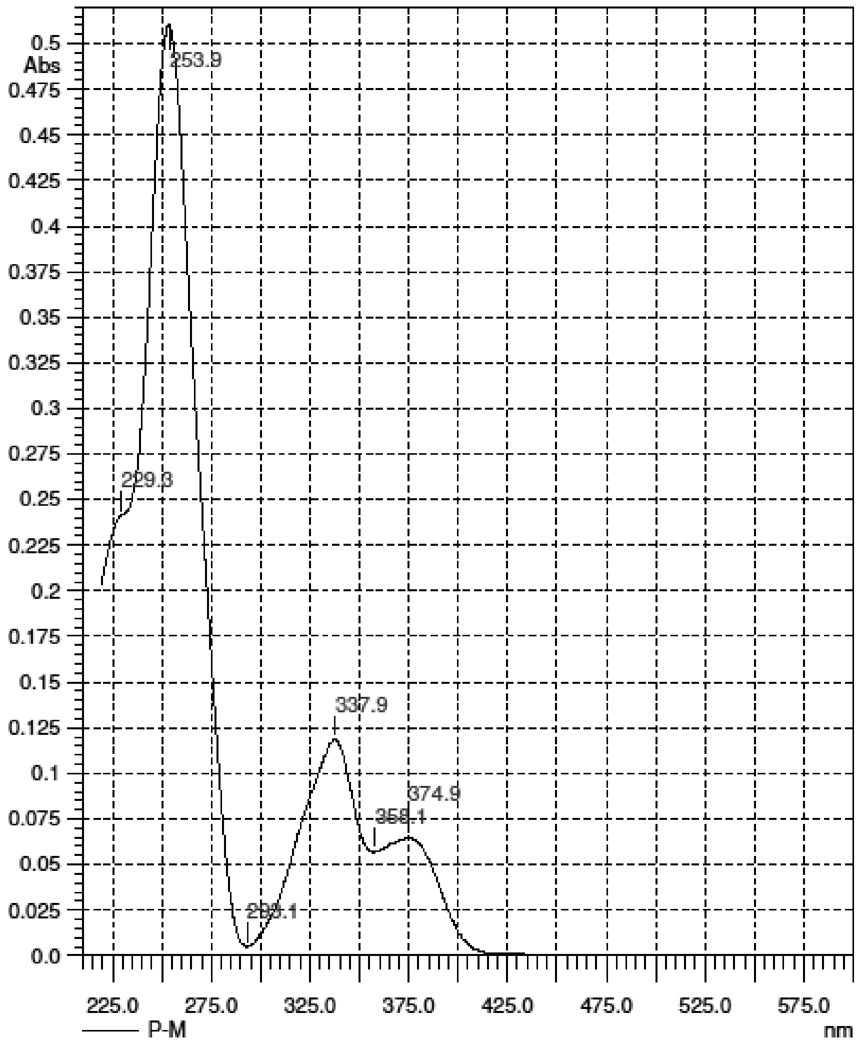


**Figure S155**. UV spectrum of **42**.

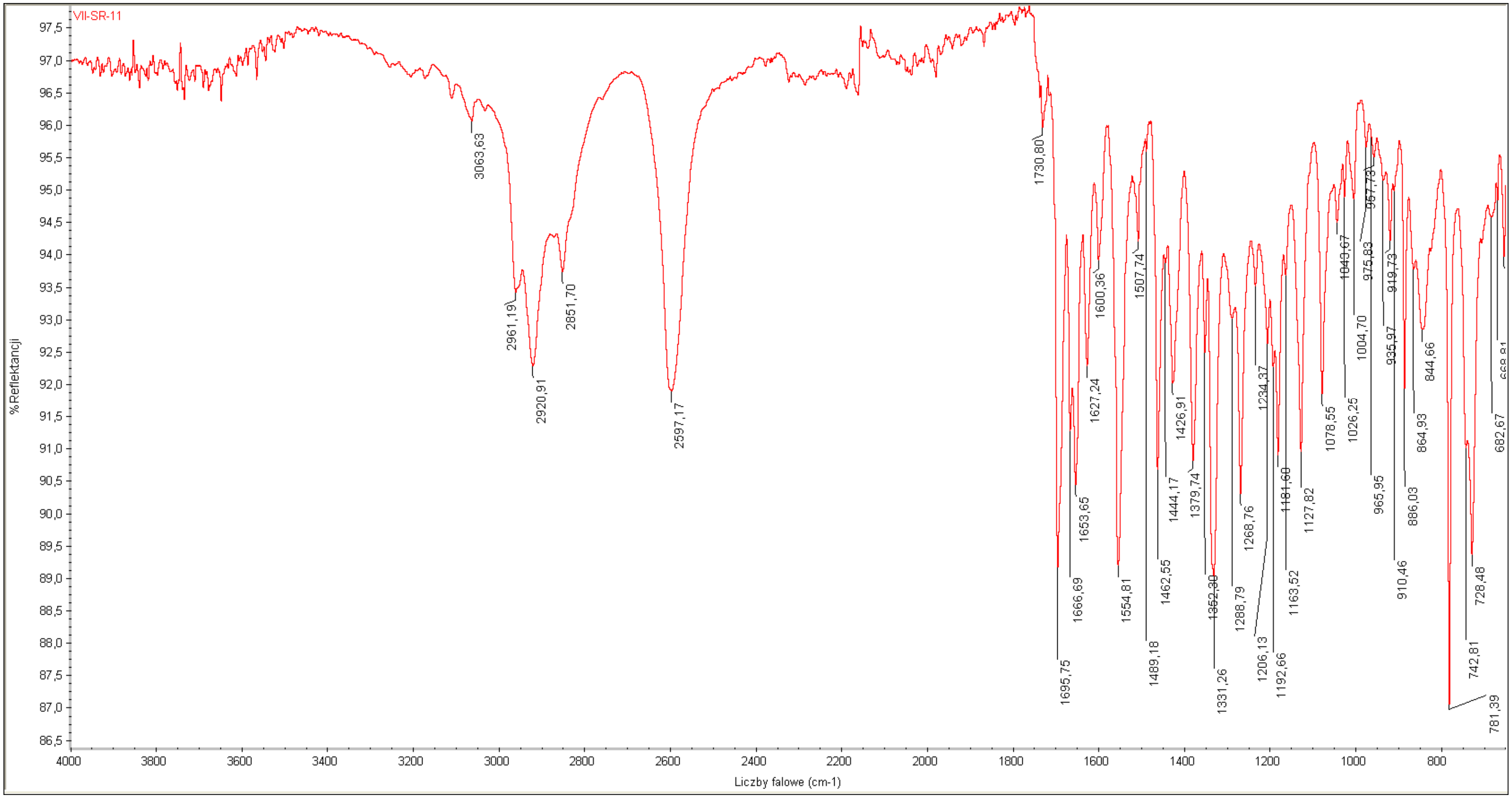


**Figure S156**. IR spectrum of **42**.

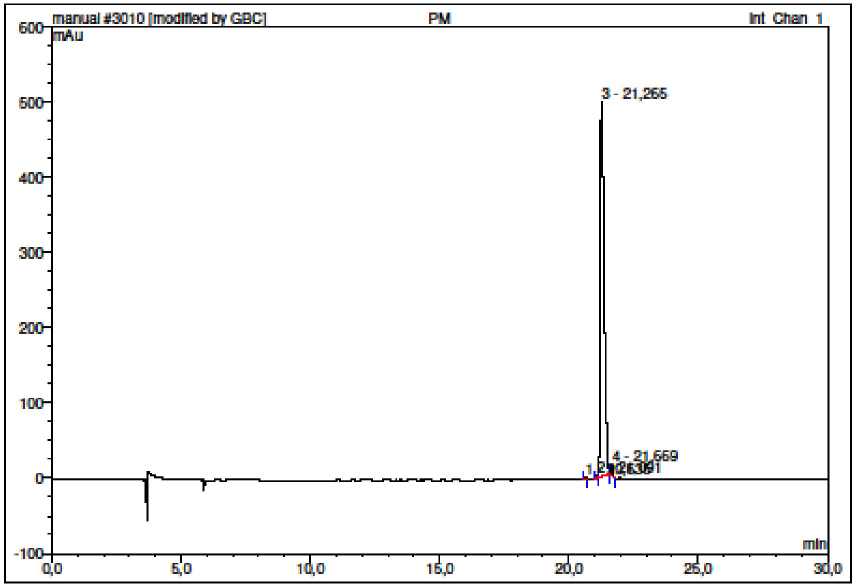


| No. | Ret.Time min | Peak Name | Height mAu | Area mAu*min | Rel.Area % | Amount | Type |
|---|---|---|---|---|---|---|---|
| 1 | 20,63 | n.a. | 2,620 | 0,196 | 0,23 | n.a. | BMB* |
| 2 | 21,09 | n.a. | 2,455 | 0,155 | 0,18 | n.a. | BMB* |
| 3 | 21,27 | n.a. | 499,429 | 85,637 | 98,17 | n.a. | BMB |
| 4 | 21,67 | n.a. | 14,407 | 1,245 | 1,43 | n.a. | BMB* |
| Total: | | | 518,910 | 87,233 | 100,00 | 0,000 | |

**Figure S157**. HPLC analysis of **42**.

Spectrum Name: VII-SR-11
Start Ion: 400
End Ion: 600
Source: APCI + 10.0µA 400C
Capillary: 150V 300C Offset: 25V Span: 0V

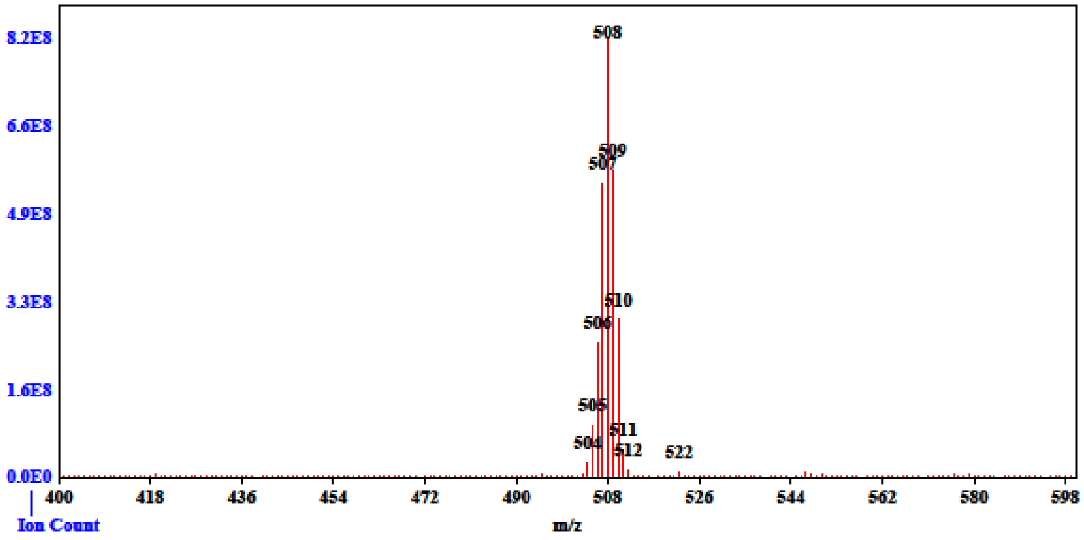


**Figure S158**. MS spectrum of **42**.

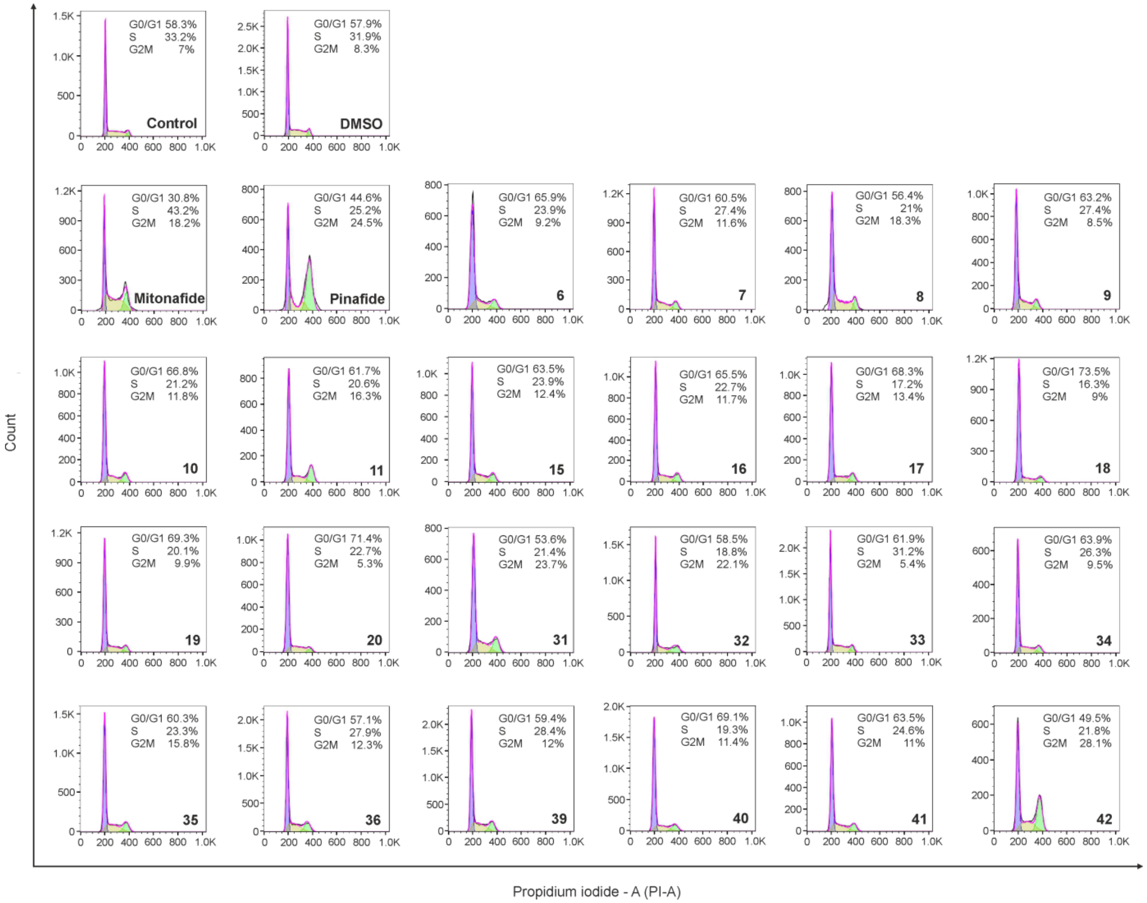


**Figure S159**. Influence of compound **6** (115 μM), **7** (104 μM), **8** (4 μM), **9** (3 μM), **10** (8 μM), **11** (5 μM), **15** (68 μM), **16** (61 μM), **17** (10 μM), **18** (15 μM), **19** (14 μM), **20** (12 μM), **31**, (53 μM), **32** (42 μM), **33** (5 μM), **34** (8 μM), **35** (9 μM), **36** (6 μM), **39** (11 μM), **40** (13 μM), **41** (10 μM), **42** (6 μM) on cell cycle distribution in HepG2 cells. Flow cytometry analysis of cells treated for 24 h with tested compounds. One representative experiment of three is shown.

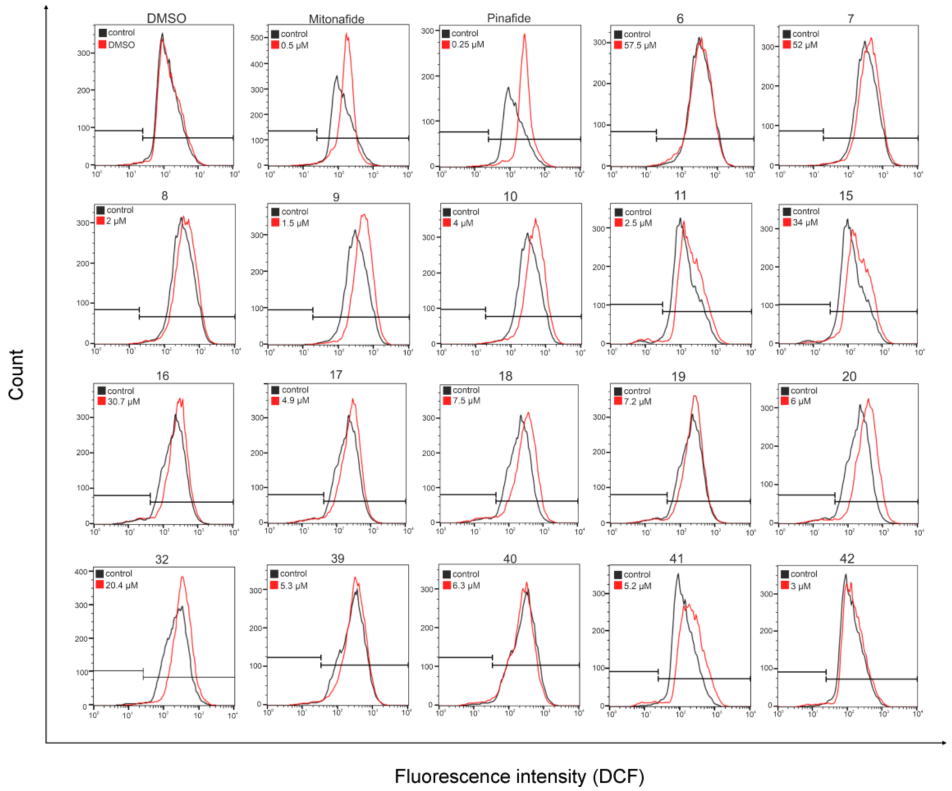


**Figure S160**. ROS production in HepG2 cells after 24 h incubation with compound **6** (57.5 µM), **7** (52 µM), **8** (2 µM), **9** (1.5 µM), **10** (4 µM), **11** (2.5 µM), **15** (34 µM), **16** (30.7 µM), **17** (4.9 µM), **18** (7.5 µM), **19** (7.2 µM), **20** (6 µM), **32** (20.4 µM), **39** (5.3 µM), **40** (6.3 µM), **41** (5.2 µM), **42** (3 µM). Intracellular production of ROS was measured using dual $H_2DCFDA$/PI staining. The intensity of DCF fluorescence corresponds to intracellular ROS level in HepG2 cells. The flow cytometric analysis indicated DCF fluorescence shift, which is correlated with higher ROS production after 24 h incubation with the tested compounds compared to control.

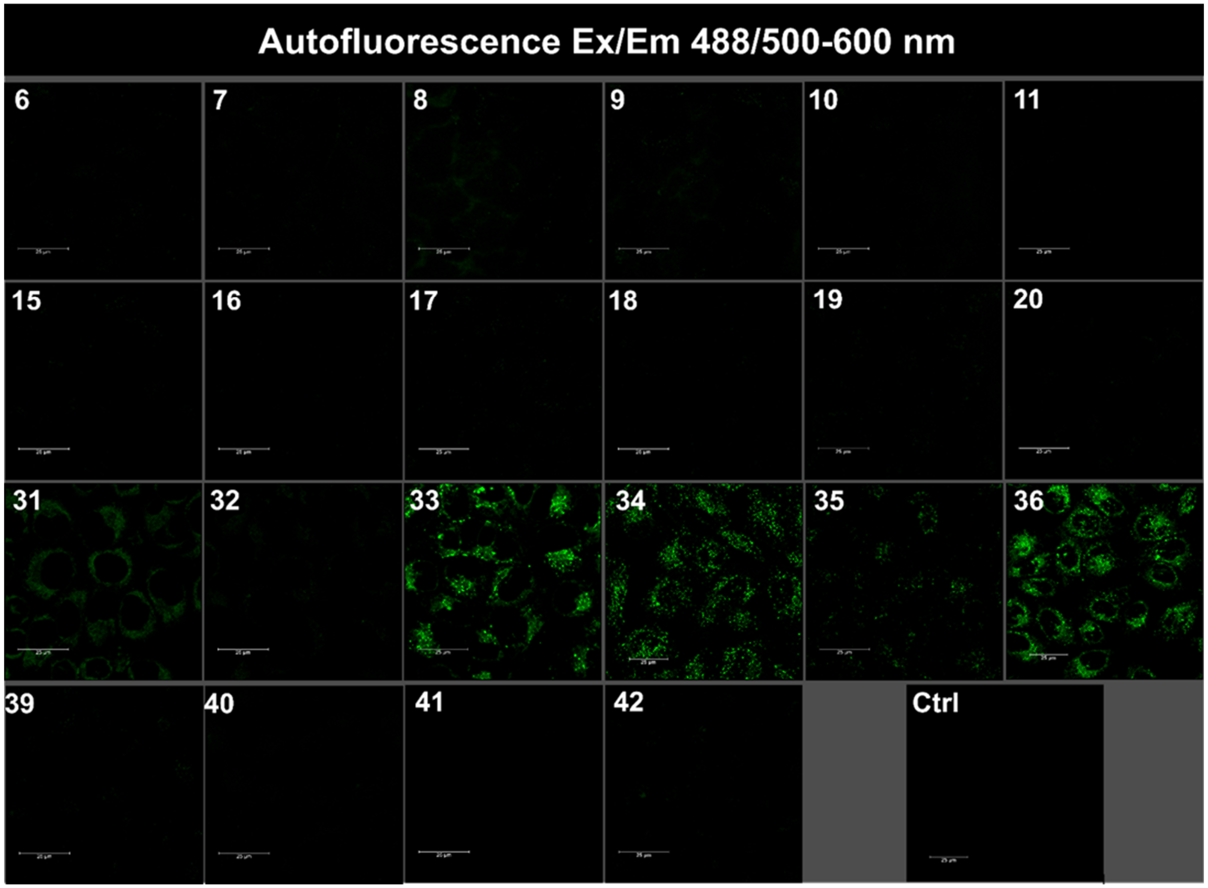


**Figure S161.** Autofluorescence analysis of the tested compounds in HepG2 cells**.** Confocal microscopy analysis was performed 8 h after treatment with the compounds in the final concentration corresponding to $IC_{50}$. The number of each compound is placed in the left corner of the picture. The intensity of the autofluorescence was analyzed at Ex/Em 488/500-600 nm.

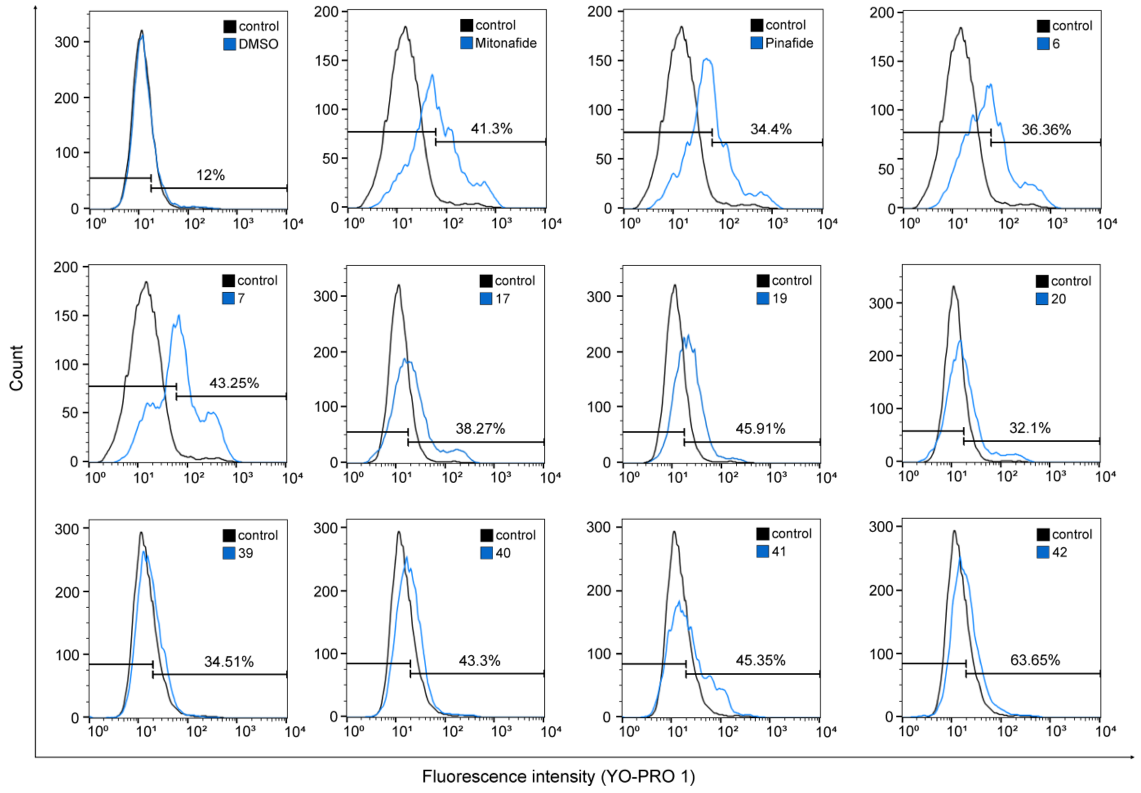


**Figure S162.** Flow cytometry analysis of apoptosis/necrosis in HepG2 cells after cell toxicity induction with compound **6** (115 µM), **7** (104 µM), **8** (4 µM), **9** (3 µM), **10** (8 µM), **11** (5 µM), **15** (68 µM), **16** (61 µM), **17** (10 µM), **18** (15 µM), **19** (14 µM), **20** (12 µM), **31** (53 µM), **32** (42 µM), **33** (5 µM), **34** (8 µM), **35** (9 µM), **36** (6 µM), **39** (11 µM), **40** (13 µM), **41** (10 µM), **42** (6 µM). The chosen concentration of each compound corresponded to whole $IC_{50}$ values. The number of apoptotic cells for each compound is indicated on histogram. Level of apoptosis was evaluated with dual staining with YO-PRO-1/PI.

**Table 1S**. Percentage distribution of early and late apoptotic and necrotic cells assessed by flow cytometry.

| compound | live cells [%] | early apoptosis [%] | late apoptosis [%] | necrosis [%] |
|---|---|---|---|---|
| **6** | 47.75 ± 4.78 | 1.56 ±0.26 | 28.80 ± 3.72 | 21.85 ± 1.12 |
| **7** | 25.90 ±0.34 | 4.55 ± 0.67 | 38.70 ± 1.72 | 30.85 ± 2.14 |
| **17** | 60.75 ± 4.49 | 30.70 ± 4.76 | 7.57 ± 0.98 | 0.98 ± 0.33 |
| **19** | 53.40 ± 3.59 | 39.25 ± 6.30 | 6.66 ± 2.55 | 0.72 ± 0.35 |
| **20** | 66.70 ± 2.86 | 18.75 ± 2.5 | 13.35 ± 2.69 | 1.23 ± 0.41 |
| **39** | 65.40 ± 0.88 | 31.65 ± 1.52 | 2.86 ± 1.05 | 0.08 ± 0.02 |
| **40** | 57.40 ± 1.9 | 32.35 ± 2.49 | 9.95 ± 2.17 | 0.35 ± 0.21 |
| **41** | 54.35 ± 3.82 | 26.75 ± 2.17 | 18.60 ± 3.01 | 0.34 ± 0.07 |
| **42** | 36.10 ± 3.8 | 24.65 ± 1.78 | 39.00 ± 5.33 | 0.24 ± 0.04 |
| Mitonafide | 37.25 ± 1.09 | 1.90 ± 0.19 | 39.40 ± 1.94 | 21.40 ± 3.13 |
| Pinafide | 47.10 ± 1.01 | 1.40 ± 0.57 | 33.00 ± 1.19 | 18.50 ± 1.72 |

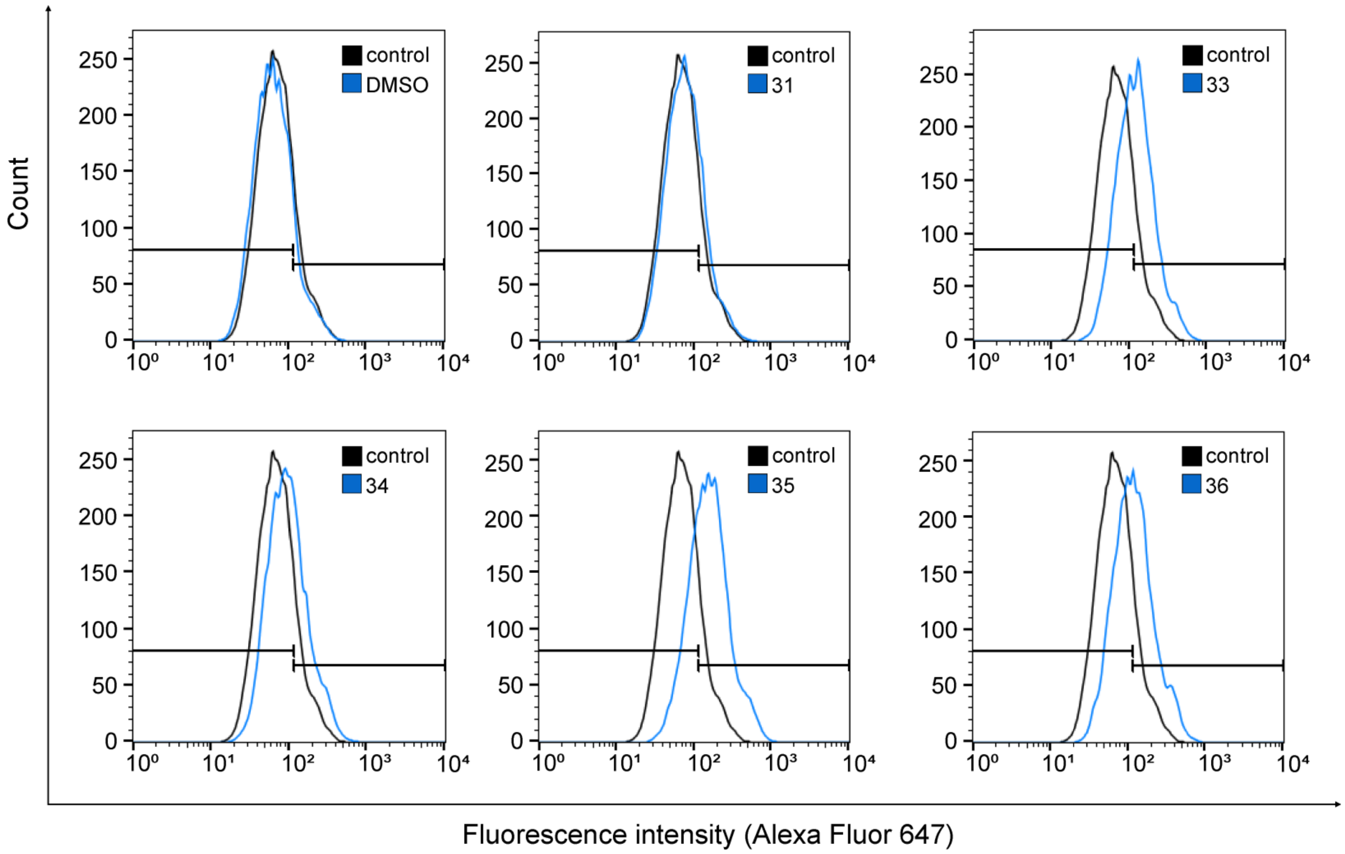


**Figure S163.** Flow cytometry analysis of apoptosis/necrosis in HepG2 cells after cell toxicity induction with compound **31** (53 μM), **33** (5 μM), **34** (8 μM), **35** (9 μM), **36** (6 μM). The chosen concentration of each compound corresponded to whole $IC_{50}$ values. The number of apoptotic cells for each compound is indicated on histogram. Level of apoptosis was evaluated with Annexin V Alexa Fluor 647 conjugate.

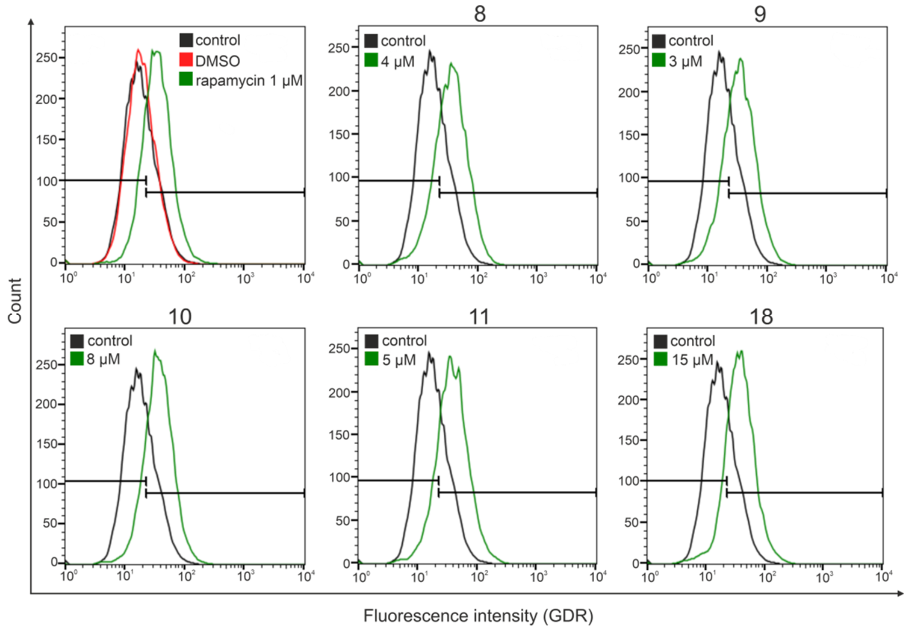


**Figure S164**. Flow cytometry analysis of autophagy in HepG2 cells after cell toxicity induction with compound **8** (4 µM), **9** (3 µM), **10** (8 µM), **11** (5 µM), **18** (15 µM). The chosen concentration of each compound corresponded to whole $IC_{50}$ values. Level of autophagy was evaluated with Green Detection Reagent. Rapamycin was used as positive control. One representative experiment of three is shown.

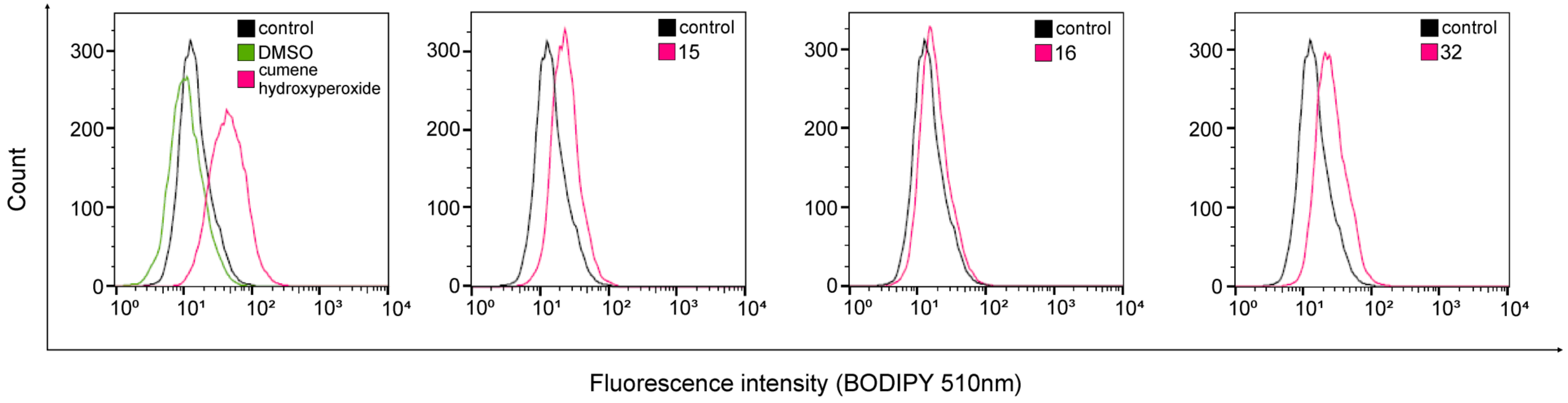


**Figure S165**. Flow cytometry analysis of lipid peroxidation in HepG2 cells after cell toxicity induction with compound **15** (68 μM), **16** (61 μM), **32** (42 μM). The chosen concentration of each compound corresponded to whole $IC_{50}$ values. Level of lipid peroxidation was evaluated with 581/591 C11 reagent. To induce strong positive signal the cells were incubated with cumene peroxide. One representative experiment of three is shown.

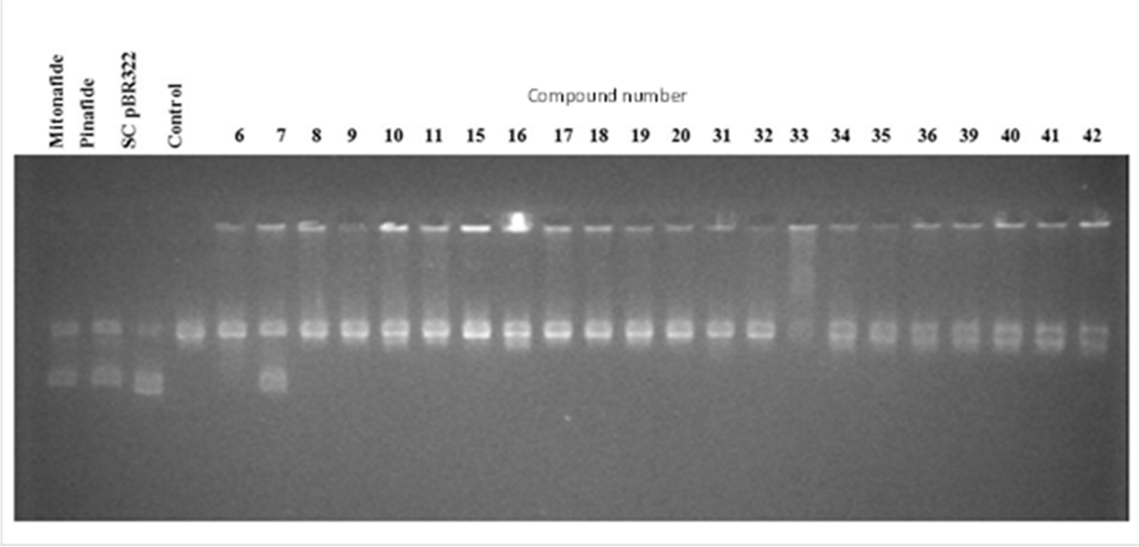


**Figure S166**. Human Topoisomerase II$\alpha$ relaxation assay in the presence of modified with carborane cluster naphthalic anhydrides (**6**, **7**, **15**, **16**, **31**, **32**), naphthalimides (**8**-**11**, **17**-**20**, **33**-**36**, **39**-**42**), and the control drugs mitonafide and pinafide at a concentration of 100 μM.
SC – supercoiled DNA.

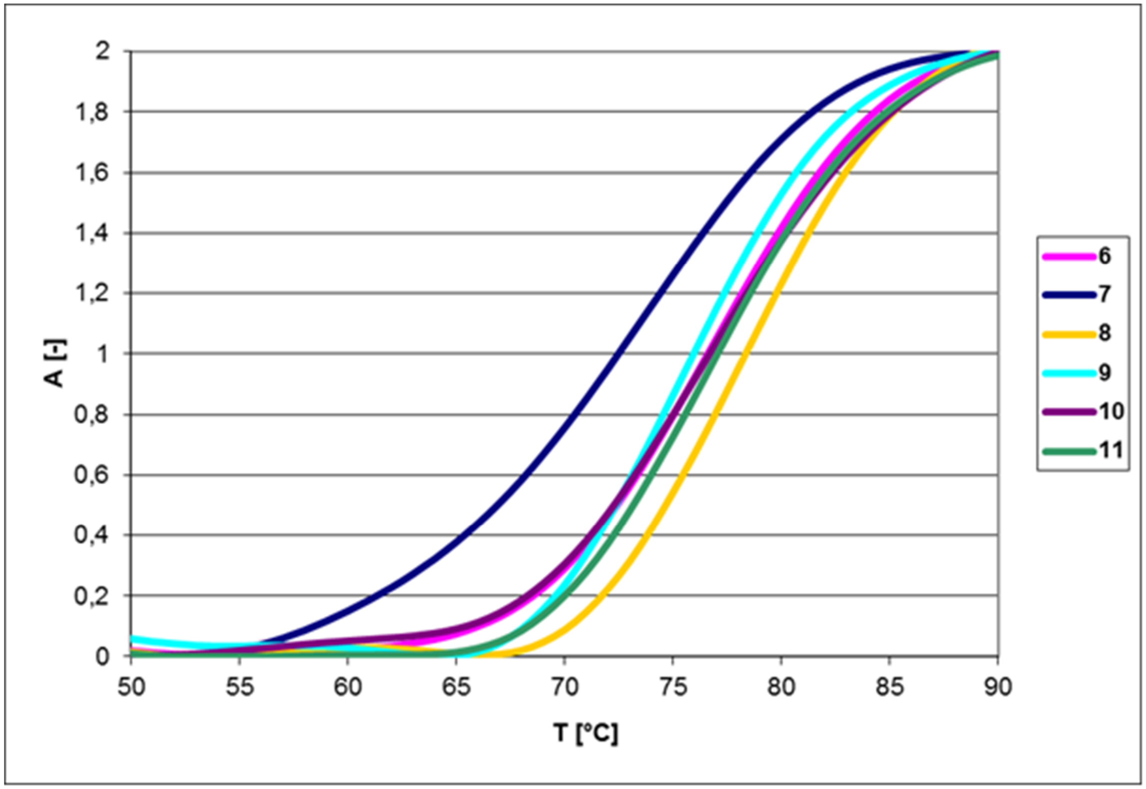


**Figure S167**. Melting curves of ct-DNA upon addition of **6-11** (c (ct-DNA) = 2 × $10^{-5}$ mol $dm^{-3}$) at molar ratio r = 0.3 (r = [compound]/[ct-DNA]), sodium cacodylate buffer (pH 7.0, 20 mM).

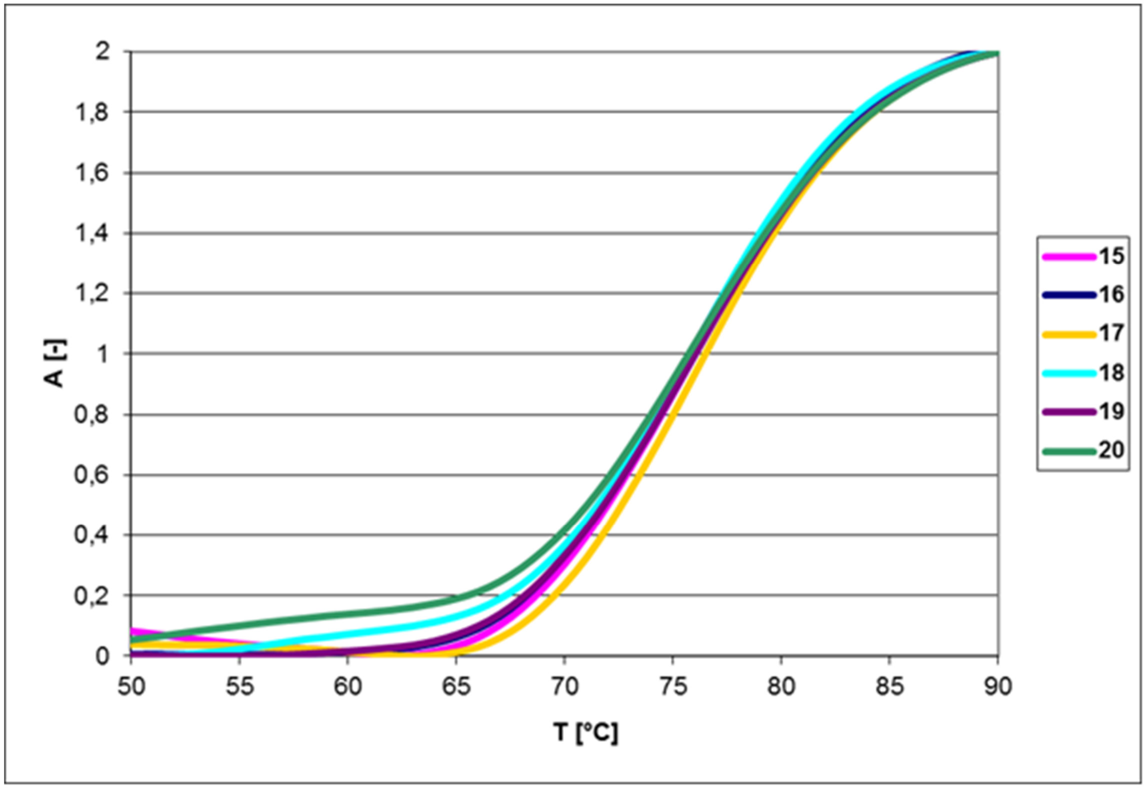


**Figure S168**. Melting curves of ct-DNA upon addition of **15**-**20** (c (ct-DNA) = 2 × $10^{-5}$ mol $dm^{-3}$) at molar ratio r = 0.3 (r = [compound]/[ct-DNA]), sodium cacodylate buffer (pH 7.0, 20 mM).

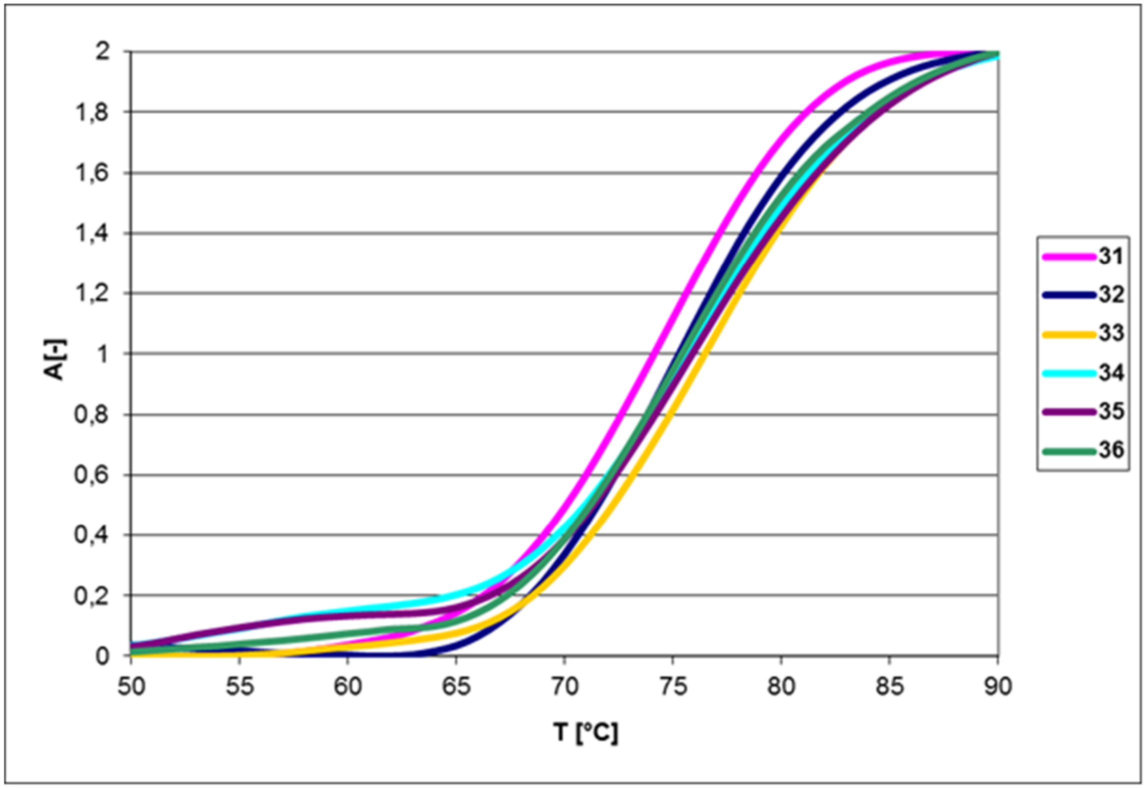


**Figure S169**. Melting curves of ct-DNA upon addition of **31**-**36** (c (ct-DNA) = 2 × $10^{-5}$ mol $dm^{-3}$) at molar ratio r = 0.3 (r = [compound]/[ct-DNA]), sodium cacodylate buffer (pH 7.0, 20 mM).

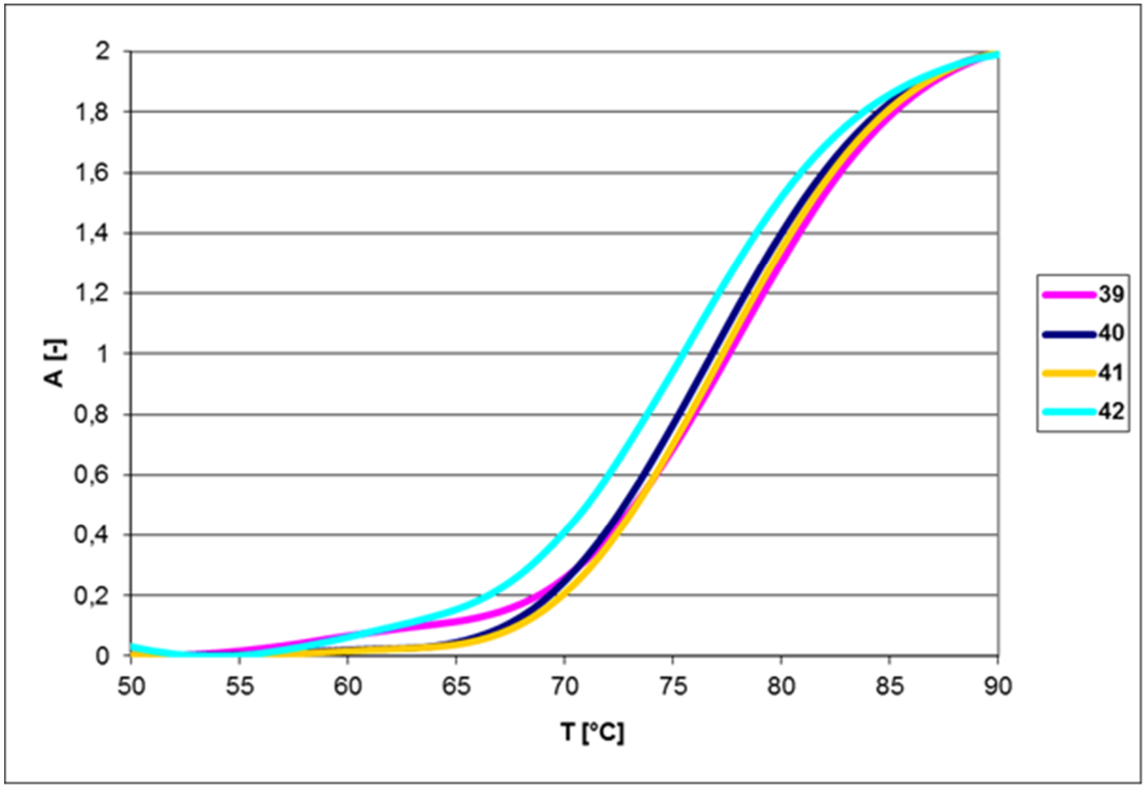


**Figure S170**. Melting curves of ct-DNA upon addition of **39**-**42** (c (ct-DNA) = 2 × $10^{-5}$ mol $dm^{-3}$) at molar ratio r = 0.3 (r = [compound]/[ct-DNA]), sodium cacodylate buffer (pH 7.0, 20 mM).

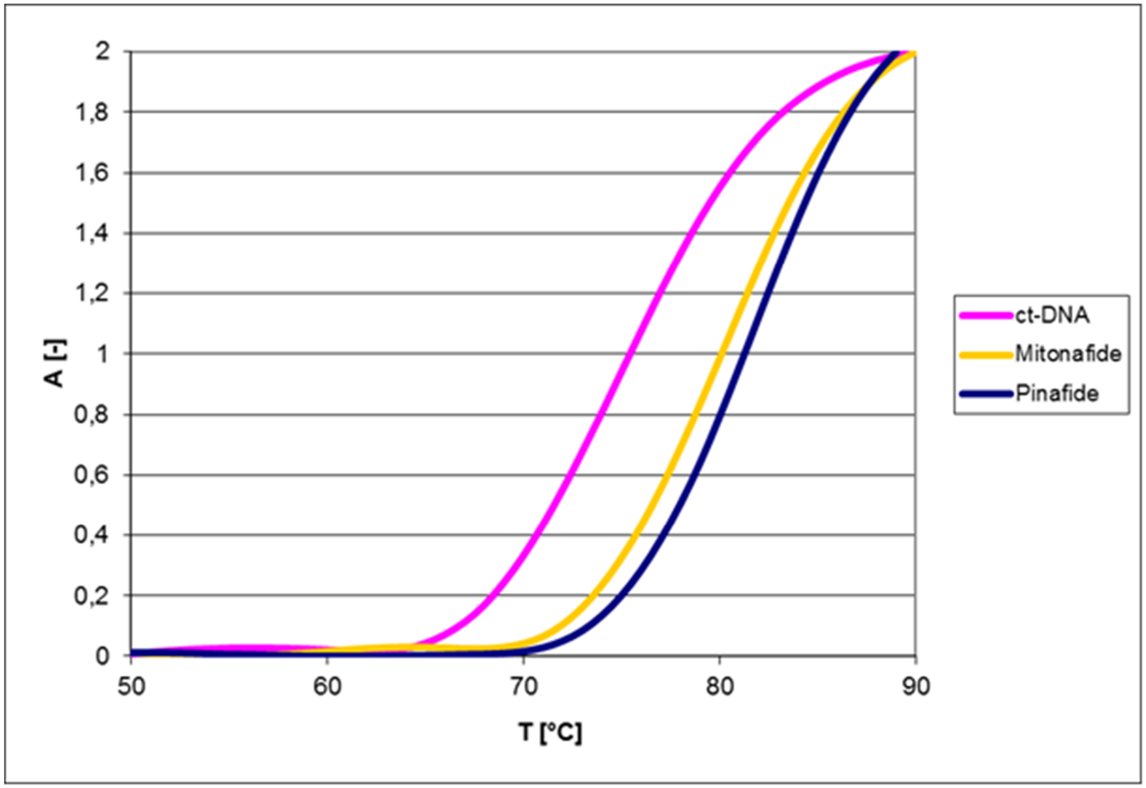


**Figure S171**. Melting curves of ct-DNA upon addition of mitonafide and pinafide (c (ct-DNA) = $2 \times 10^{-5}$ mol $dm^{-3}$) at molar ratio r = 0.3 (r = [compound]/[ct-DNA]), sodium cacodylate buffer (pH 7.0, 20 mM).

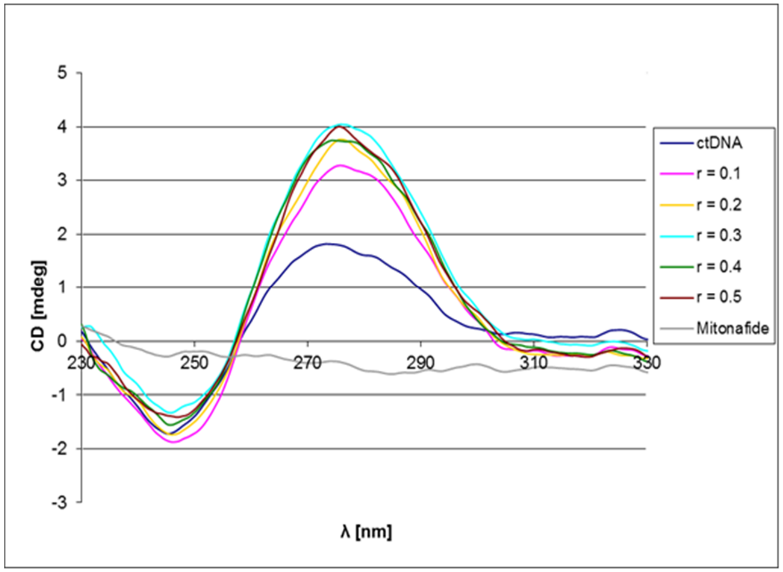


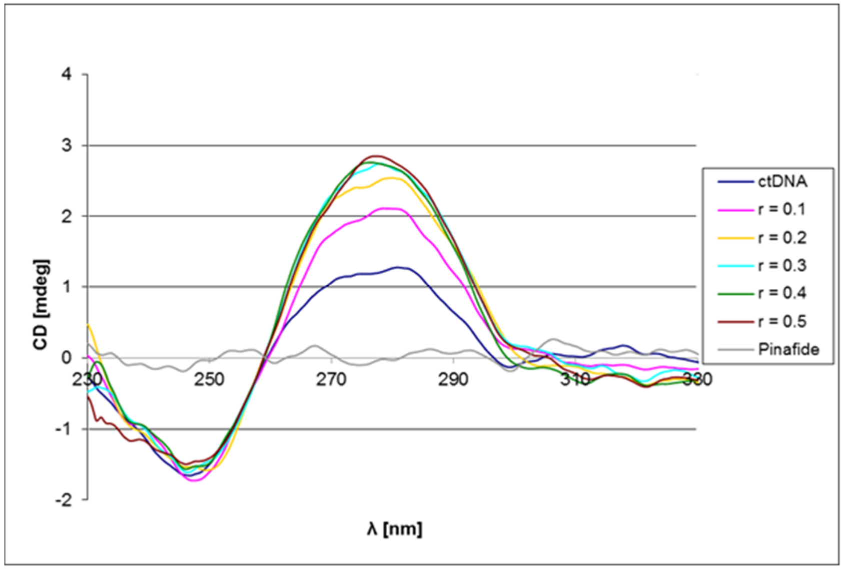


**Figure S172**. Changes in the CD spectrum of ct-DNA upon addition of mitonafide (left) and pinafide (right) (c (ct-DNA) = $4 \times 10^{-5}$ mol dm$^{-3}$) at different molar ratios r = [compound]/[ct-DNA], sodium cacodylate buffer (pH 7.0, 20 mM).

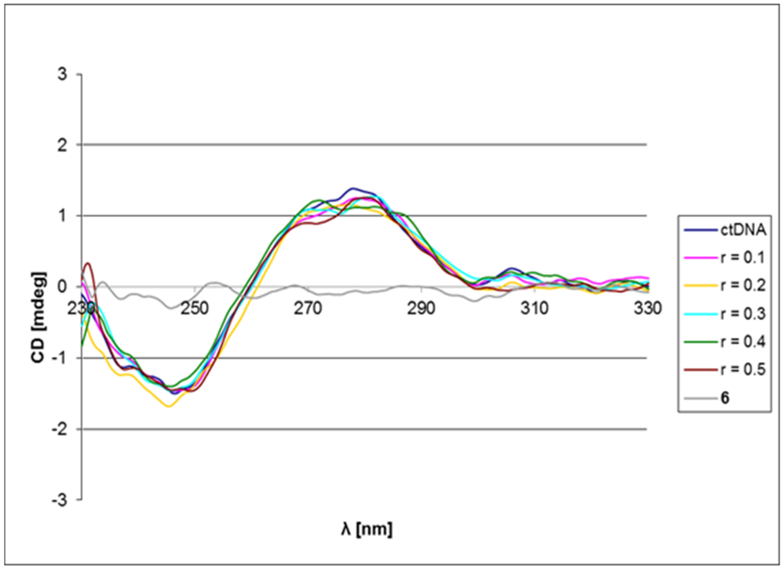


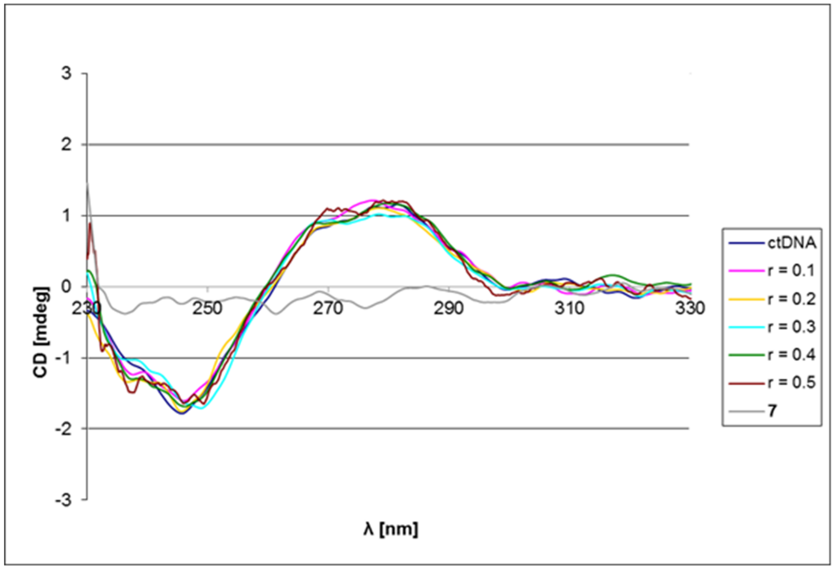


**Figure S173**. Changes in the CD spectrum of ct-DNA upon addition of **6** (c (ct-DNA) = 4 × $10^{-5}$ mol $dm^{-3}$) (left) and **7** (c (ct-DNA) = 4 × $10^{-5}$ mol $dm^{-3}$) (right) at different molar ratios r = [compound]/[ct-DNA], sodium cacodylate buffer (pH 7.0, 20 mM).

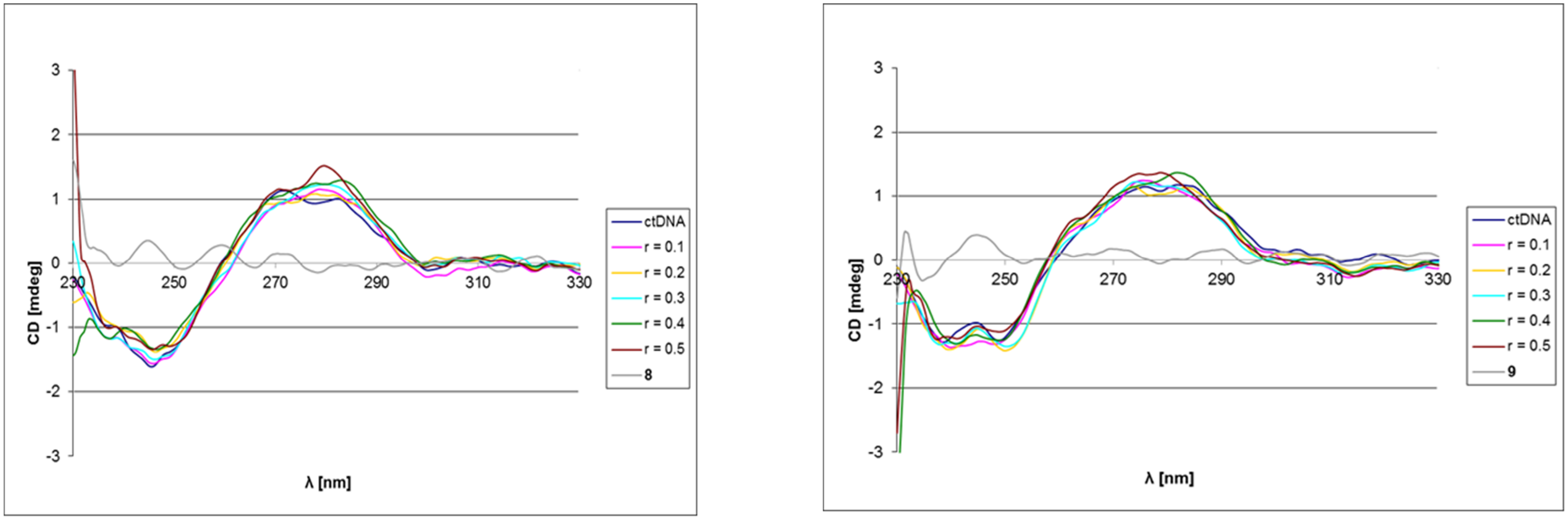


**Figure S174**. Changes in the CD spectrum of ct-DNA upon addition of **8** (c (ct-DNA) = 4 × $10^{-5}$ mol $dm^{-3}$) (left) and **9** (c (ct-DNA) = 4 × $10^{-5}$ mol $dm^{-3}$) (right) at different molar ratios r = [compound]/[ct-DNA], sodium cacodylate buffer (pH 7.0, 20 mM).

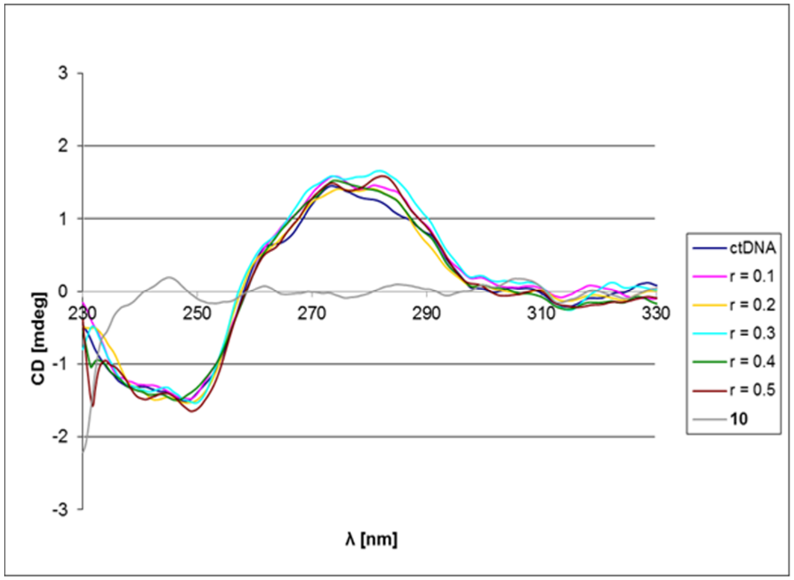


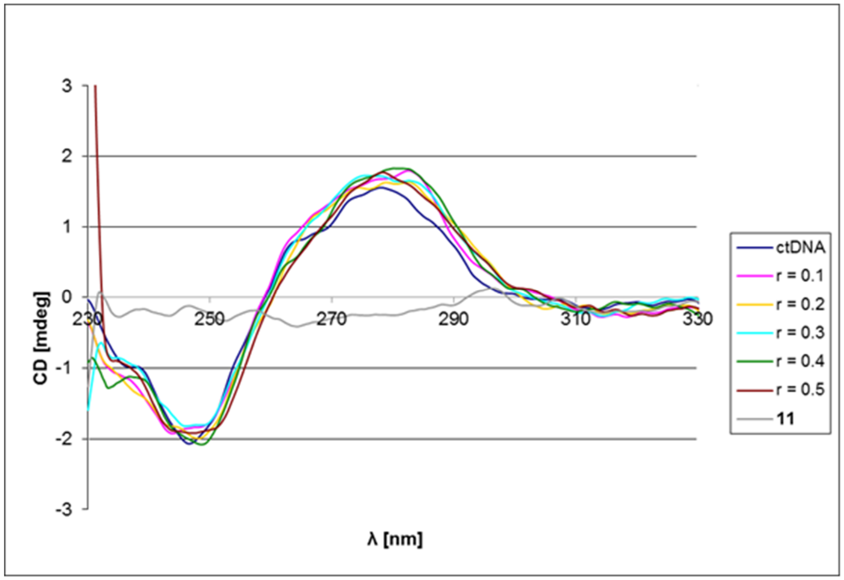


**Figure S175**. Changes in the CD spectrum of ct-DNA upon addition of **10** (c (ct-DNA) = $4 \times 10^{-5}$ mol dm$^{-3}$) (left) and **11** (c (ct-DNA) = $4 \times 10^{-5}$ mol dm$^{-3}$) (right) at different molar ratios r = [compound]/[ct-DNA], sodium cacodylate buffer (pH 7.0, 20 mM).

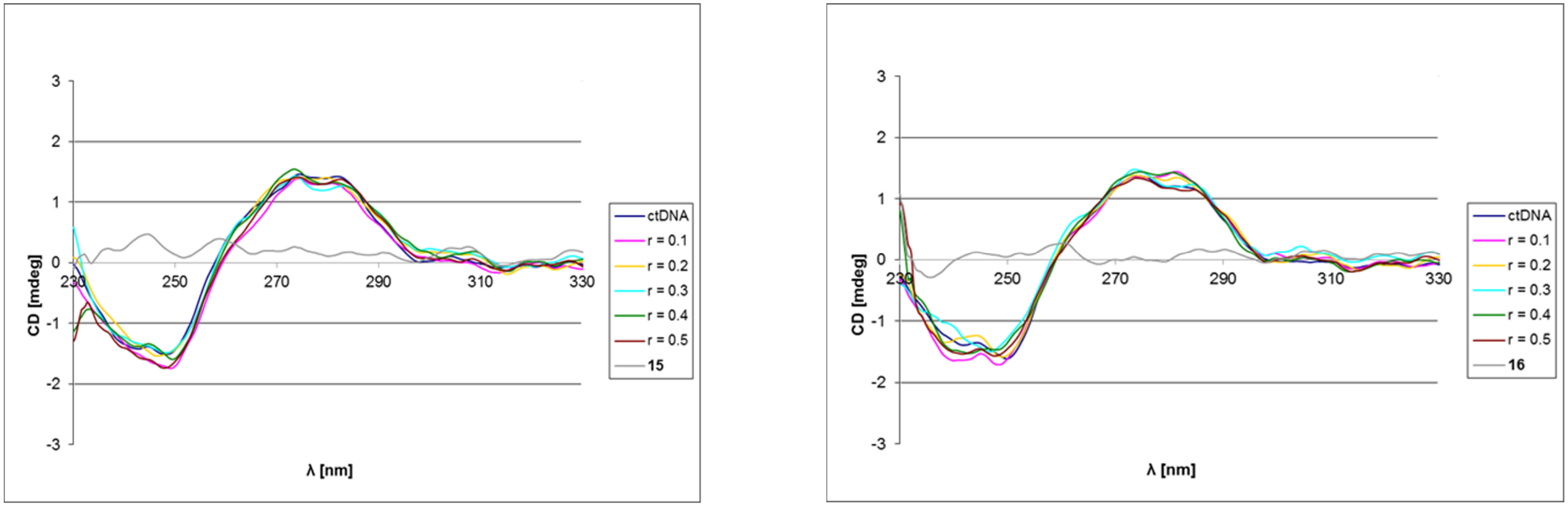


**Figure S176**. Changes in the CD spectrum of ct-DNA upon addition of **15** (c (ct-DNA) = $4 \times 10^{-5}$ mol $dm^{-3}$) (left) and **16** (c (ct-DNA) = $4 \times 10^{-5}$ mol $dm^{-3}$) (right) at different molar ratios r = [compound]/[ct-DNA], sodium cacodylate buffer (pH 7.0, 20 mM).

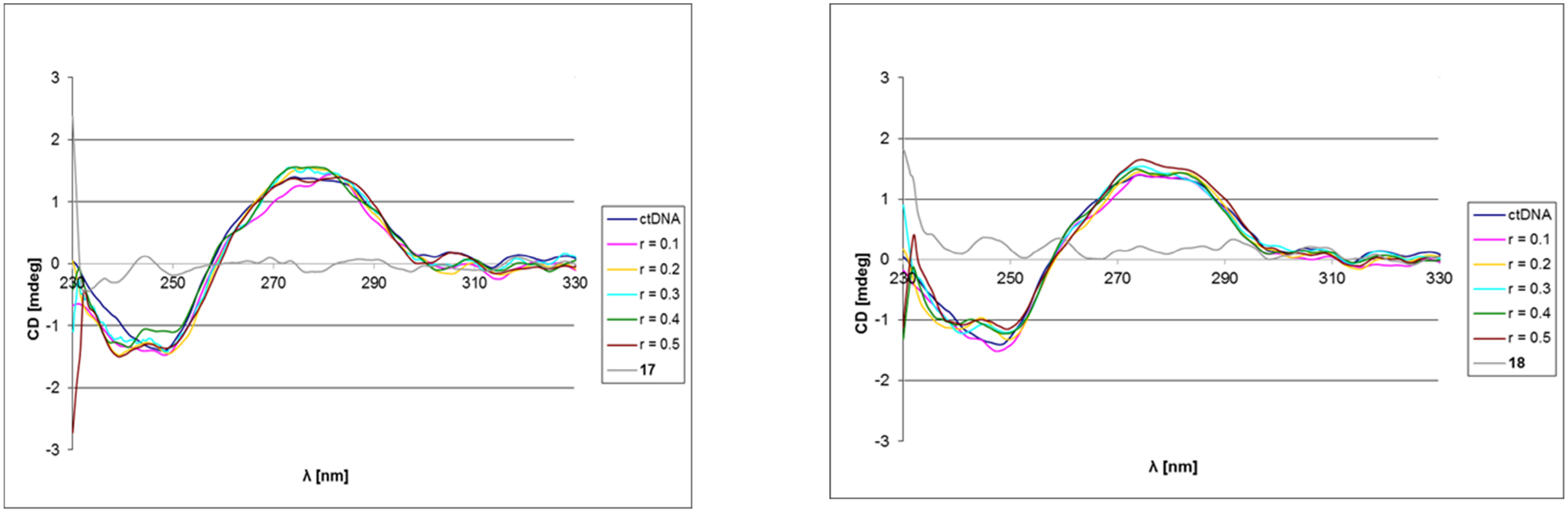


**Figure S177**. Changes in the CD spectrum of ct-DNA upon addition of **17** (c (ct-DNA) = $4 \times 10^{-5}$ mol dm$^{-3}$) (left) and **18** (c (ct-DNA) = $4 \times 10^{-5}$ mol dm$^{-3}$) (right) at different molar ratios r = [compound]/[ct-DNA], sodium cacodylate buffer (pH 7.0, 20 mM).

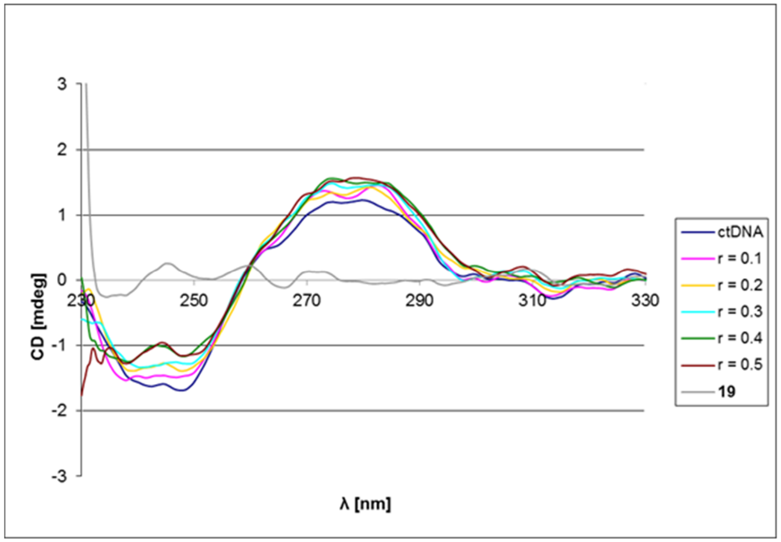


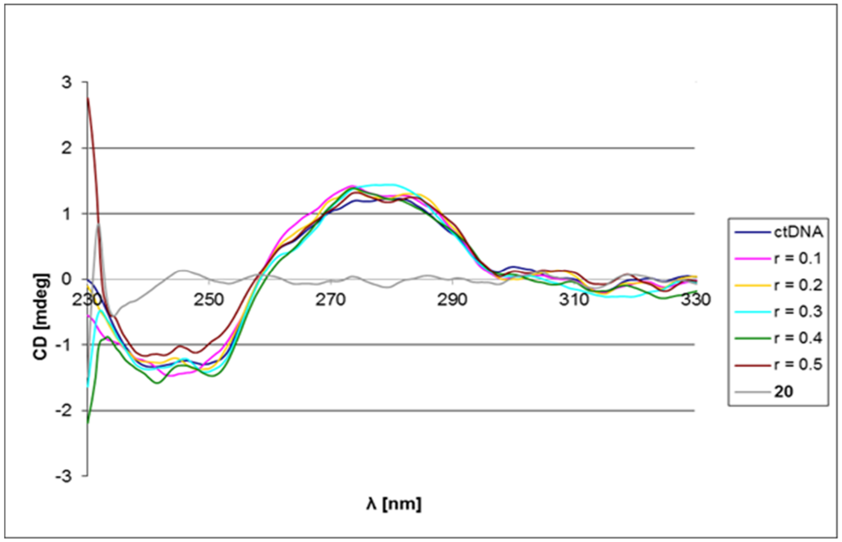


**Figure S178**. Changes in the CD spectrum of ct-DNA upon addition of **19** (c (ct-DNA) = 4 × $10^{-5}$ mol $dm^{-3}$) (left) and **20** (c (ct-DNA) = 4 × $10^{-5}$ mol $dm^{-3}$) (right) at different molar ratios r = [compound]/[ct-DNA], sodium cacodylate buffer (pH 7.0, 20 mM).

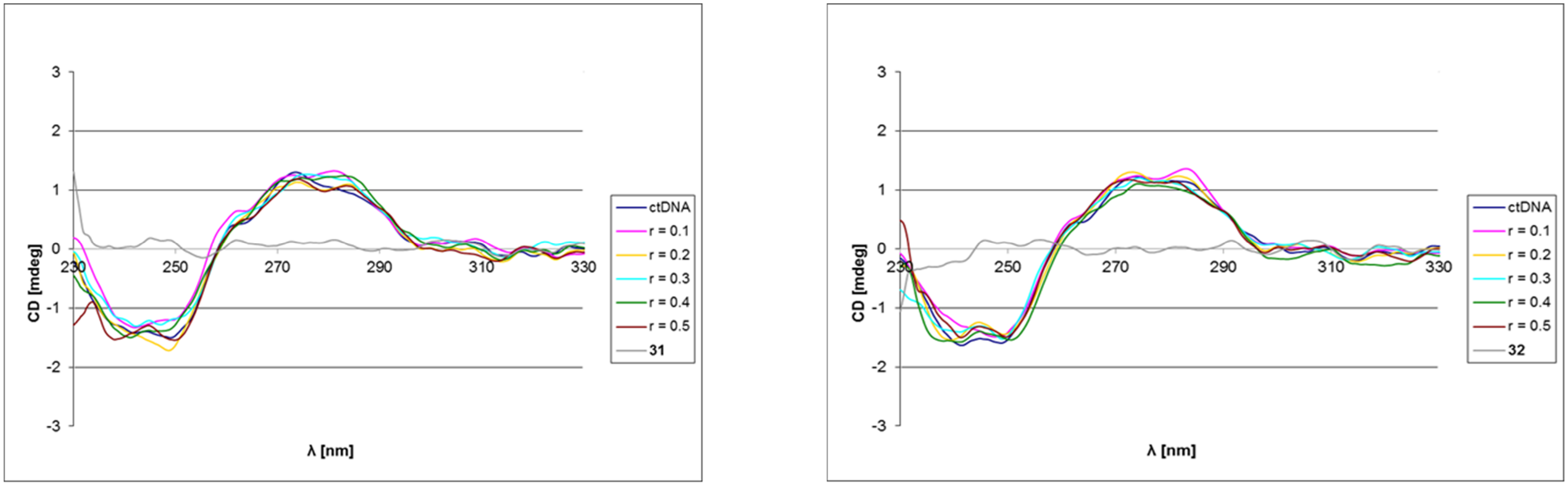


**Figure S179**. Changes in the CD spectrum of ct-DNA upon addition of **31** (c (ct-DNA) = 4 × $10^{-5}$ mol $dm^{-3}$) (left) and **32** (c (ct-DNA) = 4 × $10^{-5}$ mol $dm^{-3}$) (right) at different molar ratios r = [compound]/[ct-DNA], sodium cacodylate buffer (pH 7.0, 20 mM).

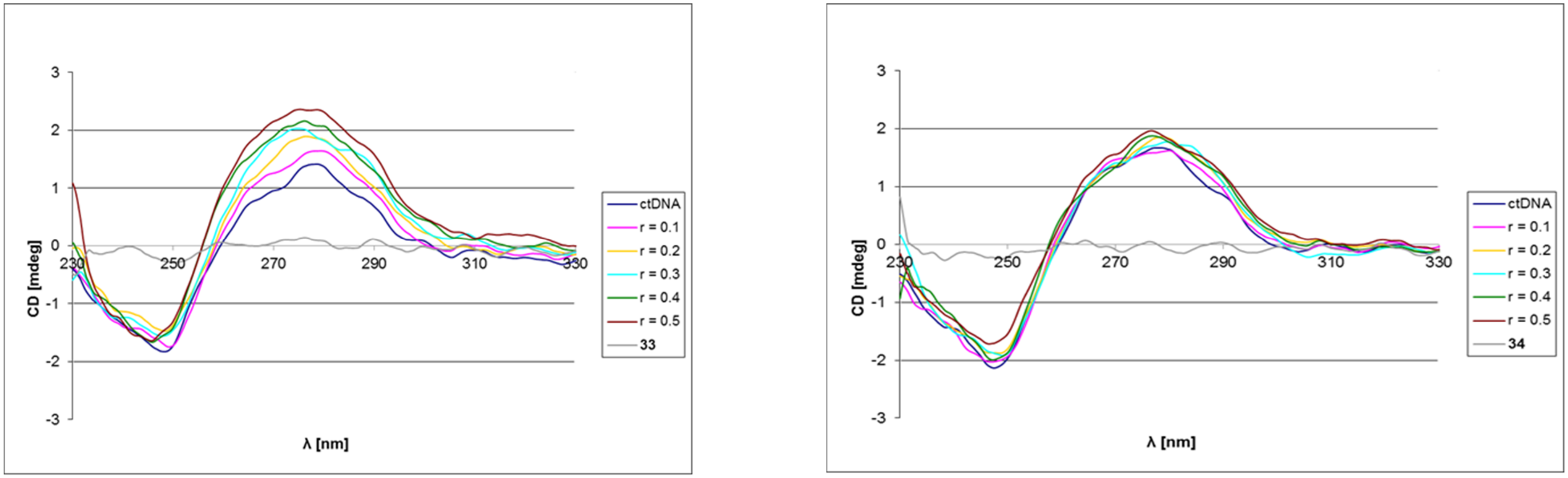


**Figure S180**. Changes in the CD spectrum of ct-DNA upon addition of **33** (c (ct-DNA) = 4 × $10^{-5}$ mol $dm^{-3}$) (left) and **34** (c (ct-DNA) = 4 × $10^{-5}$ mol $dm^{-3}$) (right) at different molar ratios r = [compound]/[ct-DNA], sodium cacodylate buffer (pH 7.0, 20 mM).

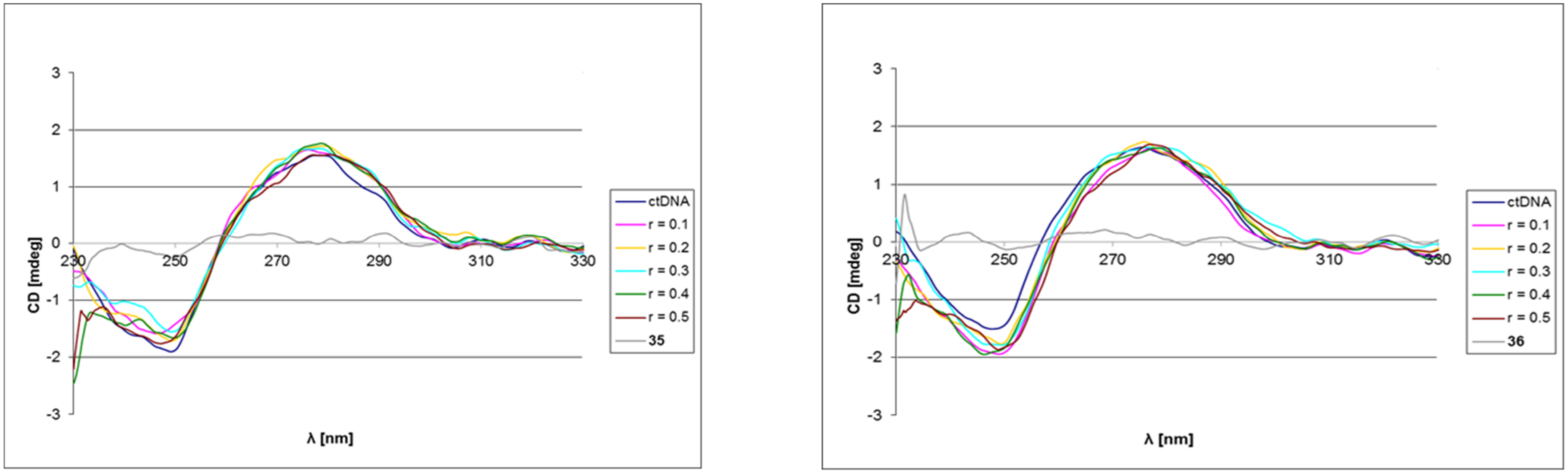


**Figure S181**. Changes in the CD spectrum of ct-DNA upon addition of **35** (c (ct-DNA) = 4 × $10^{-5}$ mol $dm^{-3}$) (left) and **36** (c (ct-DNA) = 4 × $10^{-5}$ mol $dm^{-3}$) (right) at different molar ratios r = [compound]/[ct-DNA], sodium cacodylate buffer (pH 7.0, 20 mM).

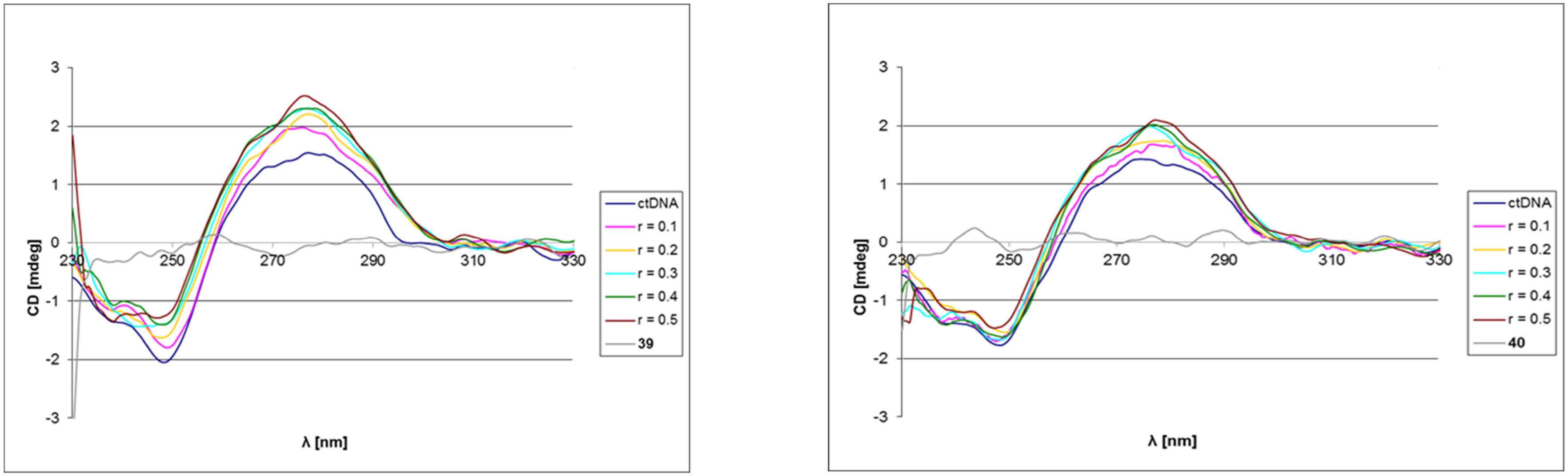


**Figure S182**. Changes in the CD spectrum of ct-DNA upon addition of **39** (c (ct-DNA) = 4 × $10^{-5}$ mol $dm^{-3}$) (left) and **40** (c (ct-DNA) = 4 × $10^{-5}$ mol $dm^{-3}$) (right) at different molar ratios r = [compound]/[ct-DNA], sodium cacodylate buffer (pH 7.0, 20 mM).

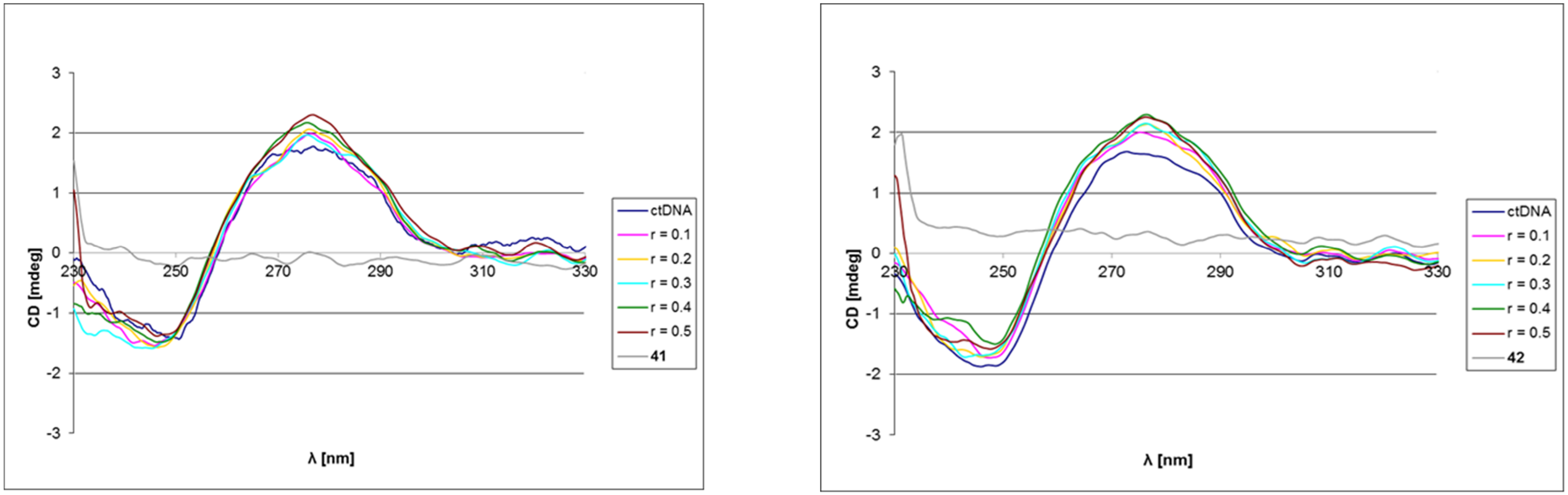


**Figure S183**. Changes in the CD spectrum of ct-DNA upon addition of **41** (c (ct-DNA) = 4 × $10^{-5}$ mol $dm^{-3}$) (left) and **42** (c (ct-DNA) = 4 × $10^{-5}$ mol $dm^{-3}$) (right) at different molar ratios r = [compound]/[ct-DNA], sodium cacodylate buffer (pH 7.0, 20 mM).

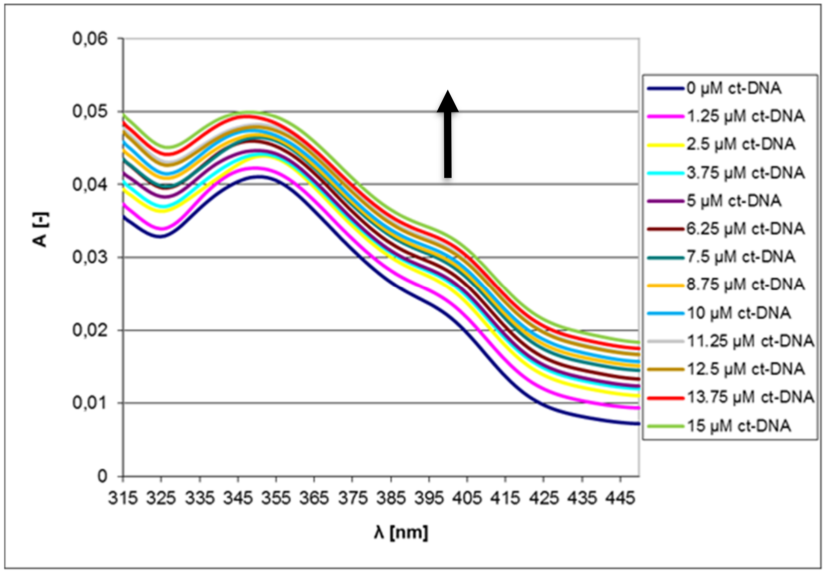


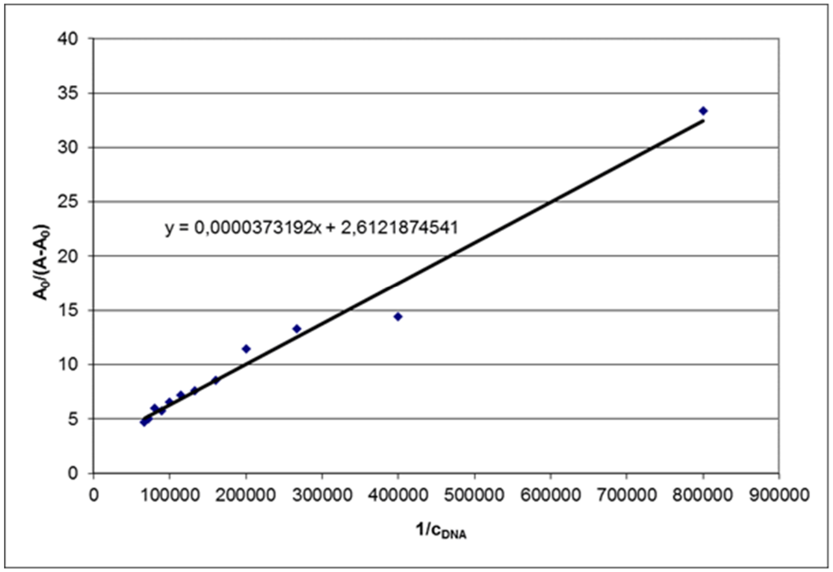


**Figure S184**. UV-vis absorption spectra of compound **6** (20 μM) in the presence of increasing amount of cf-DNA (0-15 μM) (left). The plot of $A_0/A-A_0$ versus 1/[DNA] yielded the binding constant (right).

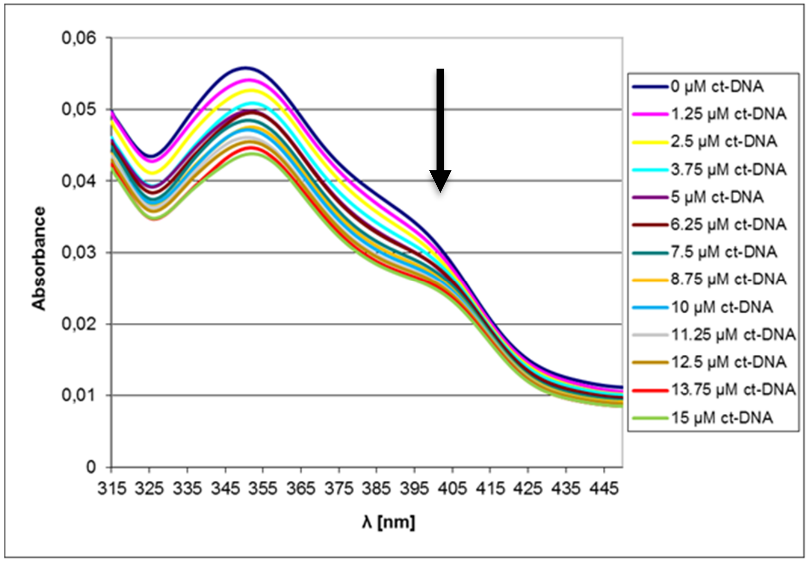


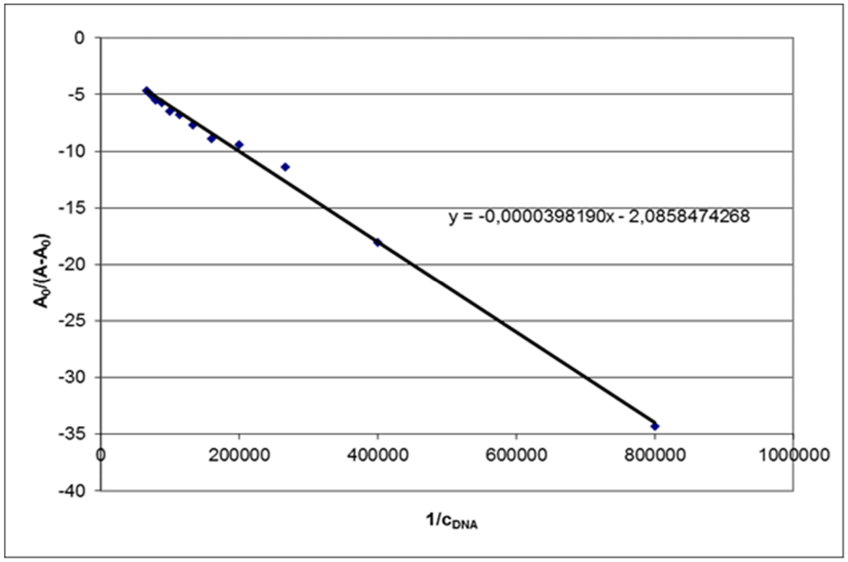


**Figure S185**. UV-vis absorption spectra of compound **7** (20 μM) in the presence of increasing amount of cf-DNA (0-15 μM) (left). The plot of $A_0/A-A_0$ versus 1/[DNA] yielded the binding constant (right).

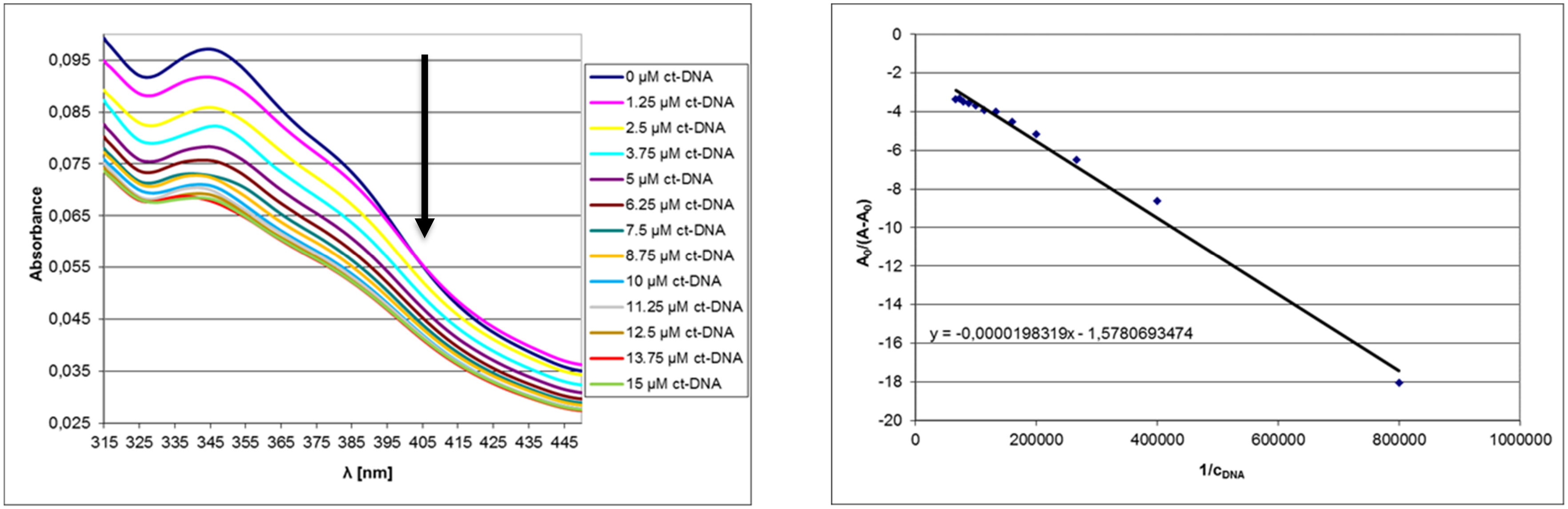


**Figure S186**. UV-vis absorption spectra of compound **8** (20 µM) in the presence of increasing amount of ct-DNA (0-15 µM) (left). The plot of $A_0/A-A_0$ versus 1/[DNA] yielded the binding constant (right).

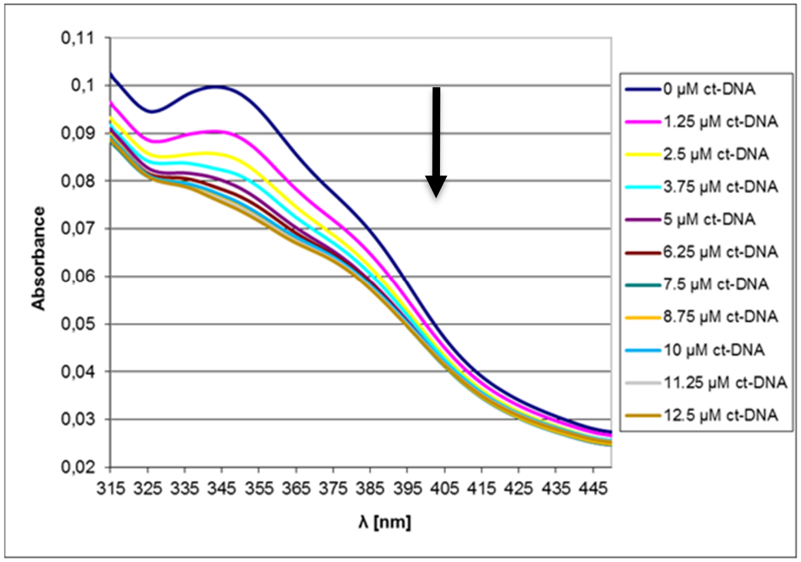


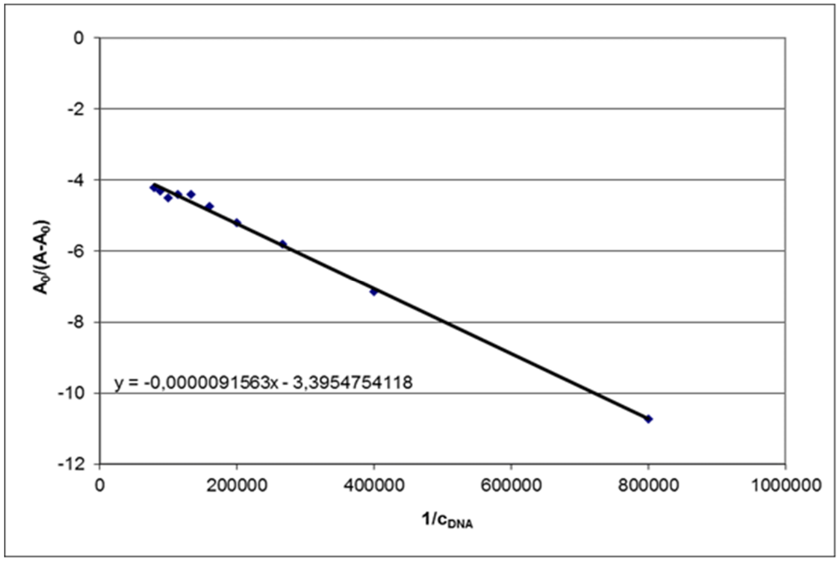


**Figure S187**. UV-vis absorption spectra of compound **9** (20 µM) in the presence of increasing amount of ct-DNA (0-15 µM) (left). The plot of $A_0/A-A_0$ versus 1/[DNA] yielded the binding constant (right).

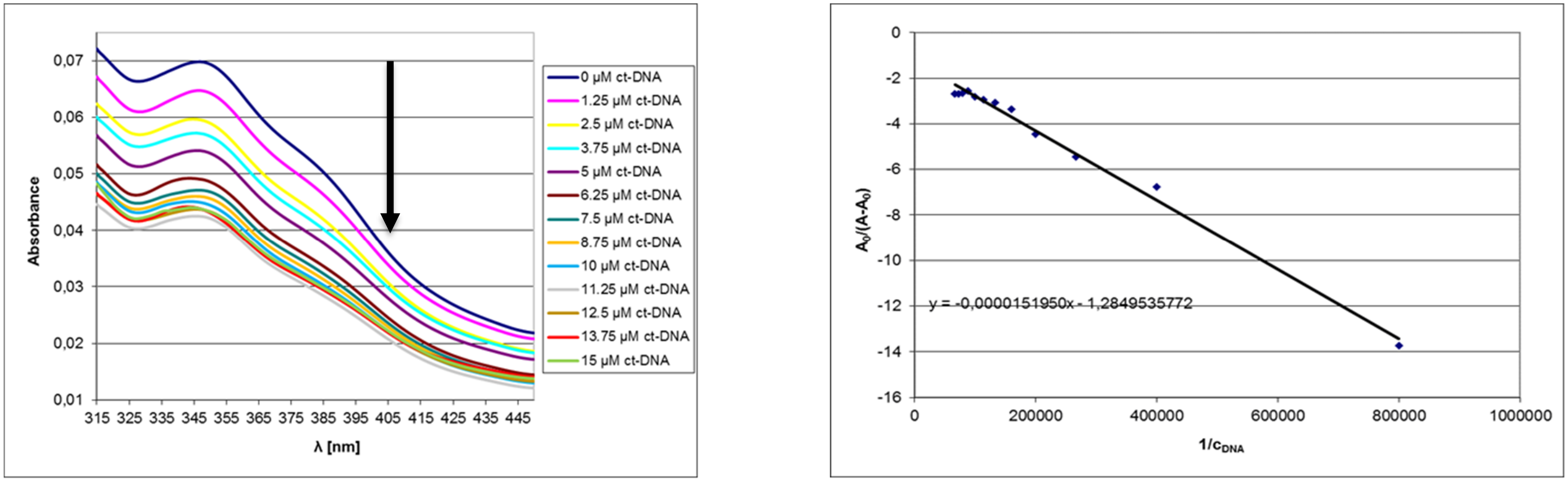


**Figure S188**. UV-vis absorption spectra of compound **10** (20 μM) in the presence of increasing amount of ct-DNA (0-15 μM) (left). The plot of $A_0/A-A_0$ versus 1/[DNA] yielded the binding constant (right).

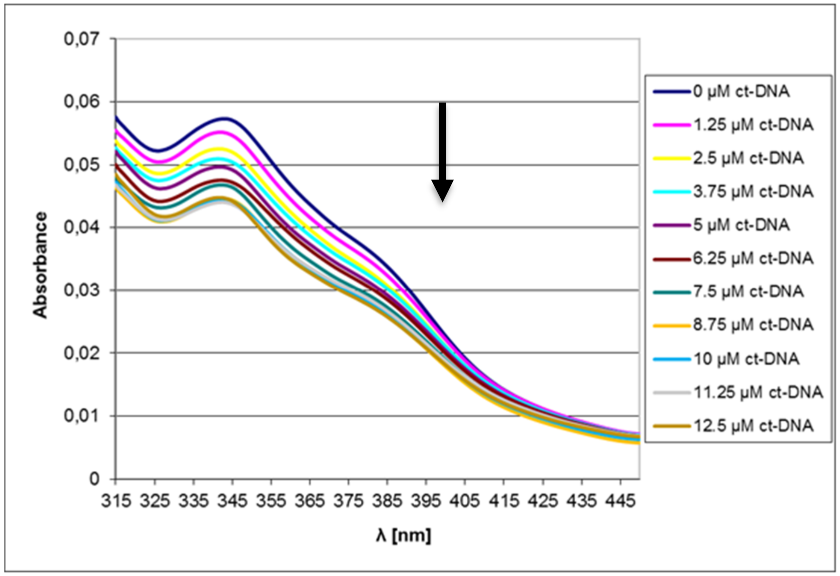


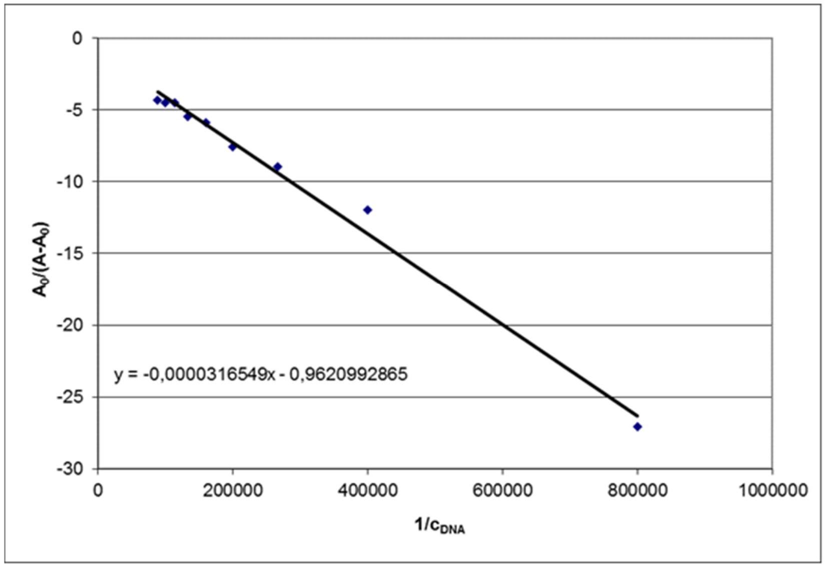


**Figure S189**. UV-vis absorption spectra of compound **11** (20 µM) in the presence of increasing amount of ct-DNA (0-15 µM) (left). The plot of $A_0/A-A_0$ versus 1/[DNA] yielded the binding constant (right).

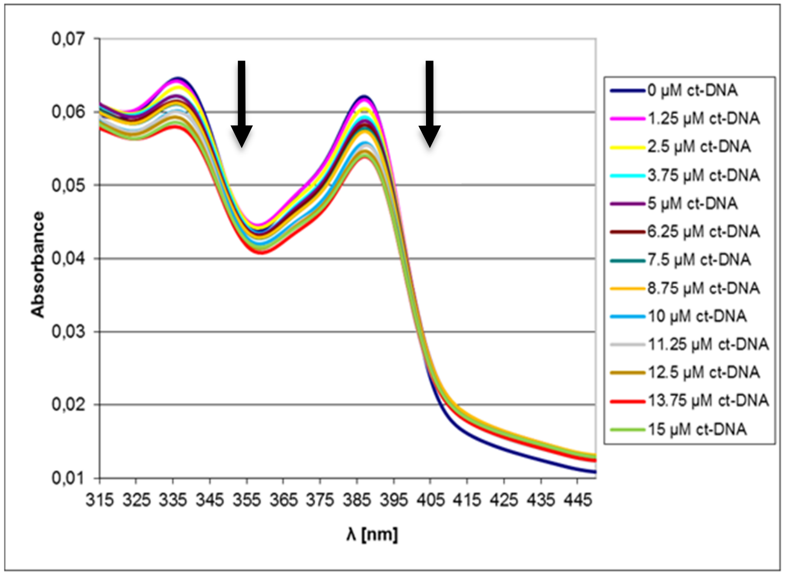


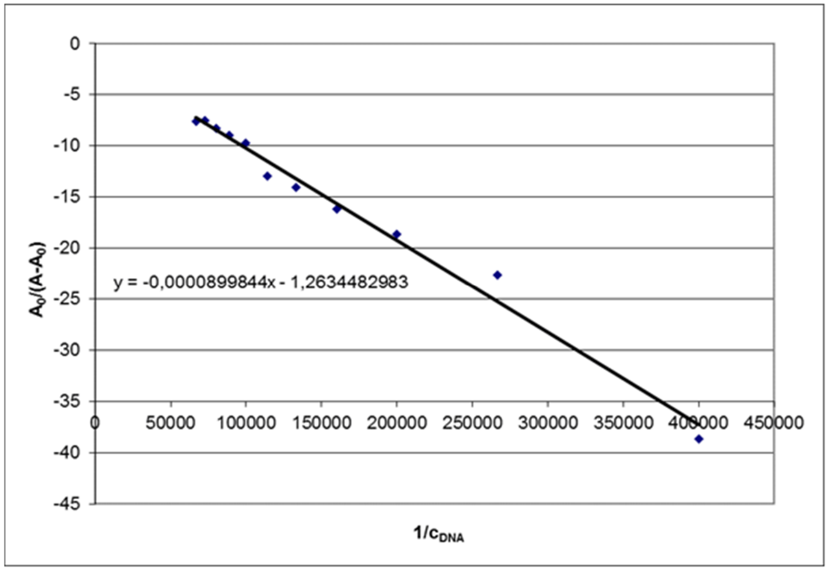


**Figure S190**. UV-vis absorption spectra of compound **15** (20 µM) in the presence of increasing amount of ct-DNA (0-15 µM) (left). The plot of $A_0/A-A_0$ versus 1/[DNA] yielded the binding constant (right).

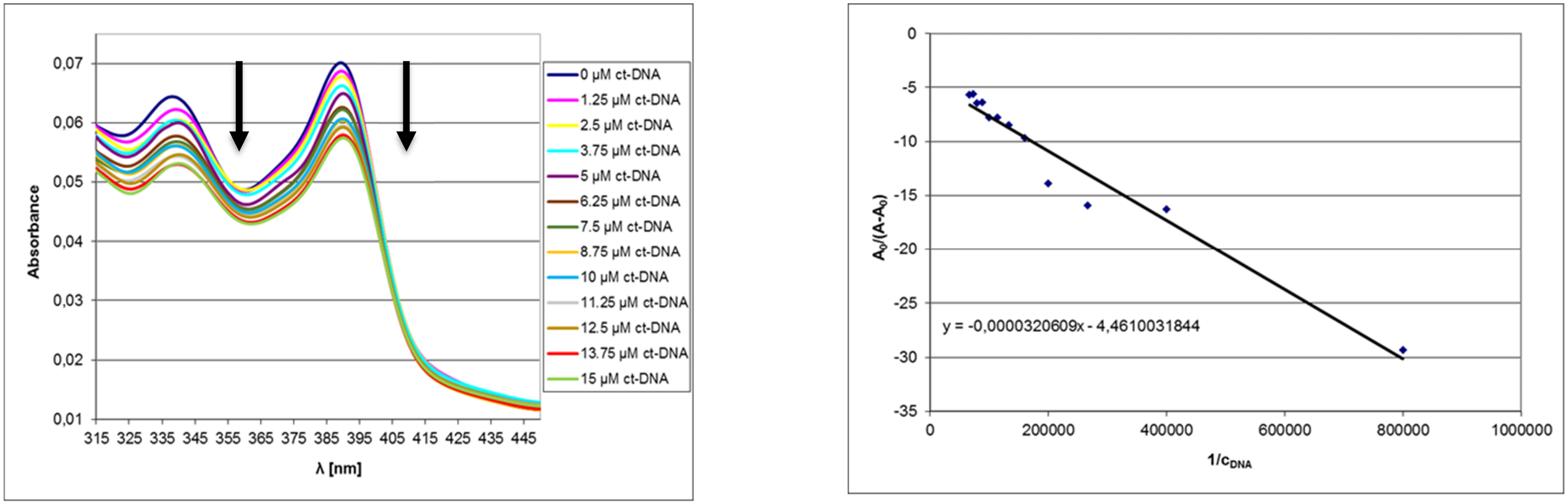


**Figure S191**. UV-vis absorption spectra of compound **16** (20 μM) in the presence of increasing amount of ct-DNA (0-15 μM) (left). The plot of $A_0/A-A_0$ versus 1/[DNA] yielded the binding constant (right).

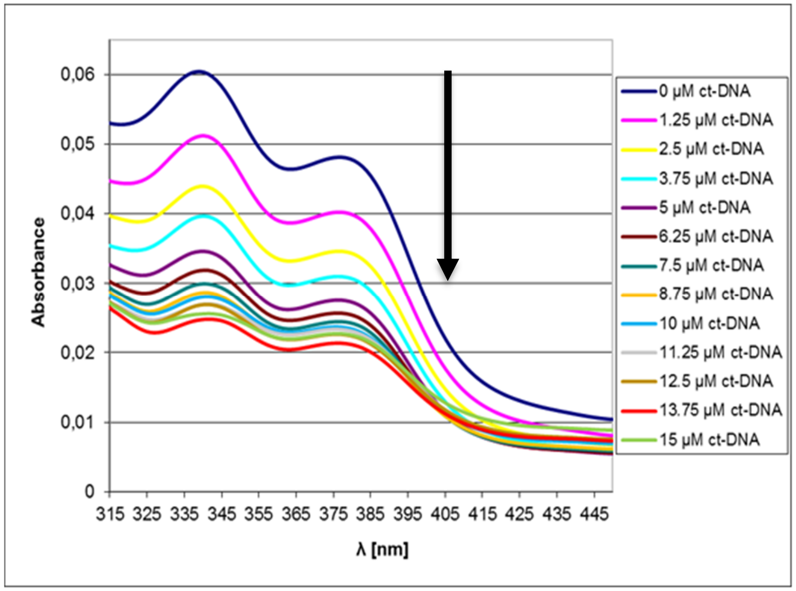


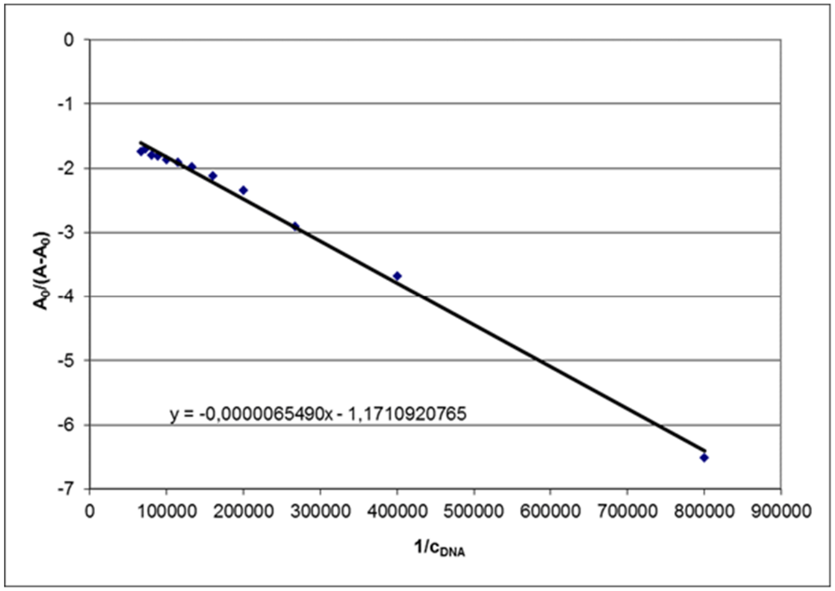


**Figure S192**. UV-vis absorption spectra of compound **17** (20 μM) in the presence of increasing amount of ct-DNA (0-15 μM) (left). The plot of $A_0/A-A_0$ versus 1/[DNA] yielded the binding constant (right).

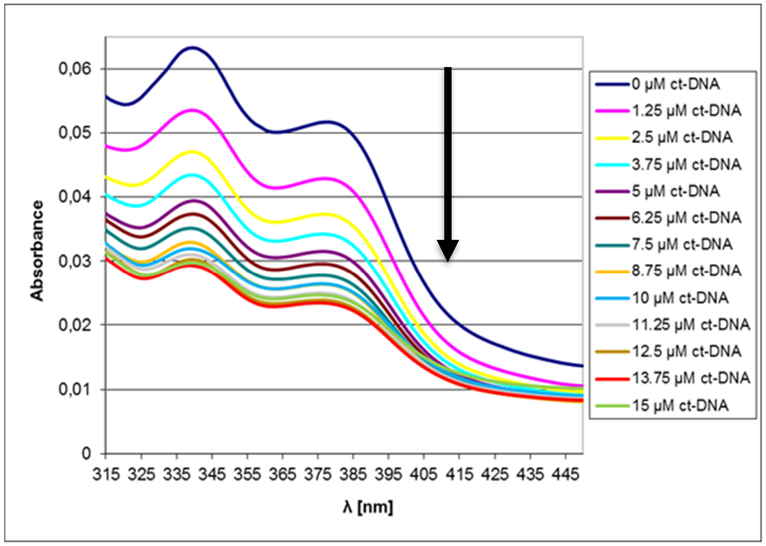


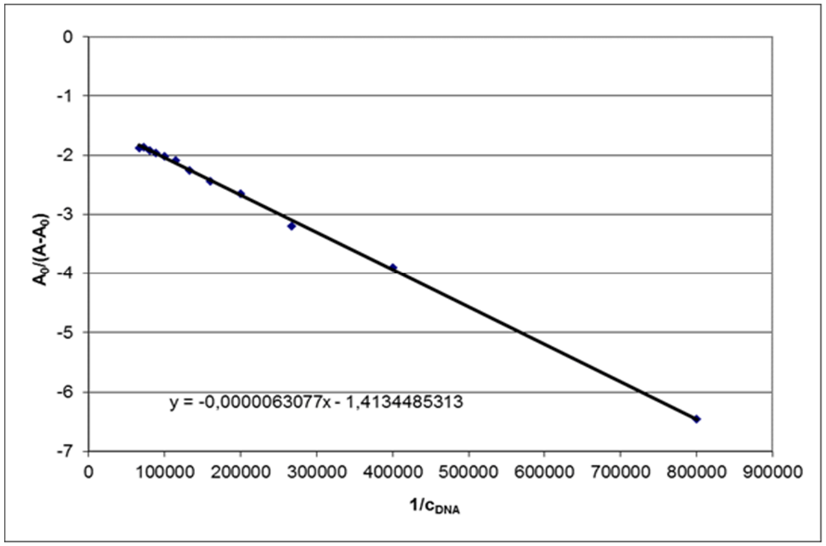


**Figure S193**. UV-vis absorption spectra of compound **18** (20 μM) in the presence of increasing amount of ct-DNA (0-15 μM) (left). The plot of $A_0/A-A_0$ versus 1/[DNA] yielded the binding constant (right).

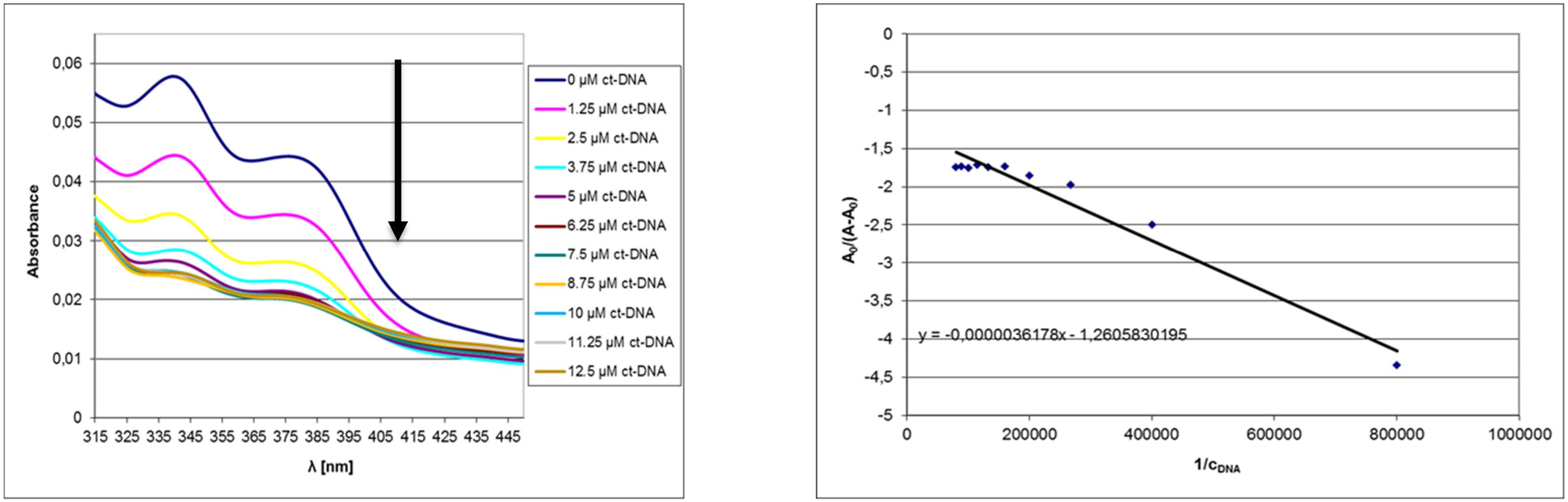


**Figure S194**. UV-vis absorption spectra of compound **19** (20 µM) in the presence of increasing amount of ct-DNA (0-15 µM) (left). The plot of $A_0/A-A_0$ versus 1/[DNA] yielded the binding constant (right).

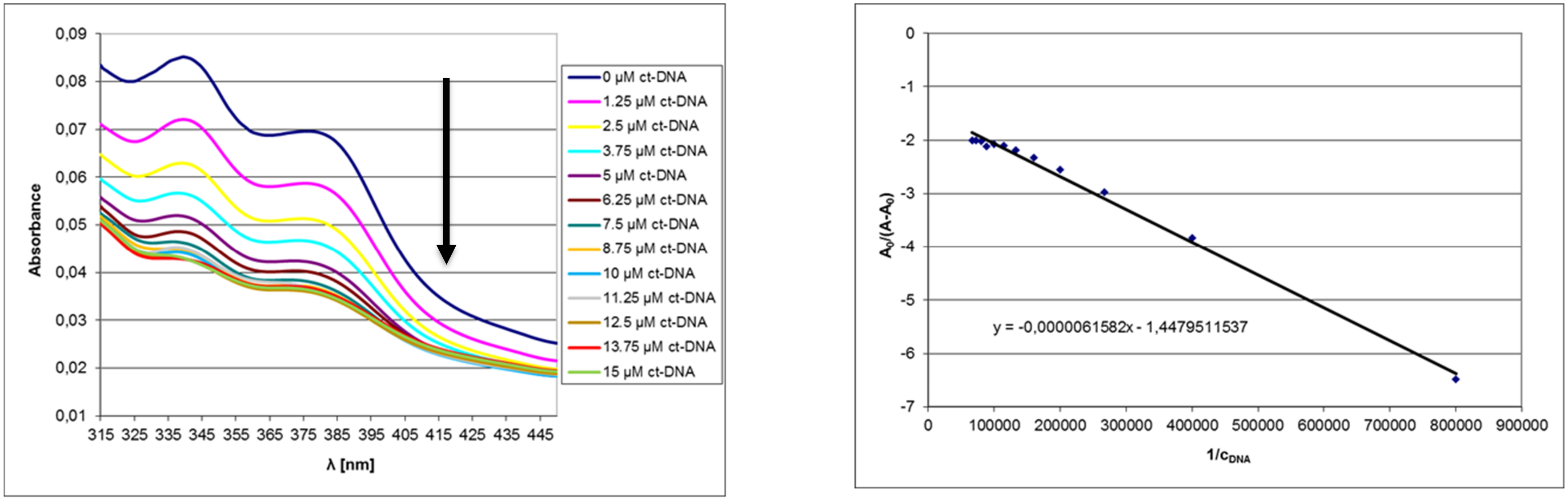


**Figure S195**. UV-vis absorption spectra of compound **20** (20 µM) in the presence of increasing amount of ct-DNA (0-15 µM) (left). The plot of $A_0/A-A_0$ versus 1/[DNA] yielded the binding constant (right).

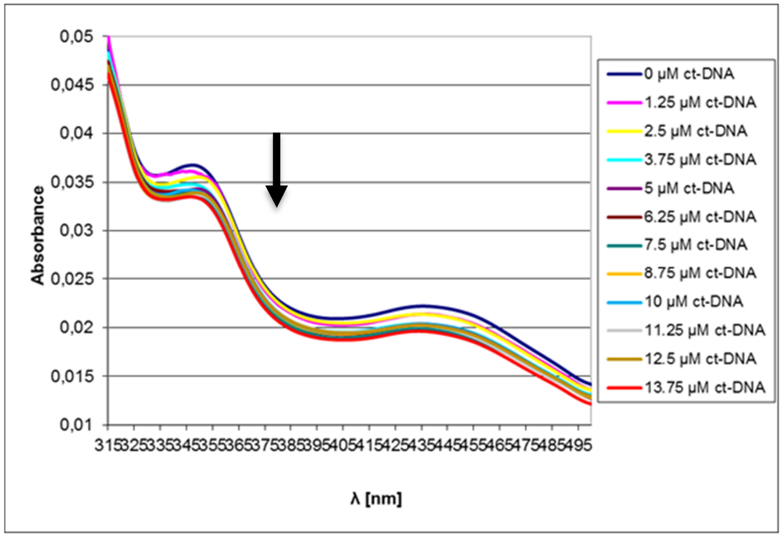


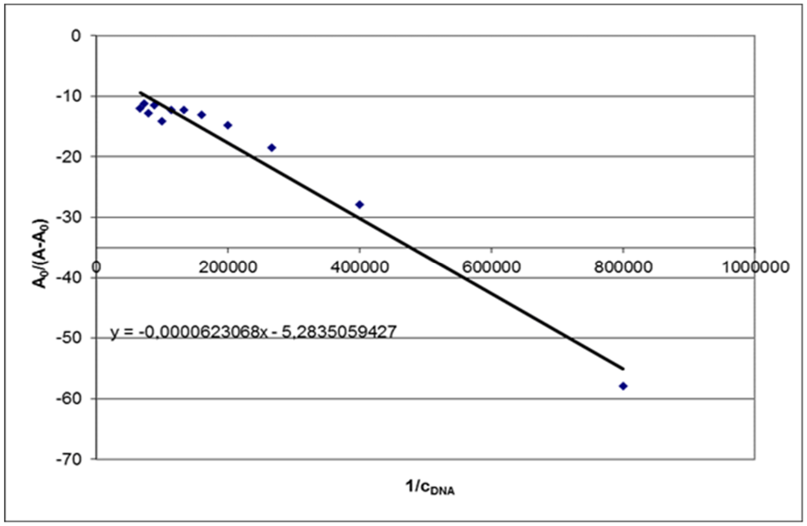


**Figure S196**. UV-vis absorption spectra of compound **33** (20 μM) in the presence of increasing amount of ct-DNA (0-15 μM) (left). The plot of $A_0/A-A_0$ versus 1/[DNA] yielded the binding constant (right).

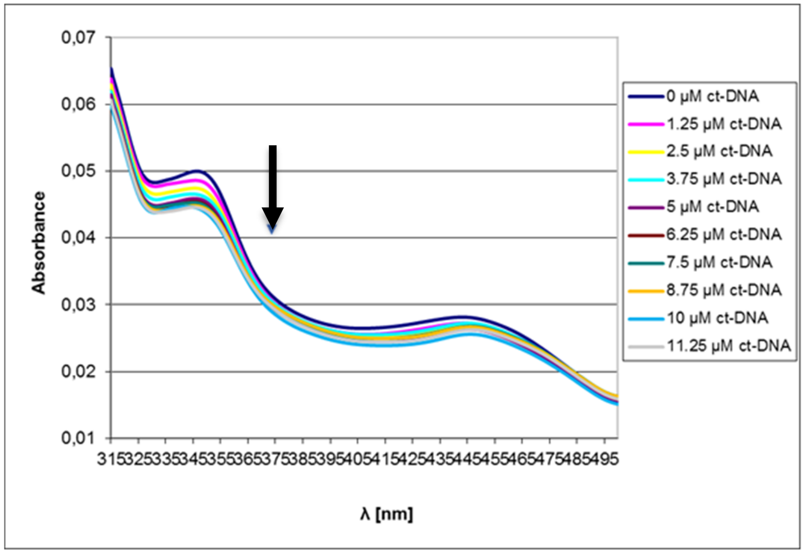


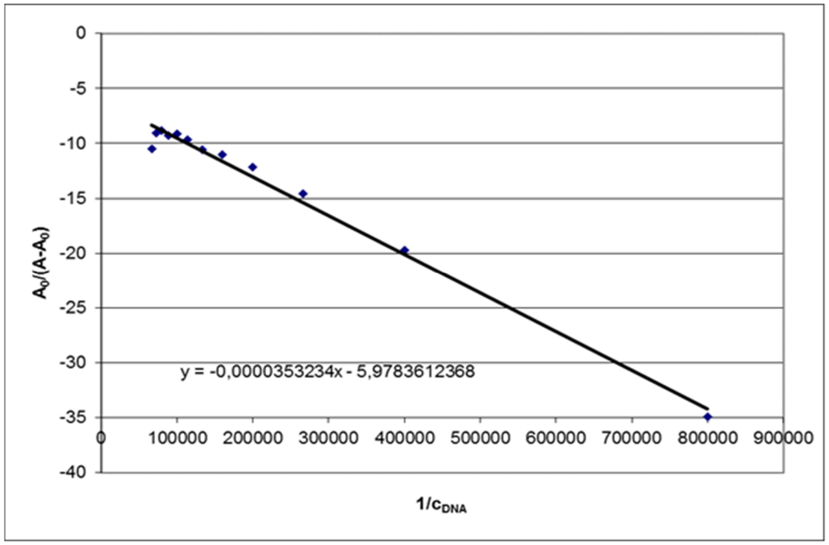


**Figure S197**. UV-vis absorption spectra of compound **34** (20 µM) in the presence of increasing amount of ct-DNA (0-15 µM) (left). The plot of $A_0/A-A_0$ versus 1/[DNA] yielded the binding constant (right).

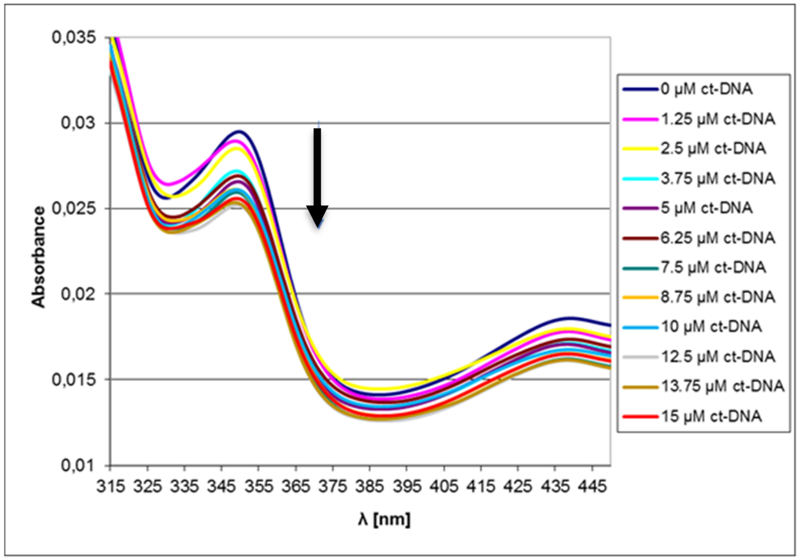


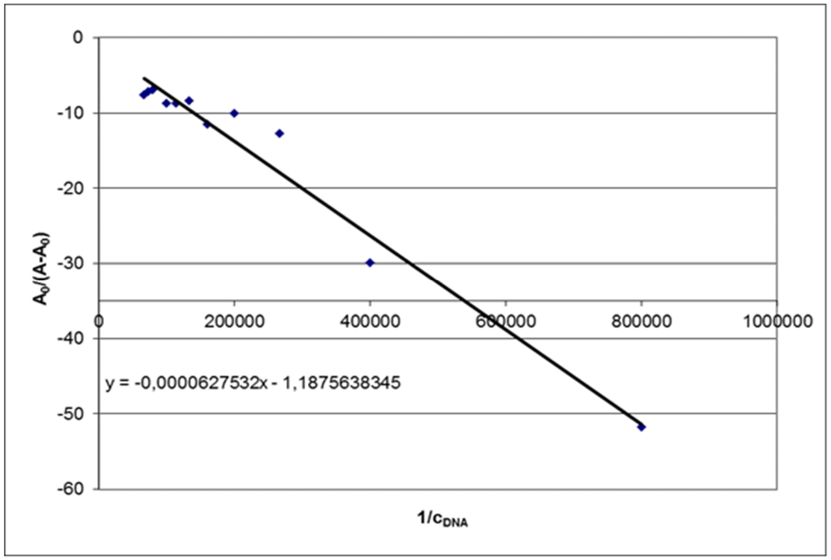


**Figure S198**. UV-vis absorption spectra of compound **35** (20 μM) in the presence of increasing amount of ct-DNA (0-15 μM) (left). The plot of $A_0/A-A_0$ versus 1/[DNA] yielded the binding constant (right).

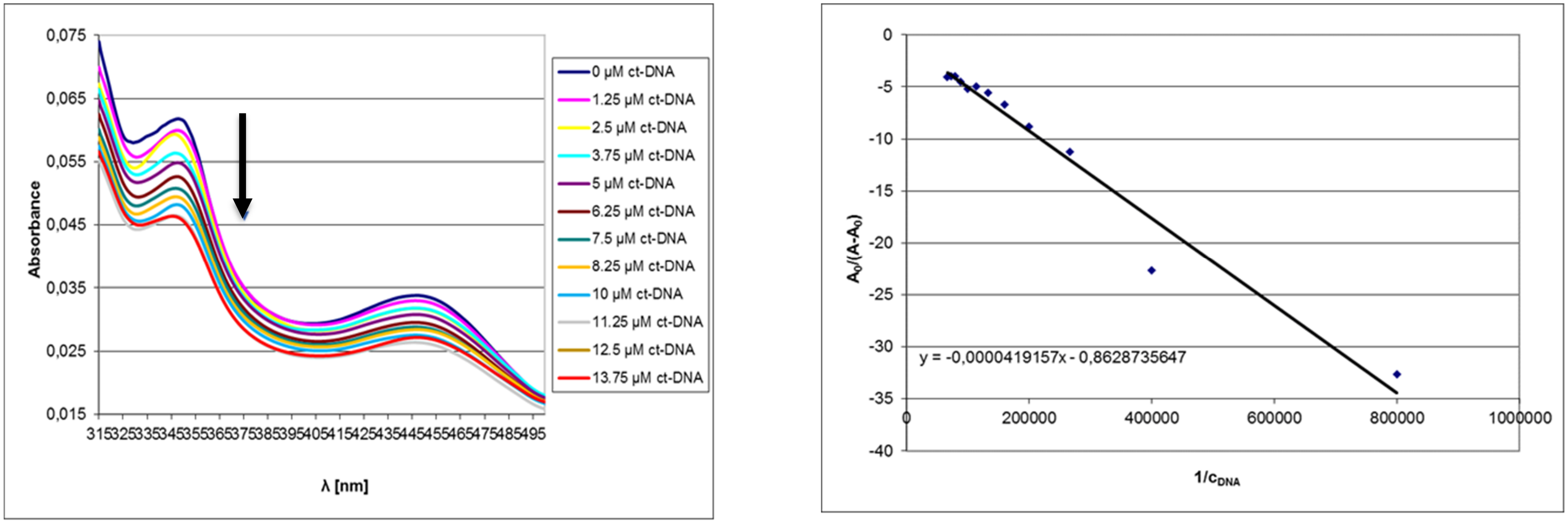


**Figure S199**. UV-vis absorption spectra of compound **36** (20 μM) in the presence of increasing amount of ct-DNA (0-15 μM) (left). The plot of $A_0/A-A_0$ versus 1/[DNA] yielded the binding constant (right).

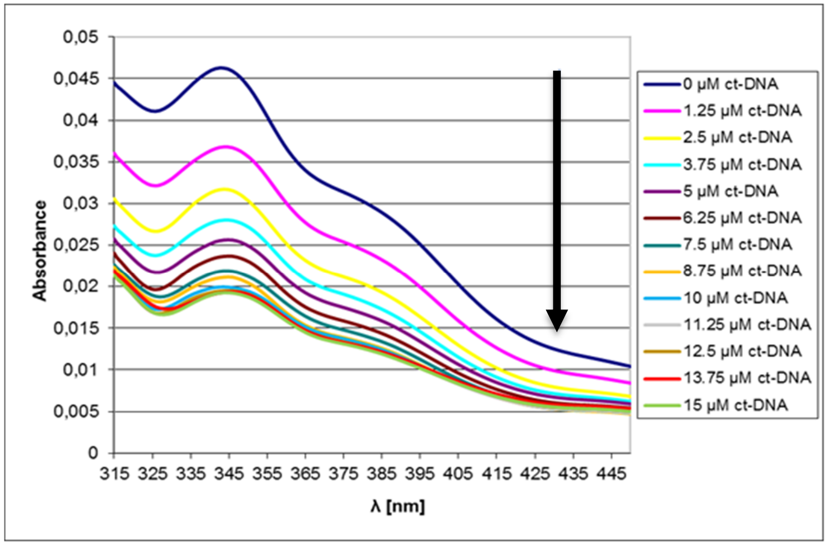


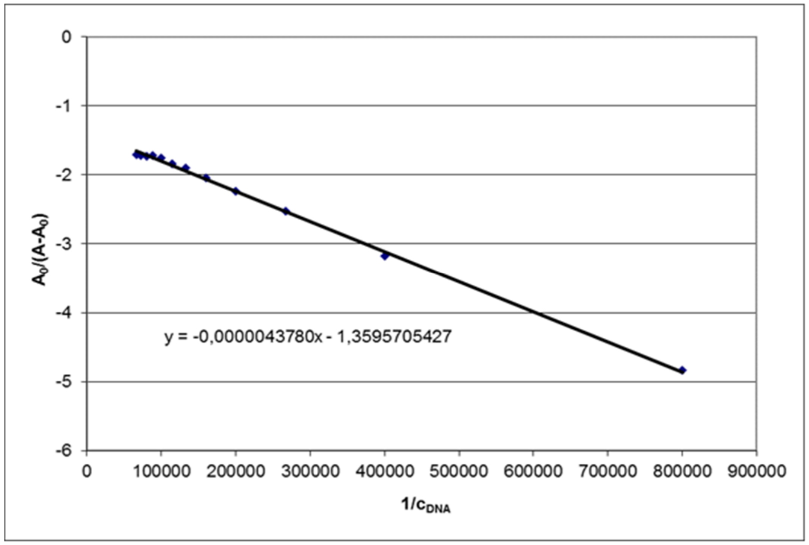


**Figure S200**. UV-vis absorption spectra of compound **39** (20 μM) in the presence of increasing amount of ct-DNA (0-15 μM) (left). The plot of $A_0/A-A_0$ versus 1/[DNA] yielded the binding constant (right).

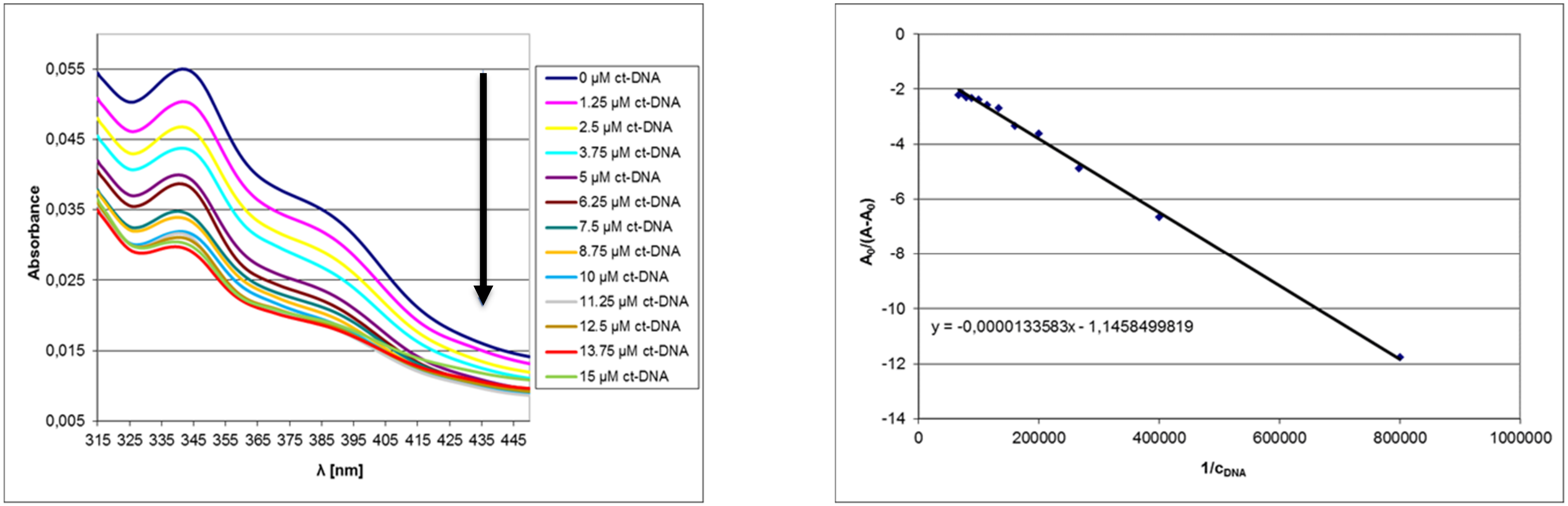


**Figure S201**. UV-vis absorption spectra of compound **40** (20 μM) in the presence of increasing amount of ct-DNA (0-15 μM) (left). The plot of $A_0/A-A_0$ versus 1/[DNA] yielded the binding constant (right).

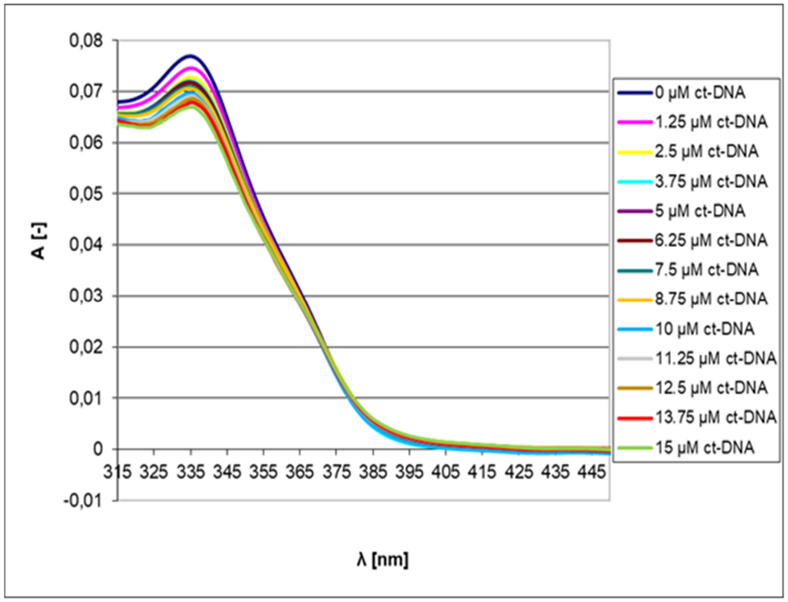


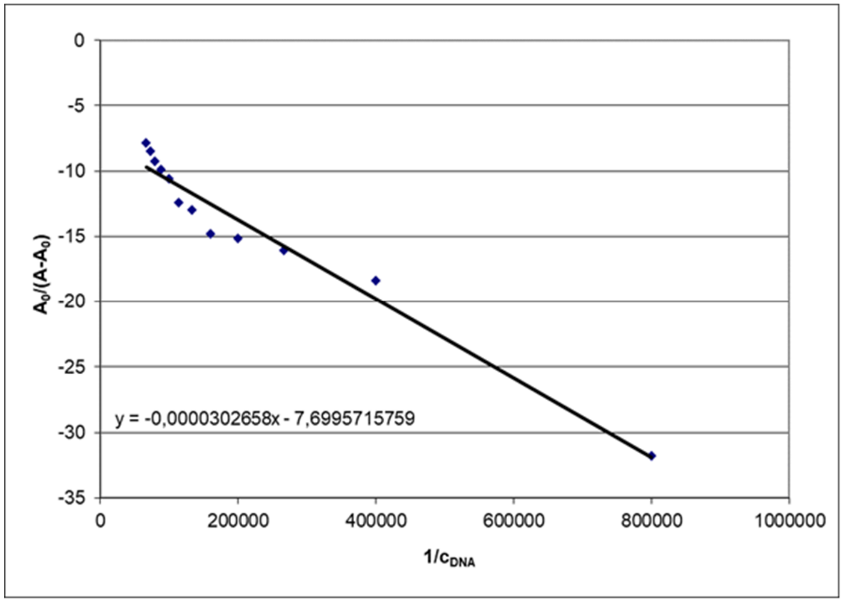


**Figure S202**. UV-vis absorption spectra of mitonafide (20 μM) in the presence of increasing amount of ct-DNA (0-15 μM) (left). The plot of $A_0/A-A_0$ versus 1/[DNA] yielded the binding constant (right).

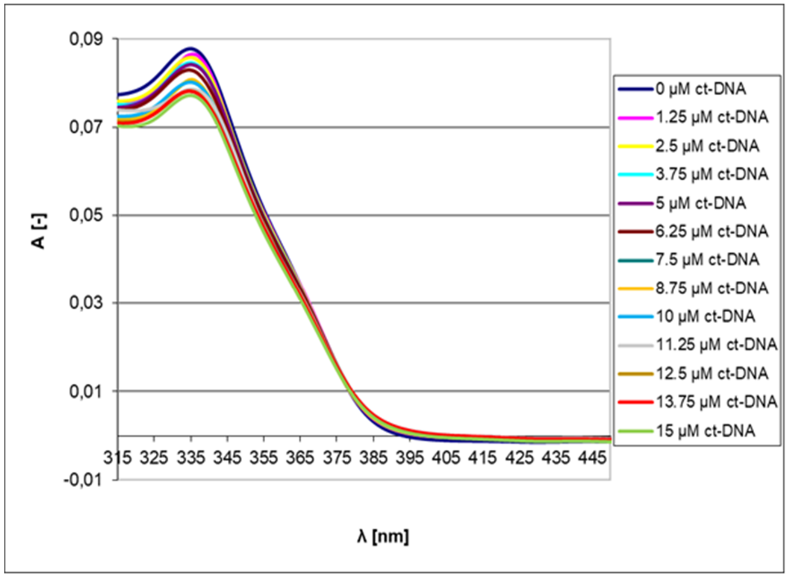


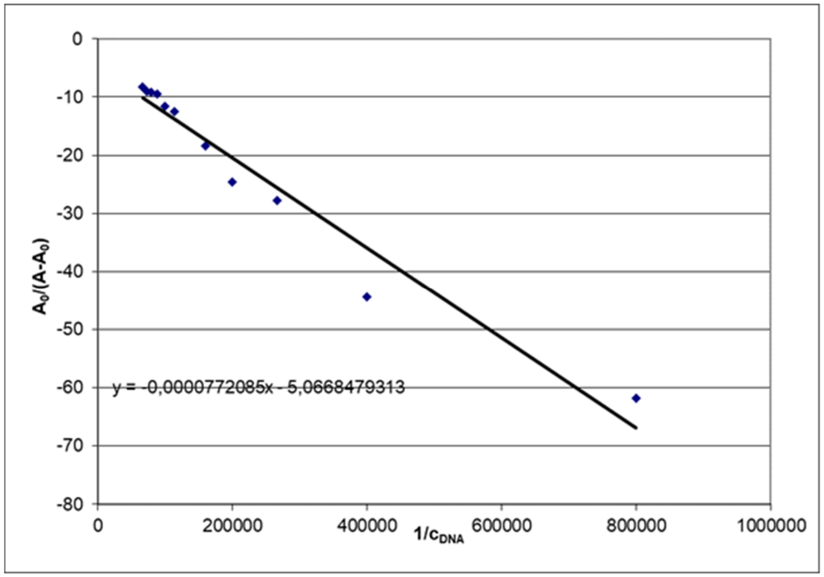


**Figure S203**. UV-vis absorption spectra of pinafide (20 μM) in the presence of increasing amount of ct-DNA (0-15 μM) (left). The plot of $A_0/A-A_0$ versus 1/[DNA] yielded the binding constant (right).

**Table 2S**. Crystallographic data.

| Compound | **39** | **41** |
|---|---|---|
| Wavelength [Å] | 0.7749 | 1.54178 |
| Temperature [K] | 100 | 130 |
| Space group | P-1 | P-1 |
| Z | 2 | 2 |
| *a* [Å] | 6.923(2) | 7.0385(8) |
| *b* [Å] | 13.239(8) | 9.5223(8) |
| *c* [Å] | 15.571(3) | 21.4413(17) |
| *α* [°] | 105.721(0) | 79.497(7) |
| *β* [°] | 102.188(3) | 81.569(8) |
| *γ* [°] | 95.815(0) | 87.093(8) |
| $R_{int}$ | 0.0550 | 0.0892 |
| Resolution [Å] | 0.79 | 0.79 |
| % completeness | 74.3 | 94.8 |
| Independent reflections | 4262 | 5628 |
| R/R(for $F_o>4\sigma$) | 0.0840/0.1106 | 0.1012/0.1755 |
| CSD code | 2059432 | 2059431 |
